# A New Generation of Arachidonic Acid Analogues as Potential Neurological Agent Targeting Cytosolic Phospholipase A_2_

**DOI:** 10.1038/s41598-017-13996-8

**Published:** 2017-10-20

**Authors:** Cheng Yang Ng, Srinivasaraghavan Kannan, Yong Jun Chen, Francis Chee Kuan Tan, Wee Yong Ong, Mei Lin Go, Chandra S. Verma, Chian-Ming Low, Yulin Lam

**Affiliations:** 1 Department of Chemistry, National University of Singapore, 3 Science Drive 3, Singapore, 117543 Singapore; 2 Bioinformatics Institute, Agency for Science, Technology and Research (A*STAR), Matrix, Singapore, 138671 Singapore; 3 Department of Pharmacy, National University of Singapore, 21 Lower Kent Ridge Road, Singapore, 119077 Singapore; 4 Department of Anaesthesia, Yong Loo Lin School of Medicine, National University of Singapore, 5 Lower Kent Ridge Road, Singapore, 119074 Singapore; 5 Department of Pharmacology, Yong Loo Lin School of Medicine, National University of Singapore, 16 Medical Drive, Singapore, 117600 Singapore

## Abstract

Cytosolic phospholipase A_2_ (cPLA_2_) is an enzyme that releases arachidonic acid (AA) for the synthesis of eicosanoids and lysophospholipids which play critical roles in the initiation and modulation of oxidative stress and neuroinflammation. In the central nervous system, cPLA_2_ activation is implicated in the pathogenesis of various neurodegenerative diseases that involves neuroinflammation, thus making it an important pharmacological target. In this paper, a new class of arachidonic acid (AA) analogues was synthesized and evaluated for their ability to inhibit cPLA_2_. Several compounds were found to inhibit cPLA_2_ more strongly than arachidonyl trifluoromethyl ketone (AACOCF_3_), an inhibitor that is commonly used in the study of cPLA_2_-related neurodegenerative diseases. Subsequent experiments concluded that one of the inhibitors was found to be cPLA_2_-selective, non-cytotoxic, cell and brain penetrant and capable of reducing reactive oxygen species (ROS) and nitric oxide (NO) production in stimulated microglial cells. Computational studies were employed to understand how the compound interacts with cPLA_2_.

## Introduction

Phospholipases A_2_ (PLA_2_s) are a superfamily of enzymes characterized by their ability to hydrolyze the ester bond at the *sn*-2 position of glycerophospholipids to yield polyunsaturated fatty acids (PUFAs) and a lysophospholipid^1^. The mammalian PLA_2_ family comprises six main types of enzymes, namely the secreted small molecular weight sPLA_2_, the larger cytosolic Ca^2+^-dependent cPLA_2_, the Ca^2+^-independent iPLA_2_, the platelet-activating factor acetylhydrolases PAF-AH and the lysosomal PLA_2_. Of these PLA_2_ enzymes, the ubiquitous cPLA_2_ is found to be responsible for the release of arachidonic acid (AA), an important PUFA which serves as the precursor for the synthesis of eicosanoids and prostanoids that mediate a wide variety of inflammatory responses^2^. In the central nervous system, cPLA_2_ activation has been implicated in the pathogenesis of a number of neurodegenerative (Alzheimer’s disease, Parkinson’s disease and prion diseases) and neuropsychiatric (schizophrenia and depressive disorder) diseases^3–7^. Effects of neuroinflammation and its involvement in nitrosative/oxidative signaling pathways for example have been frequently cited as a pathogenetic link to Aβ-amyloid production due to the actions of cPLA_2_^8^. A direct effect of sustained and aberrant cPLA_2_ activation could result in enhanced neural membrane destruction eventually compromising functions on membrane ion receptors, leading to cell death^3–7^. Thus cPLA_2_ is an important target for drug discovery, and for the development of new therapeutics to treat neurodegenerative and neuropsychiatric disorders. An ideal cPLA_2_ inhibitor should not only reach the site where inflammatory processes are taking place (this may be achieved through improved drug delivery systems), they should also inhibit inflammation, block oxidative stress and cross the blood-brain barrier (BBB). To-date, various compounds have been shown to inhibit cPLA_2_ selectively^9–16^. However many of these compounds have not yet been shown to possess brain penetrability^17,18^. Thus as part of our efforts to develop cPLA_2_ inhibitors as potential drug candidates for the treatment of neurological disorders, we have synthesized a new series of AA analogues **1**–**2** and evaluated them for their PLA_2_ inhibitory activities and ability to cross the BBB. In our inhibitor design, we have focused on the arachidonyl scaffold as earlier studies have shown that this pharmacophore is strongly recognized by cPLA_2_^19–23^. We herein present the synthesis of AA analogues **1**–**2** and the investigation of these compounds for their (i) inhibition of cPLA_2_, (ii) cytotoxicity, (iii) selectivity and anti-neuroinflammatory properties and (iv) ability to cross the blood-brain barrier; finally computational studies were performed to understand how the compounds bind to cPLA_2_.

## Results and Discussion

### Chemistry

**Figure 1:**
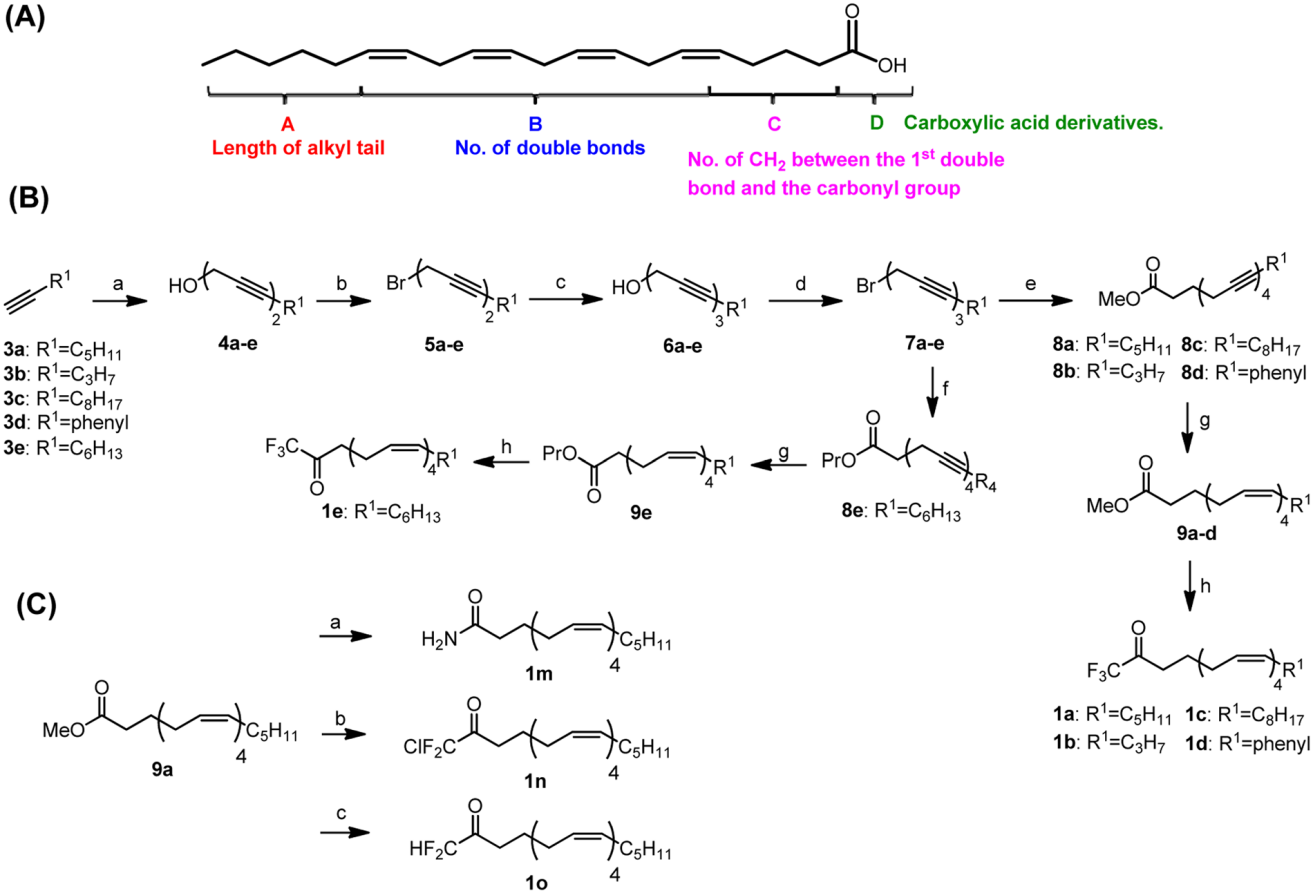
(**A**) Functionalities on AA that could be varied. (**B**) Reagent and conditions: (a) 4-Chloro-2-butyn-1-ol, CuI, NaI, K_2_CO_3_, DMF, rt, overnight (b) CBr_4_, PPh_3_, CH_2_Cl_2_, −40 °C to −20 °C, 1 h (c) Propargyl alcohol, CuI, NaI, K_2_CO_3_, DMF, rt, overnight (d) CBr_4_, PPh_3_, CH_2_Cl_2_, −40 °C to −20 °C, 1 h (e) Methyl 6-hexynoate, CuI, NaI, K_2_CO_3_, DMF, rt, overnight (f) Propyl 5-pentynoate, CuI, NaI, K_2_CO_3_, DMF, rt, overnight (g) H_2_, Ni(OAc)_2_.4H_2_O, NaBH_4_, en, 95% EtOH, rt, 2 h (h) i) NaOH, MW 120 °C, 1 h ii) Trifluoroacetic anhydride, Pyridine, CH_2_Cl_2_, rt, 2 h (**C**) (a) NH_3_, Mg(OMe)_2_, MeOH, 80 °C, overnight (b) i) NaOH, MW 120 °C, 1 h ii) Chlorodifluoroacetic anhydride, Pyridine, CH_2_Cl_2_, rt, overnight (c) i) NaOH, MW 120 °C, 1 h ii) Difluoroacetic anhydride, Pyridine, CH_2_Cl_2_, rt, overnight.

Using AA as a scaffold, our inhibitor design involved modification at different selected positions of the compound (Fig. 1A). In particular, the carboxylic acid functionality could be modified to a trifluoromethylketone group to form the well-known cPLA_2_ inhibitor, AACOCF_3_. Being cell penetrant, AACOCF_3_ has been effective in reducing the undesirable effects of dysregulated cPLA_2_ systems, including those found in neurological diseases^9,24–26^. A series of compound with the CF_3_ moiety was synthesized and presented as compound **1**.

Compound **1** was prepared by assembling the alkynes together through a series of coupling and bromination reactions according to the synthetic strategy shown in Fig. 1B ^27–30^. A total of 3 coupling and 2 bromination reactions were performed to reach the skipped diynes **8a**–**e** (4 alkynes) and **10a–c** (3 alkynes). Compound **8a** was initially hydrogenated with Brown’s P2 nickel catalyst in ethanol according to a published procedure^27^. However this did not provide the desired compound **9a**. Optimization of the hydrogenation reaction by varying the equivalent of the catalyst and reaction conditions eventually provided **9a** in 68% yield (Supplementary Table S1). Initial attempts to trifluoromethylate **9a** by stirring TMSCF_3_ and CsF as a TMS activator with the ester in CHCl_3_ at room temperature did not yield **1a** (AACOCF_3_)^31^. Replacing CsF with TBAF also failed to provide the desired compound^32^. We attempted to convert **9a** to the corresponding carboxylic acid and then treating it with LDA and EtO_2_CCF_3_^33^. However this too did not yield **1a**. Finally we trifluoromethylated the intermediate carboxylic acid with pyridine and TFAA^34,35^ and gratifyingly this gave **1a** in 48% yield (over 2 steps). Analogues **1b**–**1h** were synthesized in the same manner by varying alkyne **3** in the first step of the reaction (Supplementary Scheme S1).

In the synthesis of **1i**–**l**, the precursor intermediates **13a–d** are not commercially available and were synthesized by treating alkyl halides **12a–d** with propargyl alcohol in the presence of butyllithium and an additive as a solvating agent. Initially *N*,*N’*-dimethylpropyleneurea (DMPU) was used as the additive but it gave **13b** in only 20% yield. Hence we tried hexamethylphosphoramide (HMPA) which provided **13b** in 86% yield. This may be attributed to the increased polarity of the P=O bond in HMPA as compared to the C=O in DMPU which stabilizes the acetylide to a greater extent. HMPA was thus used as an additive for the synthesis of intermediates **13a**–**d**. To form the primary amide **1m**, tetraene **9a** was amidated by heating it overnight at 80 °C in a solution of ammonia dissolved in methanol and Mg(OMe)_2_. The chlorodifluoroketo and difluoroketo functionalities in **1n** and **1o** were respectively synthesized by hydrolyzing the ester **9a** to the corresponding carboxylic acid followed by a reaction with the respective anhydrides in pyridine and dichloromethane at room temperature (Fig. 1C).

**Figure 2:**
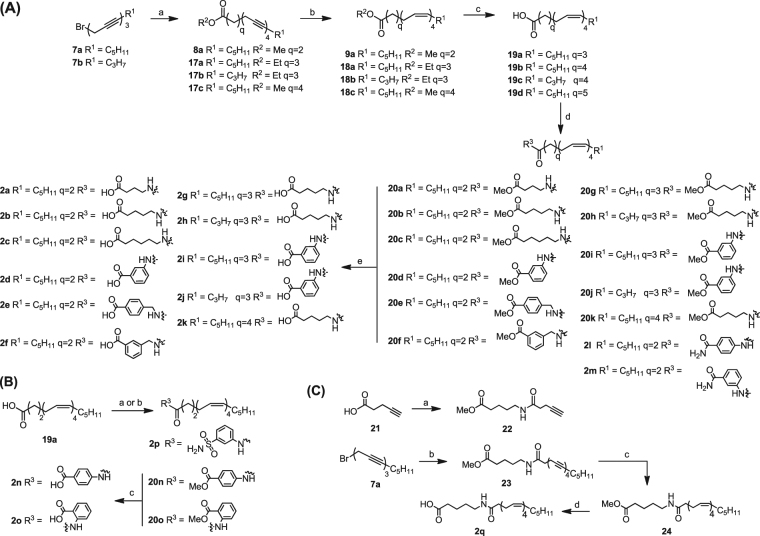
(**A**) Reagent and conditions: (a) CH≡C-(CH_2_)_q_CO_2_R^2^, CuI, NaI, K_2_CO_3_, DMF, rt, overnight (b) H_2_, Ni(OAc)_2_.4H_2_O, NaBH_4_, en, 95% EtOH, rt, 2 h (c) MeOH/NaOH, rt, 3 h (d) NH_2_-Alk-CO_2_Me or NH_2_-Ar-CO_2_Me or NH_2_-Ar-CONH_2_, EDC.HCl, HOBt, TEA, DMF, rt, overnight e) MeOH/NaOH, rt, 3 h. (**B**) Reagent and conditions: (a) 3-aminobenzenesulfonamide, EDC.HCl, TEA, MeCN, 4 °C, overnight (b) i) (COCl_2_), catalytic DMF, CH_2_Cl_2_, rt, 1 h ii) NH_2_-Ar-CO_2_Me, TEA, THF, rt, overnight (c) MeOH/NaOH, rt, 3 h. (**C**) Reagent and conditions: (a) Methyl 5-aminopentanoate hydrochloride, EDC.HCl, HOBt, TEA, DMF, rt, overnight (b) **22**, CuI, NaI, K_2_CO_3_, DMF, rt, overnight (c) H_2_, Ni(OAc)_2_.4H_2_O, NaBH_4_, en, 95% EtOH, rt, 2 h (d) MeOH/NaOH, rt, 3 h.

Compound **2** differs from **1** as it contains an extension from the carbonyl amide. **2** was synthesized by first coupling the skipped dyne **7a** and **7b** with the respective alkynes to form **8a** and **17a–c** (Fig. 2). Subsequent hydrogenation and ester hydrolysis gave the corresponding carboxylic acids **19a**–**d** which were then coupled to the appropriate amines using EDC.HCl, HOBt, and TEA to give **20a**–**20k**, **2l**–**2m**. Coupling of **19a** with 3-aminobenzenesulfonamide give **2p** under EDC.HCl and TEA coupling condition (Fig. 2B). To synthesize **2n** and **2o**, the acid **19a** had to be converted into an acid chloride with oxalyl chloride before reacting it with the respective amines to form **20n** and **20o**.This was attributed to the conjugative electron-withdrawing effects from the methyl ester on the benzene ring, resulting in a decrease in electron density of the amine which in turn affected its reactivity. As a result, a harsher amidation condition was required. **20n** and **20o** were then hydrolyzed to the free acid **2n** and **2o** in a similar manner (Fig. 2B). **2q** was synthesized by coupling **7a** with **22** followed by hydrogenation and hydrolysis (Fig. 2C).

### Testing of inhibitor compounds using cPLA_2_ assay

Table 1 shows the inhibitory activities of **1** and **2** on cPLA_2_. The compounds were evaluated with a fluorogenic PLA_2_ assay kit (EnzChek® Phospholipase A_2_ Assay Kit, Catalog No: E10217) at a standard 10 μM concentration. Potential inhibitors were determined by their ability to lower cPLA_2_ activity to a greater extent than **1a**.

**Table 1:** Inhibitory activities of **1** and **2** on cPLA_2_ at 10 μM and their physiochemical properties.

	
Cpd	R^2^	m	n	R^1^	% cPLA_2_ Activity	% Inhibition	PSA^a^	Cpd	R^3^	q	R^1^	% cPLA_2_ Activity	% Inhibition	PSA^a^
**1a**	CF_3_	2	4	C_5_H_11_	40.0 ± 0.9	60.0	17.1	**2a**		2	C_5_H_11_	39.7 ± 2.6	60.3	66.4
**1b**	CF_3_	2	4	C_3_H_7_	37.0 ± 3.9	63.0	17.1	**2b**		2	C_5_H_11_	26.7 ± 0.1	73.3	66.4
**1c**	CF_3_	2	4	C_8_H_17_	45.0 ± 3.5	55.0	17.1	**2c**		2	C_5_H_11_	40.9 ± 1.1	59.1	66.4
**1d**	CF_3_	2	4	Ph	31.8 ± 2.0	68.2	17.1	**2d**		2	C_5_H_11_	13.2 ± 1.2	86.8	66.4
**1e**	CF_3_	1	4	C_6_H_13_	40.5 ± 3.6	59.5	17.1	**2e**		2	C_5_H_11_	25.8 ± 0.1	74.2	66.4
**1f**	CF_3_	2	3	C_5_H_11_	33.8 ± 2.2	66.2	17.1	**2f**		2	C_5_H_11_	35.6 ± 2.1	64.4	66.4
**1g**	CF_3_	2	3	C_3_H_7_	42.5 ± 0.3	57.5	17.1	**2g**		3	C_5_H_11_	15.5 ± 0.3	84.5	66.4
**1h**	CF_3_	2	3	C_8_H_17_	45.6 ± 2.8	54.4	17.1	**2h**		3	C_3_H_7_	43.6 ± 0.2	56.4	66.4
**1i**	CF_3_	2	2	C_5_H_11_	36.4 ± 3.3	63.6	17.1	**2i**		3	C_5_H_11_	11.5 ± 1.0	88.5	66.4
**1j**	CF_3_	2	2	C_7_H_15_	33.9 ± 4.8	66.1	17.1	**2j**		3	C_3_H_7_	16.5 ± 1.2	83.5	66.4
**1k**	CF_3_	2	2	C_11_H_23_	54.0 ± 6.9	46.0	17.1	**2k**		4	C_5_H_11_	37.7 ± 1.5	62.3	66.4
**1l**	CF_3_	2	2	C_13_H_27_	72.4 ± 12.9	27.6	17.1	**2l**		2	C_5_H_11_	67.4 ± 5.7	32.6	72.2
**1m**	NH_2_	2	4	C_5_H_11_	60.7 ± 1.9	39.3	43.1	**2m**		2	C_5_H_11_	57.5 ± 1.4	42.5	72.2
**1n**	CClF_2_	2	4	C_5_H_11_	49.2 ± 4.5	50.8	17.1	**2n**		2	C_5_H_11_	33.3 ± 0.9	66.7	66.4
**1o**	CHF_2_	2	4	C_5_H_11_	40.2 ± 0.5	59.8	17.1	**2o**		2	C_5_H_11_	65.5 ± 4.0	34.5	66.4
								**2p**		2	C_5_H_11_	53.9 ± 2.7	46.1	89.3
								**2q**		1	C_5_H_11_	56.6 ± 4.9	43.4	66.4
								**20c**		2	C_5_H_11_	84.8 ± 1.0	15.2	55.4

^a^Values were obtained from ChemBioDraw Ultra 12.

#### Modification of the tail length

From Table 1 (Cpd **1a**–**1c** and **1i**–**1l**), varying the tail length did not provide a significant improvement in cPLA_2_ inhibitory activity as compared to AACOCF_3_. In fact, increasing the alkyl chain length from C5 (**1i**) to C13 (**1l**) resulted in a significant decrease in cPLA_2_ inhibition. Replacing the alkyl tail with a phenyl moiety provided **1d** which showed the highest cPLA_2_ inhibitory activity in the trifluoromethylketone series of compounds. This could be attributed to the additional interactions with cPLA_2_ (*vide infra*).

#### Modification of number of double bonds

The skipped alkene functionality in the arachidonyl backbone causes **1a** to be light and oxygen sensitive^36^. We hypothesized that reducing the number of skipped alkenes on the backbone could improve the compound’s stability. Hence analogues with a reduced number of double bonds (Table 1 Cpd **1f**–**l**) were synthesized (Fig. 1B,C). However the potencies of these compounds were found to be lower than that of **1a**, indicating that it was more favourable for analogues with the same number of carbons to contain more double bonds (Table 1, Cpd **1a**, **1h** and **1k**). A plausible explanation could be due to the increased rigidity of the compound with more double bonds, making them less rotatable and hence improving their affinity for the enzyme pocket.

#### Modification of the number of methylene groups between the first double bond and the carbonyl group

Earlier studies have shown that the number of methylene groups between the carbonyl carbon and the first double bond (Fig. 1A) is important for enzyme recognition^37^. To better understand the effects of these methylene groups on the inhibitory properties of the compound, **1e** with two methylene groups was synthesized. Results obtained showed that **1e** had the same cPLA_2_ inhibitory activity as **1a** (Table 1) suggesting that the position of the double bonds from the carbonyl for enzyme recognition may not be as important as it was previously postulated^37^.

#### Modification of the functionality on the carbonyl group

Earlier studies have shown that the presence of a trifluoromethylketo moiety confers a very high electrophilicity on AACOCF_3_^38^. This can result in the keto existing largely in the hydrated form, thus reducing the concentration of the active species. To vary the electrophilicity of the carbonyl group, we modulated the number of fluorine atoms or replaced the trifluoro moiety with other functionalities (Table 1, Cpd **1m**–**1o**). However, reducing the electrophilicity of the carbonyl group by reducing the number of fluorine atoms on the trifluoromethyl moiety did not result in a more potent cPLA_2_ inhibitor (Table 1, Cpd **1n**–**1o**). Similarly a decrease in cPLA_2_ inhibitory activity was observed in **1m** when the carbonyl was converted into a primary amide. However all the compounds in Table 1 have the same polar surface area (17.1 Å^2^) except **1m** (43.1 Å^2^). Since the polar surface area measures the compound’s ability to permeate cells, we decided to explore other analogues of **1m** by functionalizing the amide moiety^39–41^.

Pickard *et al*.^42^ have shown that site-directed mutagenesis of Arg200 in cPLA_2_ severely affected its catalytic property while molecular docking and crystallography studies by other groups have indicated that Arg200 plays a stabilizing role by interacting with the phosphate group on phosphatidylcholine or the carboxylate groups on cPLA_2_ inhibitors^43,44^. Through a combination of deuterium exchange mass spectrometry and modeling, Burke and co-workers have also reported the interaction of Arg200 with cPLA_2_ inhibitors containing carboxyl groups^45^. We thus postulated that functionalizing the amide moiety of **1m** with a negatively charged moiety that interacts with Arg200 could improve the compound’s cPLA_2_ inhibitory activity. Hence various analogues containing an alkyl extension ending with either a carboxylic acid, ester, sulfonamide or amide functionality were synthesized (Fig. 2).

We started the investigation by synthesizing **2a**–**2c** which contained different number of methylene groups between the amide and carboxylic acid functionalities. **2b** was found to possess the best cPLA_2_ inhibitory activity amongst the three compounds (Table 1 Cpd **2a**–**2c**), suggesting that four methylene groups were optimal between the amide and carboxyl groups. To determine if the interaction between the carboxylic acid functionality and Arg200 was responsible for increasing the compound’s cPLA_2_ inhibitory activity, **20c** containing an ester instead of a carboxylic acid was also prepared. A significant decrease in activity was observed in **20c**, thus confirming the importance of the carboxylic acid moiety.

Next, we directed our efforts to determine the effect of the number of methylene groups between the first double bond and the carbonyl group. Although it was observed in our earlier studies of compound **1** that this effect is insignificant, the presence of a carboxyl-containing extension in **2** would result in a longer molecule than **1**, which could alter the interactions between **2** and the putative binding pocket in cPLA_2_. Comparison of the activities of **2b**, **2g**, **2k** and **2q** (Table 1) showed that cPLA_2_ inhibition improved when the number of methylene groups was increased, with four methylene groups (**2g**) between the first double bond and the carbonyl group being the optimal number.

We have also explored rigidifying the compound by replacing the alkyl groups between the amide and carboxylic acid with a phenyl moiety. Three analogues containing a meta- (**2d**), para- (**2n**) and ortho- (**2o**) carboxylic acid were synthesized. **2d** exhibited the best cPLA_2_ inhibition, providing a 2.5-fold increase in activity. When the carboxylic acid moiety was replaced with an amide or sulfonamide (Table 1), lower cPLA_2_ inhibitory activities were observed.

Varying the alkyl tail of **2d** provided **2i** and **2j**. **2i** exhibited 88.5% cPLA_2_ inhibition and is the most potent compound found in this study. Hence from our structure-activity studies, three compounds, **2d**, **2g** and **2i** which significantly decreased the cPLA_2_ activity were identified. Since **2d** and **2i** are structurally analogous, only the stronger inhibitor **2i** was selected for further studies. The IC_50_ values of **2g** and **2i** were determined to be 5.3 ± 0.3 and 2.9 ± 0.2 μM respectively, indicating that these compounds display 3-fold and 5.5-fold stronger cPLA_2_ inhibition than **1a** (IC_50_ = 16.5 ± 3.0 μM).

### Cell survival test

**Figure 3:**
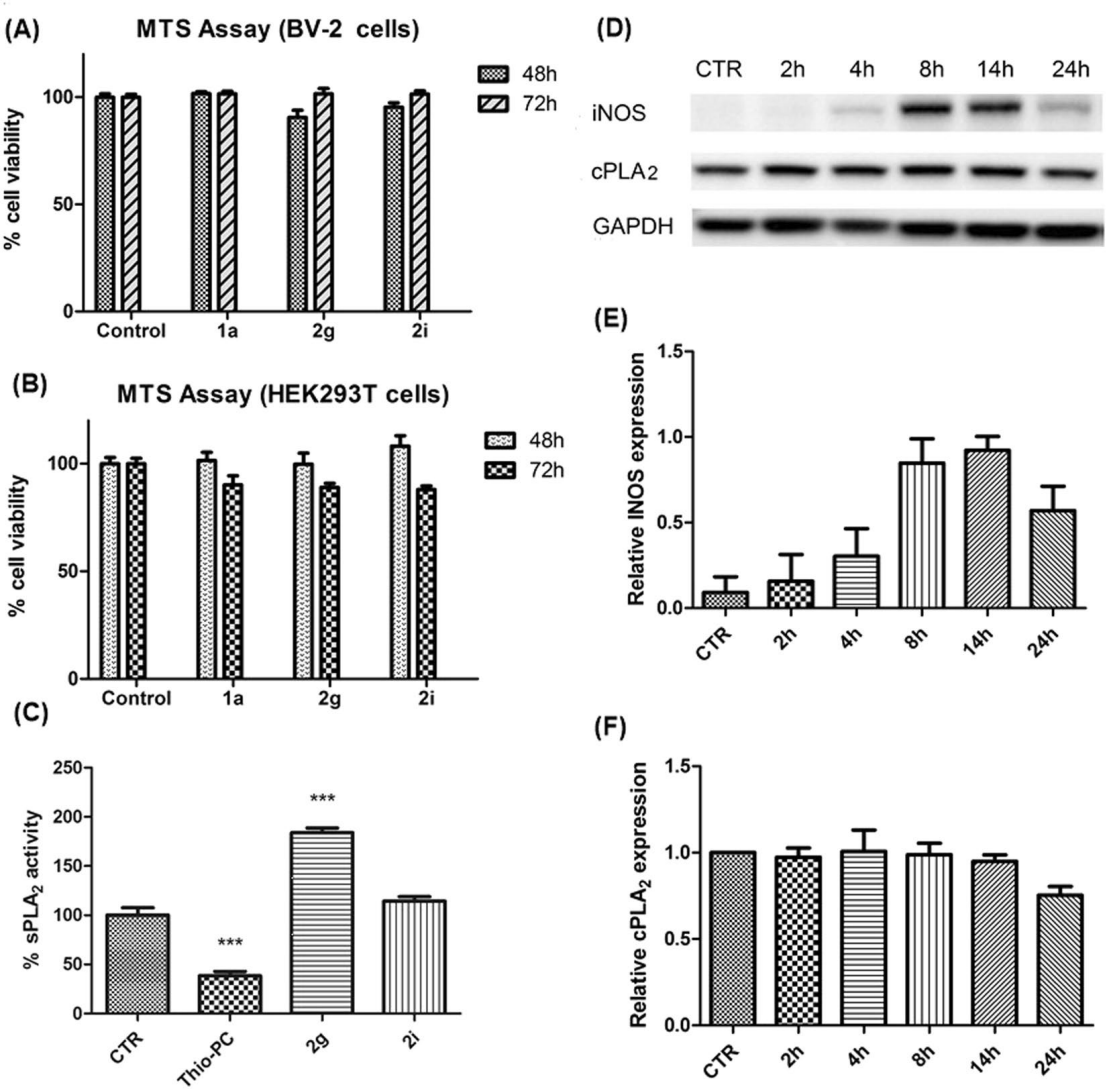
(**A**,**B**) MTS assay conducted on **1a**, **2g** and **2i** on BV-2 and HEK293T cells at different time points. Three independent biological repeat of cells were treated with 10 μM of the compound (final concentration). Data analysed by one-way ANOVA with Bonferroni’s Multiple Comparison Test against the Control set indicates no statistical differences in cell survival between the treated and control samples. (**C**) sPLA_2_ activity of the respective compounds at 10 μM. CTR refers to the DMSO control. Thio-PC refers to the set when sPLA_2_ selective inhibitor thioetheramide-PC is present. Data analysed by one-way ANOVA with Bonferroni’s Multiple Comparison Test against the CTR set indicates significant differences between DMSO control versus Thio-PC and **2g** (***P < 0.01). There is no significant difference between DMSO and **2i** indicating **2i** has no sPLA_2_ inhibitory activity. (**D–F**) Induction of iNOS expression in BV-2 cells after 2 μg/mL of LPS stimulation at different time points. BV-2 cells were collected and lysed at the respective time points and western blot was performed. (**D**) Representative western blot out of 3 independent experiments showing protein expression of iNOS, cPLA_2_ and GAPDH. Densitometry analysis of (**E**) iNOS and (**F**) cPLA_2_ after normalization with GAPDH. Uncropped images of blots can be found in Supplementary information Fig. S2.

To explore whether the inhibitors are cytotoxic, MTS assay were conducted on BV-2 cells, a murine microglial cell line derived from post-natal mouse cerebella, and HEK293T cells that are derived from human embryonic kidney. Both cell types were treated with 10 μM of **1a**, **2g** and **2i** for 48 h and 72 h (Fig. 3A,B). Dimethyl sulfoxide (DMSO) vehicle control was also included as the compounds were dissolved in DMSO. The amount of DMSO used in the assay was limited to 1% of the total volume to ensure that it does not result in cytotoxicity of cells. Both **2g** and **2i** showed no significant toxic effects on BV-2 and HEK293T cells as compared to vehicle control.

### sPLA_2_ assay

To further explore their selectivity towards cPLA_2_, 10 μM of **2g** and **2i** were evaluated for their inhibition against recombinant sPLA_2_ using the fluorogenic PLA_2_ assay (Fig. 3C). Thioetheramide-PC, a specific inhibitor of sPLA_2_ (IC_50_ = 2 μM)^46^ was used as a reference and produced a 62% reduction of sPLA_2_ activity. **2i** did not provide a significant change in sPLA_2_ activity, indicating that it could not inhibit sPLA_2_. However, **2g** showed a significant increase of sPLA_2_ activity to 184%, suggesting that it could possibly be a sPLA_2_ activator, thereby disqualifying it as a selective cPLA_2_ inhibitor. With the elimination of **2g**, only **2i** was assessed for its ability to oppose microglia activation via cPLA_2_ inhibition (there is a possibility of **2i** inhibiting iPLA_2_. However the purified enzymatically active form of iPLA_2_ is not commercially available. Hence in this study, we did not evaluate **2i** for its inhibitory activity against iPLA_2_. Such testing would be important in the future e.g. through the purification of iPLA_2_ from rat brain lysates to obtain active iPLA_2_^47^).

### LPS stimulation of BV-2 cells triggers iNOS production

**Figure 4:**
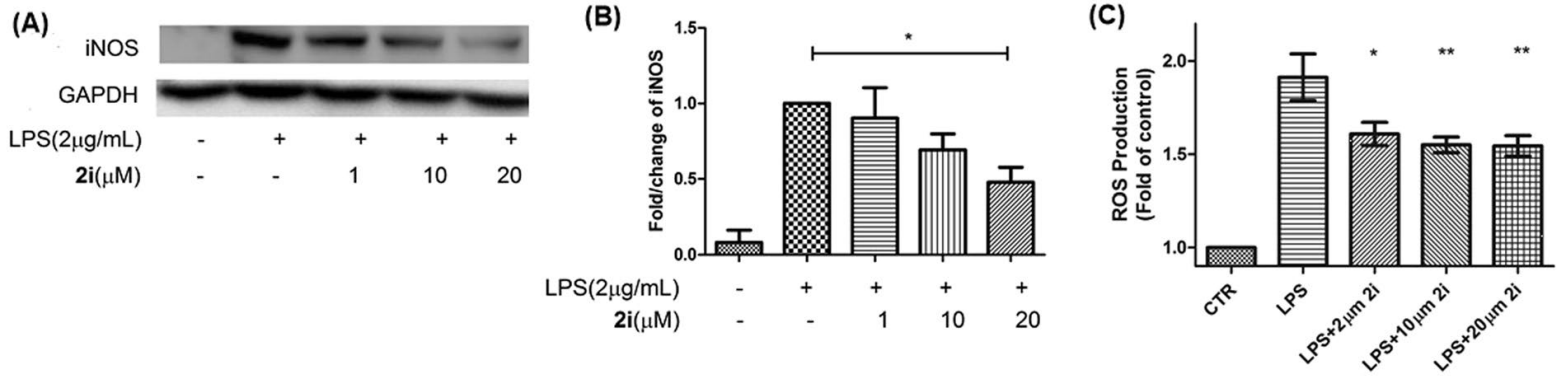
(**A**,**B**) Changes in iNOS protein expression in BV-2 cells after 14 h treatment with 2 μg/mL of LPS and different concentrations of **2i**. BV-2 cells were collected, lysed and western blot was performed. (**A**) Representative western blot out of 3 independent experiments showing protein bands of iNOS and GAPDH. (**B**) Densitometry analysis of iNOS after normalization with GAPDH. Data analysed by one-way ANOVA with Dunnett’s Multiple Comparison Test indicates significant difference between LPS treated versus LPS treated with 20 μM of **2i** (*P < 0.05). (**C**) **2i** reduces ROS production in BV-2 cells after a 14 h 2 μg/mL LPS insult. Different concentrations of **2i** were dissolved with LPS in culture medium and treated to the cells for 14 h. Five independent biological repeat of cells were conducted. Data analysed by one-way ANOVA with Bonferroni’s Multiple Comparison Test indicates significant difference when comparing between LPS-treated versus LPS-treated with different concentrations of **2i** (**P < 0.01, *P < 0.05). Uncropped images of blots can be found in Supplementary information Fig. S3.

It is well-established that reactive oxygen species (ROS) and nitric oxide (NO), produced during microglia activation, contribute to inflammatory response and cytotoxic damage to the surrounding neurons and neighbouring cells^48^. Recent studies have shown that BV-2 microglial cell could serve as an *in vitro* model to mimic such neuroinflammatory states when stimulated with lipopolysaccharide (LPS)^49^. LPS activates BV-2 cells by triggering a cascade of inflammatory events which includes the production of NO. This event is characterized by the generation of the biomarker, inducible nitric oxide synthase (iNOS), as well as ROS. Chuang *et al*.^50^ have also shown that cPLA_2_ is a major contributor in this pathway and inhibiting this enzyme reduces the production of the neurotoxic mediators and provide neuroprotective effects. Therefore to investigate the ability of **2i** in inhibiting cPLA_2_ in a neuroinflammatory model, BV-2 cells were treated with LPS (2 μg/mL), incubated for different time points, collected and lysed for protein extraction. Western blots conducted showed that iNOS level was highest between 8–14 h (Fig. 3D). Densitometry analyses showed that there were no statistical differences in the iNOS levels between the 8 and 14 h time points (Fig. 3E). The cPLA_2_ level was also determined and found to be largely constant throughout the entire activation process with a slight drop at the 24 h timepoint (Fig. 3F). Next, **2i** (1, 10 and 20 μM) were incubated together with LPS in the BV-2 cells for 14 h (Fig. 4A,B). The LPS-induced iNOS expression was significantly reduced by 50% when treated with 20 µM of compound **2i**. Varying the concentration of **2i** from 1–20 μM showed a dose-dependent decrease in the iNOS level, indicating that **2i** was capable of reducing iNOS production.

### ROS production

CM-H2DCFDA was used as the main detection reagent for ROS for this study. Being a chloromethyl derivative of the conventional H2DCFDA, it is better retained in the cells due to the reaction of its chloromethyl group with intracellular thiol species. In addition, esterase present in the cells would convert the acetate group found in CM-H2DCFDA into carboxylic acid, further retaining it in the cell. To investigate the ROS production by BV-2 cell stimulated with LPS, intracellular ROS was measured 14 h post-stimulation with CM-H2DCFDA fluorescence. A 2-fold increase in ROS was detected as compared to the control. When **2i** (2, 10 and 20 μM) were incubated with the BV-2 cells in the presence of 2 µg/mL LPS for 14 h, a dose-dependent decrease in ROS was observed (Fig. 4C), indicating **2i** could reduce ROS production under neurotoxic conditions. However, residual ROS were observed for samples treated with **2i**. This could be because the activation of ROS production might not be solely due to the inflammatory pathway mechanism involving cPLA_2_. It was reported in previous studies that a multitude of several other processes producing ROS may be at work^51,52^.

### *In vitro* evaluation of the blood-brain-barrier (BBB) permeation

A major requirement for the development of a successful drug for the treatment of central nervous system (CNS) disorder is its ability to pass through the BBB to reach the therapeutic target. Hence screening for its ability to penetrate the BBB is of great importance.

Earlier studies^53^ have demonstrated that the parallel artificial membrane permeation assay (PAMPA)^53^ assay provides good prediction of *in vivo* BBB permeability and is a useful tool to screen compounds for brain penetration. Thus to explore whether **2i** is able to penetrate into the brain, we used PAMPA with porcine brain lipids as the lipid barrier. Commercially available and highly potent cPLA_2_ inhibitors, CDIBA (an analogue of efipladib)^12^ and pyrrophenone, were also evaluated for their ability to penetrate the BBB. The effective permeability (P_e_) of **2i**, CDIBA and pyrrophenone were found to be 12.34 ± 1.46 × 10^−6^, 3.98 ± 0.24 × 10^−6^ and 2.00 ± 0.05 × 10^−6^ cm/s (16 h, 25 °C). P_e_ values of reference compounds determined under similar conditions were of the order propranolol > carbamazepine > quinidine > caffeine > dopamine, which agreed with reported literature^54,55^. A minimum P_e_ of 7 × 10^−6^ cm/s has been cited as the threshold for permeability across the blood brain barrier^56^. As the P_e_ of **2i** exceeded this value, we are optimistic that **2i** has the potential to transverse the BBB. We also found good aqueous solubility (>100 µM, 24 h, 25 °C) for **2i** at pH 7.4. Taken together, the promising physicochemical profile of **2i** warrants continued attention on this compound as an inhibitor of cPLA_2_.

### *In*-*silico* docking analysis

A docking study was performed to rationalize the inhibitory activities and to identify the possible binding sites of **2g** and **2i** on the cPLA_2_ enzyme. The crystal structure of cPLA_2_ in its apo form (PDB ID 1CJY, resolution 2.5 Å) and with a few missing regions was obtained from the protein data bank^57^. The missing regions were modelled and the complete structure was subjected to molecular dynamics (MD) simulations (as outlined in Methods). The complete model of cPLA_2_ remained stable during the simulation. The conformations sampled during the last 50 ns of the MD simulations were clustered into conformational sub-states using the Kclust program from the MMTSB tool set, with an rmsd of 2 Å set as cutoff. The cluster centroids of the top 5 most populated clusters were used for docking calculations.

**Figure 5:**
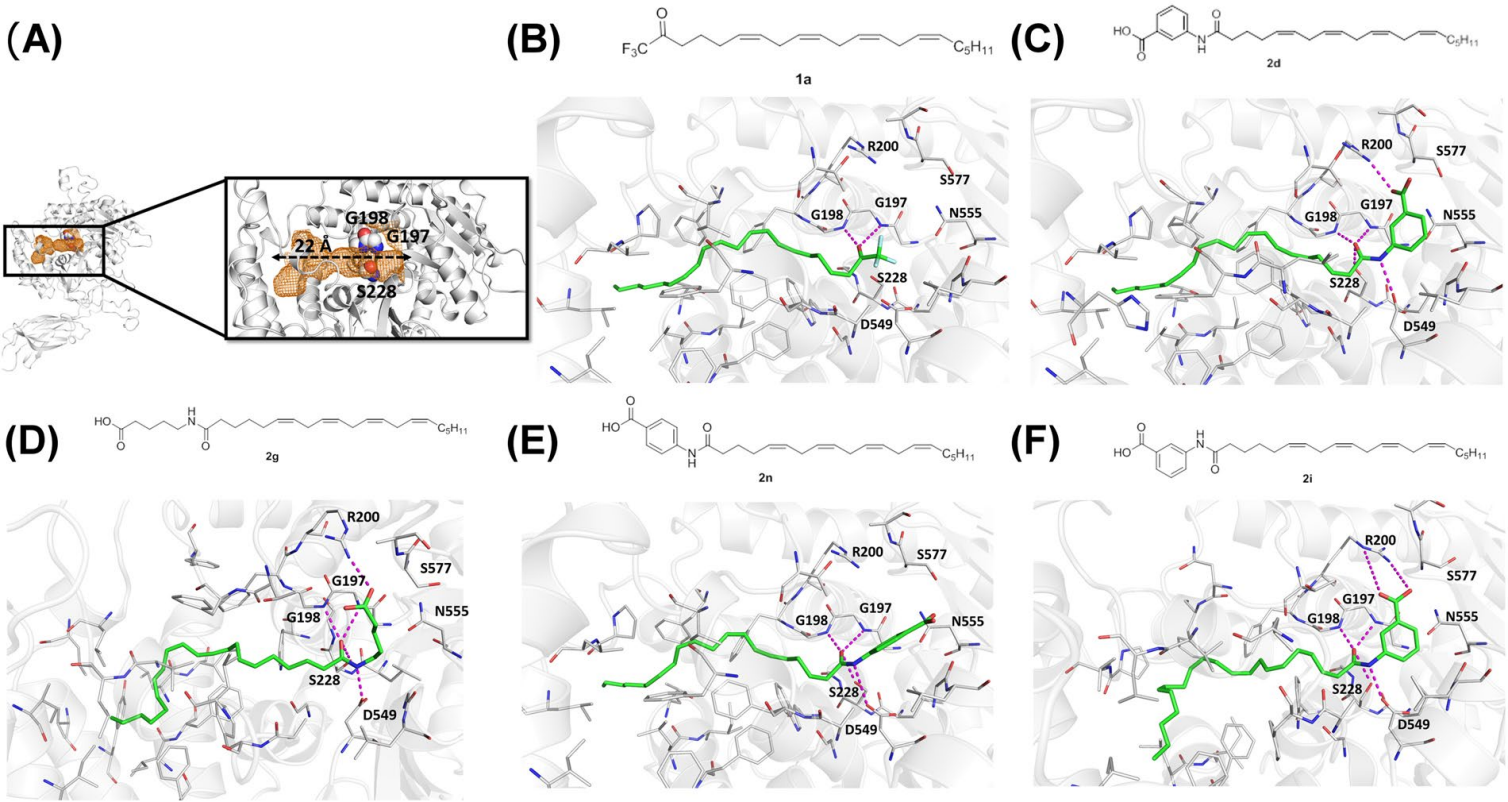
(**A**) Crystal structures of cPLA_2_ derived from 1CJY. Cartoon representations of the overall structure of the cPLA_2_ (left). Inhibitor binding site on the cPLA_2_ (right). Residues Ser228 (S228), Gly197 (G197), Gly198 (G198) are highlighted. Binding site is shown as mesh (orange). Predicted binding mode of **1a** (**B**), **2d** (**C**), **2g** (**D**), **2n** (**E**), **2i** (**F**) docked into cPLA_2_ with key interacting residues highlighted. Residues in the active sites are shown as lines; hydrogen bonds are indicated by dashed lines (magenta); protein residues involved in hydrogen bond interactions are labelled accordingly.

Docking calculations identified a cPLA_2_ binding site around the catalytically important residue Ser228^43,44^. This binding site was shown to be highly negatively charged on one end and slightly positively charged on the other end. Both these charged ends are connected by a 22 Å long, narrow tunnel that is made up of hydrophobic amino acids (Fig. 5A). The cPLA_2_ binding site was calculated to have a total volume of ~205 Å^3^. Since there are no co-crystal structures of cPLA_2_-inhibitor available, various analogues of **1** and **2** were docked and the results obtained were compared to the experimental data to understand the binding of the compounds.

Docking calculations with **1a** showed the compound penetrating into the deep hydrophobic pocket of cPLA_2_ and the carbonyl group interacting with Ser228 via hydrogen bonds and van der Waals contacts (Fig. 5B). Using this docking protocol, analogues of **1** and **2** were docked around this region with different conformations of cPLA_2_ identified through clustering the MD simulation trajectories. Ten poses for each compound were computed and the best docking pose for each compound was chosen by ranking the computed binding energy (docking score). Analysis of the top scored solutions (lowest energy solutions) showed that all the compounds bind similarly in the active sites of cPLA_2_ with (i) the electrophilic carbon of the substituted ketone in **1** or amide in **2** being in close proximity to Ser228, and (ii) Gly197/Gly198 interacting with the carbonyl oxygen of **1** or the amide oxygen of **2**. In addition, docking analysis of **2** shows the carboxylic acid moiety interacting with the side chains of Arg200. For the aminobenzoic acid analogues **2d** and **2n**, the carboxylic acid functionality is in closer proximity to Arg200 when it is in the meta position than the para position (Fig. 5C,E). This indicates a stronger attractive interaction which could contribute to the stronger inhibitory property of **2d**. Furthermore, the interactions between the carboxylic acid moiety and Arg200 provide a tighter binding for **2g** and **2i** than **1a** (Fig. 5D,F). **2i** which has its carboxylic acid moiety closest to Arg200, has the most favorable interaction with cPLA_2_. These results corroborated with our experimental cPLA_2_ inhibition assay data which show **2i** as the strongest cPLA_2_ inhibitor.

## Conclusion

cPLA_2_ activation has been shown to be critically important in the regulation of homeostatic processes and disease pathogenesis. Therefore, cPLA_2_ represents a potential novel therapeutic target to treat a wide range of diseases from cancer to neurodegenerative diseases such as Alzheimer’s disease and multiple sclerosis^8,58^. In our efforts to develop potential drug candidates to treat neurological disorders, we have synthesized a new class of AA analogues and identified one compound, **2i** that is a non-cytotoxic, cPLA_2_-selective inhibitor which inhibits the enzyme more potently than AACOCF_3_. Computational modelling and extensive simulations support the ability of **2i** to dock into the active site of cPLA_2_. **2i** is predicted to be brain penetrant through our PAMPA assay and is able to reduce ROS and NO production in LPS-stimulated BV-2 microglial cells. Further studies are presently ongoing to develop the compound as a lead compound. Understanding its *in vivo* effect will be crucial in evaluating its clinical potential.

## Methods

Synthesis of 1 and 2. Detailed experimental procedures and compound characterization data can be found in the supplementary information, available in the online version of the paper.

### Enzchek phospholipase A_2_ fluorogenic assay (% activity)

The assay was conducted according to the manufacturer’s instruction (E10217, Molecular Probes) with slight modification. Briefly, substrate solution was separately prepared by mixing 5 μL of DOPG, 5 μL of DOPC and 5 μL of substrate respectively in 1.5 mL of 1X phospholipase A_2_ reaction buffer. The mixture was vortexed vigorously for 3 min before allowing to stand at room temperature for 1 h. 48 μL of 1X phospholipase A_2_ reaction buffer were added into each well of a 96-wells plate. This was followed by addition of 1 μL of recombinant cPLA_2_ (P4074-03R, US Biological) and 1 μL of 1 mM of the respective compound dissolved in DMSO. The final concentration of the test compound achieved is 10 μM. The reaction was initiated by adding 50 μL of the substrate solution and incubating at room temperature for 1 h. Fluorescence was measured using a Varioskan plate reader (Thermo Scientific, MA, USA) by exciting at 485 nm, while fluorescence emission was detected at 515 nm. Positive control was performed when 1 μL of test compound was replaced by vehicle DMSO. % activity was expressed as the mean value of duplicate wells from three experiments. % activity was calculated by the following formula

1 $$ \% activity=\frac{fluorescence\,of\,tested\,well-background}{fluorescence\,of\,postive\,control-background}$$

### Enzchek phospholipase A_2_ fluorogenic assay (IC_50_)

The assay was conducted as described above. Instead of adding a fixed concentration of the test compound, various concentrations of compound were reconstituted in DMSO. 48 μL of 1X phospholipase A_2_ reaction buffer were added into each well of a 96-wells plate. This was followed by addition of 1 μL of recombinant cPLA_2_ (P4074-03R, US Biological) and 1 μL of the respective concentration of the test compound dissolved in DMSO. The reaction was initiated by adding 50 μL of the substrate solution and incubating at room temperature for 1 h. Fluorescence was measured using a Varioskan plate reader by exciting at 485 nm, while fluorescence emission was detected at 515 nm. Positive control was performed when 1 μL of test compound was replaced by vehicle DMSO. IC_50_ was expressed as the mean value of duplicate wells from three experiments.

### Enzchek phospholipase A_2_ fluorogenic assay (selectivity)

The assay was conducted as described above. Instead of adding cPLA_2_, it was replaced by sPLA_2_. 48 μL of 1X phospholipase A_2_ reaction buffer was added into each well of a 96-wells plate. This was followed by addition of 1 μL of sPLA_2_, human recombinant type V (10009563, Cayman Chemical) and 1 μL of 1 mM of the respective compound or thioetheramide-PC (62750, Cayman Chemical) dissolved in DMSO. The final concentration of the test compound achieved is 10 μM. The reaction was initiated by adding 50 μL of the substrate solution and incubating at room temperature for 1 h. Fluorescence was measured using a Varioskan plate reader by exciting at 485 nm, while fluorescence emission was detected at 515 nm. Positive control was performed when 1 μL of test compound was replaced by vehicle DMSO. % activity was expressed as the mean value of duplicate wells from four experiments. % activity was calculated by the following formula:

2 $$ \% activity=\frac{fluorescence\,of\,tested\,well-background}{fluorescence\,of\,postive\,control-background}$$

### Cell-culture and LPS treatment

Murine BV-2 cells were cultured in DMEM (11965092, ThermoFisher Scientific) supplemented with 10% fetal bovine serum, 100 μg/mL streptomycin and 100 units/mL penicillin. Cells were maintained in an incubator at 37 °C, under a 5% CO_2_ and in a water saturated environment. When performing LPS treatment, BV-2 cells were plated on a 10 cm dish in 10 mL culture medium at a density of 1 × 10^6^ cells per dish one day prior to the experiment. After observing that cells were at 90% confluency, lipopolysaccharide (L6529, Sigma) pre-dissolved in culture medium to achieve a concentration of 2 μg/mL was added to the dish. Different concentrations of test compounds in DMSO were added to the LPS containing medium which was then transferred to the 10 cm dish. DMSO was used as a vehicle control and added to the non-LPS treated control. The cells were incubated at 37 °C, under a 5% CO_2_ environment. After the respective timepoints, cells were washed once with 1xPBS (10 mL) and scrapped on ice with 8 mL 1xPBS. Collected cells were centrifuged at 800 rpm for 3 min at 4 °C, before discarding the supernatant. Cells were lysed by RIPA buffer supplemented with 1× protease inhibitor (Roche) and 1 mM PMSF and incubated under ice for 30 min. This was followed by pelleting the cell debris at 4 °C for 30 min at 13200 rpm before transferring the supernatant to a clean tube and storing the sample at −20 °C. Three independent biological repeats were performed.

### MTS cell proliferation assay

Cells were plated on a 96-wells plate and grown for 24 h to a density of 8000 cells per well for both HEK293T and BV-2 cells. Cells were cultured at 37 °C under a 5% CO_2_ environment in 100 μL of culture medium. Thereafter, the compound of interest dissolved in culture medium at a concentration of 10 μM was replaced into each well. DMSO was added as vehicle in the control group. 3 biological replicates were performed. The plates were then incubated at 37 °C with 5% CO_2_ for 48 h and 72 h. At each individual time point, 20 μL of CellTiter 96® AQueous One Solution (MTS reagent) was added into each well and re-incubated at 37 °C with 5% CO_2_ without light for 3 h. Colorimetric readings were taken using a Varioskan plate reader at 490 nm and analyzed. Three independent biological repeats with four technical replicates were performed.

### BCA Assay

BCA assay (23225, Pierce BCA Protein Assay Kit) was performed to quantify the concentration of proteins in the cell lysate. 10 µL of test samples (1 µL with 9 µL of lysis buffer) and BSA standards (range from 1.25 to 50 μg/ml) were mixed in a 96 well-plate and incubated with 190 µL of working reagent mixture (Ratio of Reagent A:B used was 50:1) at 37 °C for 20 min. The colour intensity that was developed was read at 562 nm using a spectrophotometer. A standard curve of different concentrations of BSA was plotted using the average blank-corrected readings for each standard *vs*. its respective concentration (µg/ml). This was then used to determine the protein concentration for unknown samples.

### Western Blot

25 μg of individual protein samples were mixed with an equal volume of 2x Laemmli buffer containing DTT and denatured at 90 °C for 10 min. The lysates were ran in a 10% tris-glycine SDS-PAGE gel at 100 V for 1.5 h in 1x tris-glycine running buffer. The protein samples on the gel were then transferred onto a PVDF membrane. The membranes were then blocked for 1 h at room temperature in 5% skim milk in 1x TBS-0.1% Tween 20 (TBST), followed by overnight incubation with the respective primary antibody dissolved in 5% milk in TBST at 4 °C. Secondary detection were conducted with a secondary horse-radish peroxidase-conjugated antibody (1:10000) dissolved in 5% skim milk in TBST for 1 h at room temperature. Enhanced chemiluminescence (ECL) detection of protein bands was performed using ECL Plus Western Blotting detecting system (GE Healthcare). Antibodies used in western blot include rabbit polyclonal anti-NOS2 (Santa-Cruz, sc-651, 1:100), mouse monoclonal anti-cPLA_2_ primary antibody (Santa Cruz, sc-454, 1:200), mouse monoclonal anti-GAPDH (Merck, 1:10000), with secondary horse-radish peroxidase-conjugated antibodies (Santa Cruz, 1:10000).

### ROS assay

20,000 BV-2 cells were seeded into a 96-well plate in 100 uL of culture medium and allowed to grow overnight. When a 90% cell confluency was observed, lipopolysaccharide pre-dissolved in culture medium to achieve a concentration of 2 μg/mL was added to each well of the 96 well. Different concentrations of test compounds in DMSO were added to the LPS containing medium and was then transferred to the cells. DMSO was used as vehicle control and added to the non-LPS treated control. The cells were incubated for 14 h at 37 °C, under a 5% CO_2_ environment. Medium were removed and washed once with 1x HBSS. 100 μL of 10 µM CM-H2DCFDA (C6827, Molecular Probes) in 1x HBSS solution was added into each well and incubated at 37 °C for 1 h. Fluorescence detection was done by exciting the dye at 485 nm and detection of emission at 525 nm. Five independent biological repeats with six technical replicates were conducted.

### Solubility determinations

Solubility determinations were carried out on Multiscreen® Solubility filter plates (Millipore-MSSLBPC10) from Millipore Corporation (MA, USA) following the protocol (PC2445EN00) from the manufacturer.

### Determination of permeability

The Parallel Artificial Membrane Permeability Assay (PAMPA) was used to determine the effective permeability (P_e_) of **2i**. The method reported by Di *et al*.^53^ was followed with modifications. Determinations were carried out on MultiScreen-IP PAMPA assay (donor) plates (MAIPNTR10) and MultiScreen Receiver Plates (MATRNPS50) from Millipore Corporation (USA) with porcine polar brain lipids (Avanti Polar Lipids Inc, Alabaster, AL) as the lipid barrier.

An aliquot (5 µL) of 2% porcine brain lipids in dodecane (ReagentPlus^®^, Sigma Aldrich, USA) was dispensed into the donor well. A stock solution (5 mM) of **2i** prepared in DMSO was diluted with 1x phosphate buffer (PBS, pH 7.4) to give a 50 µM solution. An aliquot (300 µL) of the solution was dispensed into the donor well and an equal volume of buffer (1xPBS with 1% DMSO) was added to the corresponding acceptor well. The donor and acceptor plates were assembled, the unit was placed in a humidified box and gently agitated on a mini shaker at room temperature (25 °C) for 16 h. After this time, aliquots (200 µL) were withdrawn from the donor and acceptor wells, diluted separately to 1 mL with PBS, and quantified at λmax of 290 nm on a Shimadzu UV-1800 Spectrophotometer.

The PAMPA permeabilities of standards (caffeine, quinidine, carbamazepine, dopamine and propanolol) were determined under similar conditions. 300 µL aliquots of stock solutions (500 µM, 1xPBS, 0.1% DMSO) were dispensed to donor wells as described earlier. Quantification was by UV at λmax of 272 nm (caffeine), 284 nm (carbamazepine), 330 nm (quinidine), 280 nm (dopamine) and 288 nm (propanolol). Calibration plots of standards were determined under similar analytical conditions. The effective permeability (Pe) of these compounds have been reported to vary in the sequence propranolol (most permeable) > carbamazepine > quinidine > caffeine > dopamine (least permeable)^54,55^ and was verified experimentally in our hand. P_e_ was obtained from (3):

3 $${\rm{Pe}}=-2.303\times \{{{\rm{V}}}_{{\rm{A}}}{{\rm{V}}}_{{\rm{D}}}/[({{\rm{V}}}_{{\rm{A}}}+{{\rm{V}}}_{{\rm{D}}})\times {\rm{A}}\times {\rm{t}}]\}\times \,\mathrm{log}\{1-[({{\rm{V}}}_{{\rm{A}}}+{{\rm{V}}}_{{\rm{D}}})/({{\rm{V}}}_{{\rm{D}}}.{\rm{S}})]\times {{\rm{C}}}_{{\rm{A}}(t)}/{{\rm{C}}}_{{\rm{D}}(0)}\}$$

where V_A_ and V_D_ are the volumes of acceptor (cm^3^) and donor (cm^3^) wells respectively, A is the area of the surface area of the membrane (0.24 cm^2^), t is the permeation time (s). The P_e_ of each compound was obtained from at least 3 separate experiments using 2 different stock solutions. For each independent determination, triplicates (3 wells) were run for each compound.

### *In silico* docking analysis

Details on protein modeling, ligand preparation and ligand docking can be found in the supplementary information, available in the online version of the paper.

### Data Availability

All data generated or analysed during this study are included in this published article (and its Supplementary Information files).

## Electronic supplementary material

Supplementary Information
