# Supplementary material for: A New Generation of Arachidonic Acid Analogues as Potential Neurological Agent Targeting Cytosolic Phospholipase A2
Source: Sci Rep. 2017 Oct 20;7:13683. doi: 10.1038/s41598-017-13996-8 (PMC5651845; doi:10.1038/s41598-017-13996-8)
Supplement: Supplementary file 1 — Supplementary Information [file 41598_2017_13996_MOESM1_ESM.pdf]

Supporting Information

A New Generation of Arachidonic Acid Analogs as Potential Neurological Agent Targeting Cytosolic Phospholipase A_2

Cheng Yang Ng[a], Srinivasaraghavan Kannan[b], Yong Jun Chen[c], Francis Chee Kuan Tan[d], Wee Yong Ong[a], Mei Lin Go[c], Chandra S Verma[b], Chian-Ming Low*[e] & Yulin Lam*[a]*

[a] Department of Chemistry, National University of Singapore, 3 Science Drive 3, Singapore 117543

[b] Bioinformatics Institute, Agency for Science, Technology and Research (A*STAR), Matrix, Singapore 138671

[c] Department of Pharmacy, National University of Singapore, 21 Lower Kent Ridge Road, Singapore 119077

[d] Department of Anesthesia, Yong Loo Lin School of Medicine, National University of Singapore, 5 Lower Kent Ridge Road, Singapore 119074

[e] Department of Pharmacology, Yong Loo Lin School of Medicine, National University of Singapore, 16 Medical Drive, Singapore 117600

* For Y.L.: phone: (65)-6516-2688; fax: (65)-6779-1691; e-mail: chmlamyl@nus.edu.sg

* For C.-M.L.: phone: (65)-6516-5841; fax: (65)-6873-7690; e-mail: phclowcm@nus.edu.sg

* For C.V.: phone: (65)-6478-8273; fax: (65)-6478-9047; email: chandra@bii.a-star.edu.sg

Table of Content

Synthesis of **1** and **2**	3 - 7
**Table S1.** Optimization for the hydrogenation of **8a**	8
**Figure S1**: IC_{50} of **1a**, **2g** and **2i** on $cPLA_2$	8
Synthesis of (5Z,8Z,11Z,14Z)-icosa-5,8,11,14-tetraenamide **1m**	21
Synthesis of (5Z,8Z,11Z,14Z)-N-(3-sulfamoylphenyl)icosa-5,8,11,14-tetraenamide **2p**	39
^{1}H and ^{13}C NMR Spectra of **1a-1o**, **2a-2q**, **4a-4e**, **5a-5e**, **6a-6e**, **7a-7e**, **9a-9e**	
**11a-11c**, **13a-13d**, **14a-14d**, **16a-16d**, **18a-18c**, **20a-20k**, **20n-20o, 22** and **24**	9 – 100
In-silico docking analysis (protein modeling, ligand preparation and ligand docking)	100 – 101
**Figure S2**: Images of blots presented in Figure 3	101
**Figure S3**: Images of blots presented in Figure 4	102
References	102

Synthesis of 1 and 2

Commercially available reagents were bought from Sigma Aldrich, Alfa Aesar, Acros and Tokyo Chemical Industry and used without purification. $AACOCF_3$, CDIBA and pyrrophenone were bought from Sigma Aldrich, Axon Medchem and Santa Cruz respectively. Solvents such as hexane, ethyl acetate, dichloromethane, and methanol were pre-distilled while others were used without further purification. Flash column chromatography was carried out on Merck silica gel 60. Thin-layer chromatography (TLC) was performed on precoated F254 silica plates from Merck and visualized with UV light. TLC were heated with potassium permanganate stain whenever necessary. Preparative TLC was from Analtech Silica GEL GP (Cat 02015). ^{1}H and ^{13}C NMR spectra were recorded on Bruker ACF300 (300MHz) or AMX 500 (500MHz) spectrometer at 298K. All J values are reported in Hz and chemical shift (δ) reported in parts per million (ppm) relative to tetramethylsilane (TMS). Mass spectra were determined by high resolution mass spectrometry (HRMS) electrospray ionization (ESI) or atmospheric-pressure chemical ionization (APCI).

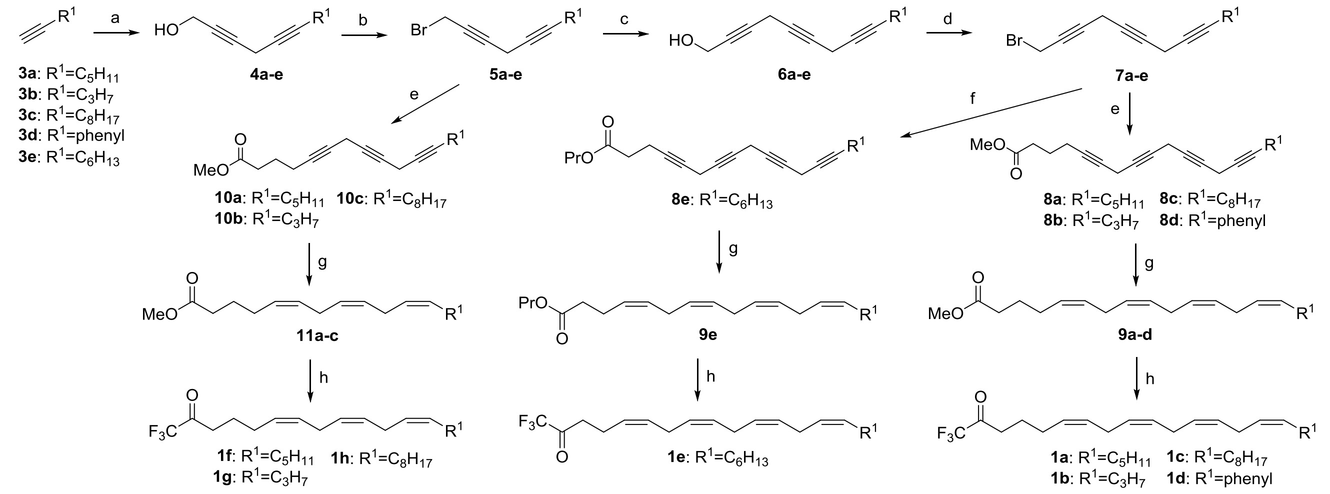

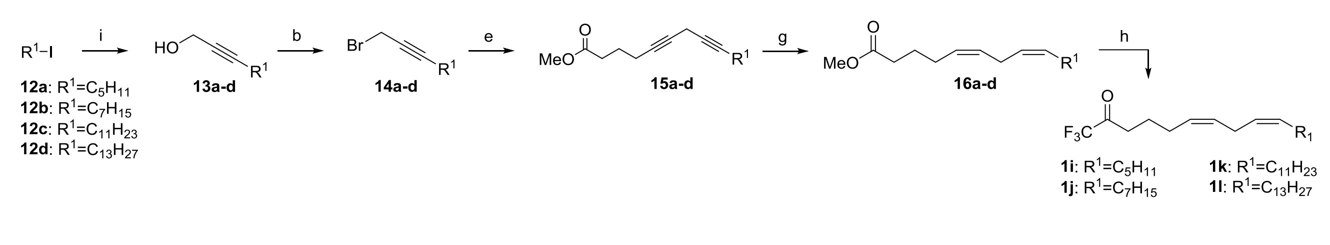

Scheme S1. Reagent and conditions: (a) 4-Chloro-2-butyn-1-ol, CuI, NaI, K_2CO_3, DMF, rt, overnight (b) CBr_4, PPh_3, CH_2Cl_2, -40°C to -20°C, 1 h (c) Propargyl alcohol, CuI, NaI, K_2CO_3, DMF, rt, overnight (d) CBr_4, PPh_3, CH_2Cl_2, -40°C to -20°C, 1 h (e) Methyl 6-hexynoate,

CuI, NaI, K_2CO_3, DMF, rt, overnight (f) Propyl 5-pentynoate, CuI, NaI, K_2CO_3, DMF, rt, overnight (g) H_2, $Ni(OAc)_2.4H_2O$, $NaBH_4$, en, 95% EtOH, rt, 2 h (h) i) NaOH, MW 120^oC, 1 h ii) Trifluoroacetic anhydride, Pyridine, CH_2Cl_2, rt, 2 h (i) Propargyl alcohol, n-BuLi, HMPA, THF, -78^oC to rt, overnight

General procedure for the synthesis of 4a-e, 6a-e, 8a-e, 10a-c, 15a-d, 17a-d and 23. A mixture of the terminal alkyne (17.41 mmol), allylic halide (11.61 mmol), CuI (3.32 g, 17.41 mmol), NaI (2.61 g, 17.41 mmol) and K_2CO_3 (2.41 g, 17.41 mmol) were dissolved in DMF (36 mL) and stirred overnight at room temperature. The reaction mixture was then quenched by adding an equal volume of ethyl acetate and the organic layer was washed with saturated NH_4Cl (3 x 20 mL) and brine (3 x 20 mL). The organic layer was then dried using anhydrous $MgSO_4$, concentrated and purified using flash column chromatography to afford the product as either a yellow oil or a yellow solid. The following cross-coupled products, **8a-e**, **10a-c**, **15a-d**, **17a-d** and **23**, were not isolated and were used directly for the next step of the reaction.

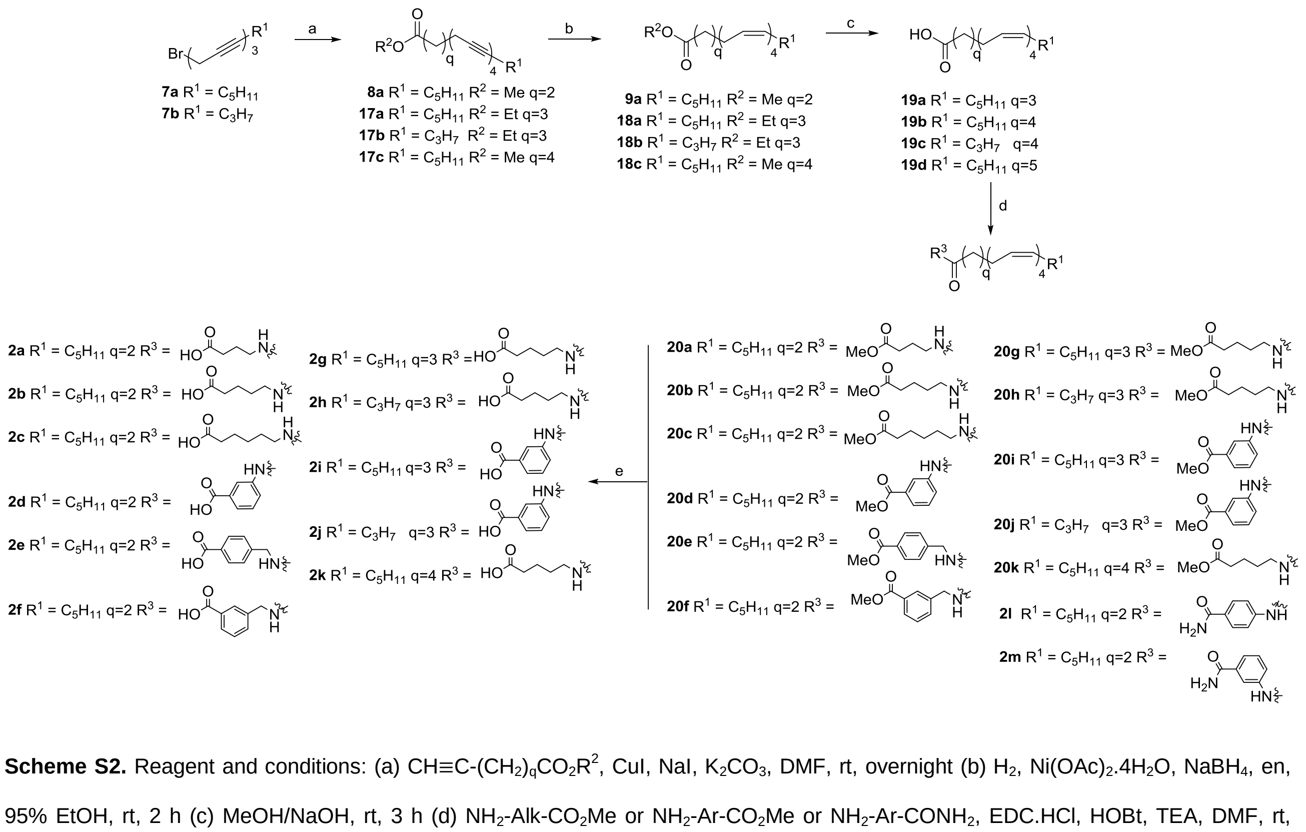

Scheme S2. Reagent and conditions: (a) CH≡C-$(CH_2)_qCO_2R^2$, CuI, NaI, K_2CO_3, DMF, rt, overnight (b) H_2, $Ni(OAc)_2.4H_2O$, $NaBH_4$, en, 95% EtOH, rt, 2 h (c) MeOH/NaOH, rt, 3 h (d) NH_2-Alk-CO_2Me or NH_2-Ar-CO_2Me or NH_2-Ar-$CONH_2$, EDC.HCl, HOBt, TEA, DMF, rt, overnight e) MeOH/NaOH, rt, 3 h.

General procedure for the synthesis of 5a-e, 7a-e and 14a-d. The respective alcohol (6.124 mmol) and CBr_4 (4.062 g, 12.25 mmol) were dissolved in CH_2Cl_2 (5.2 mL) and cooled to -40^oC. PPh_3 (3.213 g, 12.25 mmol) was dissolved in CH_2Cl_2 (5.2 mL) and added dropwise via a canella to the reaction mixture, which was then stirred for 1 h at -20^oC. Upon completion of the reaction, hexane was added to the reaction mixture,

resulting in the precipitation of PPh_3O. The resulting suspension was filtered through Celite, concentrated and purified by flash column chromatography using a 99:1 hexane-ethyl acetate eluent system to afford the respective product as either a yellow or colourless oil.

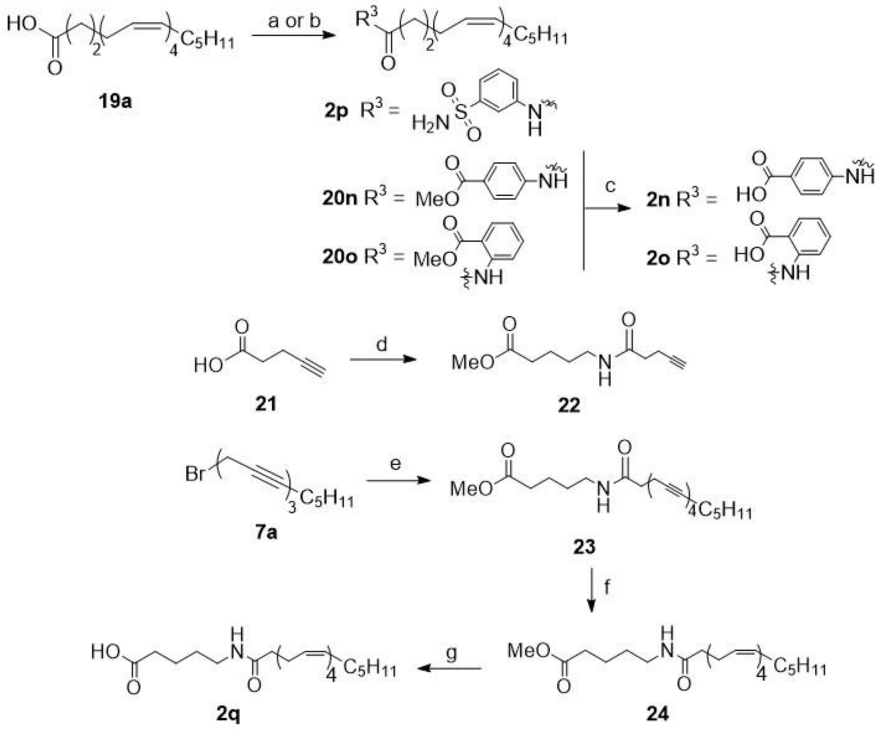

Scheme S3. Reagent and conditions: (a) 3-aminobenzenesulfonamide, EDC.HCl, TEA, MeCN, 4°C, overnight (b)i) ($COCl_2$), catalytic DMF, CH_2Cl_2, rt, 1 h ii) NH_2-Ar-CO_2Me, TEA, THF, rt, overnight (c) MeOH/NaOH, rt, 3 h (d) Methyl 5-aminopentanoate hydrochloride, EDC.HCl, HOBt, TEA, DMF, rt, overnight (e) **22**, CuI, NaI, K_2CO_3, DMF, rt, overnight (f) H_2, $Ni(OAc)_2.4H_2O$, $NaBH_4$, en, 95% EtOH, rt, 2 h (g) MeOH/NaOH, rt, 3 h.

General procedure for the synthesis of 9a-e, 11a-c, 16a-d, 18b-d and 24. The hydrogenation procedure employed was adapted from the procedure described by Qi *et al.*[1] $Ni(OAc)_2.4H_2O$ (0.119 g, 0.4773 mmol) was dissolved in a 95% EtOH : 5% 2M NaOH solution and saturated with hydrogen gas through bubbling. 0.4773 mL of 1M $NaBH_4$ (0.4773 mmol) solution was then added to the $Ni(OAc)_2.4H_2O$ mixture under a H_2 atmosphere and stirred vigorously to yield a black suspension. Ethylenediamine (0.032 mL, 0.4773 mmol) was then added to the mixture and H_2 gas was bubbled through it for another 15 – 20 min. The alkyne (0.3182 mmol) was then dissolved in CH_2Cl_2 (2 mL) and added to the reaction mixture. The reaction mixture was stirred under positive hydrogen pressure for 2 h, quenched by passing the mixture through Celite, concentrated and

purified via flash column chromatography with a 98:2 hexane-ethyl acetate eluent system to afford the respective product as a yellow oil.

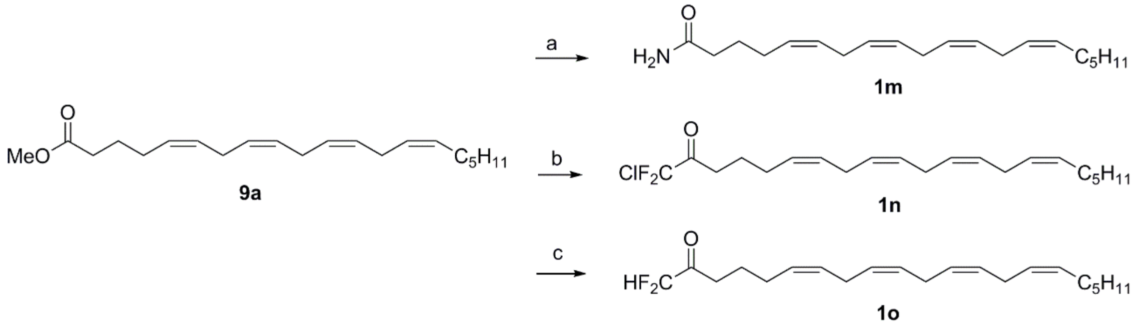

Scheme S4. Reagent and conditions: (a) NH_3, $Mg(OMe)_2$, MeOH, 80°C, overnight (b) i) NaOH, MW 120°C, 1 h ii) Chlorodifluoroacetic anhydride, Pyridine , CH_2Cl_2, rt, overnight (c) i) NaOH, MW 120°C, 1 h ii) Difluoroacetic anhydride, Pyridine , CH_2Cl_2, rt, overnight.

General procedure for the synthesis of 1a-l and 1n-o. To the respective ester (0.31 mmol) was added 4M NaOH (5 mL) and the reaction mixture was microwaved for 1 h at 120°C. Upon cooling to ambient temperature, the reaction mixture was quenched with 3M HCl (7 mL) and extracted with CH_2Cl_2 (3 x 20 mL). The combined organic phase was dried with anhydrous $MgSO_4$, concentrated and used for the next step of the reaction without further purification. Trifluoromethylation was performed by adapting the procedure reported by Ackermann *et al*.[2] The acid obtained in the aforementioned reaction (0.31 mmol) was dissolved in CH_2Cl_2 (5 mL). Thereafter, pyridine (0.26 mL, 3.20 mmol) and trifluoroacetic/chlorodifluoroacetic anhydride (0.44 mL, 3.123 mmol) were added. For **1a-h**, the reaction mixture was stirred for 2 h. For **1n** and **1o**, the mixture was stirred overnight. Upon completion of reaction (based on TLC), the reaction was quenched by shaking the mixture with saturated $NaHCO_3$ (50 mL) and subsequently extracting the aqueous layer with ethyl acetate (3 x 20 mL). The combined organic layer was then dried with anhydrous $MgSO_4$, concentrated and purified using flash column chromatography with a 98:2 hexane-ethyl acetate eluent system to afford the respective product as a yellow oil.

General procedure for the alkylation of 12a-d to form 13a-d. The general procedure employed was adapted from the protocol reported by Yoshida *et al*.[3] To propargyl alcohol (116 µL, 2.00 mmol) in THF (3 mL) was added HMPA (1.1 mL, 6.3 mmol) and the reaction mixture was cooled to -78°C. 2 M n-Butyllithium in cyclohexane (2.0 mL, 4 mmol) was then added to the mixture via a canella and stirred vigorously. Thereafter, the respective **12** (1.00 mmol) dissolved in THF (3 mL) was transferred to the reaction mixture via

a canella. The temperature of the reaction mixture was allowed to rise to room temperature while stirring overnight. Upon completion of the reaction (based on TLC), the reaction was quenched by adding an equivalent amount of ethyl acetate and washed with saturated NH_4Cl (3 x 20 mL). The organic layer was dried with $MgSO_4$, concentrated and purified by flash column chromatography using a 5:1 hexane-ethyl acetate eluent system to afford the respective product as a colourless oil or white solid.

General procedure amidation reaction to afford 2l-m, 20a-k and 22. EDC.HCl (0.029 g, 0.15 mmol), HOBt (0.017 g, 0.120 mmol), the respective **19** (30.6 mg, 0.100 mmol), TEA (15 μL, 0.105 mmol) and the respective amine (0.105 mmol) were dissolved in DMF (1.5 mL) and stirred for 3 h at room temperature. Upon the completion of reaction, the reaction was quenched by adding an equivalent amount of ethyl acetate and washed with aqueous $NaHCO_3$ (3 x 20 mL) and brine (3 x 20 mL). The organic layer was dried with $MgSO_4$ and purified by flash column chromatography.

General procedure for the synthesis of 20n and **20o.** The respective acid **19** (0.12mmol) was dissolved in CH_2Cl_2 (3.5 mL). Thereafter, oxalyl chloride (21.2 μL, 0.243 mmol) and 1 drop of DMF were added and the mixture was stirred for 1 h at room temperature. After which, the solvent was removed. The formed acid chloride was re-dissolved in DMF (1.0 mL), the respective amine (0.243 mmol) and TEA (34 μL, 0.243 mmol) and stirred at room temperature overnight. When no more starting material was observed, ethyl acetate and brine were added to quench the reaction. The mixture was extracted with ethyl acetate (3x 20mL) and the organic layer was combined, dried with $MgSO_4$ and purified by flash column chromatography.

General hydrolysis procedure to yield 2a-k, 2n-o and 2q. The respective ester **20** or **24** (0.03 mmol) was dissolved in 2M methanolic NaOH (2.0 mL) and stirred at room temperature for 3 h. When no more starting material was observed, the reaction was quenched with 1M HCl and then extracted with ethyl acetate (3x20 mL). The organic layer was separated and the combined organic layer was dried with $MgSO_4$, concentrated and purified by flash column chromatography.

Table S1. Optimization for the hydrogenation of **8a**

Entry	Equivalents of Brown's P2 nickel catalyst	Solvent	Time (h)	Yield (%)
1[9d]	0.2	Pure EtOH	8	-
2	2.24	95.5 EtOH-2M NaOH	8	-
3	2.24	95.5 EtOH-2M NaOH	3	19
4	2.24	95.5 EtOH-2M NaOH	1	63
5	1.5	95.5 EtOH-2M NaOH	1	68

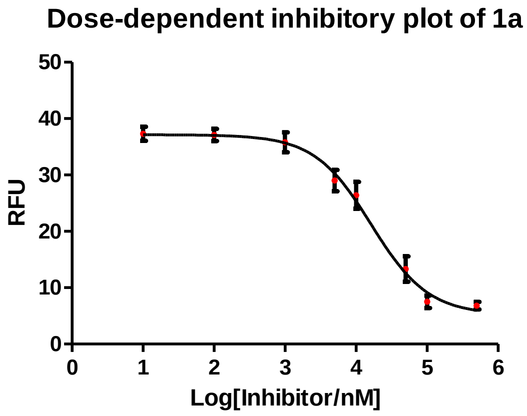

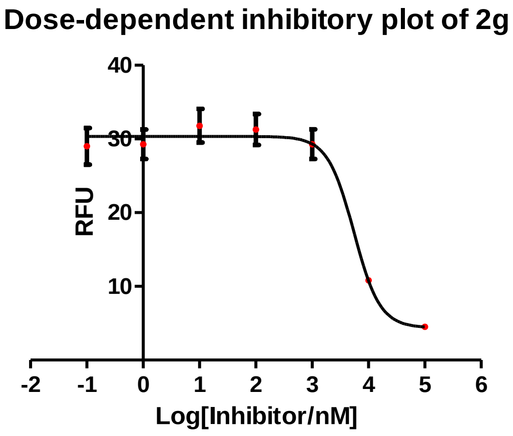

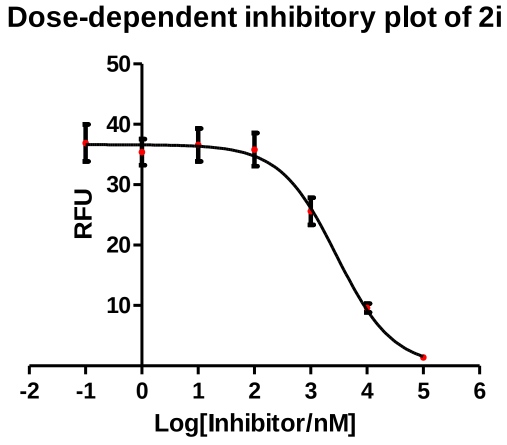

Figure S1: IC_{50} of **1a**, **2g** and **2i** on $cPLA_2$

(6Z,9Z,12Z,15Z)-1,1,1-trifluorohenicosa-6,9,12,15-tetraen-2-one (1a): Yellow oil, 48% yield (over 2 steps). ^{1}H NMR (500 MHz, $CDCl_3$) δ 5.57 – 5.26 (m, 8H), 2.97 – 2.79 (m, 6 H), 2.74 (t, *J* = 7.1 Hz, 2 H), 2.23 – 2.12 (m, 2 H), 2.08 (dd, *J* = 13.9, 6.8 Hz, 2 H), 1.80 (dd, *J* = 14.4, 7.2 Hz, 2 H), 1.50 – 1.22 (m, 6 H), 0.94 (t, *J* = 6.95 Hz, 3 H); ^{13}C NMR (125 MHz, $CDCl_3$) δ 191.42 (d, *J* = 35 Hz), 130.51, 129.65, 128.65, 128.41, 128.16, 127.85, 127.76, 127.50, 115.57 (q, *J* = 291 Hz), 35.60, 31.51, 29.31, 27.21, 26.01, 25.63, 25.59 (2 C), 22.55, 22.17, 14.02. HRMS (APCI): calculated for $C_{21}H_{32}F_3O$ (M+1) 357.2400, found 357.2384.

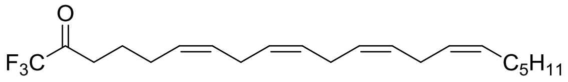

1a

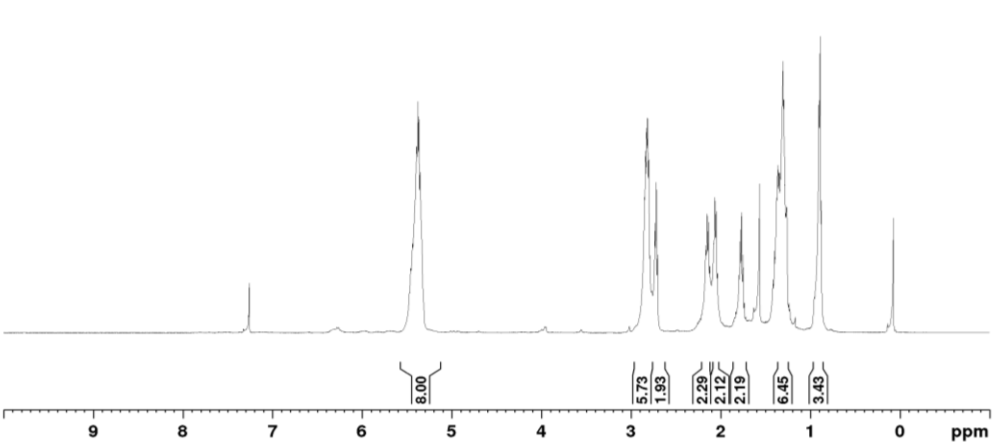

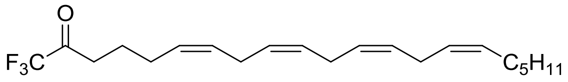

1a

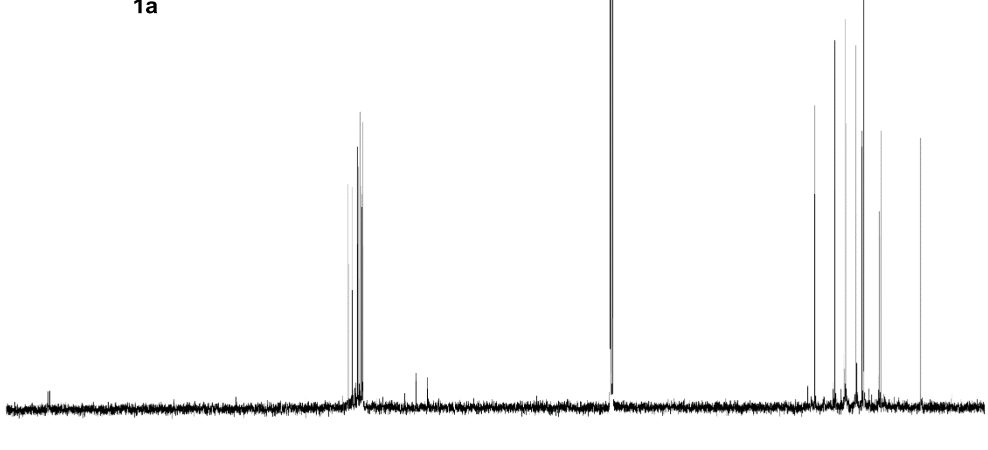

(6Z,9Z,12Z,15Z)-1,1,1-trifluorononadeca-6,9,12,15-tetraen-2-one (1b): Yellow oil, 46% yield (over 2 steps). ^{1}H NMR (500 MHz, $CDCl_3$) δ 5.44 – 5.34 (m, 8H), 2.82 (dt, *J* = 19.3, 6.7 Hz, 6H), 2.72 (t, *J* = 7.2 Hz, 2H), 2.14 (dd, *J* = 14.4, 7.2 Hz, 2H), 2.05 (dd, *J* = 14.1, 7.1 Hz, 2H), 1.76 (dd, *J* = 14.4, 7.2 Hz, 2H), 1.38 (dt, *J* = 14.6, 7.3 Hz, 2H), 0.91 (t, *J* = 7.3 Hz, 3H); ^{13}C NMR (125 MHz, $CDCl_3$) δ 191.40 (d, *J* = 35 Hz), 130.24, 129.67, 128.66, 128.43, 128.16, 127.86, 127.78, 127.75, 115.59 (q, *J* = 292 Hz), 35.62, 29.31, 26.04, 25.66, 25.65, 25.61, 22.76, 22.20, 13.74. HRMS (APCI): calculated for $C_{19}H_{28}F_3O$ (M+1): 329.2087; found 329.2086.

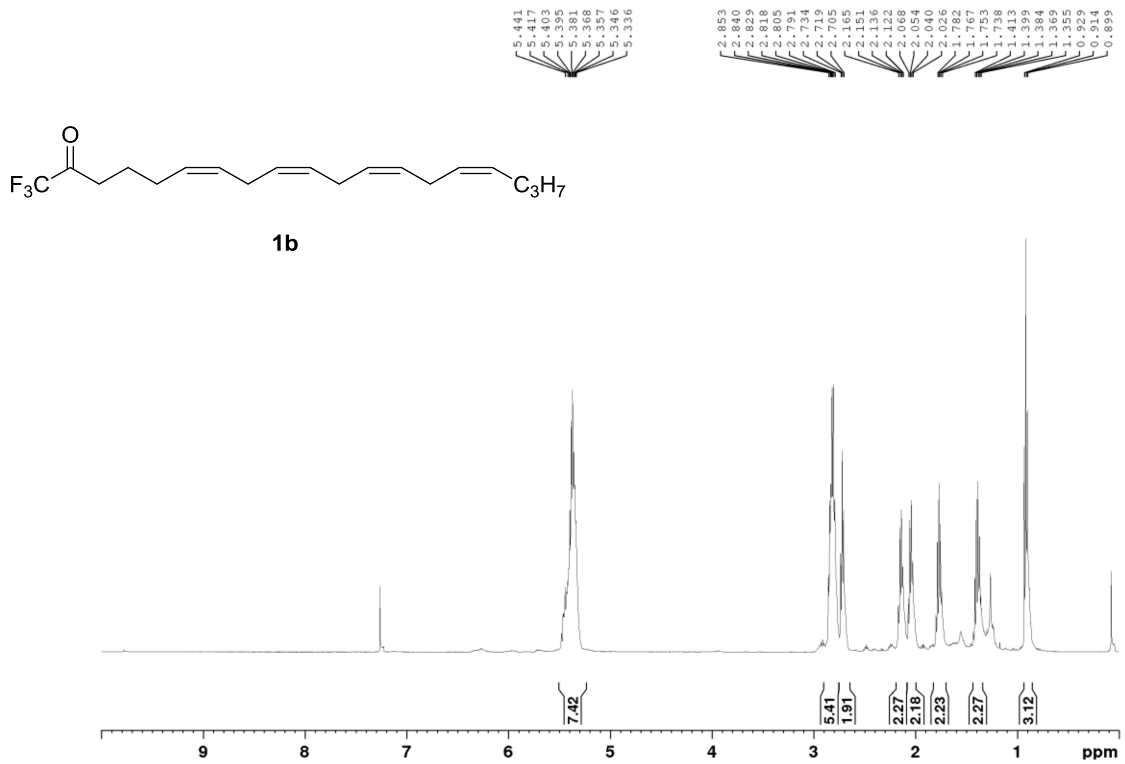

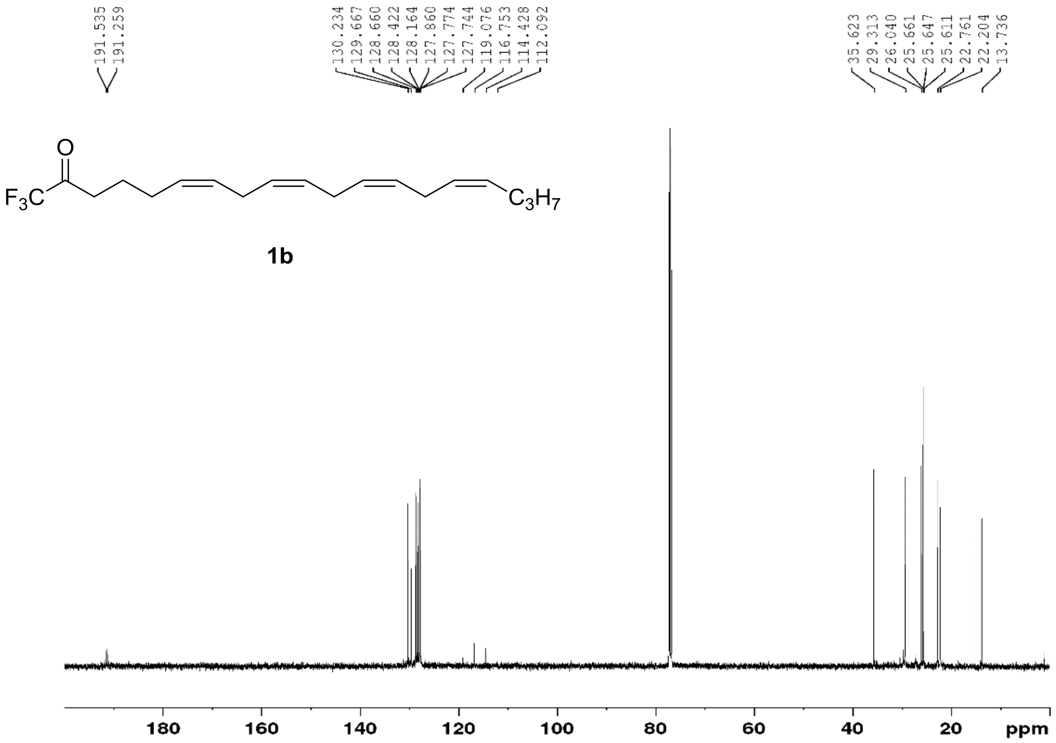

(6Z,9Z,12Z,15Z)-1,1,1-trifluorotetracosa-6,9,12,15-tetraen-2-one (1c): Yellow oil, 52% yield (over 2 steps). ^{1}H NMR (300 MHz, $CDCl_3$) δ 5.44 (tt, *J* = 8.5, 4.6 Hz, 8H), 2.96 – 2.67 (m, 6 H), 2.14 (ddd, *J* = 19.1, 13.4, 6.5 Hz, 6 H), 1.81 (dt, *J* = 13.6, 6.8 Hz, 2 H), 1.48 – 1.21 (m, 14 H), 0.93 (t, *J* = 8.9, 3 H); ^{13}C NMR (75 MHz, $CDCl_3$) δ 191.21 (d, *J* = 34 Hz), 130.37, 129.54, 128.53, 128.29, 128.04, 127.73, 127.64, 127.40, 115.49 (q, *J* = 291 Hz), 35.47, 31.81, 29.56 (2 C), 29.43, 29.23, 27.16, 25.91 (2 C), 25.53, 25.49, 22.57, 22.07, 13.93. HRMS (APCI): calculated for $C_{24}H_{38}F_3O$ (M+1) 399.2869, found 399.2855.

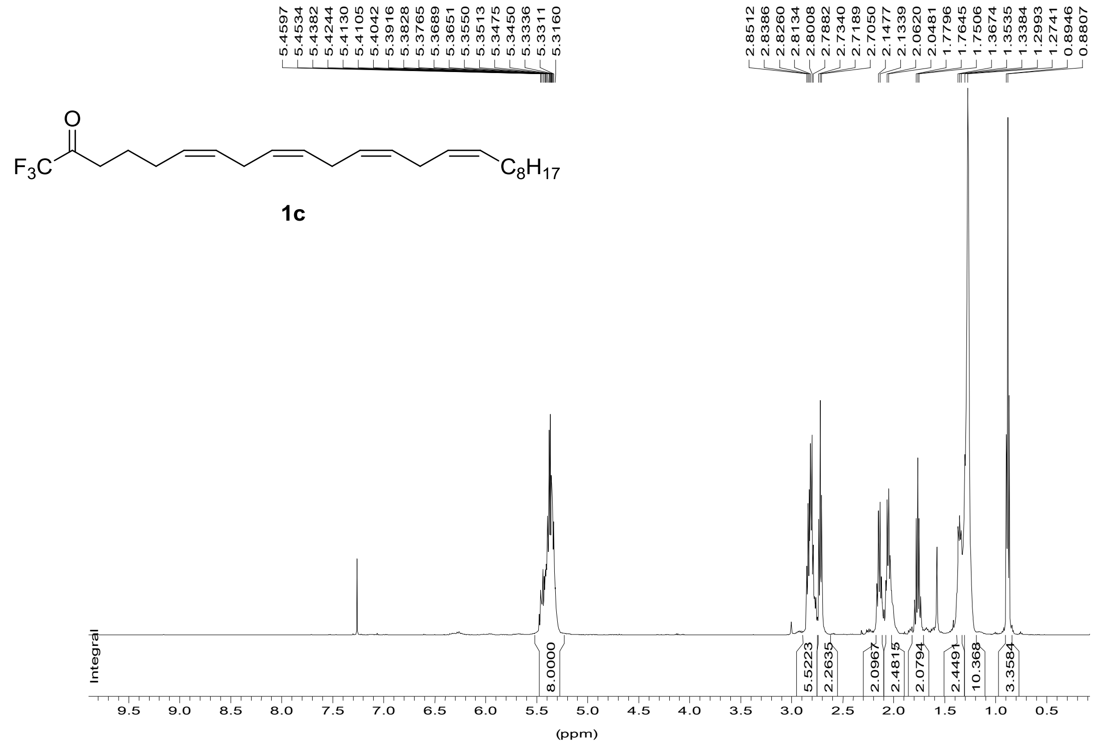

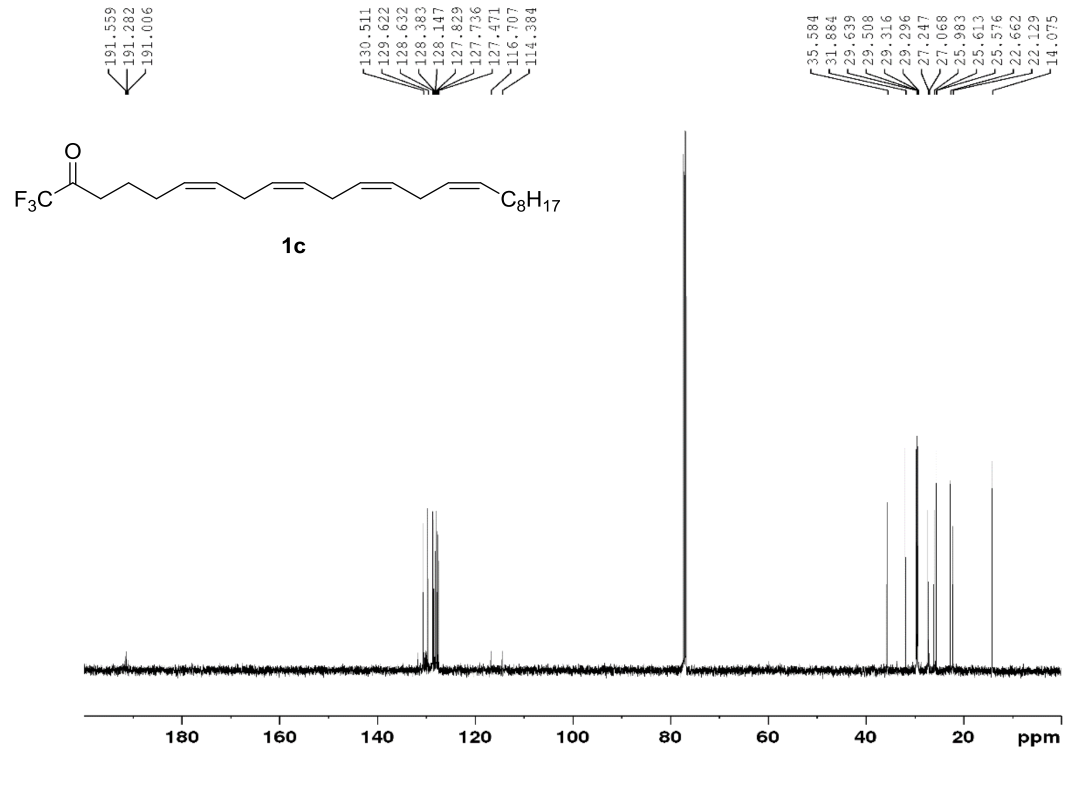

(6Z,9Z,12Z,15Z)-1,1,1-trifluoro-16-phenylhexadeca-6,9,12,15-tetraen-2-one (1d): Yellow oil, 60% yield (over 2 steps). ^{1}H NMR (500 MHz, $CDCl_3$) δ 7.30 (ddt, *J* = 14.5, 7.4, 4.4 Hz, 5H), 6.49 (d, *J* = 11.5 Hz, 1H), 5.68 (dt, *J* = 11.5, 7.5 Hz, 1H), 5.53 – 5.34 (m, 6H), 3.12 (t, *J* = 6.9 Hz, 2H), 2.83 – 2.71 (m, 6H), 2.14 (dd, *J* = 14.5, 7.3 Hz, 3H), 1.78 (dd, *J* = 14.5, 7.2 Hz, 2H); ^{13}C NMR (125 MHz, $CDCl_3$) δ 191.56 (d, *J* = 35 Hz), 137.35, 130.54, 129.57, 129.32, 128.71, 128.63, 128.15, 128.11, 127.96, 126.64, 115.55 (d, *J* = 292 Hz), 35.58, 26.95, 25.97, 25.71, 25.56, 22.13. HRMS (APCI): calculated for $C_{22}H_{26}F_3O$ (M+1): 363.1930; found 363.1917.

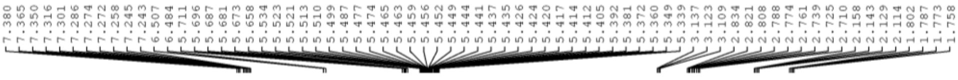

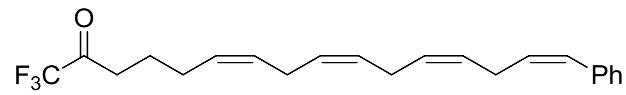

1d

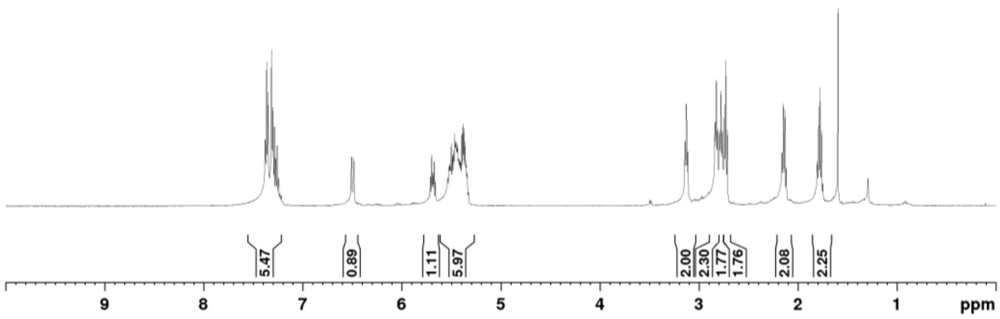

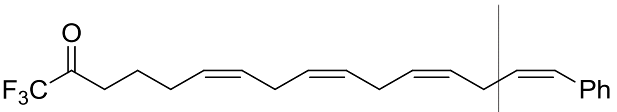

1d

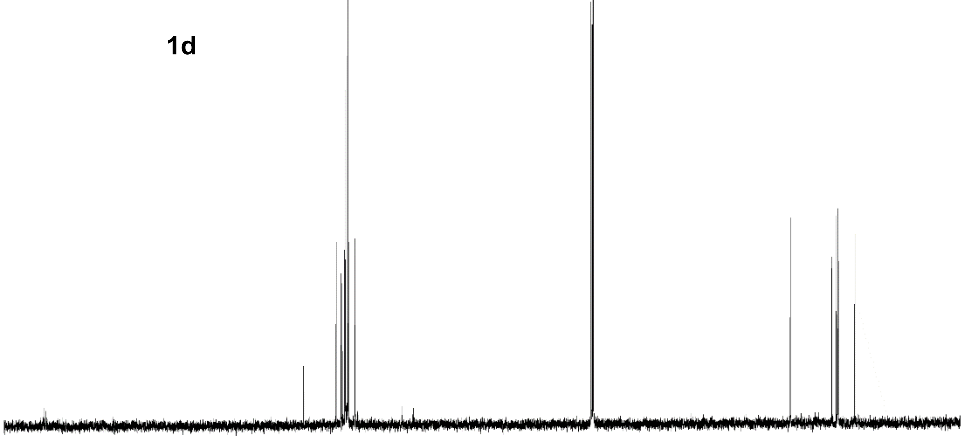

(5Z,8Z,11Z,14Z)-1,1,1-trifluorohenicosa-5,8,11,14-tetraen-2-one (1e): Yellow oil, 64% yield (over 2 steps). ^{1}H NMR (500 MHz, $CDCl_3$) δ 5.41 – 5.32 (m, 8H), 2.85 – 2.77 (m, 6H), 2.47-2.43 (m, 2H), 2.05 (dd, *J* = 13.8, 6.9 Hz, 2H), 1.30– 1.26 (m, 10H), 0.88 (t, *J* = 6.7 Hz, 3H); ^{13}C NMR (125 MHz, $CDCl_3$) δ 190.85 (d, *J* = 35.2 Hz), 130.53, 130.36, 128.70, 128.62, 127.69, 127.61, 127.48, 126.47, 115.52 (d, *J* = 292.1 Hz), 36.33, 31.76, 29.60, 28.98, 27.26, 25.64, 25.63, 25.57, 22.63, 20.30, 14.06. HRMS (APCI): calculated for $C_{21}H_{32}F_3O$ (M+1): 357.2400; found 357.2411.

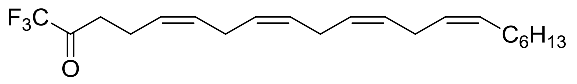

1e

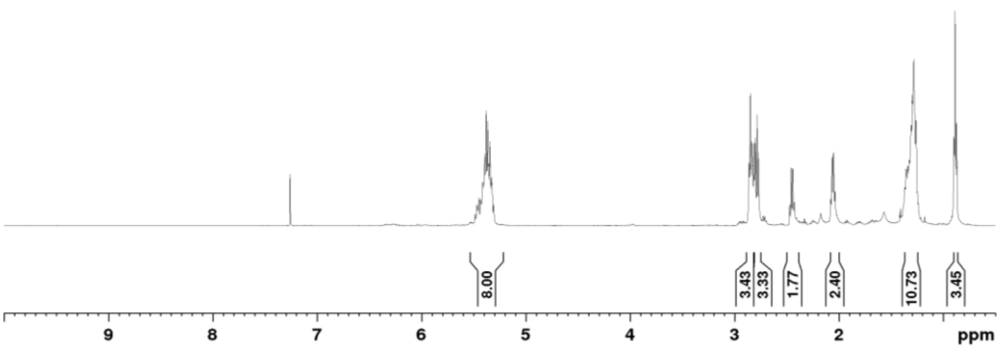

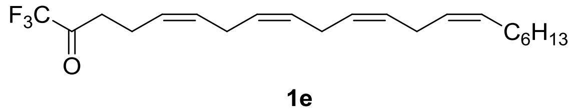

1e

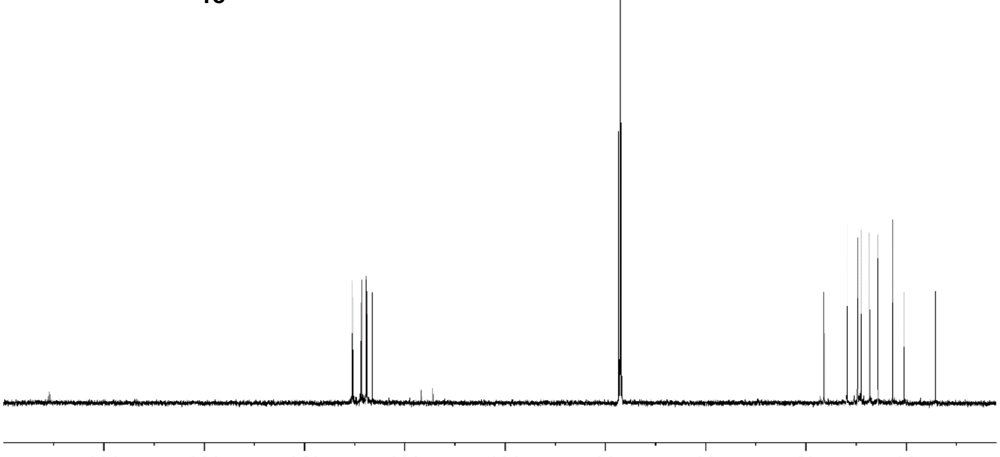

(6Z,9Z,12Z)-1,1,1-trifluorooctadeca-6,9,12-trien-2-one (1f): Yellow oil, 58% yield (over 2 steps). ^{1}H NMR (500 MHz, $CDCl_3$) δ 5.53 – 5.26 (m, 6 H), 2.83 (s, 2 H), 2.75 (t, *J* = 7.1 Hz, 2 H), 2.15 (dt, *J* = 12.5, 7.3 Hz, 2 H), 2.13 – 1.98 (m, 2 H), 1.85 – 1.72 (m, 2 H), 1.42 – 1.22 (m, 8 H), 0.92 (t, *J* = 6.7 Hz, 3 H); ^{13}C NMR (125 MHz, $CDCl_3$) δ 191.44 (d, *J* = 35 Hz), 130.53, 129.71, 128.69, 128.10, 127.61, 127.44, 115.57 (d, *J* = 291 Hz), 35.59, 31.52, 29.32, 27.22, 25.99, 25.63, 25.58, 22.57, 22.14, 14.03. HRMS (APCI): calculated for $C_{18}H_{26}F_3O$ (M-1): 315.1941; found 315.1939.

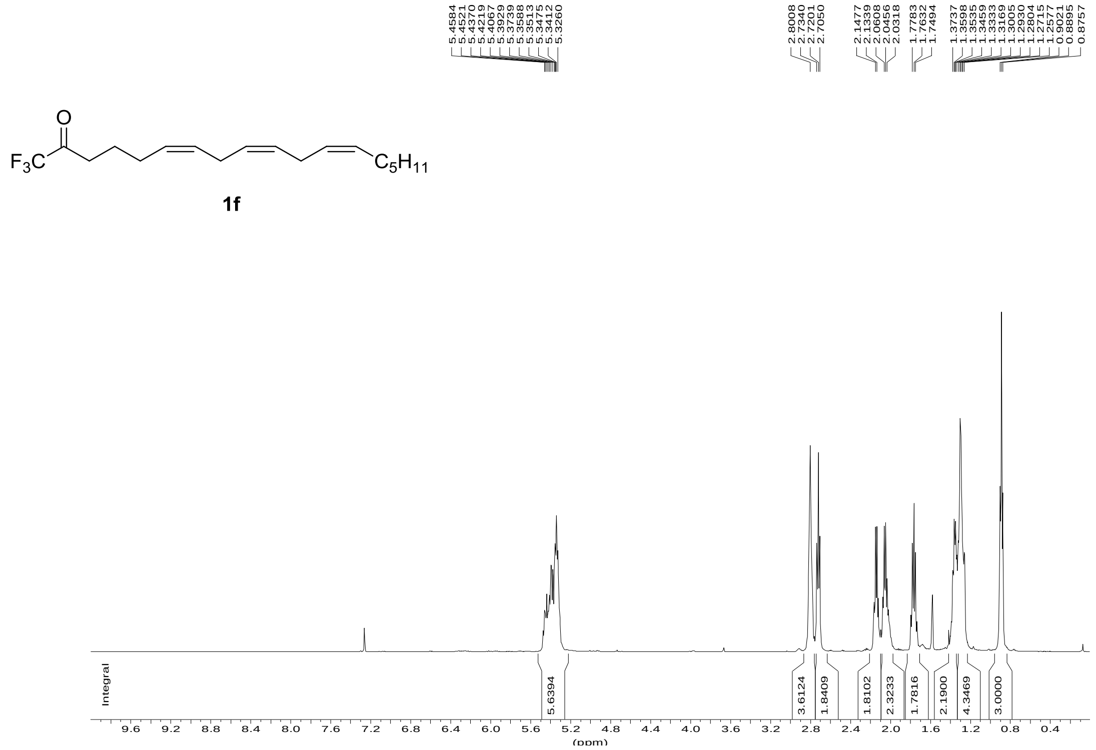

1f

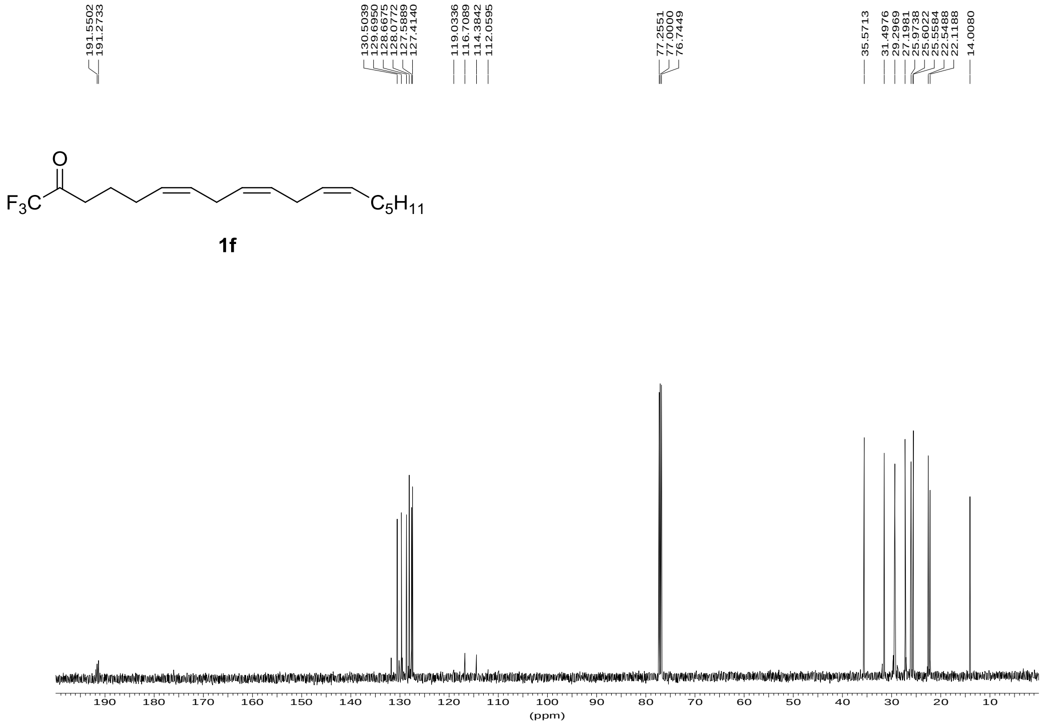

1f

(6Z,9Z,12Z)-1,1,1-trifluorohexadeca-6,9,12-trien-2-one (1g): Yellow oil, 51% yield (over 2 steps). ^{1}H NMR (500 MHz, $CDCl_3$) δ 5.44 – 5.33 (m, 6H), 2.82 – 2.79 (m, 4H), 2.72 (t, *J* = 7.2 Hz, 2H), 2.14 (dd, *J* = 14.6, 7.3 Hz, 2H), 2.04 (dd, *J* = 14.0, 7.6 Hz, 2H), 1.76 (m, 2H), 1.39 (dd, *J* = 14.7, 7.4 Hz, 2H), 0.91 (t, *J* = 7.4 Hz, 3H); ^{13}C NMR (125 MHz, $CDCl_3$) δ 191.41 (q, *J* = 34.9 Hz), 130.23, 129.71, 128.68, 128.08, 127.65, 127.61, 115.56 (d, *J* = 292.3 Hz), 35.59, 29.29, 25.99, 25.63, 25.57, 22.73, 22.15, 13.71.HRMS (APCI): calculated for $C_{16}H_{24}F_3O$ (M+1): 289.1774; found 289.1780.

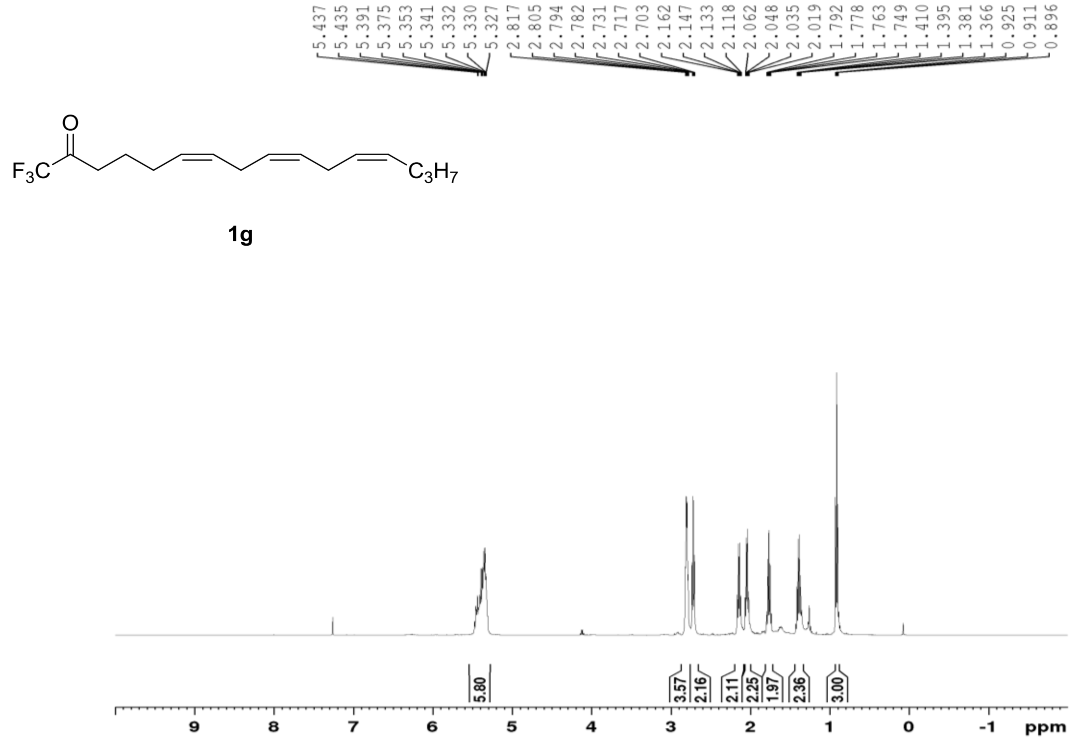

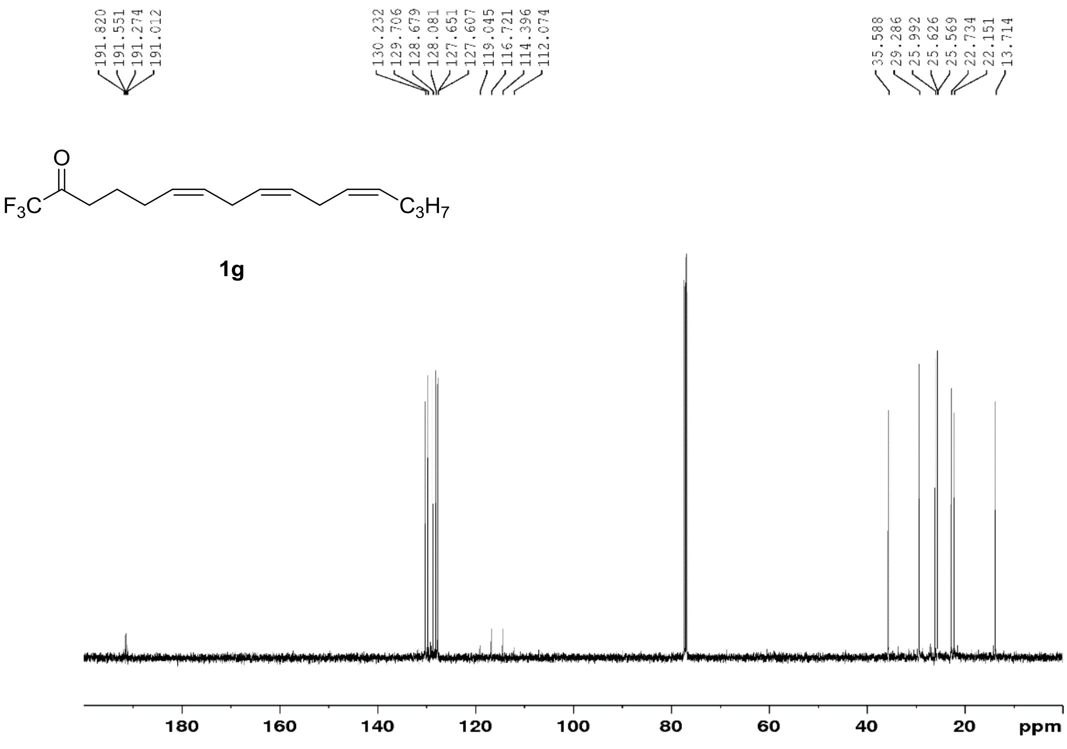

(6Z,9Z,12Z)-1,1,1-trifluorohenicosa-6,9,12-trien-2-one (1h): Yellow oil, 60% yield (over 2 steps). ^{1}H NMR (300 MHz, $CDCl_3$) δ 5.49 – 5.24 (m, 6 H), 2.76 (dt, *J* = 14.2, 6.7 Hz, 6 H), 2.10 (ddd, *J* = 20.1, 13.7, 6.8 Hz, 4 H), 1.84 – 1.68 (m, 2 H), 1.19 – 1.41 (s, 12 H), 0.88 (t, *J* = 5.8 Hz, 3 H); ^{13}C NMR (125 MHz, $CDCl_3$) δ 191.43 (d, *J* = 35 Hz), 130.54, 129.72, 128.70, 128.10, 127.61, 127.43, 115.57 (d, *J* = 290 Hz), 35.60, 31.90, 29.65, 29.52, 29.33, 29.31, 27.26, 26.00, 25.63, 25.59, 22.68, 22.15, 14.08. HRMS (APCI): calculated for $C_{21}H_{32}F_3O$ (M-1): 357.2411; found 357.2399.

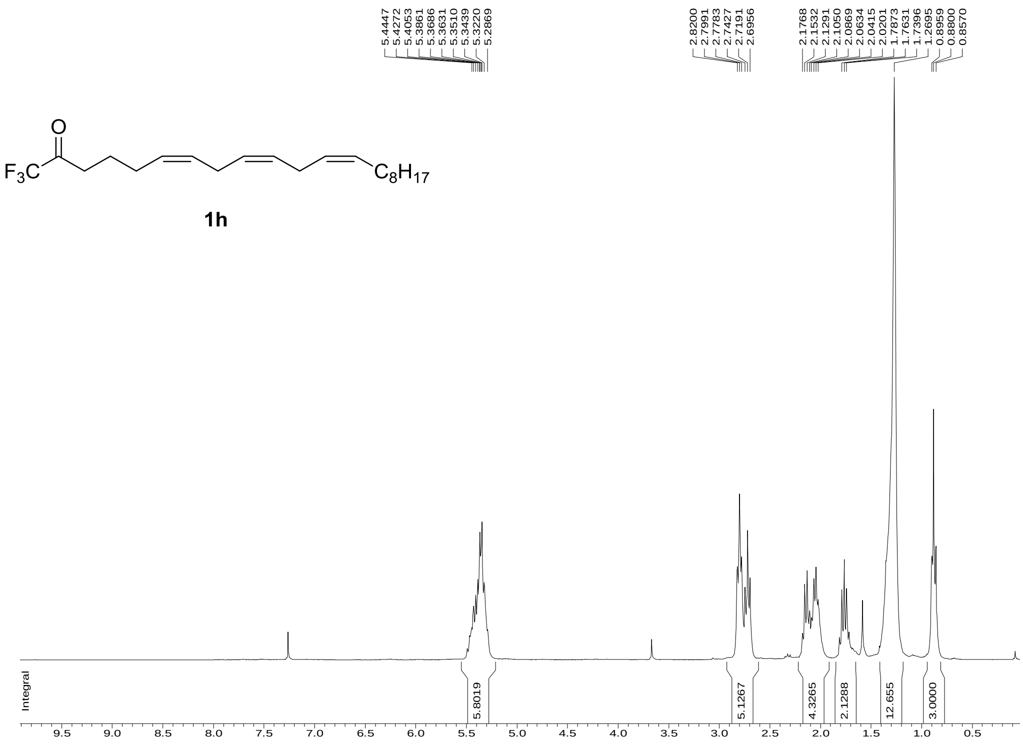

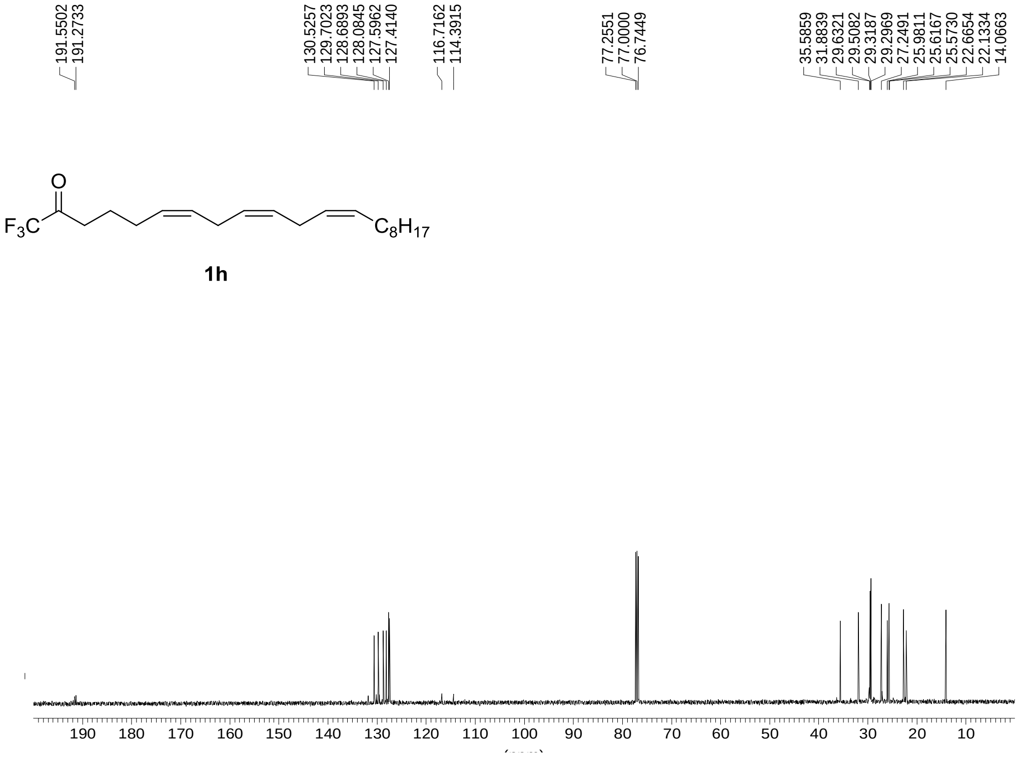

(6Z,9Z)-1,1,1-trifluoropentadeca-6,9-dien-2-one (1i): Pale yellow oil, 75% yield (over 2 steps). ^{1}H NMR (500 MHz, $CDCl_3$) δ 5.46 – 5.37 (m, 2H), 5.36 – 5.28 (m, 2H), 2.76 (t, *J* = 7.2 Hz, 2H), 2.72 (t, *J* = 7.2 Hz, 2H), 2.14 (dd, *J* = 14.5, 7.2 Hz, 2H), 2.04 (dd, *J* = 14.2, 7.1 Hz, 2H), 1.76 (p, *J* = 7.2 Hz, 2H), 1.34 – 1.28 (m, 6H), 0.89 (t, *J* = 6.7 Hz, 3H); ^{13}C NMR (125 MHz, $CDCl_3$) δ 191.45 (d, *J* = 34.8 Hz), 130.61, 130.03, 127.86, 127.30, 115.57 (q, *J* = 292.3 Hz), 35.61, 31.50, 29.28, 27.21, 25.99, 25.58, 22.54, 22.18, 14.01.HRMS (APCI): calculated for $C_{15}H_{22}F_3O$ (M-1): 275.1628; found 275.1620.

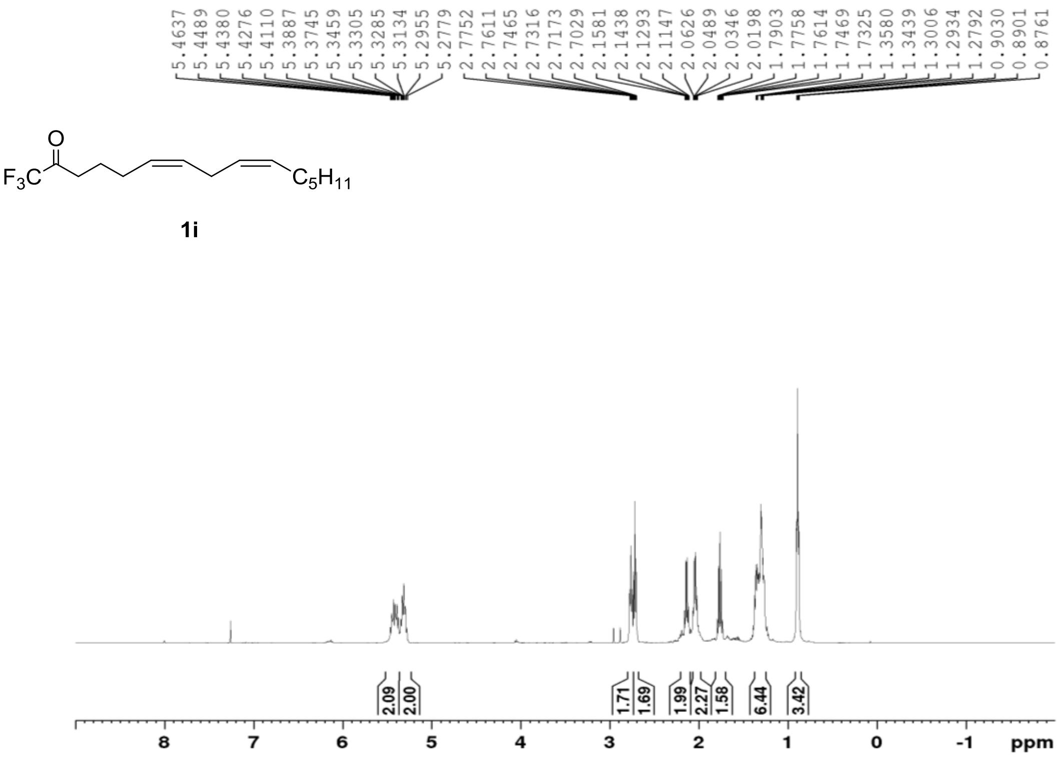

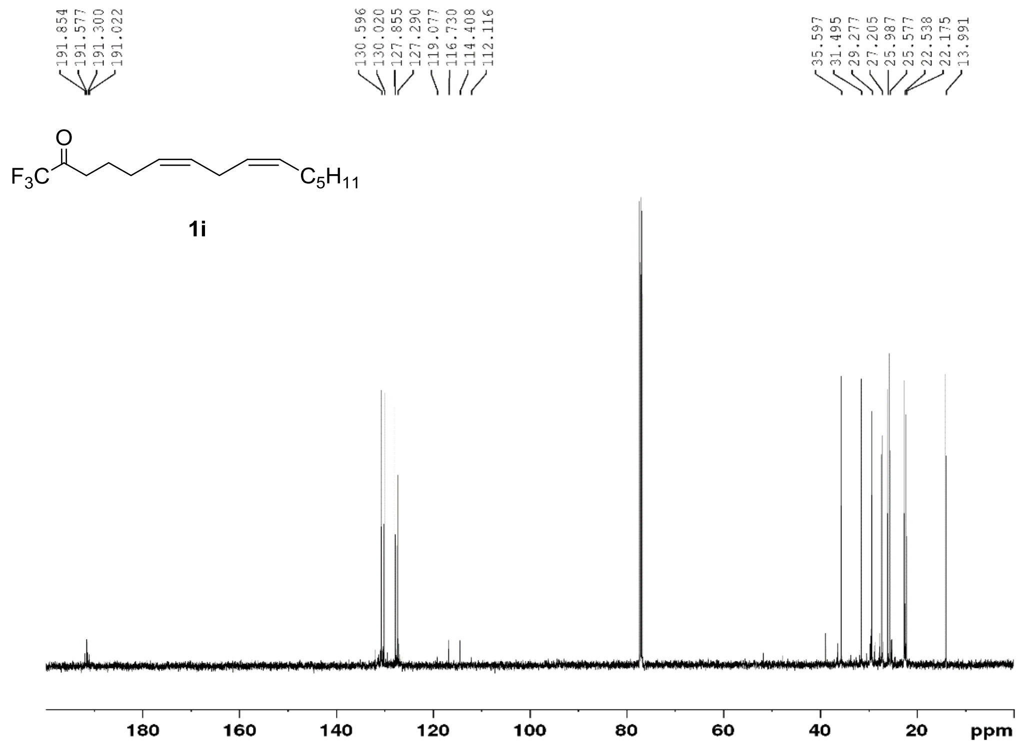

(6Z,9Z)-1,1,1-trifluoroheptadeca-6,9-dien-2-one (1j): Yellow oil, 65% yield (over 2 steps). ^{1}H NMR (500 MHz, $CDCl_3$) δ 5.52 – 5.28 (m, 4 H), 2.79 (t, J = 7.2 Hz, 2 H), 2.74 (t, J = 7.2 Hz, 2 H), 2.16 (dd, J = 14.3, 7.1 Hz, 2 H), 2.07 (dd, J = 14.1, 7.0 Hz, 2 H), 1.79 (p, J = 7.2 Hz, 2 H), 1.42 – 1.22 (m, 10 H), 0.91 (t, J = 6.9 Hz, 3 H); ^{13}C NMR (125 MHz, $CDCl_3$) δ 191.43 (d, J = 34 Hz), 130.59, 130.02, 127.85, 127.28, 115.57 (q, J = 291 Hz), 35.58, 31.86, 29.62, 29.27, 29.21, 27.25, 25.98, 25.58, 22.65, 22.15, 14.04. HRMS (APCI): calculated for $C_{17}H_{26}F_3O$ (M-1) 303.1941, found 303.1946.

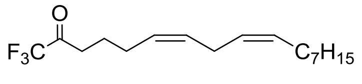

1j

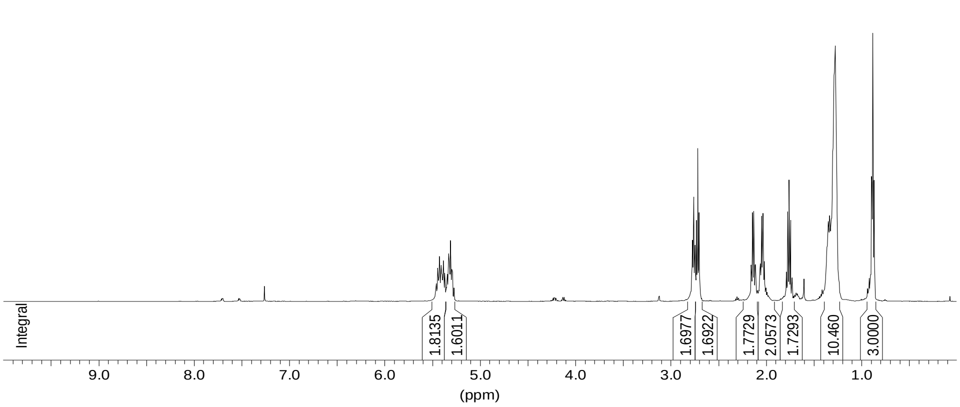

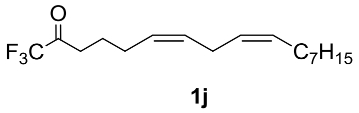

1j

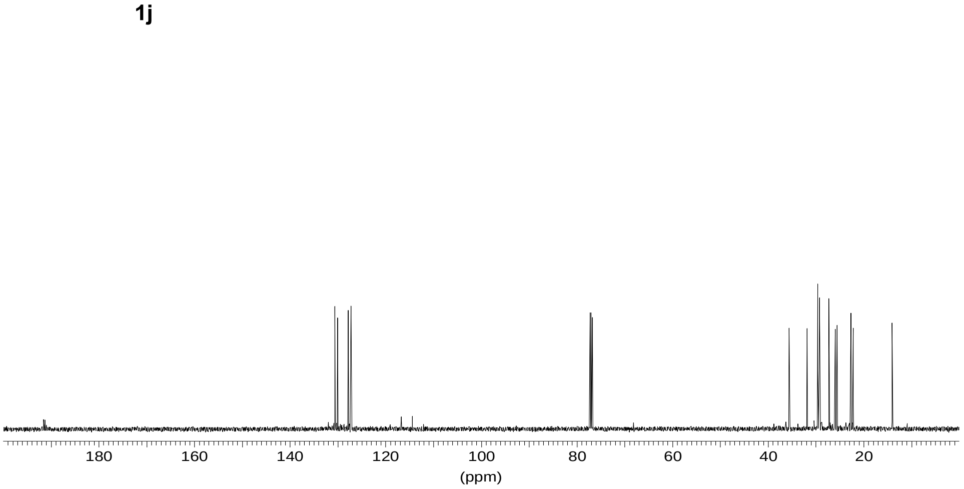

(6Z,9Z)-1,1,1-trifluorohenicosa-6,9-dien-2-one (1k): Yellow oil, 47% yield (over 2 steps). ^{1}H NMR (500 MHz, $CDCl_3$) δ 5.53 – 5.24 (m, 4 H), 2.77 (dt, *J* = 21.1, 7.1 Hz, 4 H), 2.16 (dd, *J* = 14.2, 7.0 Hz, 2 H), 2.07 (dd, *J* = 13.9, 6.9 Hz, 2 H), 1.83 – 1.73 (m, 2 H), 1.29 (s, 18 H), 0.91 (t, *J* = 6.9 Hz, 3 H); ^{13}C NMR (125 MHz, $CDCl_3$) δ 191.45 (q, *J* = 36 Hz), 130.62, 130.04, 127.86, 127.28, 115.57 (d, *J* = 290 Hz), 35.60, 31.92, 29.68 (2 C), 29.64 (2 C), 29.56, 29.35, 29.32, 27.26, 25.99, 25.59, 22.69, 22.17, 14.08. HRMS (APCI): calculated for $C_{21}H_{34}F_3O$ (M-1) 359.2567, found 359.2551.

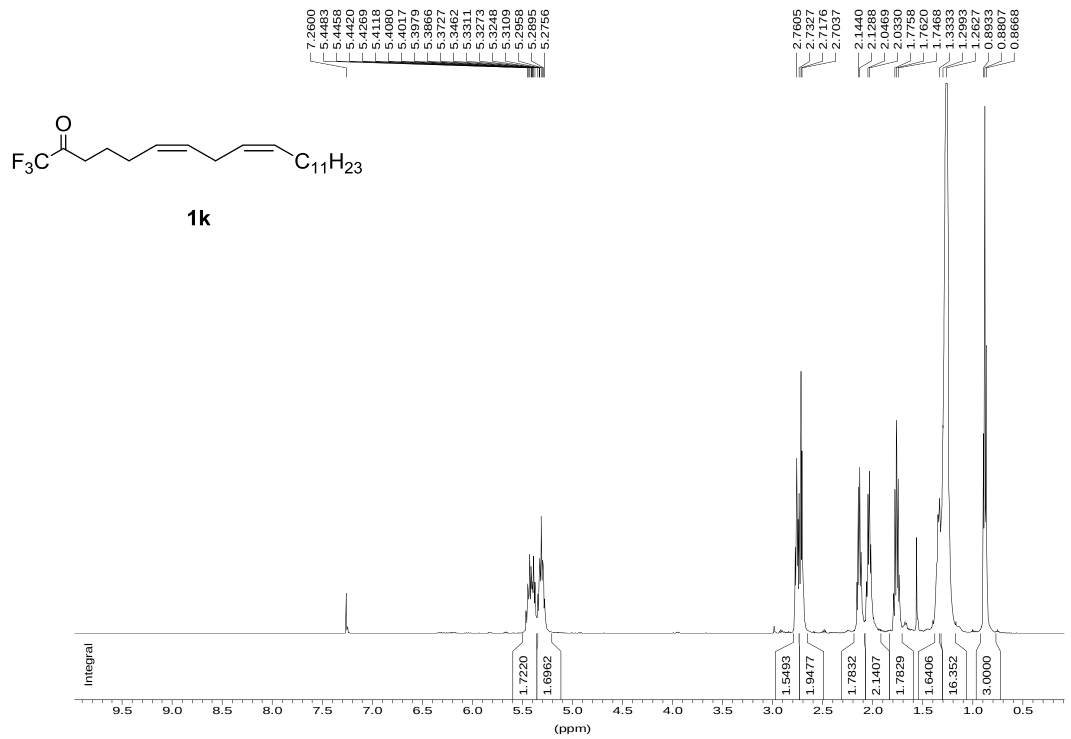

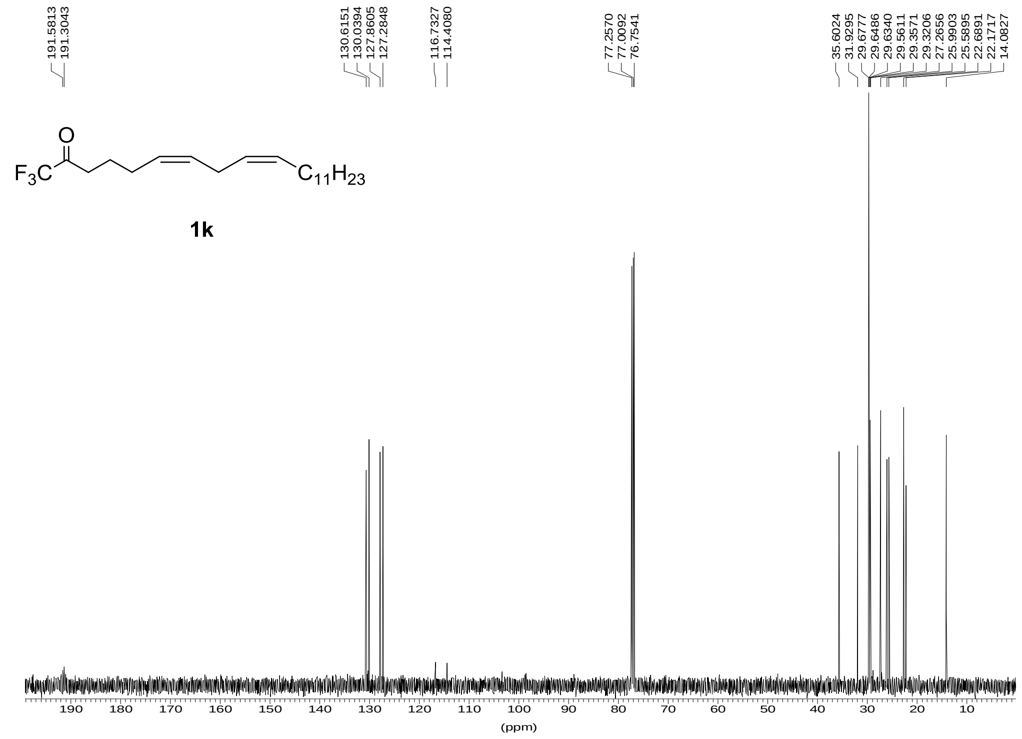

(6Z,9Z)-1,1,1-trifluorotricosa-6,9-dien-2-one (1l): Yellow oil, 46% yield (over 2 steps). ^{1}H NMR (500 MHz, $CDCl_3$) δ 5.50 – 5.39 (m, 2 H), 5.39 – 5.29 (m, 2 H), 2.79 (t, *J* = 7.2 Hz, 2 H), 2.74 (t, *J* = 7.2 Hz, 2 H), 2.17 (q, *J* = 7.1 Hz, 2 H), 2.11 – 1.99 (m, 2 H), 1.79 (p, *J* = 7.2 Hz, 2 H), 1.37 (dd, *J* = 15.9, 8.5 Hz, 2 H), 1.31 (d, *J* = 19.3 Hz, 20 H), 0.91 (t, *J* = 6.9 Hz, 3 H); ^{13}C NMR (125 MHz, $CDCl_3$) δ 191.42 (q, *J* = 34 Hz), 130.61, 130.04, 127.85, 127.28, 115.58 (q, *J* = 291 Hz), 35.60, 31.92, 29.69 (2 C), 29.67, 29.65, 29.64, 29.63, 29.55, 29.36, 29.32, 27.26, 25.99, 25.59, 22.68, 22.18, 14.07; HRMS (APCI): calculated for $C_{23}H_{38}F_3O$ (M-1) 387.2880, found 387.2863.

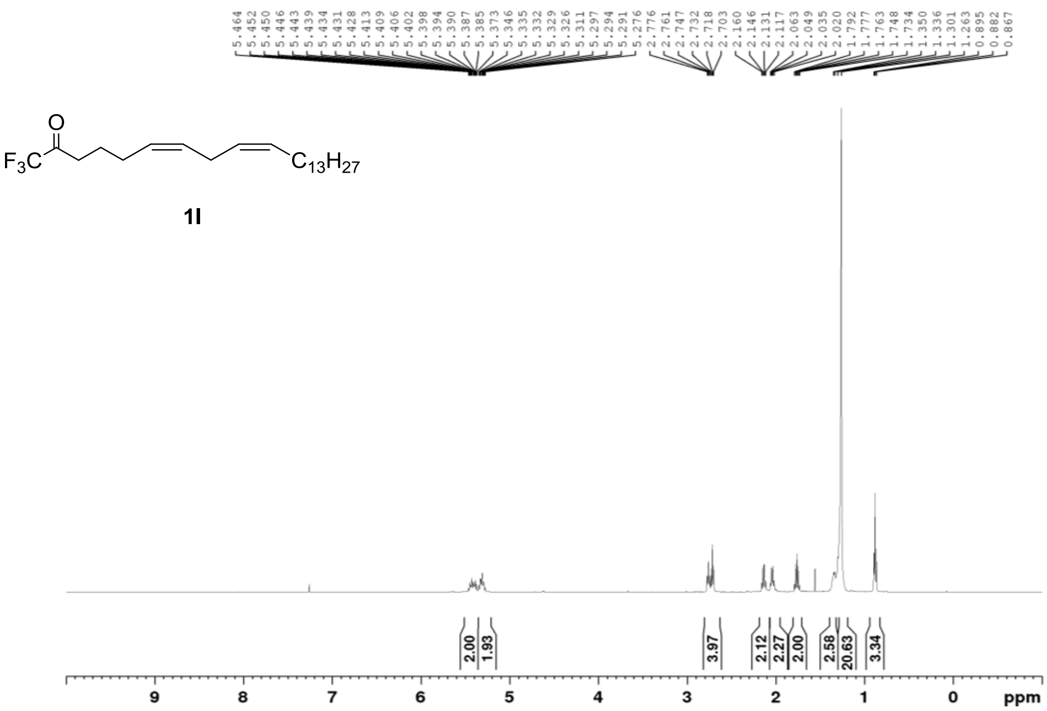

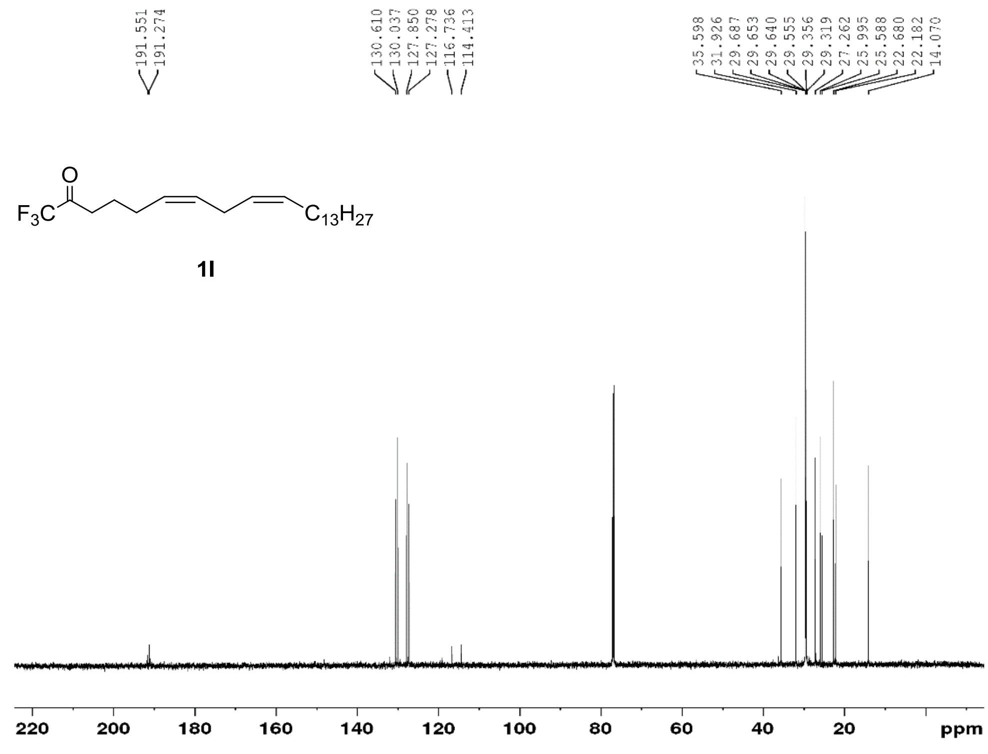

Synthesis of (5Z,8Z,11Z,14Z)-icosa-5,8,11,14-tetraenamide (1m): To **9a** (67 mg, 0.21 mmol) was added 7M ammonia in methanol (0.30 mL, 2.1 mmol) and 7% magnesium methoxide in methanol (0.32 mL, 0.21 mmol) and the reaction mixture was heated in a sealed tube at 80°C overnight. When no starting material was observed, the mixture was cooled to room temperature, quenched with saturated NaCl and extracted with ethyl acetate. The combined organic phase was dried with $MgSO_4$, concentrated and purified by flash column chromatography with a gradient elution of 3:1 hexane-ethyl acetate to 1:2 hexane-ethyl acetate system to afford **1m** as a yellow oil (20 mg, 31%). ^{1}H NMR (500 MHz, $CDCl_3$) δ 5.62 (s, 1H), 5.42 – 5.29 (m, 8H), 2.84 – 2.79 (m, 4H), 2.24 – 2.21 (m, 2H), 2.16 – 2.11 (m, 2H), 2.05 (dd, J = 14.0, 6.9 Hz, 2H), 1.72 (dt, J = 14.7, 7.4 Hz, 2H), 1.35 – 1.27 (m, 8H), 0.88 (dd, J = 8.8, 4.7 Hz, 3H); ^{13}C NMR (125 MHz, $CDCl_3$) δ 175.33, 130.53, 129.01, 128.90, 128.62, 128.26, 128.16, 127.86, 127.53, 35.16, 31.51, 29.31, 27.22, 26.58, 25.65(2C), 25.26, 22.56, 14.09, 14.05. HRMS (APCI): calculated for $C_{20}H_{34}NO$(M+1) 304.2640; found 304.2645.

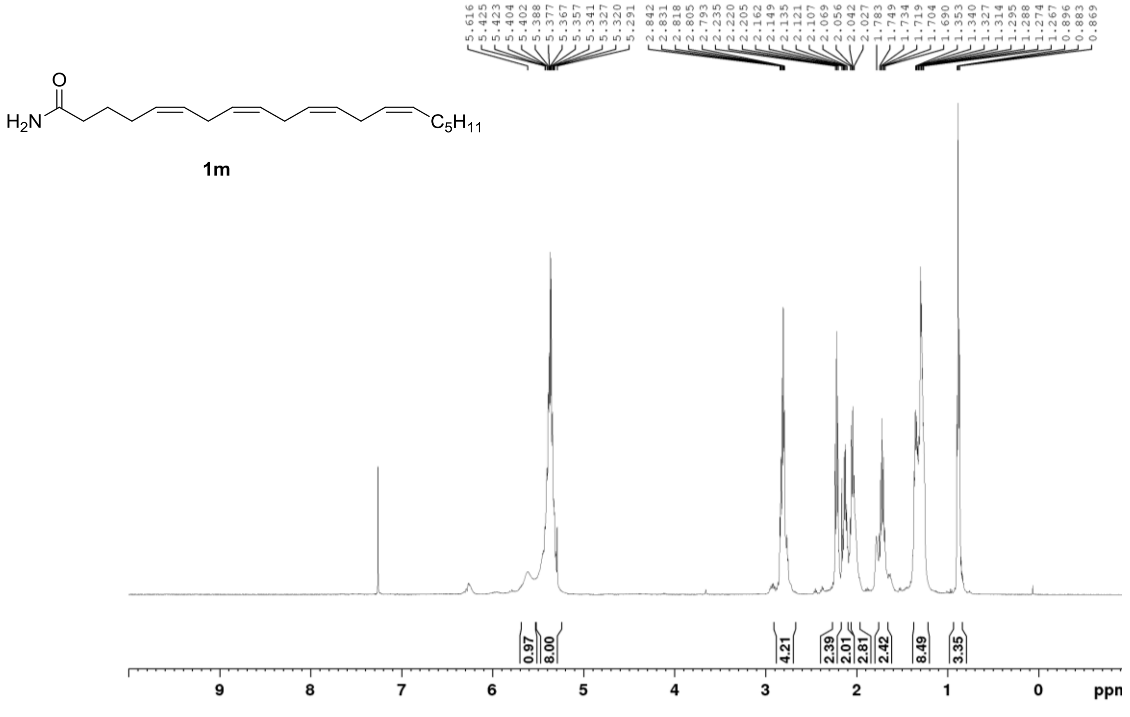

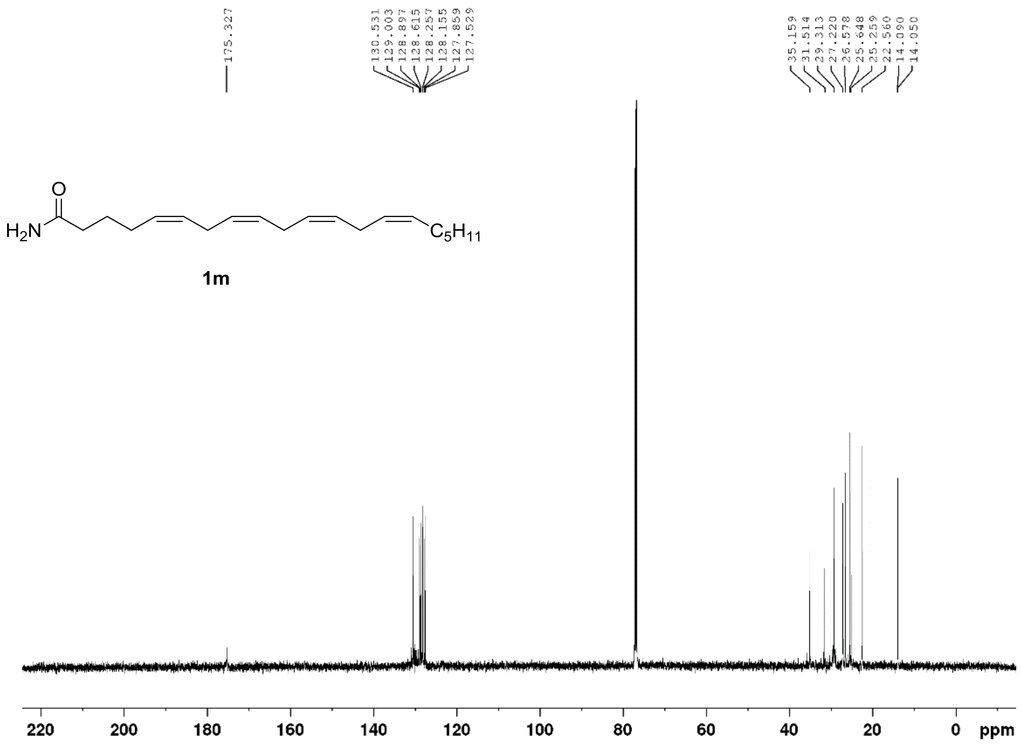

1m

(6Z,9Z,12Z,15Z)-1-chloro-1,1-difluorohenicosa-6,9,12,15-tetraen-2-one (1n): Yellow oil, 46% yield. ^{1}H NMR (500 MHz, $CDCl_3$) δ 5.54 – 5.27 (m, 6 H), 2.91 – 2.71 (m, 6 H), 2.16 (dt, *J* = 12.1, 7.5 Hz, 2 H), 2.08 (dd, *J* = 14.1, 7.0 Hz, 2 H), 1.87 – 1.73 (m, 2 H), 1.44 – 1.22 (m, 8 H), 0.88 (t, *J* = 3.15 Hz. 3 H); ^{13}C NMR (125 MHz, $CDCl_3$) δ 191.77, 130.52, 129.58, 128.64, 128.39, 128.25, 127.90, 127.78, 127.51, 119.81 (t, *J* = 304 Hz), 34.36, 31.53, 29.32, 27.23, 26.07, 25.64 (2 C), 25.62, 22.68, 22.57, 14.05. HRMS (APCI): calculated for $C_{21}H_{32}ClF_2O$ (M+1) 373.2104, found 373.2117.

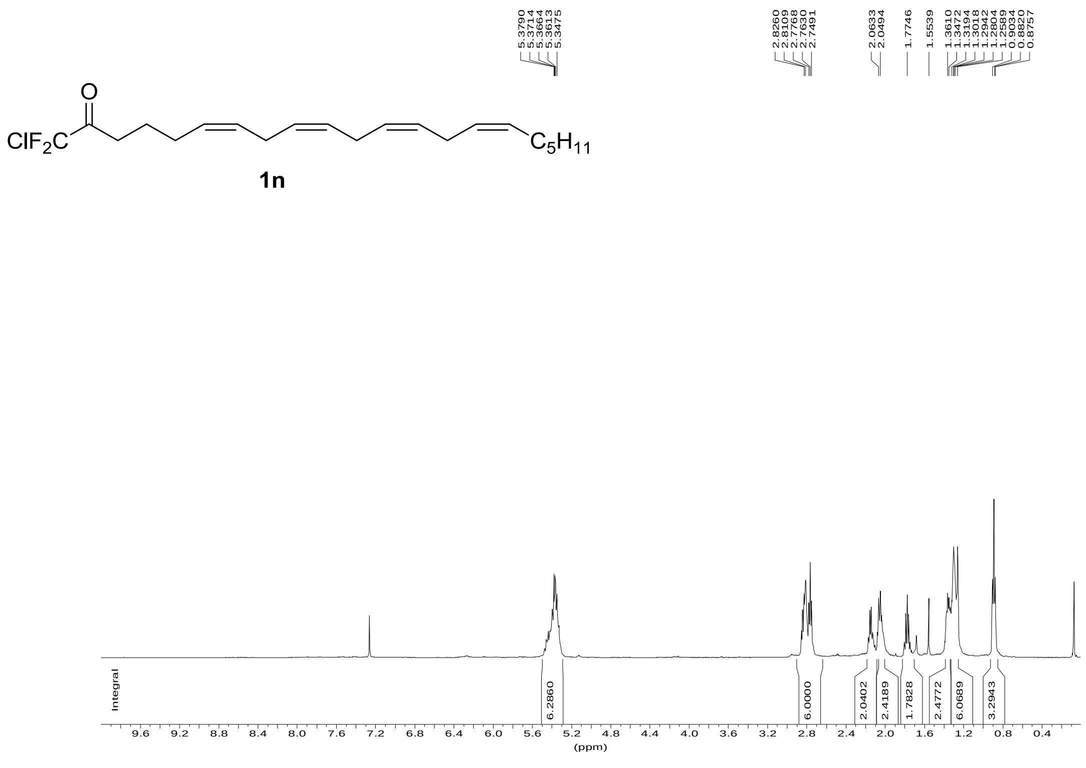

1n

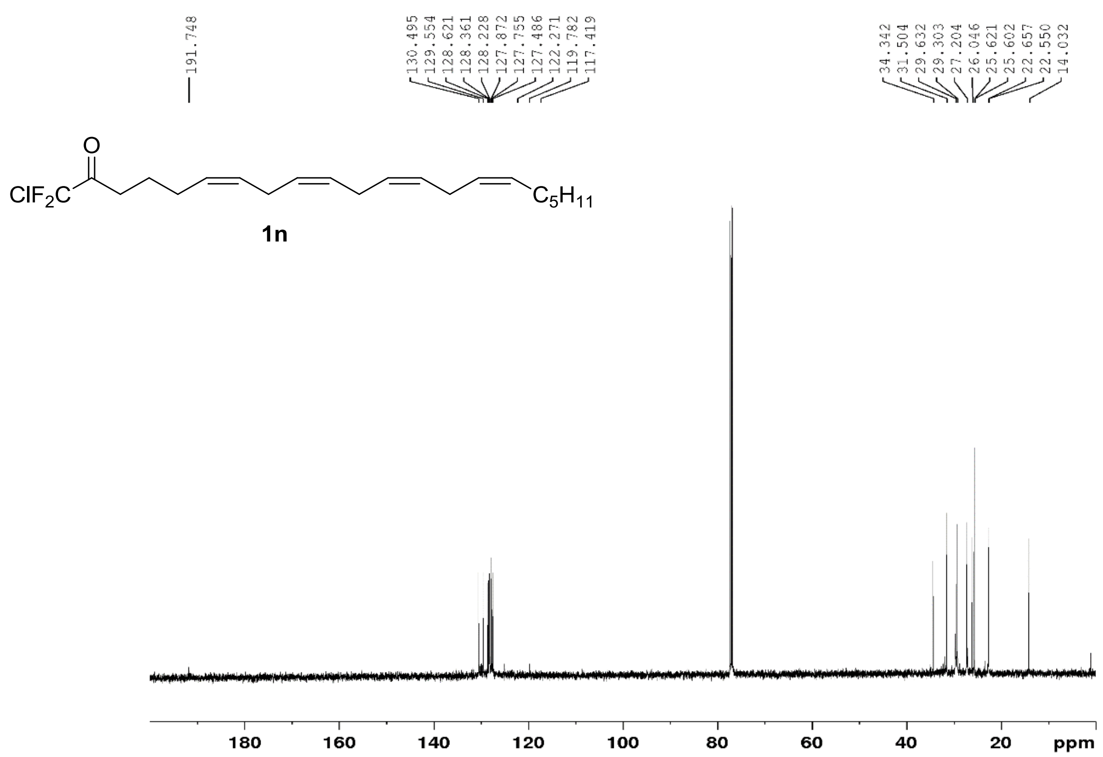

1n

(6Z,9Z,12Z,15Z)-1,1-difluorohenicosa-6,9,12,15-tetraen-2-one (1o): Yellow oil, 51% yield. ^{1}H NMR (500 MHz, $CDCl_3$) δ 5.67 (t, *J* = 54.1 Hz, 1H), 5.40 – 5.34 (m, 8H), 2.85 – 2.80 (m, 6H), 2.69 – 2.66 (m, 2H), 2.15 – 2.12 (m, 2H), 2.06 (t, *J* = 7.2 Hz, 2H), 1.77 – 1.71 (m, 2H), 1.37 – 1.33 (m, 2H), 1.31 – 1.28 (m, 4H), 0.89 (dd, *J* = 9.0, 4.7 Hz, 5H); ^{13}C NMR (125 MHz, $CDCl_3$) δ 199.71 (t, *J* = 26.0 Hz), 130.50, 130.36, 129.31, 128.61, 128.54, 128.32, 127.97, 127.80, 127.51, 109.87 (t, *J* = 253.0 Hz), 35.29, 31.51, 29.31, 29.27, 27.21, 26.22, 25.62, 25.60, 22.56, 22.13, 14.04. HRMS (APCI): calculated for $C_{21}H_{31}F_2O$ (M-1) 337.2348; found 337.2345.

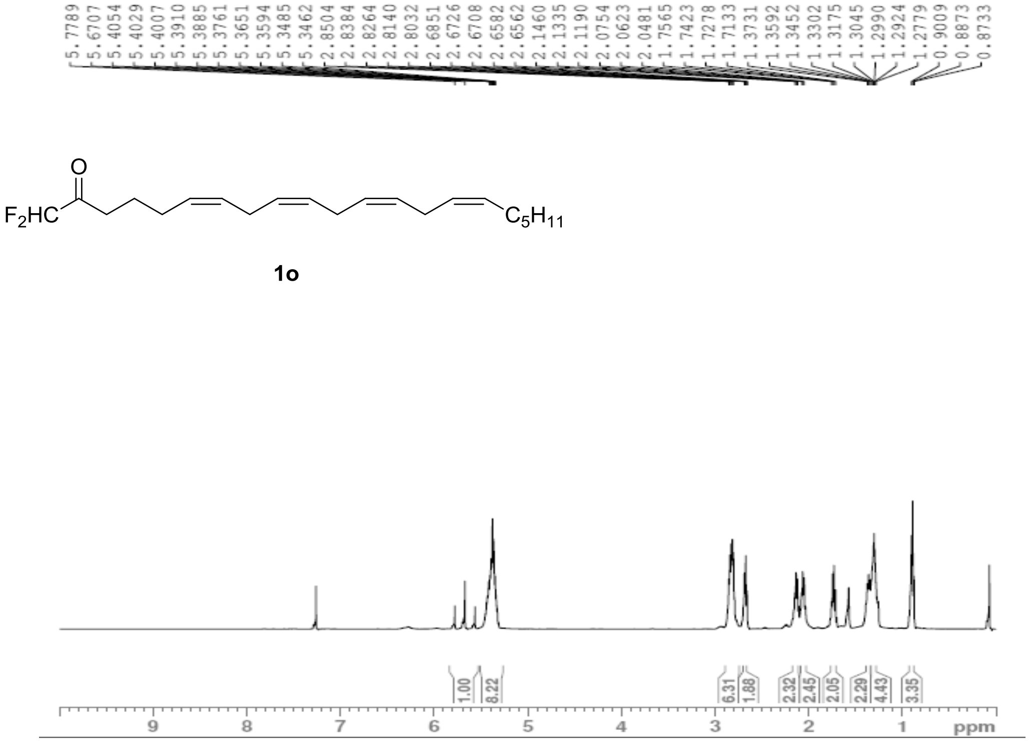

1o

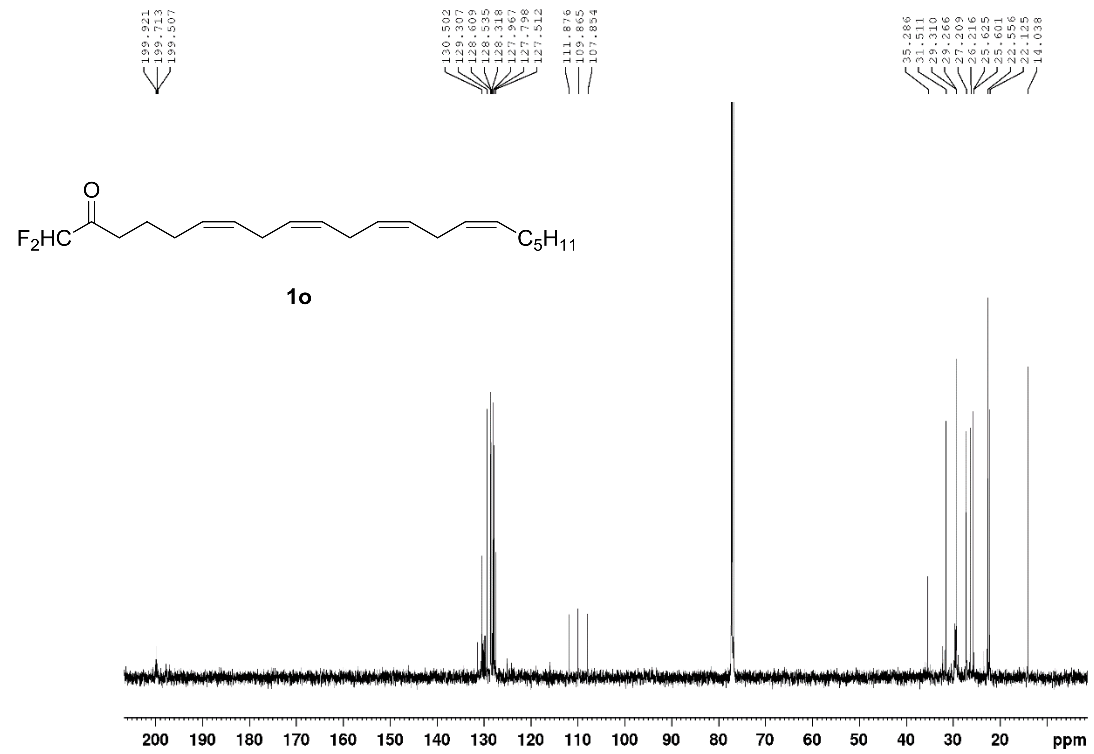

1o

4-((5Z,8Z,11Z,14Z)-icosa-5,8,11,14-tetraenamido)butanoic acid (2a): Pale yellow solid, 59% yield, eluent system: 2:1:0.03 hexane-ethyl acetate-AcOH. ^{1}H NMR (500 MHz, $CDCl_3$) δ 5.83 (s, 1 H), 5.44 – 5.34 (m, 8 H), 3.34(dd, *J* = 12.9, 6.7 Hz, 2 H), 2.87 – 2.81 (m, 4 H), 2.42 (t, *J* = 7.0 Hz, 2 H), 2.25 – 2.17 (m, 2H), 2.13 (dd, *J* = 13.8, 7.1 Hz, 2H), 2.08 (dd, *J* = 14.3, 7.2 Hz, 2H), 1.86 (p, *J* = 6.9 Hz, 2H), 1.76 – 1.70 (m, 2 H), 1.421 – 1.28 (m, 8 H), 0.91 (t, *J* = 6.7 Hz, 3 H); ^{13}C NMR (125 MHz, $CDCl_3$) δ 177.15, 173.72, 130.54, 129.01, 128.87, 128.63, 128.29, 128.14, 127.85, 127.53, 38.83, 36.05, 31.51, 29.69, 29.64, 29.31, 27.22, 26.65, 25.64 (2 C), 25.49, 24.83, 22.57, 14.05. HRMS (ESI): calculated for $C_{24}H_{39}NO_3Na$ (M+Na) 412.2822, found 412.2830.

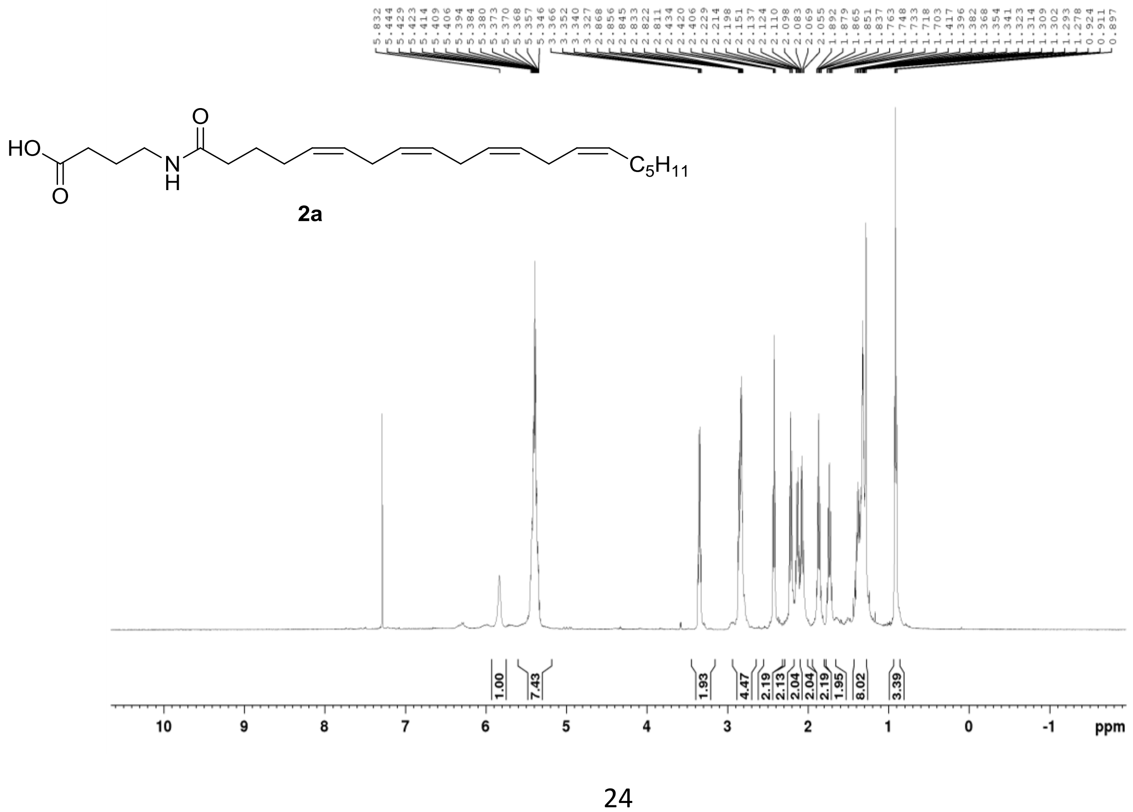

2a

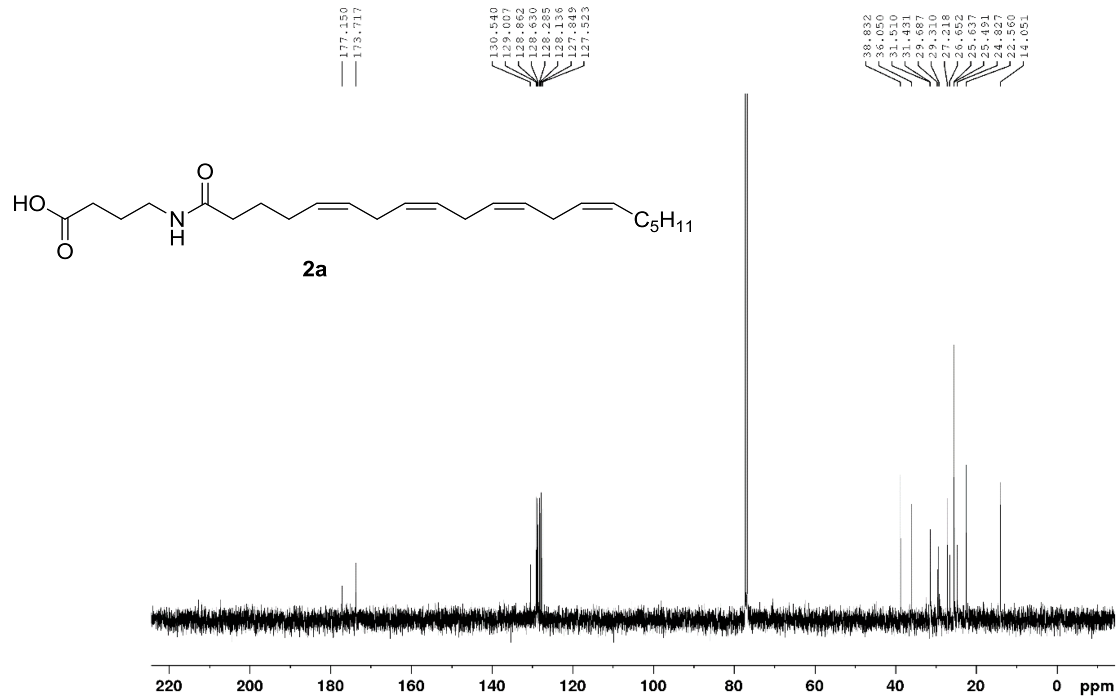

5-((5Z,8Z,11Z,14Z)-icosa-5,8,11,14-tetraenamido)pentanoic acid (2b): Pale yellow solid, 52% yield, eluent system: 2:1:0.03 hexane-ethyl acetate-AcOH. ^{1}H NMR (500 MHz, $CDCl_3$) δ 5.67 (s, 1 H), 5.50 – 5.28 (m, 8 H), 3.29 (d, *J* = 5.9 Hz, 2 H), 2.91 – 2.72 (m, 4 H), 2.40 (t, *J* = 7.1 Hz, 2 H), 2.24 – 2.17 (m, 2 H), 2.13 (dd, *J* = 13.1, 6.6 Hz, 2 H), 2.07 (dd, *J* = 14.2, 7.1 Hz, 2 H), 1.71 – 1.62 (m, 4 H), 1.59 (dd, *J* = 14.3, 6.9 Hz, 2 H), 1.44 – 1.24 (m, 8 H), 0.91 (t, *J* = 7.0 Hz, 3 H)[j]; ^{13}C NMR (125 MHz, $CDCl_3$) δ 178.06, 173.30, 130.53, 129.09, 128.79, 128.62, 128.25, 128.17, 127.86, 127.53, 39.05, 36.14, 33.41, 31.51, 29.31, 28.99, 27.22, 26.68, 25.64 (2 C), 25.56 (2 C), 22.56, 21.88, 14.05. HRMS (ESI): calculated for $C_{25}H_{42}NO_3$ (M+1) 404.3159, found 404.3164.

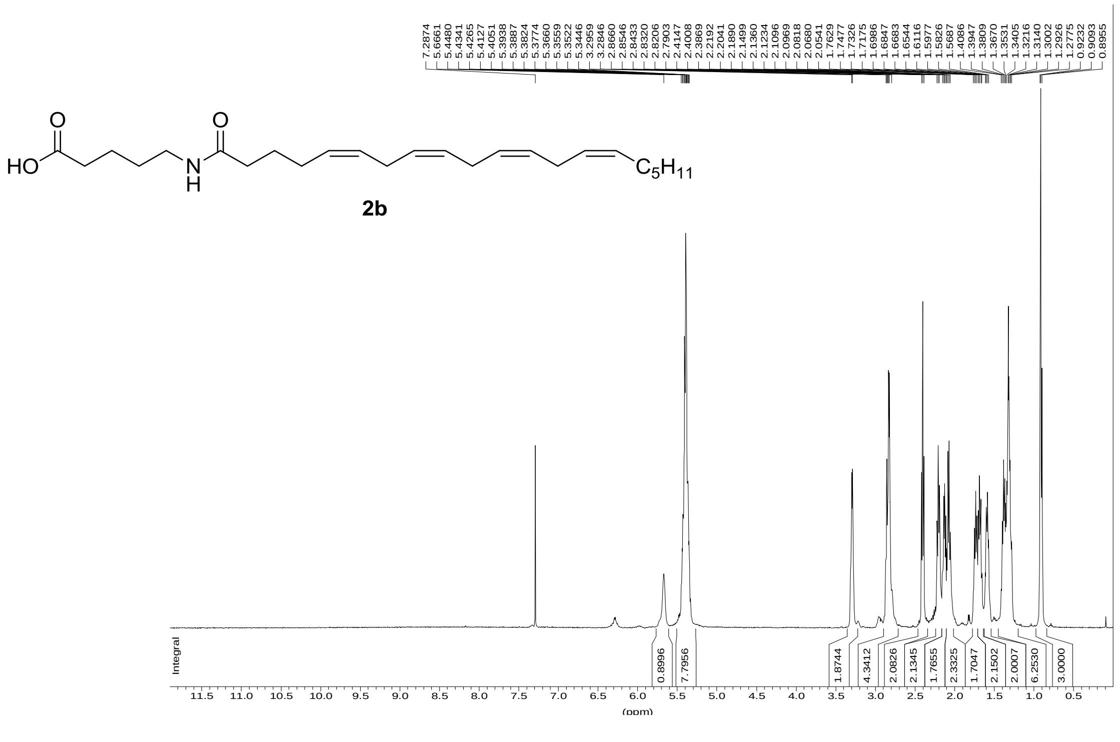

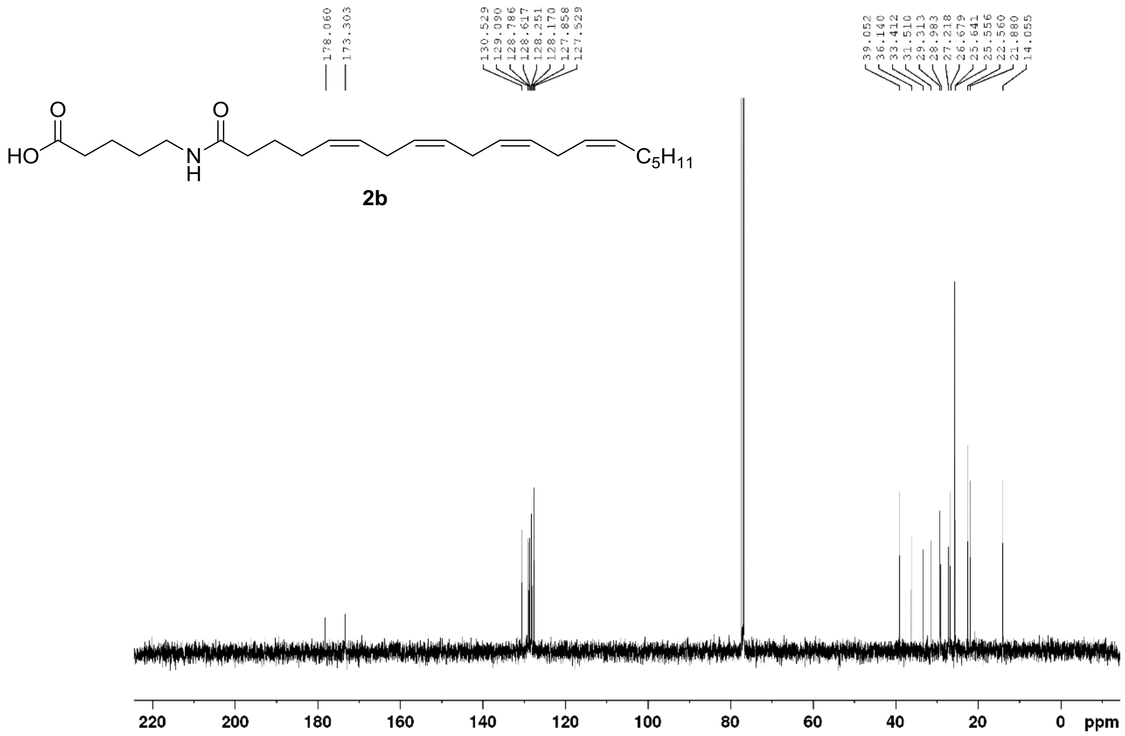

2b

6-((5Z,8Z,11Z,14Z)-icosa-5,8,11,14-tetraenamido)hexanoic acid (2c): Pale yellow solid, 98% yield, eluent system: 2:1:0.03 hexane-ethyl acetate-AcOH. ^{1}H NMR (500 MHz, $CDCl_3$) δ 5.57 (s, 1H), 5.38 – 5.34 (m, 8H), 3.25 (dd, *J* = 13.3, 6.8 Hz, 2H), 2.84 – 2.75 (m, 6H), 2.34 (t, *J* = 7.3 Hz, 2H), 2.18 – 2.15 (m, 2H), 2.11 – 2.08 (m, 2H), 2.07 – 2.02 (m, 2H), 1.72 – 1.63 (m, 4H), 1.53 – 1.50 (m, 2H), 1.39 – 1.34 (m, 4H), 1.32 – 1.25 (m, 4H), 0.88 (t, *J* = 6.8 Hz, 3H); ^{13}C NMR (125 MHz, $CDCl_3$) δ 178.25, 173.14, 130.50, 129.09, 128.73, 128.58, 128.22, 128.14, 127.83, 127.50, 39.25, 36.12, 33.77, 31.48, 29.29, 29.24, 27.19, 26.64, 26.24, 25.61(3C), 25.54, 24.23, 22.54, 14.03. HRMS (ESI): calculated for $C_{26}H_{42}NO_3$ (M-1) 416.3170, found 416.3170.

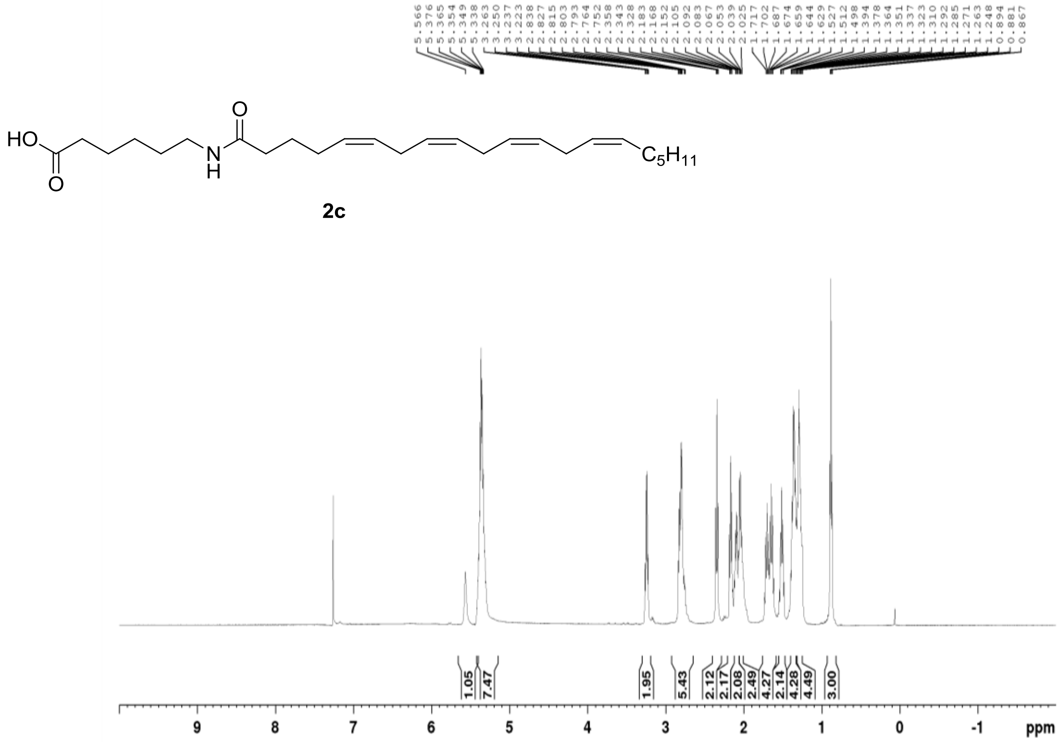

2c

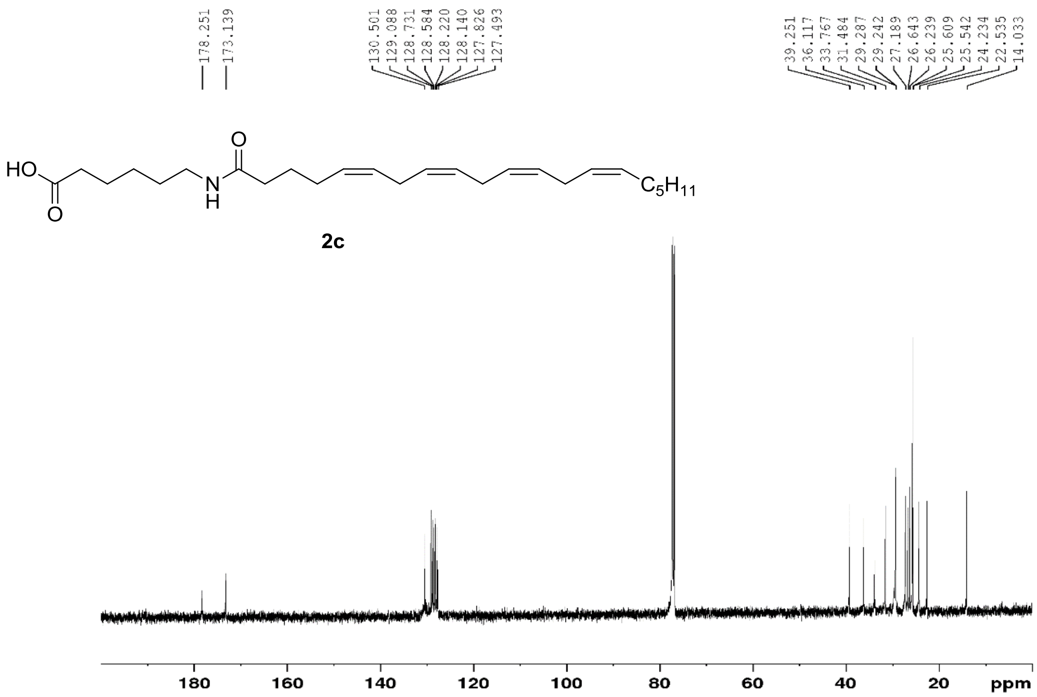

3-((5Z,8Z,11Z,14Z)-icosa-5,8,11,14-tetraenamido)benzoic acid (2d): Pale yellow solid, 63% yield, eluent system: 2:1:0.03 hexane-ethyl acetate-AcOH. ^{1}H NMR (500 MHz, DMSO) δ 12.91 (s, 1H), 10.05 (s, 1H), 8.22 (s, 1H), 7.82 (d, *J* = 8.0 Hz, 1H), 7.59 (d, *J* = 7.7 Hz, 1H), 7.39 (t, *J* = 7.9 Hz, 1H), 5.39 – 5.31 (m, 7H), 2.77 (dt, *J* = 11.5, 5.5 Hz, 4H), 2.32 (dd, *J* = 13.7, 6.3 Hz, 2H), 2.10 – 2.07 (m, 2H), 2.00 – 1.98 (m, 4H), 1.67 – 1.64 (m, 2H), 1.30 – 1.27 (m, 2H), 1.24 – 1.21 (m, 4H), 0.83 (t, *J* = 6.9 Hz, 3H); ^{13}C NMR (125 MHz, DMSO) δ 171.23, 167.18, 139.51, 131.21, 129.90, 129.29, 128.82, 128.20, 128.07, 127.95, 127.78, 127.62, 127.48, 123.70, 123.04, 119.76, 35.74, 30.86, 28.69, 26.58, 26.19, 25.21(2C), 25.18, 24.91, 21.95, 13.89. HRMS (ESI): calculated for $C_{27}H_{36}NO_3$ (M-1) 422.2701, found 422.2685.

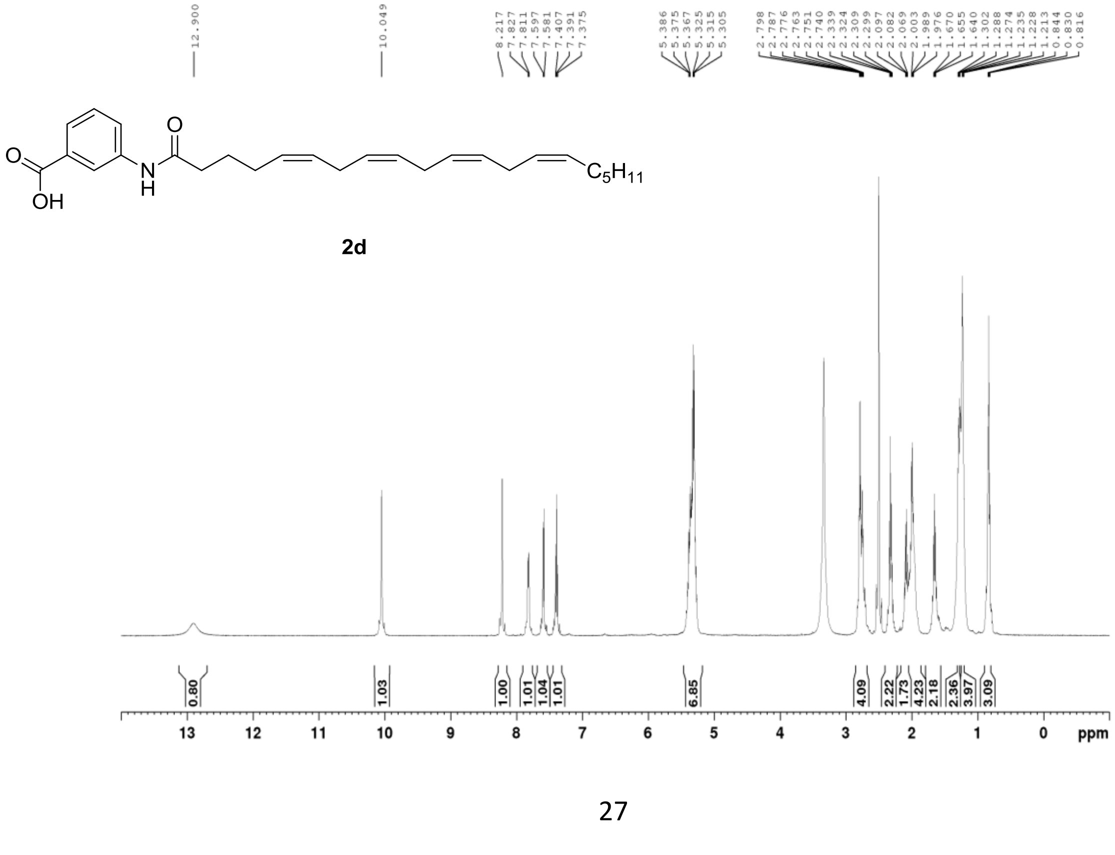

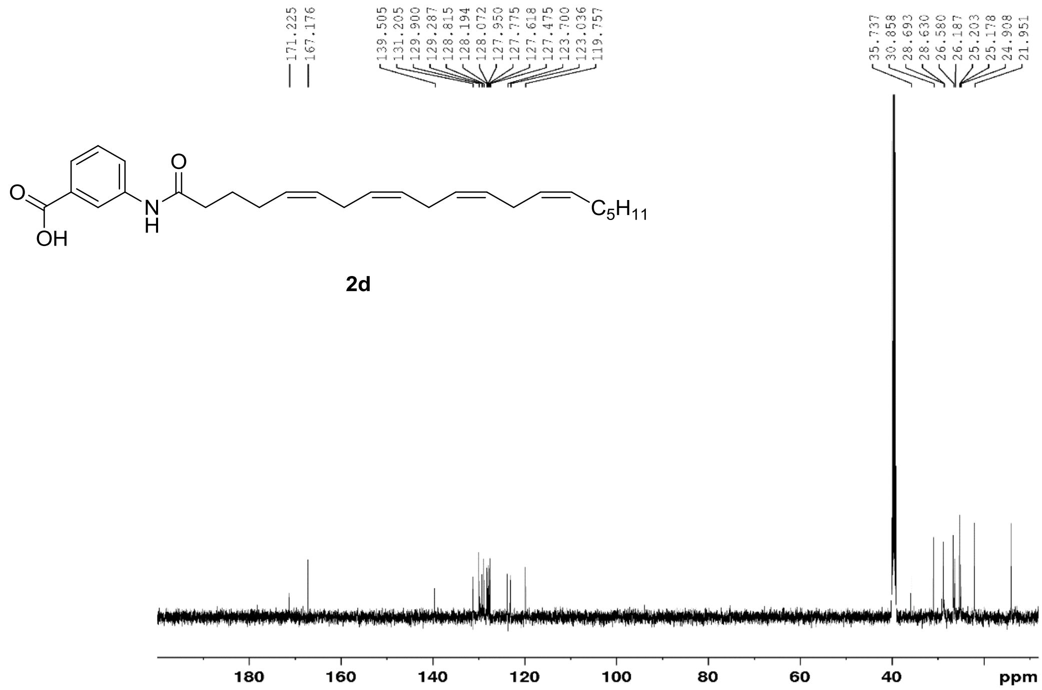

4-((5Z,8Z,11Z,14Z)-icosa-5,8,11,14-tetraenamidomethyl)benzoic acid (2e): Pale yellow solid, 95% yield, eluent system: 2:1:0.03 hexane-ethyl acetate-AcOH. ^{1}H NMR (500 MHz, DMSO) δ 12.83 (s, 1H), 8.37 (s, 1H), 7.88 (d, *J* = 8.2 Hz, 2H), 7.34 (d, *J* = 8.3 Hz, 2H), 5.36 – 5.32 (m, 9H), 4.32 (d, *J* = 5.9 Hz, 2H), 2.82 – 2.77 (m, 6H), 2.16 (t, *J* = 7.4 Hz, 2H), 2.03 (td, *J* = 13.9, 7.0 Hz, 4H), 1.62 – 1.56 (m, 2H), 1.32 – 1.28 (m, 2H), 1.27 – 1.22 (m, 4H), 0.84 (t, *J* = 7.0 Hz, 3H); ^{13}C NMR (125 MHz, DMSO) δ 172.48, 167.59, 145.37, 130.39, 129.83, 129.76, 129.70, 128.57, 128.51, 128.44, 128.24, 128.13, 127.96, 127.57, 42.24, 35.22, 31.31, 29.14, 27.05, 26.74, 25.69, 25.67, 25.65(2C), 22.40, 14.34.HRMS (ESI): calculated for $C_{28}H_{38}NO_3$ (M-1) 436.2587, found 436.2861.

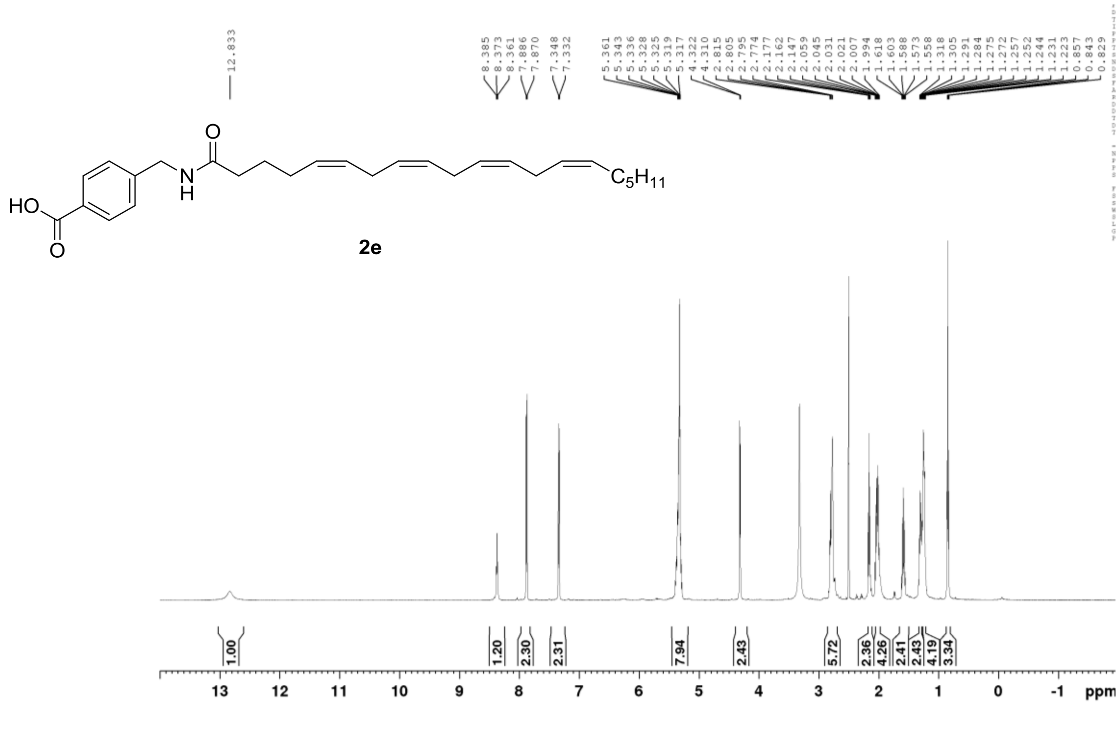

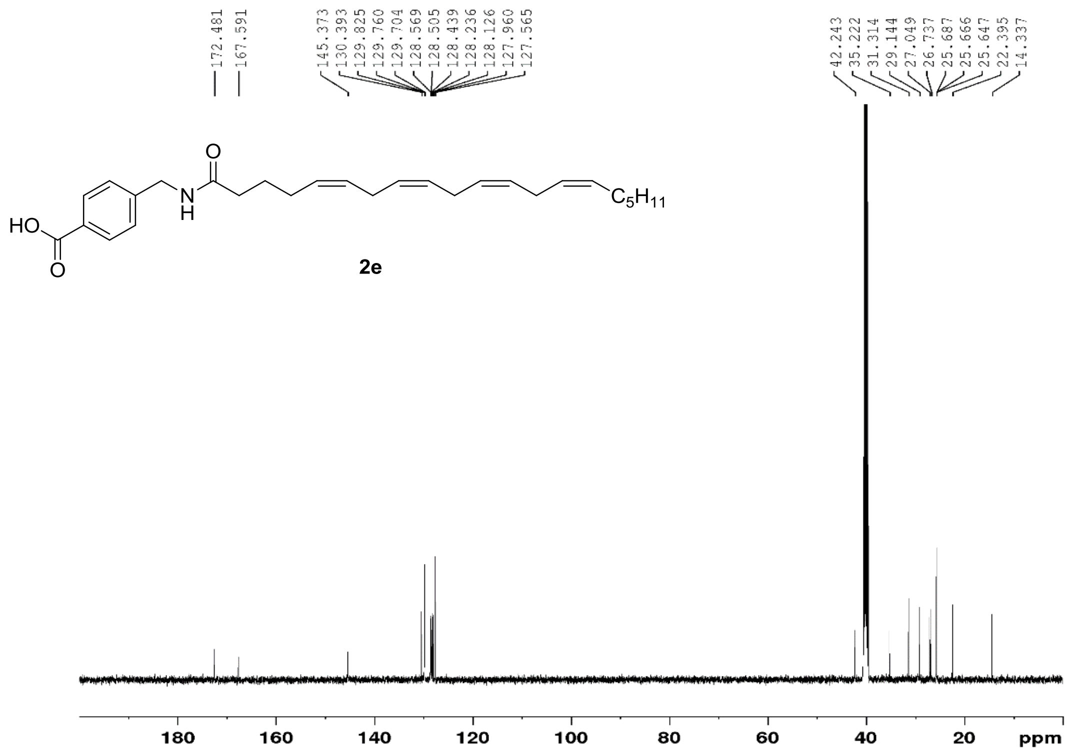

3-((5Z,8Z,11Z,14Z)-icosa-5,8,11,14-tetraenamidomethyl)benzoic acid (2f): Pale yellow solid, 98% yield, eluent system: 2:1:0.03 hexane-ethyl acetate-AcOH. ^{1}H NMR (500 MHz, $CDCl_3$) δ 8.01 (d, *J* = 8.1 Hz, 2H), 7.53 (d, *J* = 7.6 Hz, 1H), 7.43 (t, *J* = 7.7 Hz, 1H), 5.93 (s, 1H), 5.39 – 5.33 (m, 8H), 4.51 (d, *J* = 5.8 Hz, 2H), 2.83 – 2.79 (m, 6H), 2.28 – 2.25 (m, 2H), 2.12 (dd, *J* = 13.3, 6.8 Hz, 2H), 2.06 – 2.02 (m, 2H), 1.78 – 1.75 (m, 2H), 1.35 – 1.33 (m, 2H), 1.29 – 1.25 (m, 4H), 0.88 (t, *J* = 6.9 Hz, 3H); ^{13}C NMR (125 MHz, $CDCl_3$) δ 173.21, 170.77, 138.83, 133.02, 130.50, 129.94, 129.27, 129.22, 128.98, 128.90, 128.84, 128.58, 128.23, 128.11, 127.83, 127.49, 43.18, 35.98, 31.48, 29.28, 27.18, 26.64, 25.60(3C), 25.46, 22.53, 14.03.HRMS (ESI): calculated for $C_{28}H_{38}NO_3$ (M-1) 436.2587, found 436.2854.

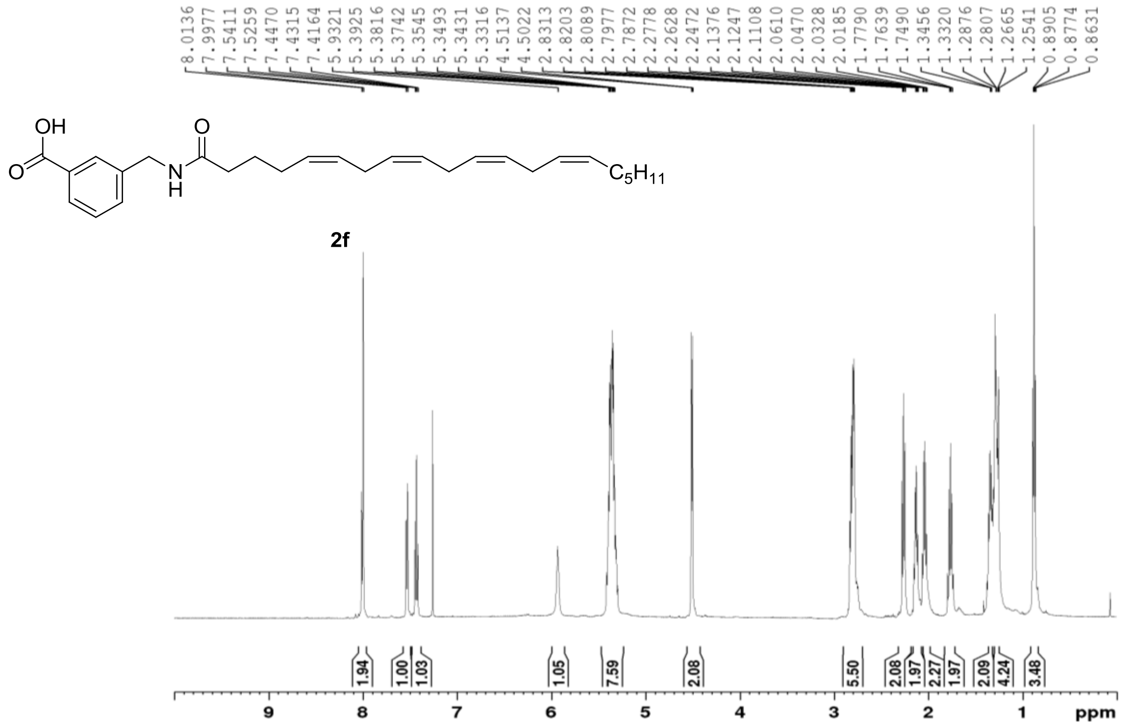

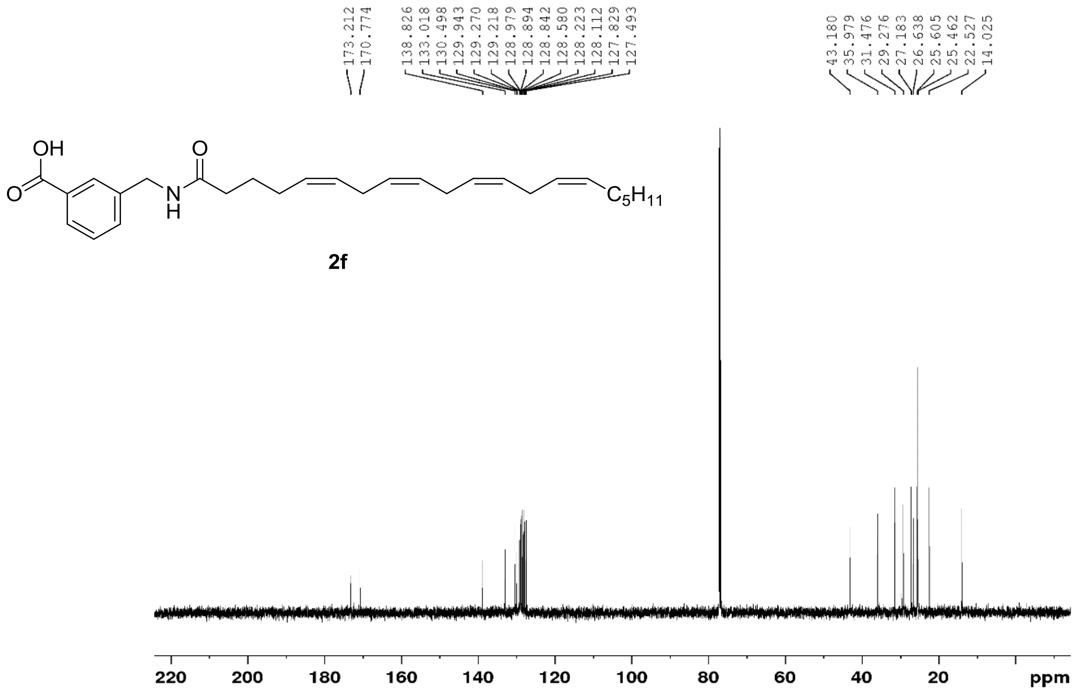

5-((6Z,9Z,12Z,15Z)-henicosa-6,9,12,15-tetraenamido)pentanoic acid (2g): pale yellow solid, 95% yield, eluent system: 2:1:0.03 hexane-ethyl acetate-AcOH. ^{1}H NMR (500 MHz, DMSO) δ 11.95 (s, 1H), 7.70 (s, 1H), 5.28 (s, 8H), 2.98 – 2.96 (m, 2H), 2.77 – 2.69 (m, 4H), 2.15 (t, *J* = 7.2 Hz, 2H), 1.99 – 1.98 (m, 8H), 1.45 – 1.42 (m, 4H), 1.35 – 1.32 (m, 2H), 1.29 – 1.24 (m, 2H), 1.22 (s, 6H), 0.81 (t, *J* = 6.4 Hz, 3H); ^{13}C NMR (125 MHz, DMSO) δ 174.33, 171.74, 129.90, 129.73, 129.48, 128.07, 128.02, 127.72, 127.66, 127.49, 40.41, 37.98, 35.27, 33.28, 30.89, 28.72, 28.700, 28.68, 26.61, 26.46, 25.20(2C), 24.99, 21.97, 21.93, 13.89. HRMS (ESI): calculated for $C_{26}H_{42}NO_3$ (M-1) 416.3170, found 416.3165.

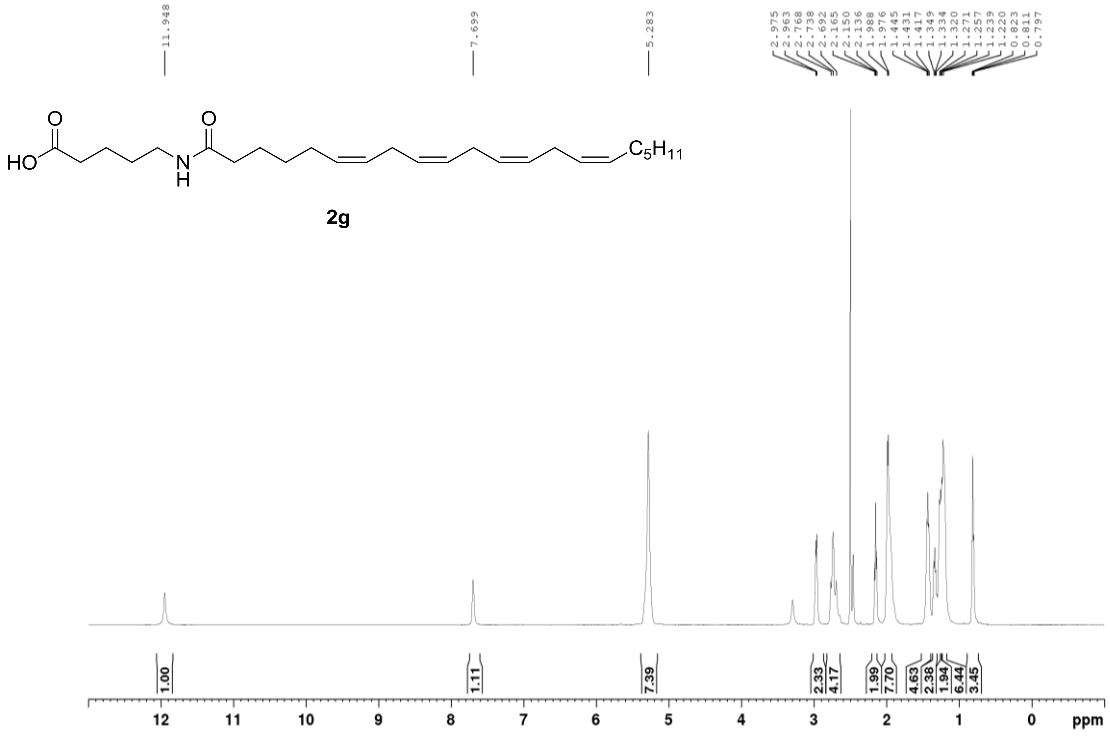

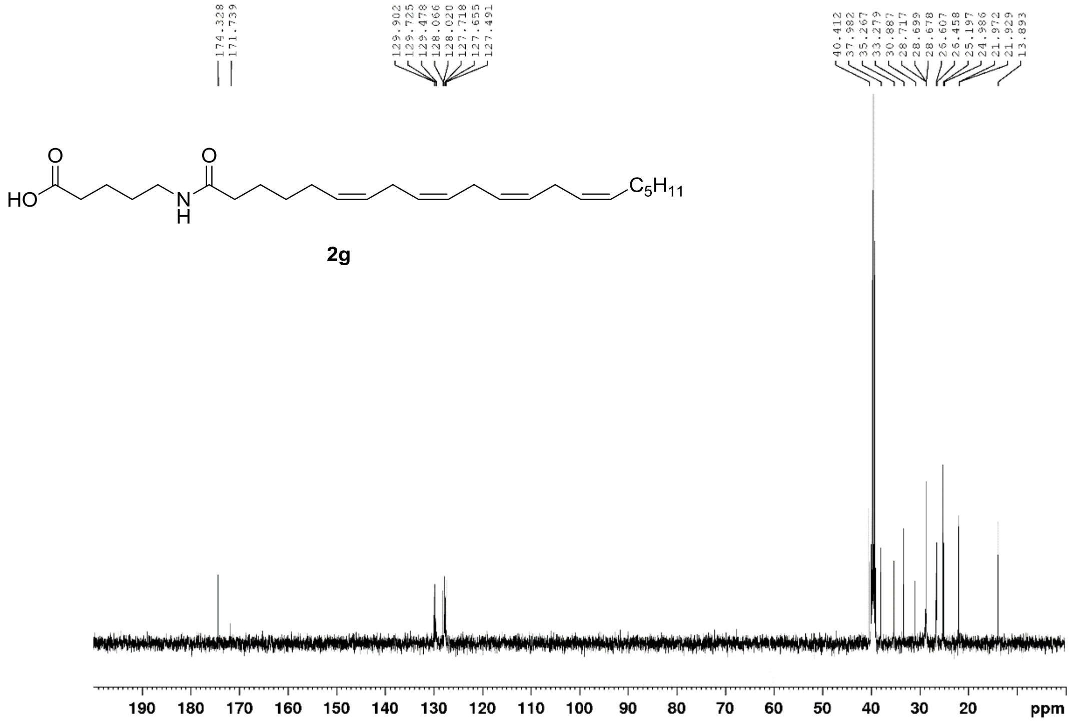

5-((6Z,9Z,12Z,15Z)-nonadeca-6,9,12,15-tetraenamido)pentanoic acid (**2h**): Pale yellow solid, 95% yield, eluent system: 2:1:0.03 hexane-ethyl acetate-AcOH. ^{1}H NMR (500 MHz, DMSO) δ 11.99 (s, 1H), 7.74 (m, 1H), 5.38 – 5.33 (m, 8H), 3.02 (q, *J* = 6.5 Hz, 2H), 2.83 – 2.78 (m, 4H), 2.20 (t, *J* = 7.3 Hz, 2H), 2.06 – 2.00 (m, 6H), 1.51 – 1.47 (m, 4H), 1.40 – 1.35 (m, 2H), 1.34 – 1.29 (m, 2H), 1.27 –1.25 (s, 2H), 0.88 (t, *J* = 7.3 Hz, 3H); ^{13}C NMR (125 MHz, DMSO) δ 174.77, 172.21, 130.20, 130.13, 128.54, 128.50, 128.19(2C), 128.15, 128.08, 38.43, 35.73, 33.75, 29.15, 29.13, 26.90, 25.65, 25.43, 22.65, 22.38, 14.01.HRMS (ESI): calculated for $C_{24}H_{38}NO_3$ (M-1) 388.2857, found 388.2866.

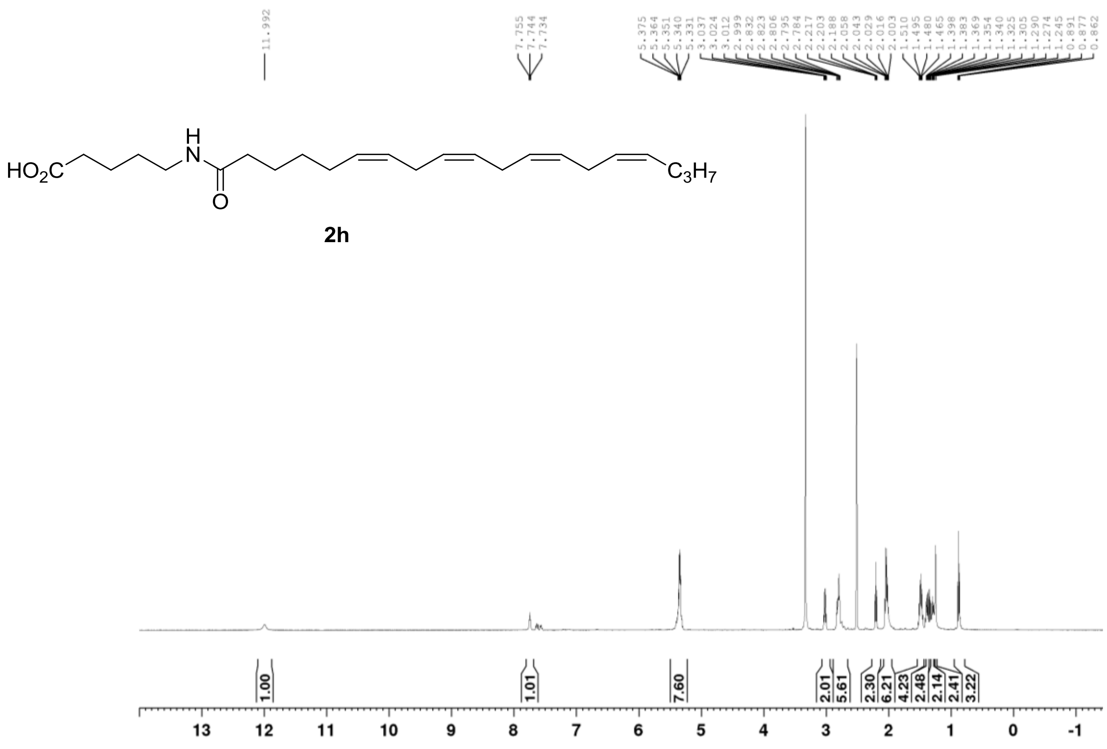

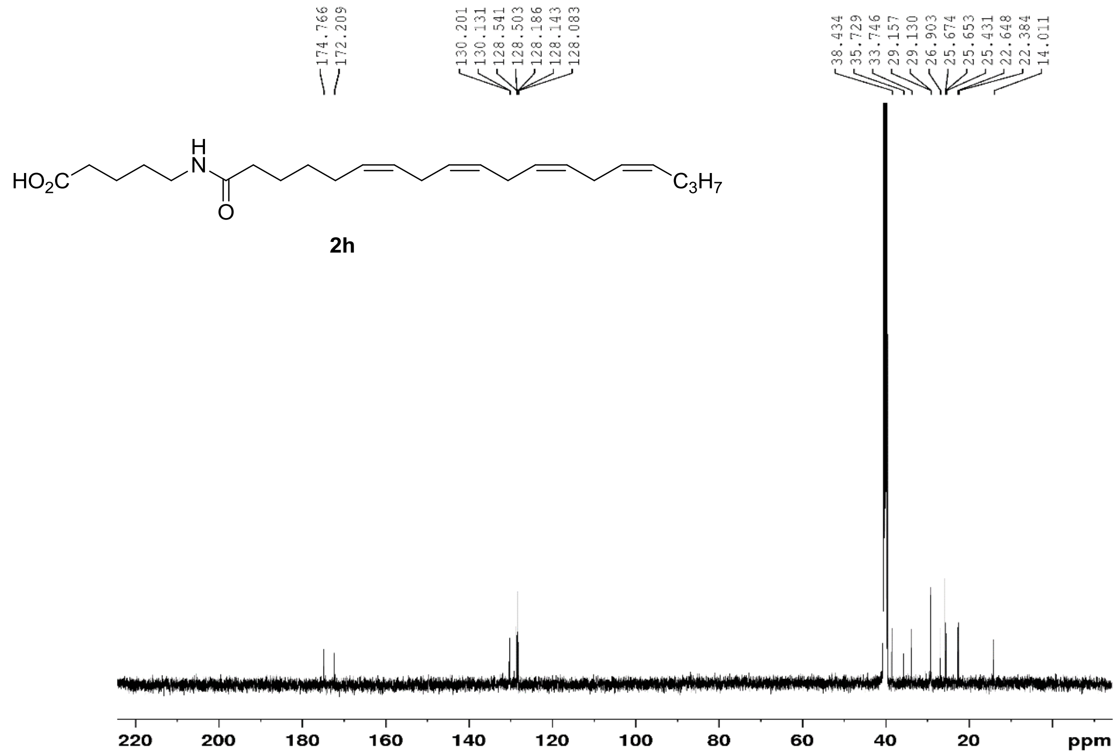

3-((6Z,9Z,12Z,15Z)-henicosa-6,9,12,15-tetraenamido)benzoic acid (2i): Pale yellow solid, 81 % yield, eluent system: 2:1:0.03 hexane-ethyl acetate-AcOH. ^{1}H NMR (500 MHz, DMSO) δ 12.86 (s, 1H), 10.01 (s, 1H), 8.21 (s, 1H), 7.82 (d, *J* = 8.1 Hz, 1H), 7.59 (d, *J* = 7.7 Hz, 1H), 7.39 (t, *J* = 7.9 Hz, 1H), 5.37 – 5.29 (m, 8H), 2.79 (dt, *J* = 17.2, 5.2 Hz, 6H), 2.32 (t, *J* = 7.4 Hz, 2H), 2.07 (dd, *J* = 14.1, 7.0 Hz, 2H), 2.01 (dd, *J* = 13.8, 6.8 Hz, 2H), 1.61 (dt, *J* = 15.1, 7.5 Hz, 2H), 1.37 (dt, *J* = 15.0, 7.6 Hz, 2H), 1.30 – 1.23 (m, 6H), 0.84 (t, *J* = 6.9 Hz, 3H); ^{13}C NMR (125 MHz, DMSO) δ 171.27, 167.08, 139.43, 131.18, 129.85, 129.59, 128.73, 128.00, 127.93, 127.68, 127.65, 127.59, 127.41, 123.63, 122.99, 119.73, 36.19, 30.77, 28.59(2C), 26.51, 26.40, 25.14, 25.13(2C), 24.63, 21.84, 13.78. HRMS (ESI): calculated for $C_{28}H_{38}NO_3$ (M-1) 436.2587, found 436.2866.

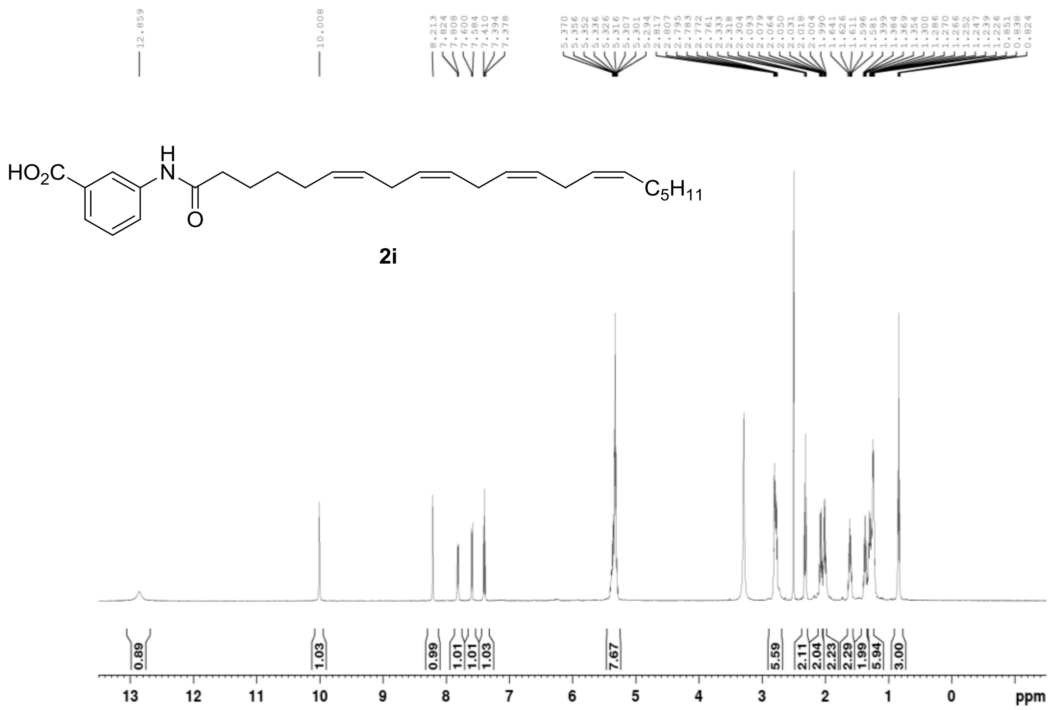

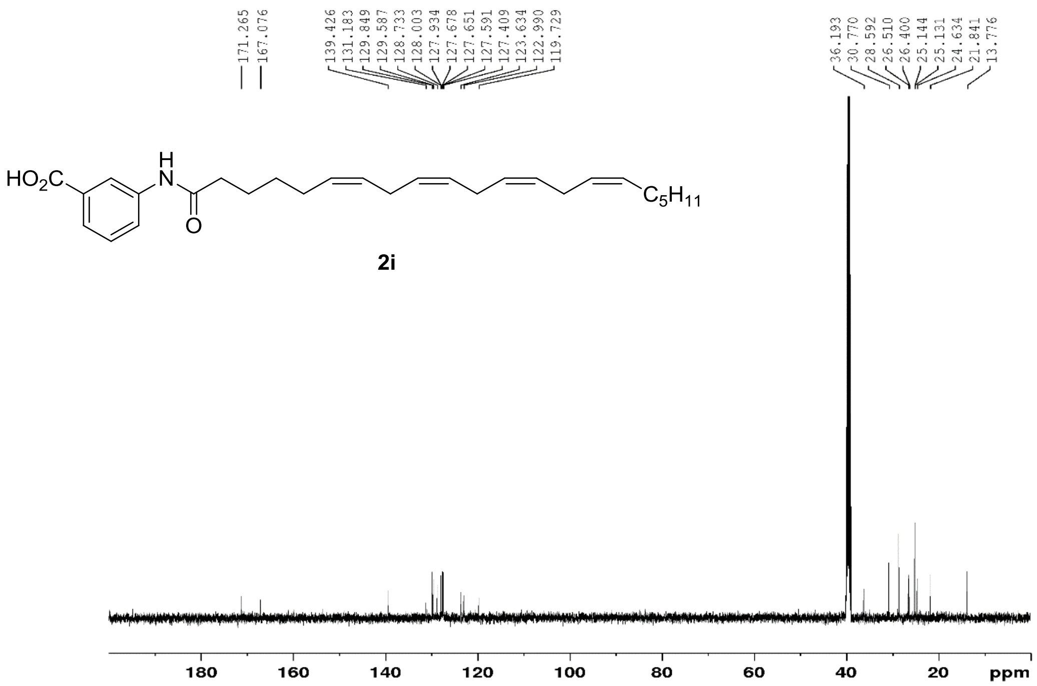

2i

3-((6Z,9Z,12Z,15Z)-nonadeca-6,9,12,15-tetraenamido)benzoic acid (2j): Pale yellow solid, 98% yield, eluent system: 2:1:0.03 hexane-ethyl acetate-AcOH. ^{1}H NMR (500 MHz, DMSO) δ 12.87 (s, 1H), 10.02 (s, 1H), 8.22 (s, 1H), 7.82 (d, *J* = 7.6 Hz, 1H), 7.60 (d, *J* = 7.7 Hz, 1H), 7.40 (t, *J* = 7.9 Hz, 1H), 5.38 – 5.33 (m, 8H), 2.82 – 2.78 (m, 6H), 2.33 (t, *J* = 7.4 Hz, 2H), 2.10 – 2.06 (m, 2H), 2.04 (dd, *J* = 13.4, 6.9 Hz, 2H), 1.62 (dt, *J* = 15.2, 7.5 Hz, 2H), 1.39 – 1.35 (m, 2H), 1.32 (dd, *J* = 14.7, 7.3 Hz, 2H), 0.86 (d, *J* = 7.3 Hz, 3H); ^{13}C NMR (125 MHz, DMSO) δ 171.27, 167.11, 139.41, 131.35, 129.59, 128.71, 128.01, 127.95, 127.68, 127.66, 127.66, 127.64, 127.61, 123.64, 122.94, 119.74, 36.19, 28.61, 28.59, 26.40, 25.14 (2C), 24.63, 22.10, 13.45. HRMS (ESI): calculated for $C_{26}H_{34}NO_3$ (M-1) 408.2544, found 408.2543.

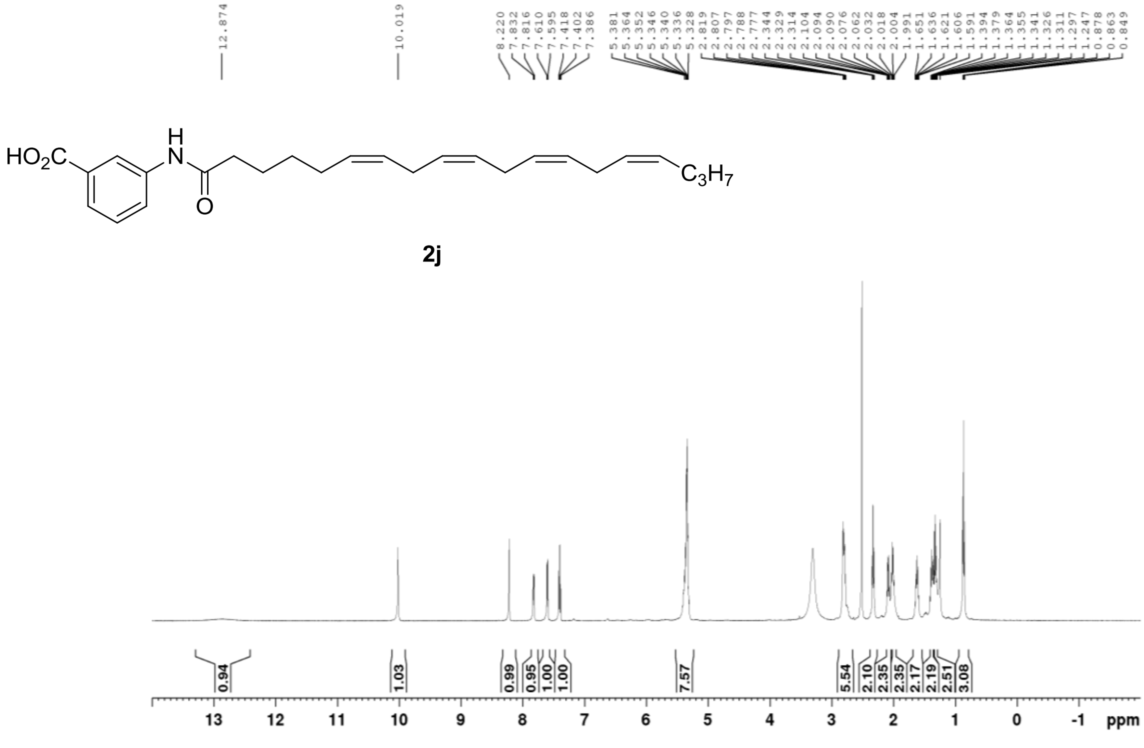

2j

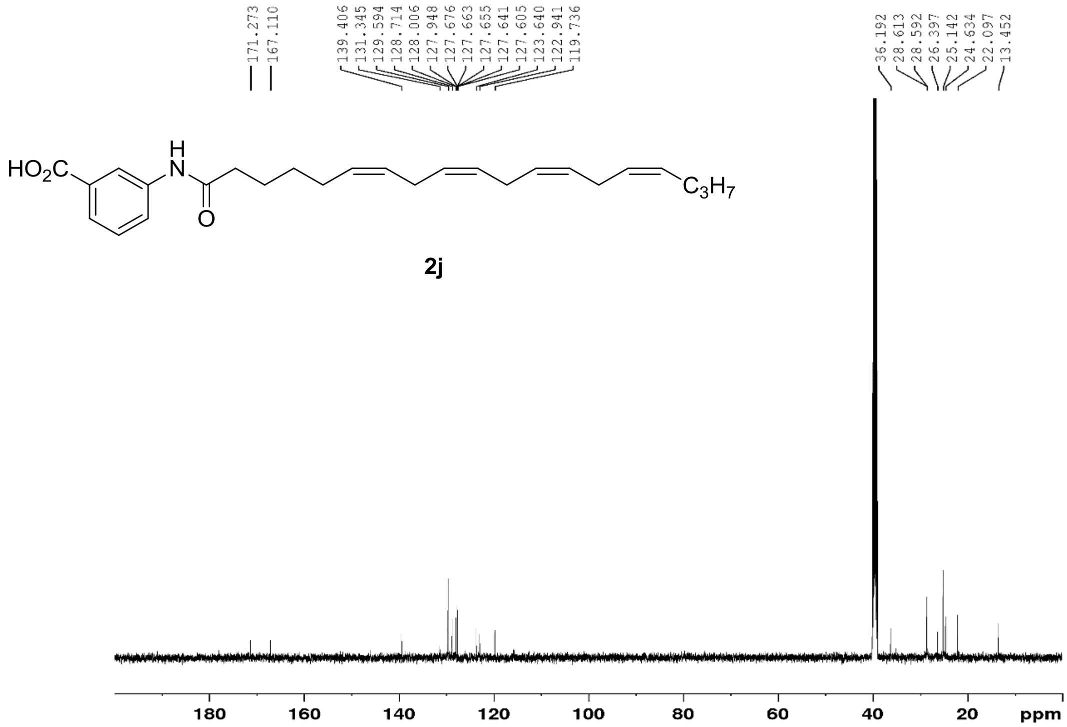

5-((7Z,10Z,13Z,16Z)-docosa-7,10,13,16-tetraenamido)pentanoic acid (2k): Pale yellow solid, 69% yield, eluent system: 2:1:0.03 hexane-ethyl acetate-AcOH. ^{1}H NMR (500 MHz, DMSO) δ 11.98 (s, 1H), 7.73 (s, 1H), 5.34 – 5.33 (m, 8H), 3.34(s, 6H), 3.02 (dd, *J* = 12.6, 6.5 Hz, 2H), 2.82 – 2.75 (m, 2H), 2.20 (t, *J* = 7.3 Hz, 2H), 2.04 – 2.02 (m, 4H), 1.50 – 1.47 (m, 4H), 1.40 – 1.36 (m, 2H), 1.33 – 1.25 (m, 10H), 0.86 (t, *J* = 6.7 Hz, 3H); ^{13}C NMR (125 MHz, DMSO) δ 174.76, 172.28, 130.37, 130.32, 128.52, 128.49, 128.16, 128.13, 127.99, 127.95, 38.42, 35.82, 33.74, 31.32, 29.45, 29.26, 29.23, 29.15, 29.12, 28.75, 27.06, 27.02, 27.00, 25.66, 25.064, 22.38, 14.34. HRMS (ESI): calculated for $C_{27}H_{44}NO_3$ (M-1) 430.3327, found 430.3323.

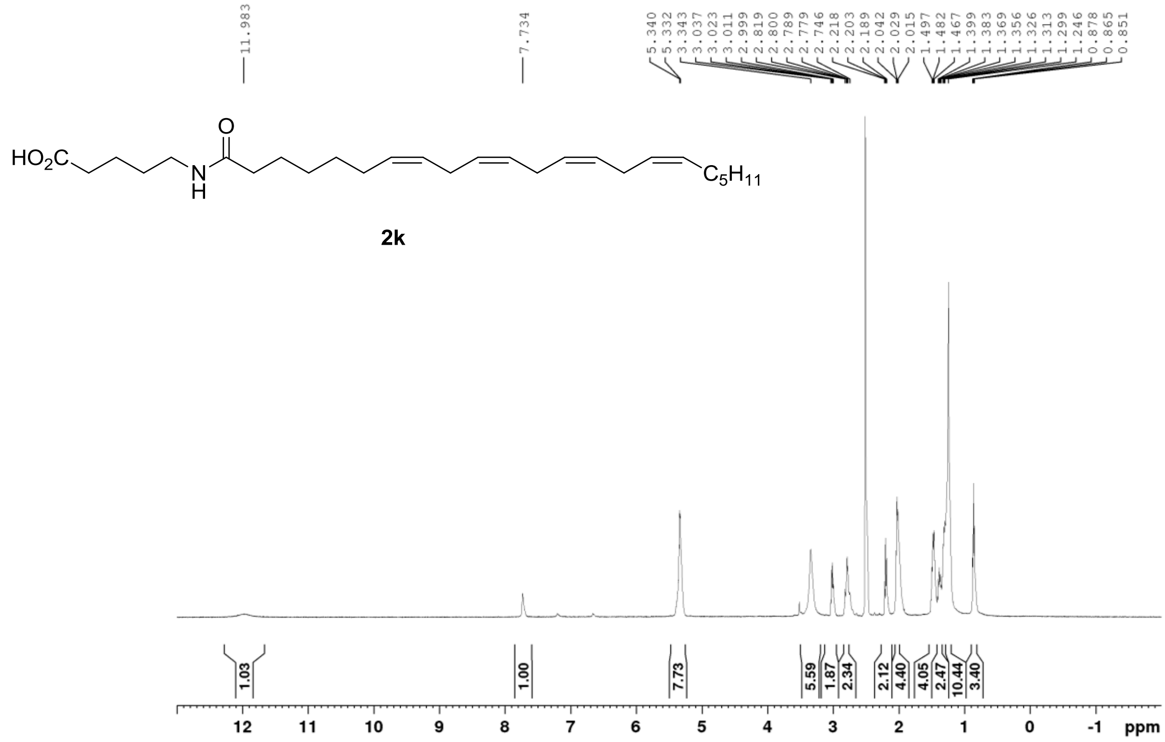

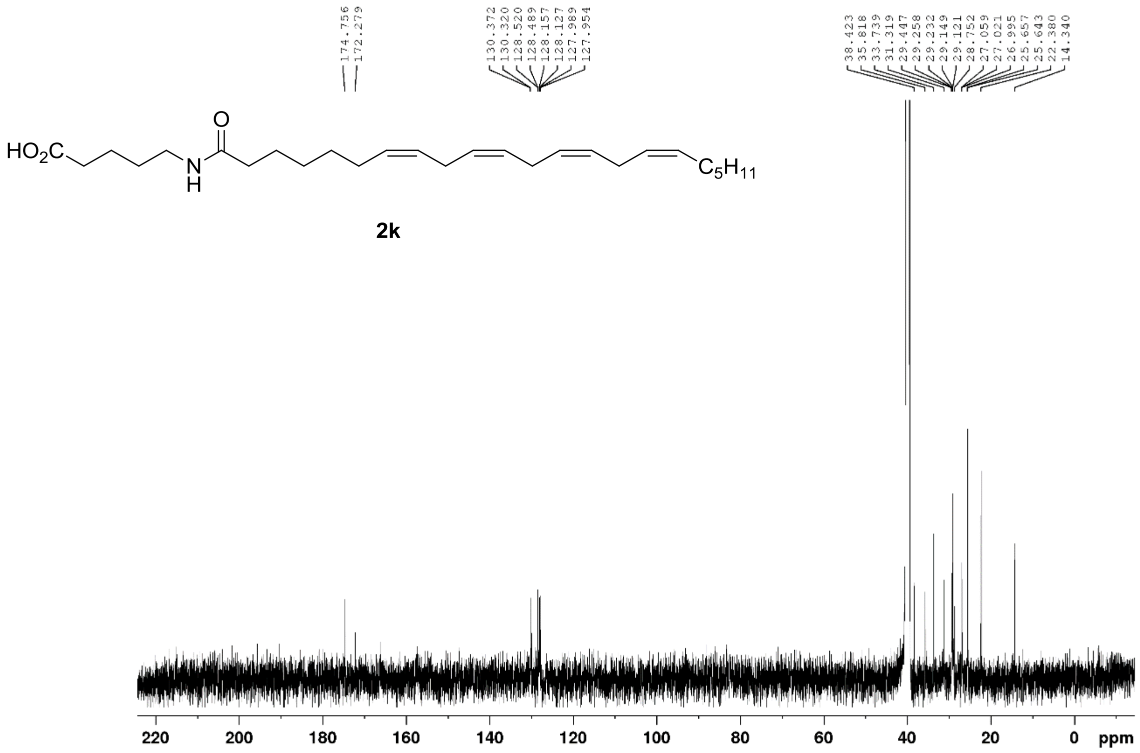

2k

4-((5Z,8Z,11Z,14Z)-icosa-5,8,11,14-tetraenamido)benzamide (2l): Pale yellow solid, 34% yield, eluent system: 1:3 hexane-ethyl acetate. ^{1}H NMR (500 MHz, MeOD) δ 7.86 (d, *J* = 8.8 Hz, 2 H), 7.70 (d, *J* = 8.8 Hz, 2 H), 5.60 – 5.22 (m, 8 H), 2.98 – 2.71 (m, 6 H), 2.44 (t, *J* = 7.4 Hz, 2 H), 2.20 (dd, *J* = 13.2, 7.3 Hz, 2 H), 2.07 (dd, *J* = 13.7, 6.6 Hz, 2 H), 1.80 (dt, *J* = 14.8, 7.4 Hz, 2 H), 1.55 – 1.20 (m, 8 H), 0.91 (t, *J* = 7.0 Hz, 3 H); ^{13}C NMR (125 MHz, $CDCl_3$) δ 173.22, 170.39, 142.07, 129.80, 128.72, 128.51, 128.37, 128.24 (2 C), 128.04, 127.76, 127.74 (2 C), 127.46, 127.35, 118.82, 35.96, 31.24, 29.04, 26.77, 26.25, 25.19, 25.16, 25.15 (2 C), 22.21, 13.02; HRMS (ESI): calculated for $C_{27}H_{37}N_2O_2$ (M-1) 421.2861, found 421.2873.

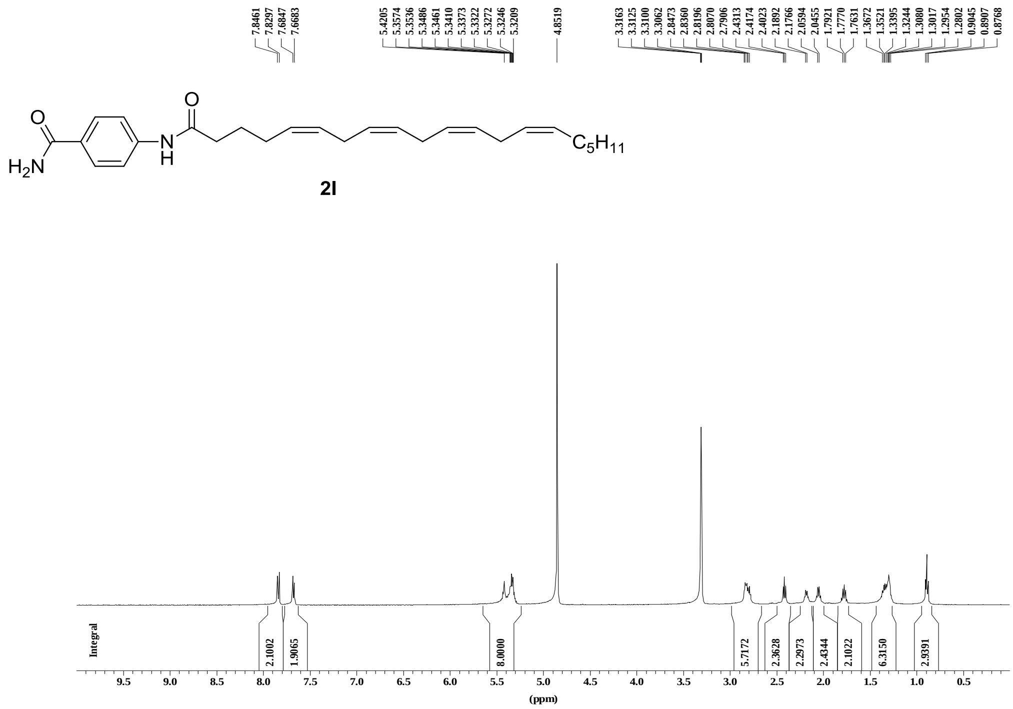

2l

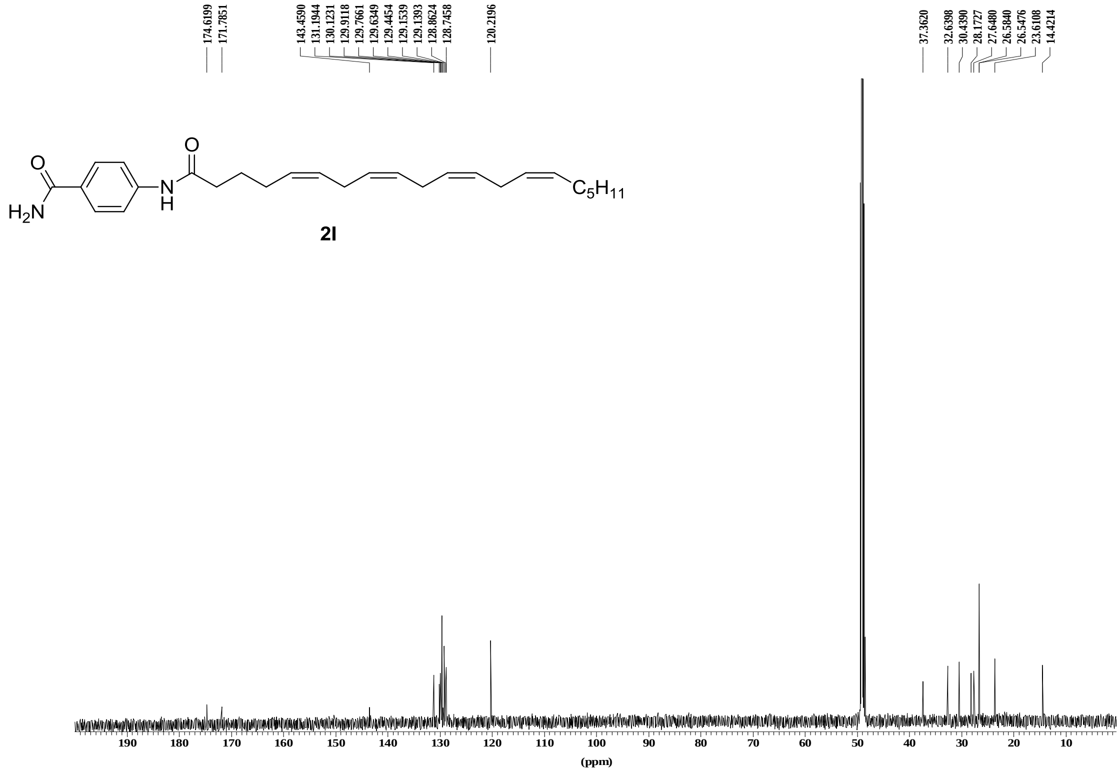

3-((5Z,8Z,11Z,14Z)-icosa-5,8,11,14-tetraenamido)benzamide (2m): white solid, 74% yield (over 2 steps), eluent system: 1:3 hexane-ethyl acetate. ^{1}H NMR (300 MHz, DMSO) δ 8.04 (s, 1H), 7.76 (dd, *J* = 8.1, 1.0 Hz, 1H), 7.57 (d, *J* = 7.8 Hz, 1H), 7.38 (t, *J* = 7.9 Hz, 1H), 5.42 – 5.38 (m, 2H), 5.37 – 5.31 (m, 5H), 2.85 – 2.77 (m, 4H), 2.41 (t, *J* = 7.5, 2H), 2.19 – 2.15 (m, 2H), 2.06 – 2.02 (m, 3H), 1.80 – 1.75 (m, 2H), 1.37 – 1.30 (m, 6H), 0.89 (t, *J* = 6.8 Hz, 3H); ^{13}C NMR (75 MHz, DMSO) δ 174.45, 172.11, 140.23, 135.76, 131.17, 130.12, 129.96, 129.88, 129.43, 129.15, 129.13, 128.86, 128.74, 124.37, 123.95, 120.38, 37.29, 32.65, 30.46, 28.18, 27.68, 26.65, 26.58, 26.54(2C), 23.63, 14.46. HRMS (ESI): calculated for $C_{27}H_{37}N_2O_2$ (M-1) 421.2861, found 421.2842.

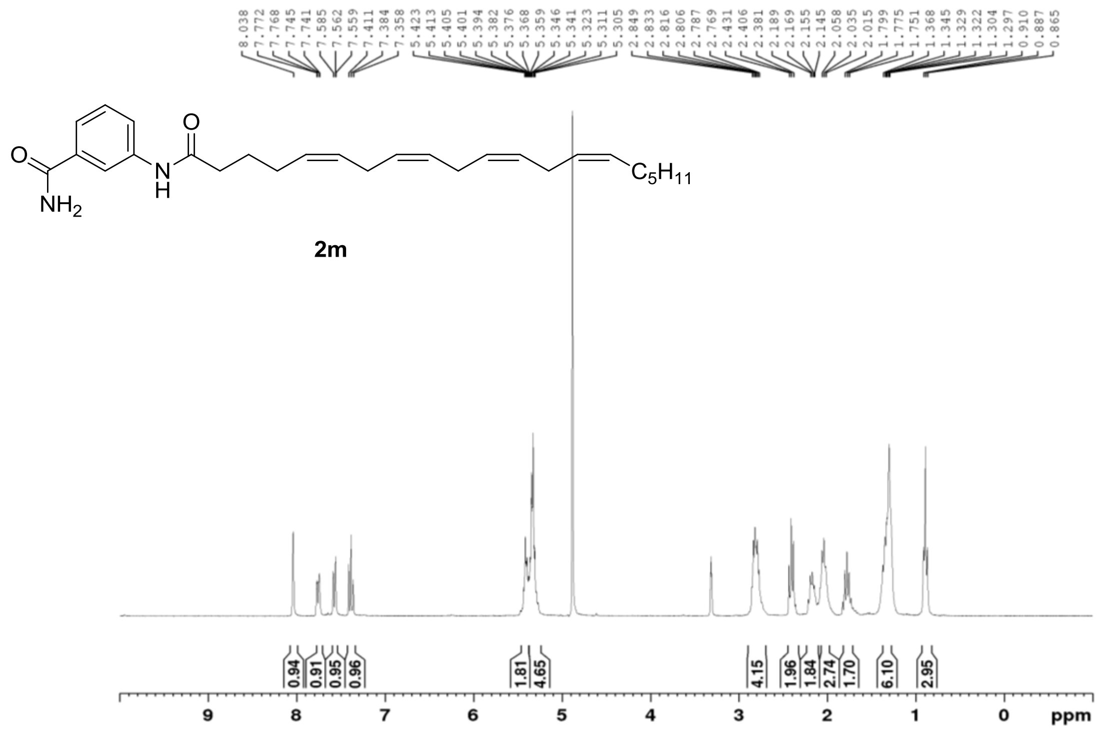

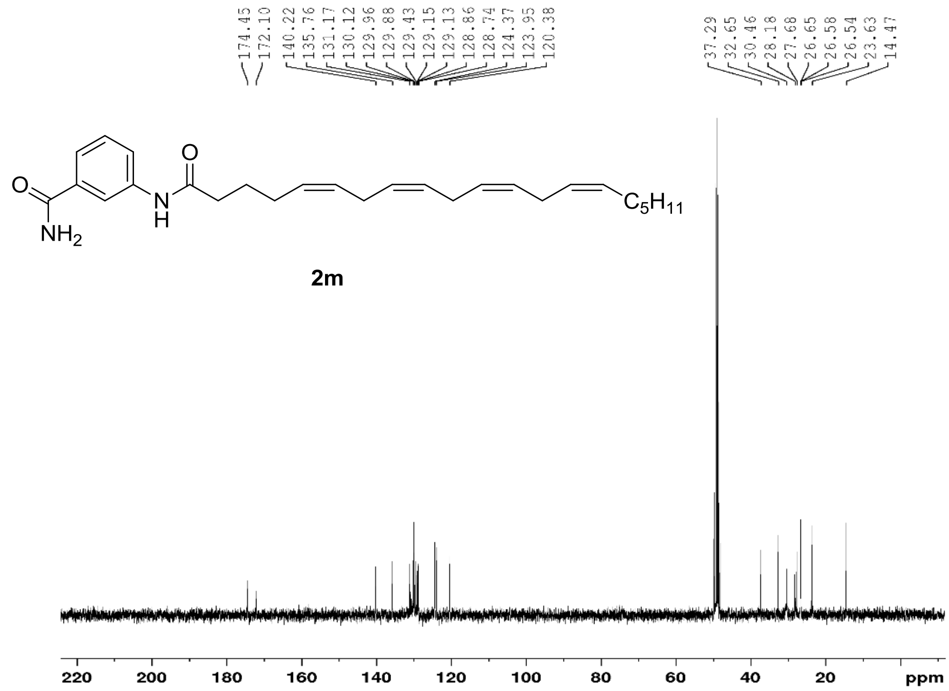

4-((5Z,8Z,11Z,14Z)-icosa-5,8,11,14-tetraenamido)benzoic acid (2n): Pale yellow solid, 68% yield, eluent system: 2:1:0.03 hexane-ethyl acetate-AcOH. ^{1}H NMR (500 MHz, DMSO) δ 12.65 (s, 1H), 10.18 (s, 1H), 7.86 (d, *J* = 8.6 Hz, 2H), 7.70 (d, *J* = 8.3 Hz, 2H), 5.37 – 5.31 (m, 7H), 2.80 – 2.76 (m, 5H), 2.35 (t, *J* = 7.1 Hz, 2H), 2.10 – 2.08 (m, 2H), 2.01 – 1.98 (m, 3H), 1.67 – 1.64 (m, 2H), 1.30 – 1.26 (m, 2H), 1.24 – 1.23 (m, 4H), 0.83 (t, *J* = 6.6 Hz, 3H); ^{13}C NMR (125 MHz, DMSO) δ 171.54, 166.91, 143.32, 130.30(2C), 129.91, 129.26, 128.23, 128.07, 127.95, 127.77, 127.63, 127.47, 124.81, 118.17(2C), 35.80, 30.85, 28.68, 26.57, 26.16, 25.18(3C), 24.81, 21.94, 13.88. HRMS (ESI): calculated for $C_{27}H_{36}NO_3$ (M-1) 422.2701, found 422.2690.

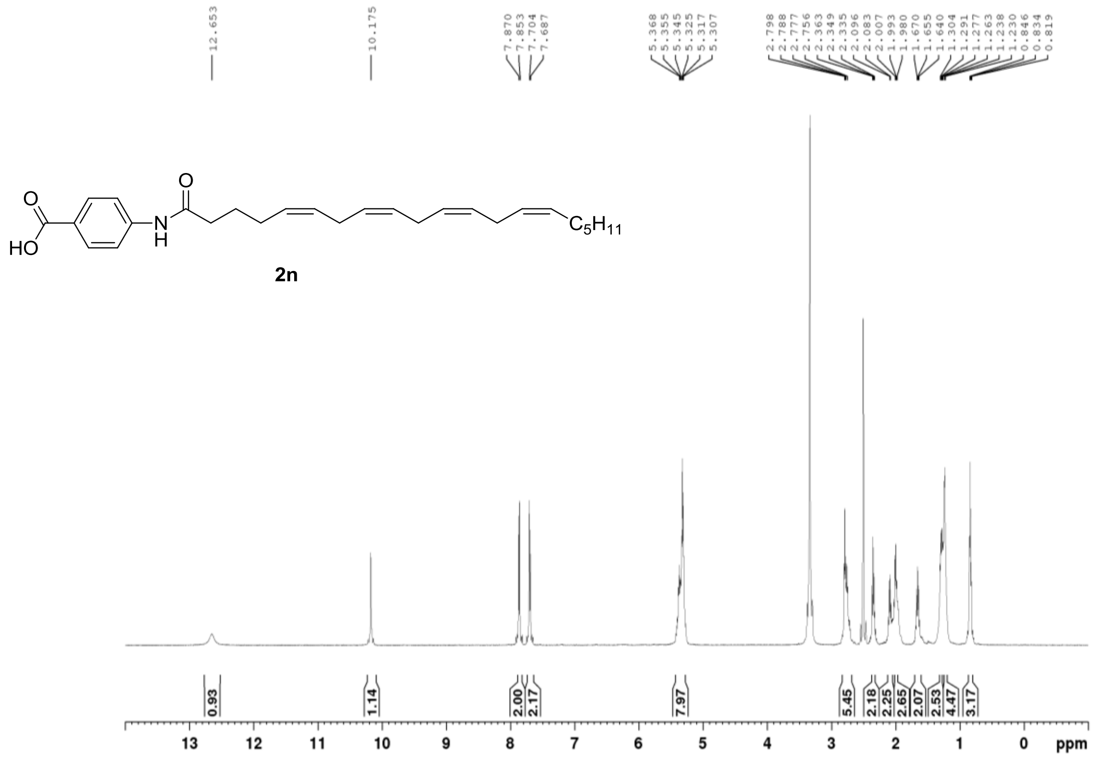

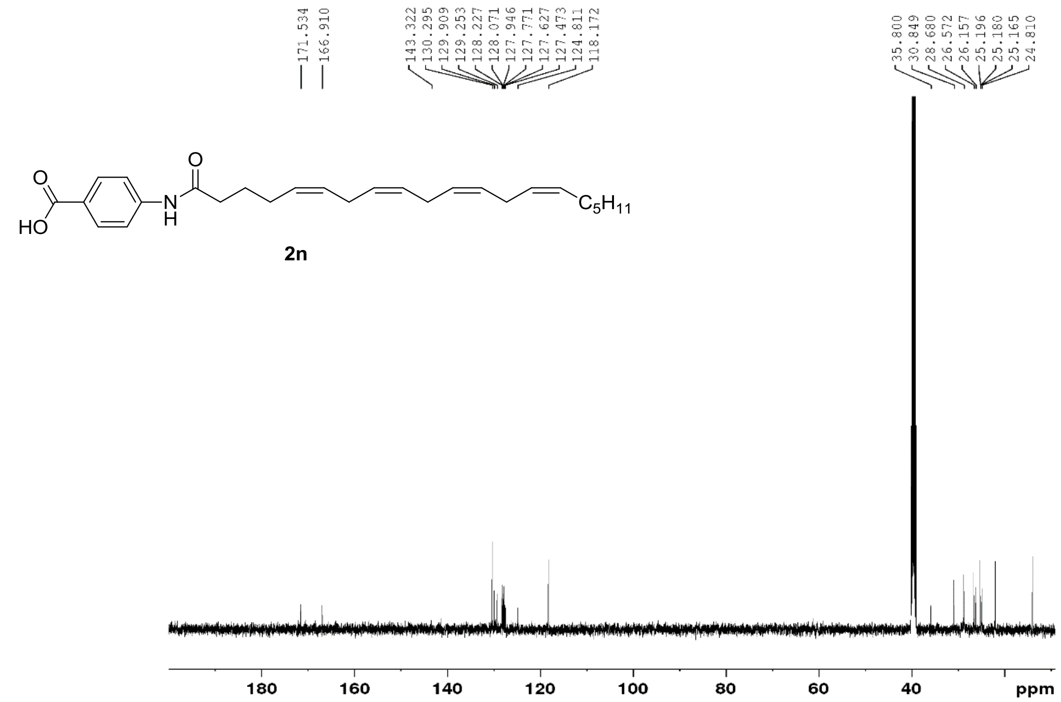

2-((5Z,8Z,11Z,14Z)-icosa-5,8,11,14-tetraenamido)benzoic acid (2o): Pale yellow solid, 96% yield, eluent system: 2:1:0.03 hexane-ethyl acetate-AcOH. ^{1}H NMR (300 MHz, $CDCl_3$) δ 10.93 (s, 1H), 8.76 (d, *J* = 8.5 Hz, 1H), 8.11 (dd, *J* = 8.0, 1.5 Hz, 1H), 7.62 – 7.57 (m, 1H), 7.14 – 7.09 (m, 1H), 5.44 – 5.33 (m, 8H), 2.84 – 2.77 (m, 6H), 2.47 (t, *J* = 7.8 Hz, 2H), 2.20 – 2.06 (m, 2H), 2.05 – 2.01(m, 2H), 1.85 (t, *J* = 7.4 Hz, 2H), 1.37 – 1.25 (m, 6H), 0.88 (t, *J* = 6.7 Hz, 3H); ^{13}C NMR (75 MHz, $CDCl_3$) δ 172.13, 171.73, 142.11, 135.61, 131.72, 130.49, 128.97, 128.93, 128.56, 128.21, 128.14, 127.85, 127.53, 122.56, 120.49, 113.74, 37.95, 31.49, 29.30, 27.19, 26.57, 25.64, 25.61(2C), 25.25, 22.55, 14.05. HRMS (ESI): calculated for $C_{27}H_{36}NO_3$ (M-1) 422.2701, found 422.2707.

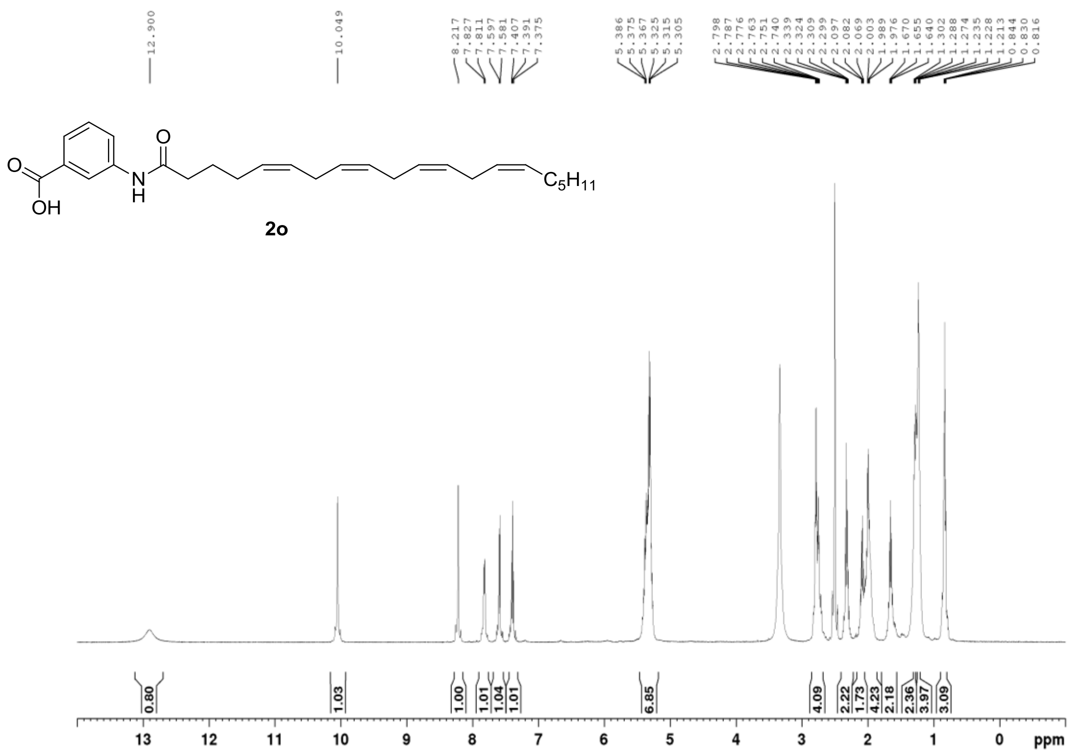

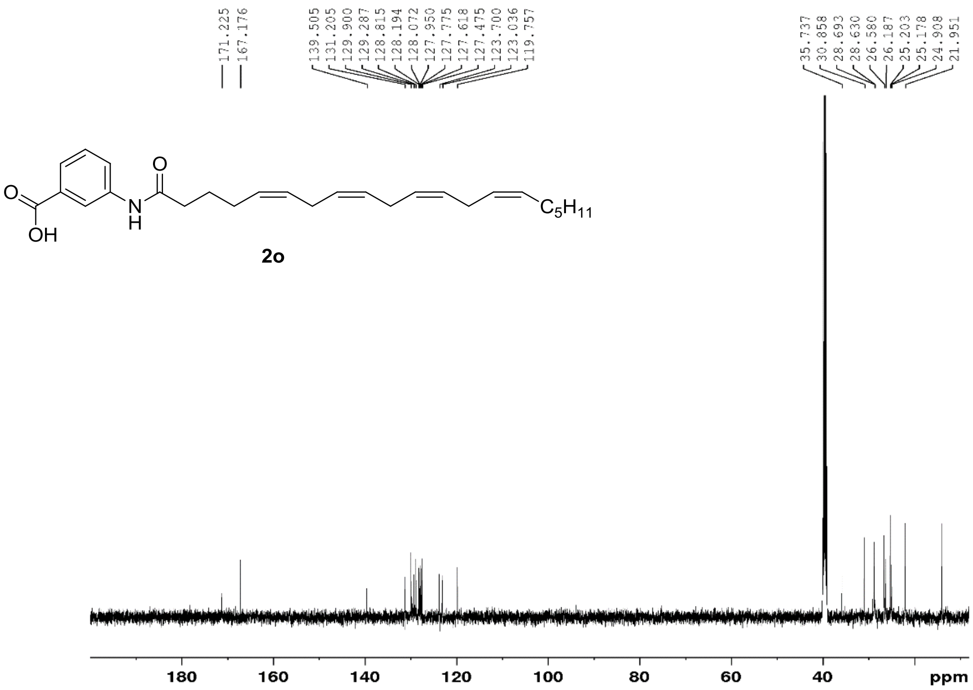

Synthesis of (5Z,8Z,11Z,14Z)-N-(3-sulfamoylphenyl)icosa-5,8,11,14-tetraenamide (2p): EDC.HCl (13 mg, 0.07 mmol), **19a** (18 mg, 0.06 mmol), TEA (9 µL, 0.07 mmol) and 3-aminobenzenesulfonamide (11 mg, 0.07 mmol) were dissolved in MeCN (1.5 mL) and stirred overnight at 4°C. The reaction was quenched with ethyl acetate and the mixture was washed with aqueous $NaHCO_3$ (3 x 20 mL) and brine (3 x 20 mL). The organic layer was dried, concentrated and purified by flash column chromatography using a 1:1 hexane-ethyl acetate eluent system to yield **2p** as a pale yellow solid (13 mg, 47% yield). ^{1}H NMR (500 MHz, DMSO) δ 10.18 (s,

1H), 8.17 (s, 1H), 7.76 – 7.74 (m, 1H), 7.48 – 7.46 (m, 2H), 7.34 (s, 2H), 5.40 – 5.31 (m, 8H), 2.82 – 2.77 (m, 6H), 2.35 (t, *J* = 7.4 Hz, 2H), 2.11 (dd, *J* = 13.9, 6.9 Hz, 2H), 2.03 – 2.00 (m, 2H), 1.70 – 1.64 (m, 2H), 1.32 – 1.29 (m, 2H), 1.28 – 1.24 (m, 4H), 0.85 (t, *J* = 6.2 Hz, 3H); ^{13}C NMR (125 MHz, DMSO) δ 171.85, 145.02, 140.05, 130.39(2C), 129.74, 128.67, 128.57, 128.41, 128.29, 128.10, 127.95, 122.24, 120.46, 116.47, 36.24, 31.31, 29.42, 29.13, 27.04, 26.66, 25.66(2C), 25.38, 22.38, 14.33. HRMS (ESI): calculated for $C_{26}H_{37}NO_3S$ (M-1) 457.2530, found 457.2537

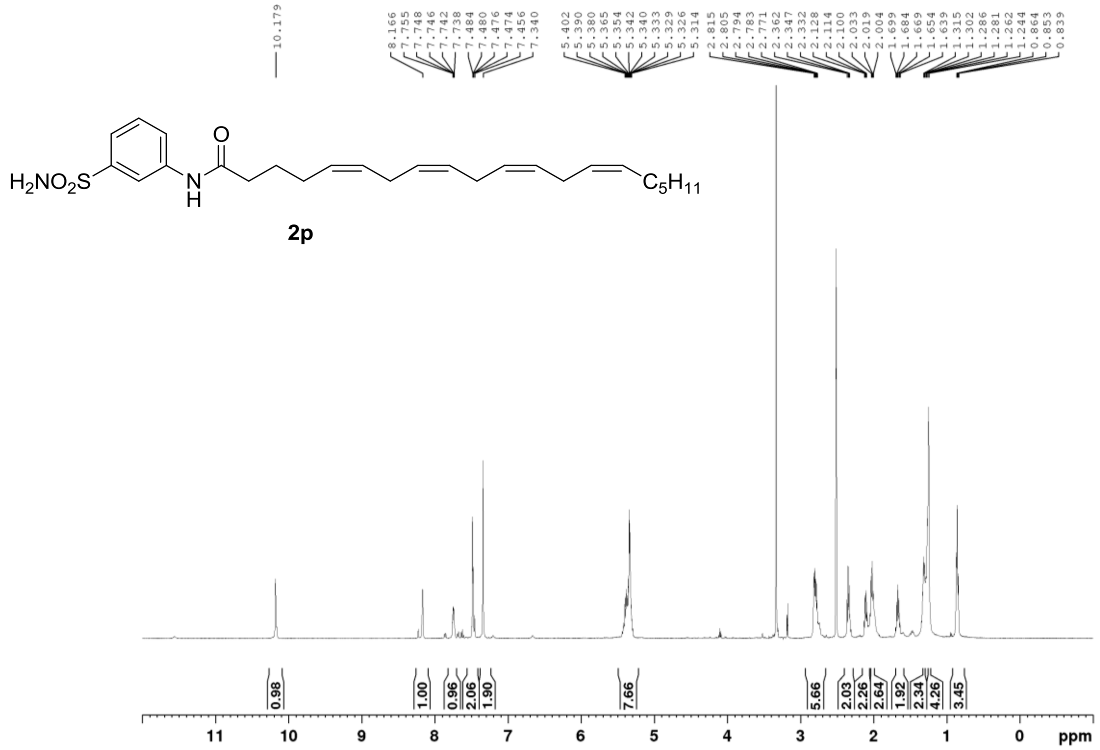

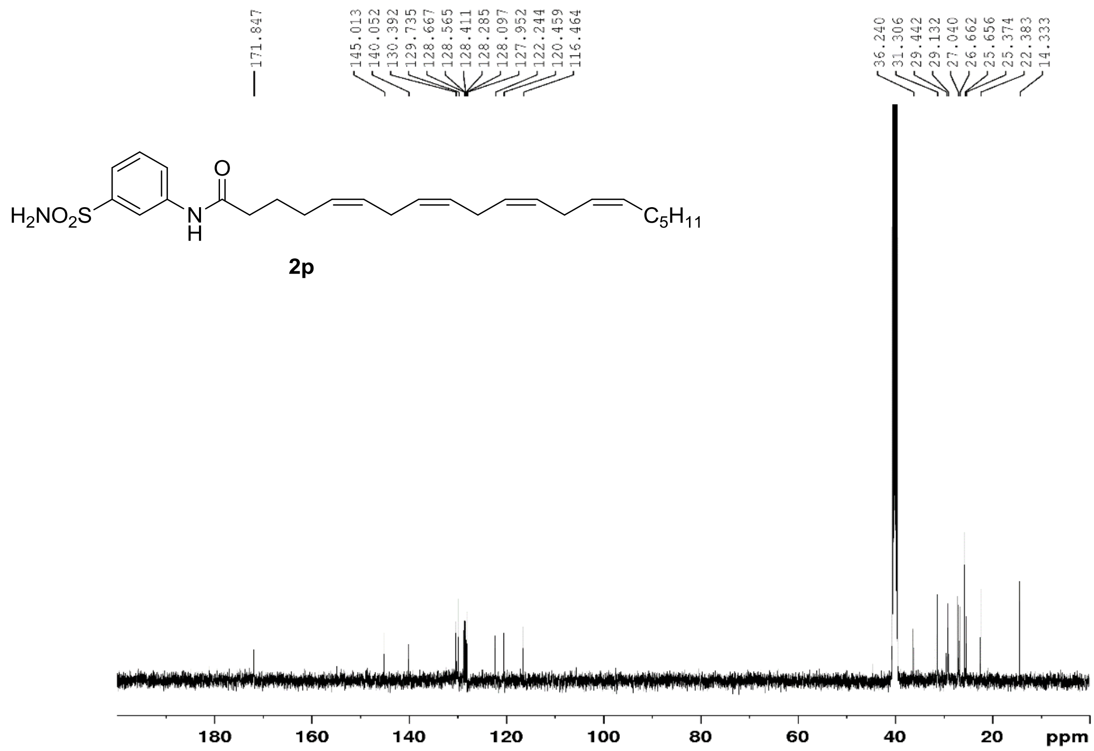

2p

5-((4Z,7Z,10Z,13Z)-nonadeca-4,7,10,13-tetraenamido)pentanoic acid (2q): Pale yellow solid, 64% yield, eluent system: 2:1:0.03 hexane-ethyl acetate-AcOH. ^{1}H NMR (500 MHz, DMSO) δ 11.99 (s, 1H), 7.78 – 7.74 (m, 1H), 5.36 – 5.31 (m, 7H), 3.04 – 3.00 (m, 2H), 2.83 – 2.75 (m, 4H), 2.20 (t, *J* = 7.3 Hz, 2H), 2.08 (dd, *J* = 8.9, 6.1 Hz, 2H), 2.04 – 2.00 (m, 4H), 1.47 (d, *J* = 7.3 Hz, 2H), 1.40 – 1.38 (m, 2H), 1.33 – 1.30 (m, 2H), 1.27 – 1.25 (m, 6H), 0.86 (t, *J* = 6.9 Hz, 3H); ^{13}C NMR (125 MHz, DMSO) δ 174.32, 171.16, 129.92, 128.86, 128.08, 128.03, 128.00, 127.75, 127.67, 127.50, 38.02, 35.21, 33.27, 30.84, 28.68, 28.62, 26.57, 25.18, 25.17, 25.13, 23.16, 21.93, 21.91, 13.88. HRMS (ESI): calculated for $C_{24}H_{38}NO_3$ (M-1) 388.2857, found 388.2841.

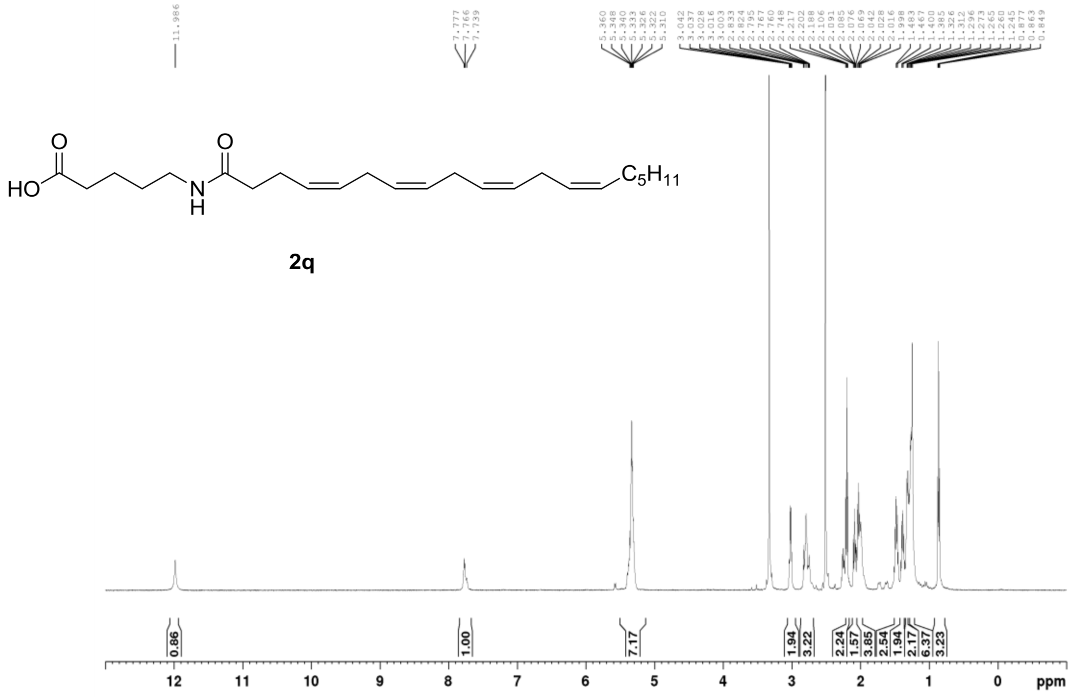

2q

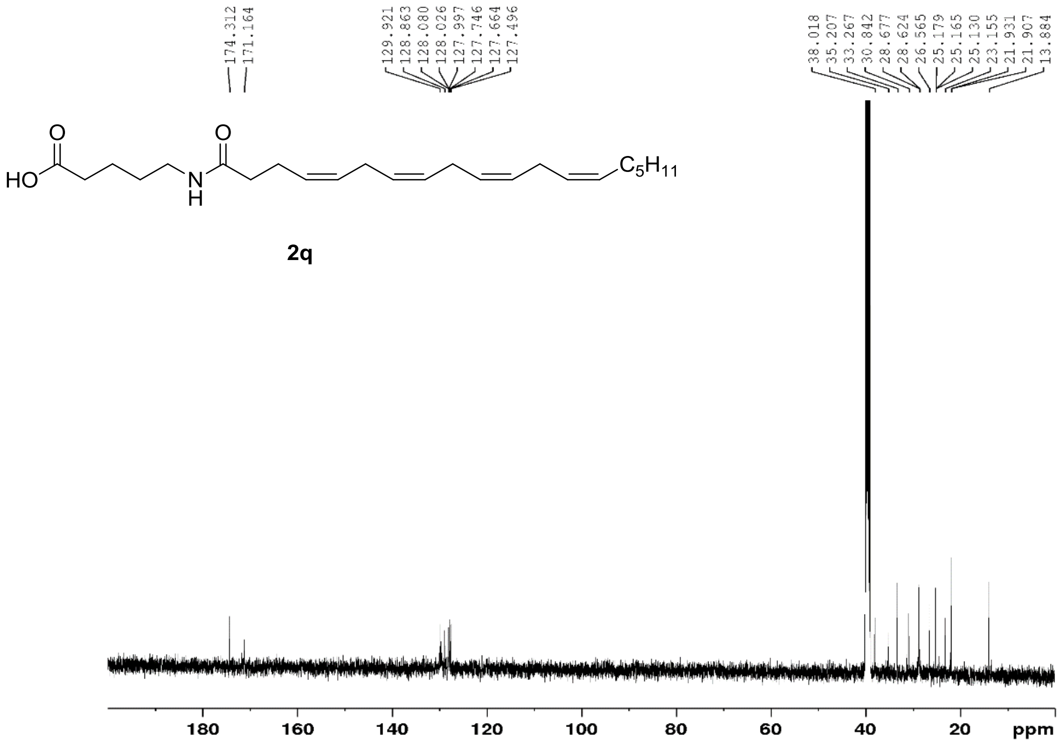

2q

Undeca-2,5-diyn-1-ol (4a): Yellow oil, 88% yield, eluent system: 5:1 hexane-EA, ^{1}H NMR (500 MHz, $CDCl_3$) δ 4.26 (t, *J* = 2.1 Hz, 2 H), 3.23 – 3.14 (m, 2 H), 2.22 (s, 1 H), 2.15 (tt, *J* = 7.1, 2.3 Hz, 1 H), 1.56 – 1.43 (m, 1 H), 1.39 – 1.24 (m, 2 H), 0.89 (t, *J* = 7.0 Hz, 3 H); ^{13}C NMR (125 MHz, $CDCl_3$): δ 81.12, 80.71, 78.37, 73.23, 51.14, 31.02, 28.77, 22.15, 18.64, 13.97, 9.71.

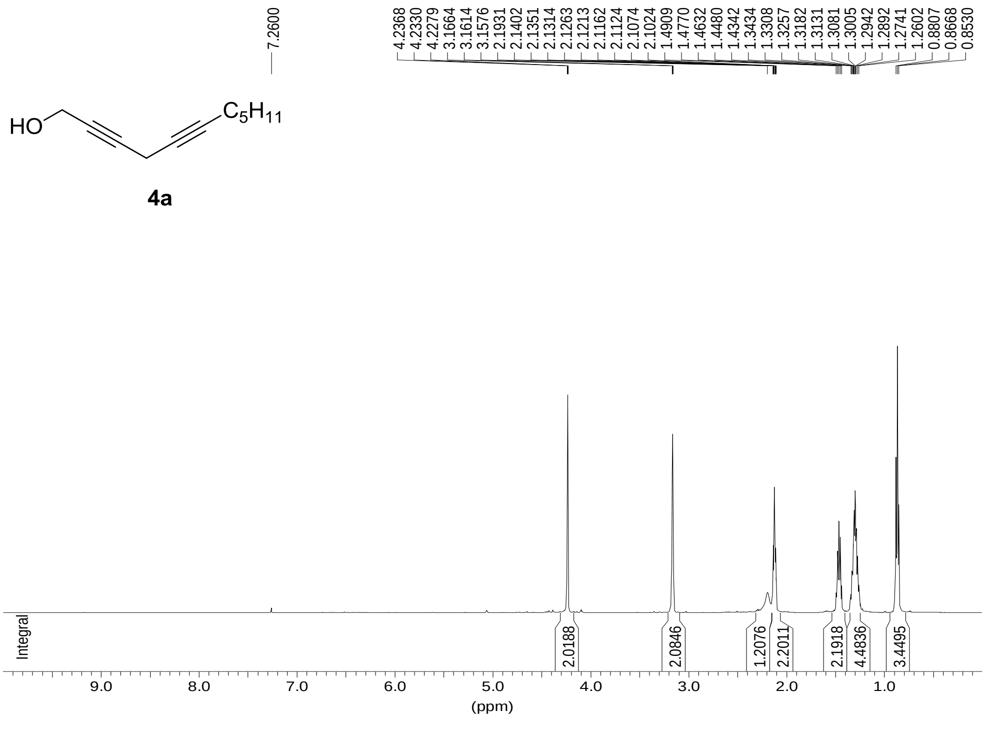

4a

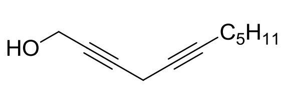

4a

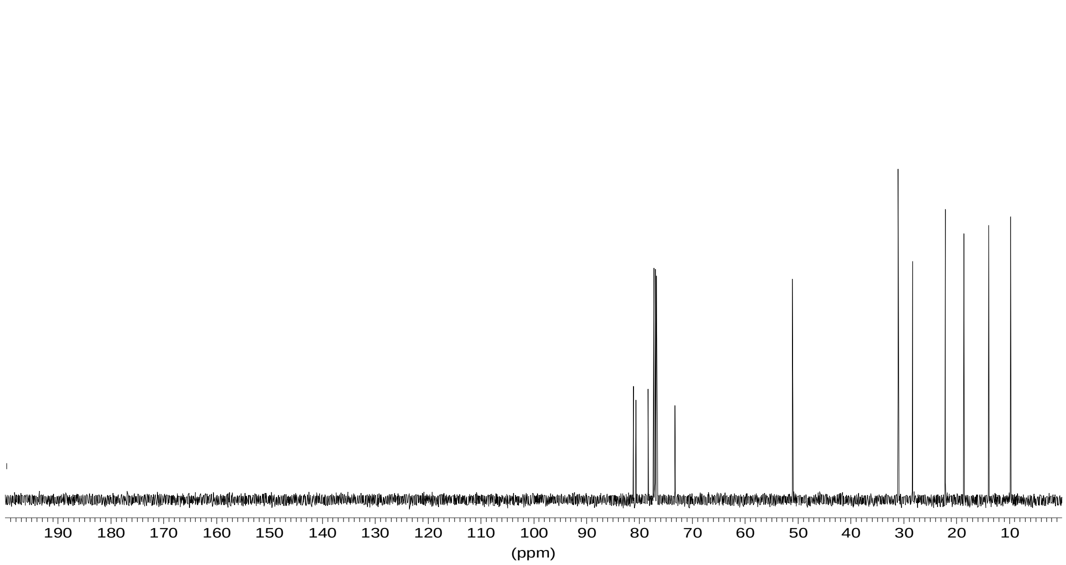

Nona-2,5-diyn-1-ol (4b): Pale yellow oil, 96% yield, eluent system: 5:1 hexane-EA, ^{1}H NMR (300 MHz, $CDCl_3$) δ 4.19 (t, *J* = 2.2 Hz, 2H), 3.13 (dd, *J* = 4.6, 2.3 Hz, 2H), 2.77 (s, 1H), 2.11 – 2.00 (m, 2H), 1.54 – 1.34 (m, 2H), 0.90 (t, *J* = 7.4 Hz, 3H); ^{13}C NMR (75 MHz, $CDCl_3$) δ 80.76, 80.35, 78.25, 73.38, 50.78, 21.91, 20.44, 13.27, 9.59.

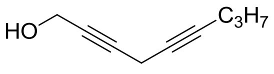

4b

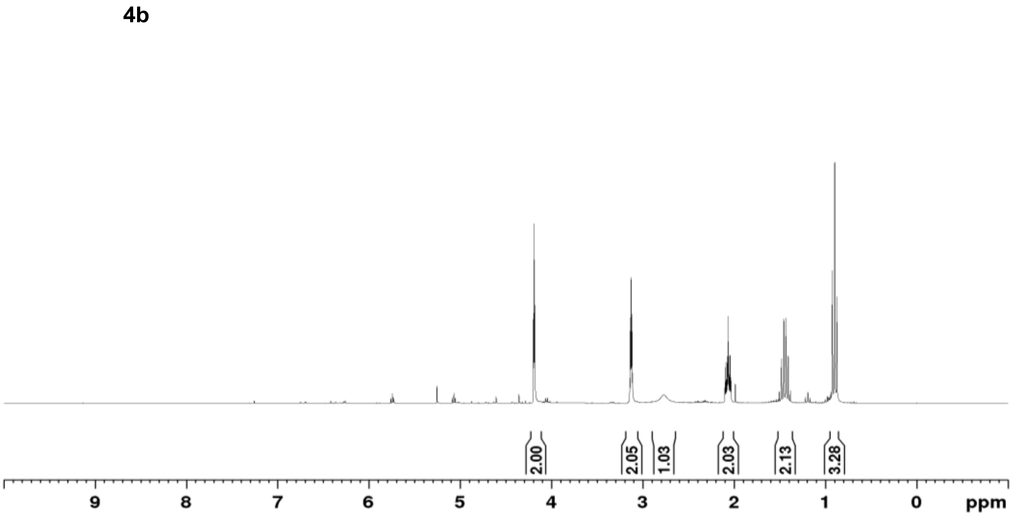

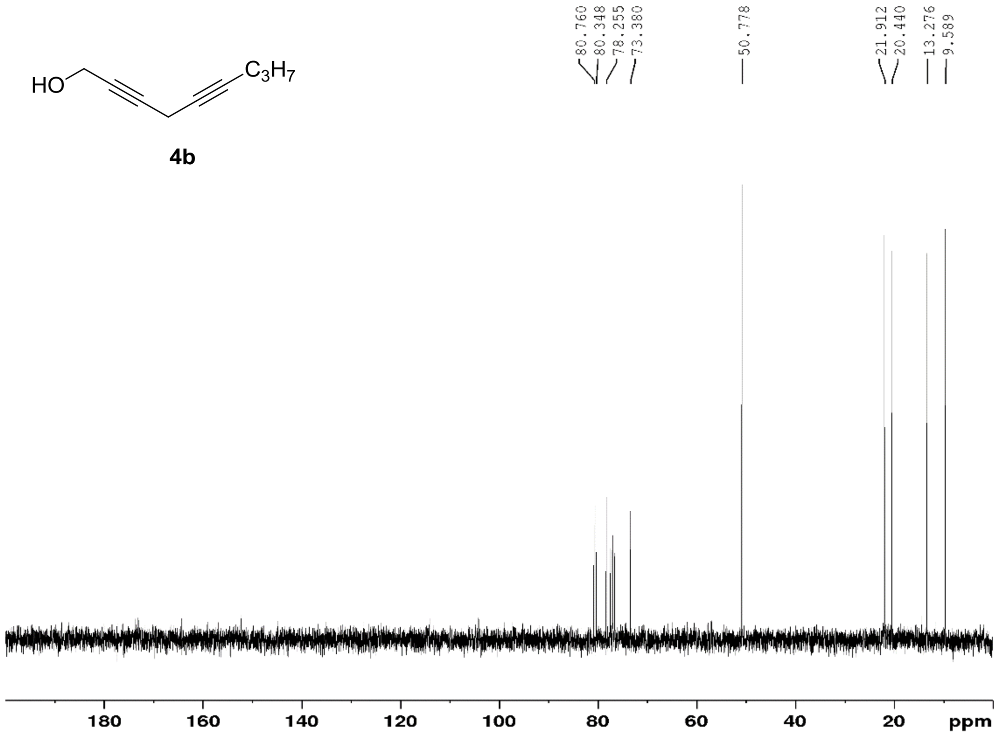

4b

Tetradeca-2,5-diyn-1-ol (4c): Yellow oil, 74% yield, eluent system: 5:1 hexane-EA, ^{1}H NMR (300 MHz, $CDCl_3$) δ 4.17 (t, *J* = 2.1 Hz, 2 H), 3.11 (dd, *J* = 4.5, 2.2 Hz, 2 H), 2.07 (tt, *J* = 7.1, 2.3 Hz, 2 H), 1.39 (dd, *J* = 14.6, 7.1 Hz, 2 H), 1.32 – 1.08 (m, 10 H), 0.80 (t, *J* = 6.7 Hz, 3 H); ^{13}C NMR (75 MHz, $CDCl_3$) δ 80.85, 80.19, 78.24, 73.20, 50.60, 31.63, 28.98, 28.91, 28.70, 28.50, 22.43, 18.43, 13.83, 9.55.

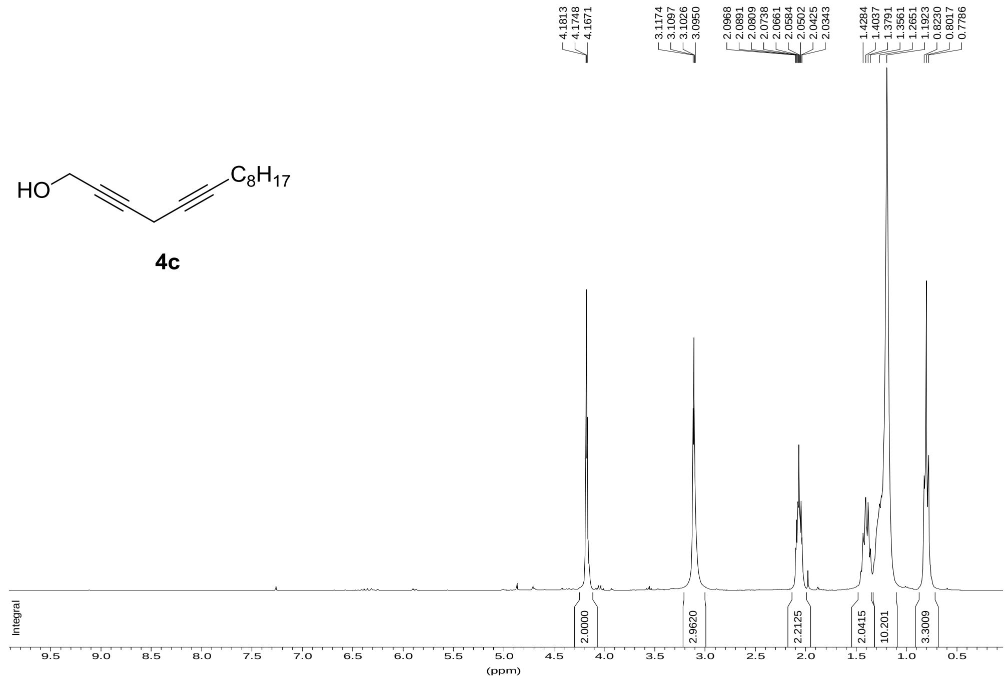

4c

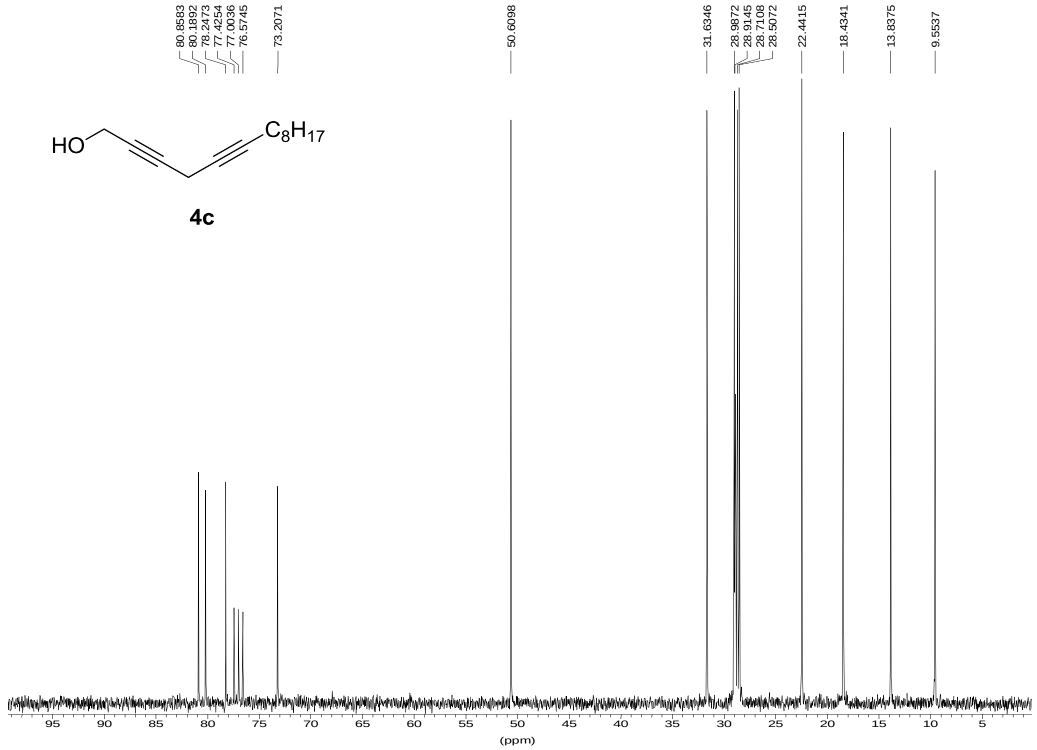

6-phenylhexa-2,5-diyn-1-ol (4d): Yellow oil, 79% yield, eluent system: 5:1 hexane-EA, ^{1}H NMR (500 MHz, $CDCl_3$) δ 7.44– 7.42 (m, 2H), 7.30 – 7.29 (m, 3H), 4.29 (t, *J* = 2.1 Hz, 2H), 3.45 (t, *J* = 2.1 Hz, 2H), 1.83 (s, 1H); ^{13}C NMR (125 MHz, $CDCl_3$) δ 131.72(2C), 128.26(2C), 128.21, 122.93, 83.06, 80.90, 79.90, 78.92, 51.27, 10.60.

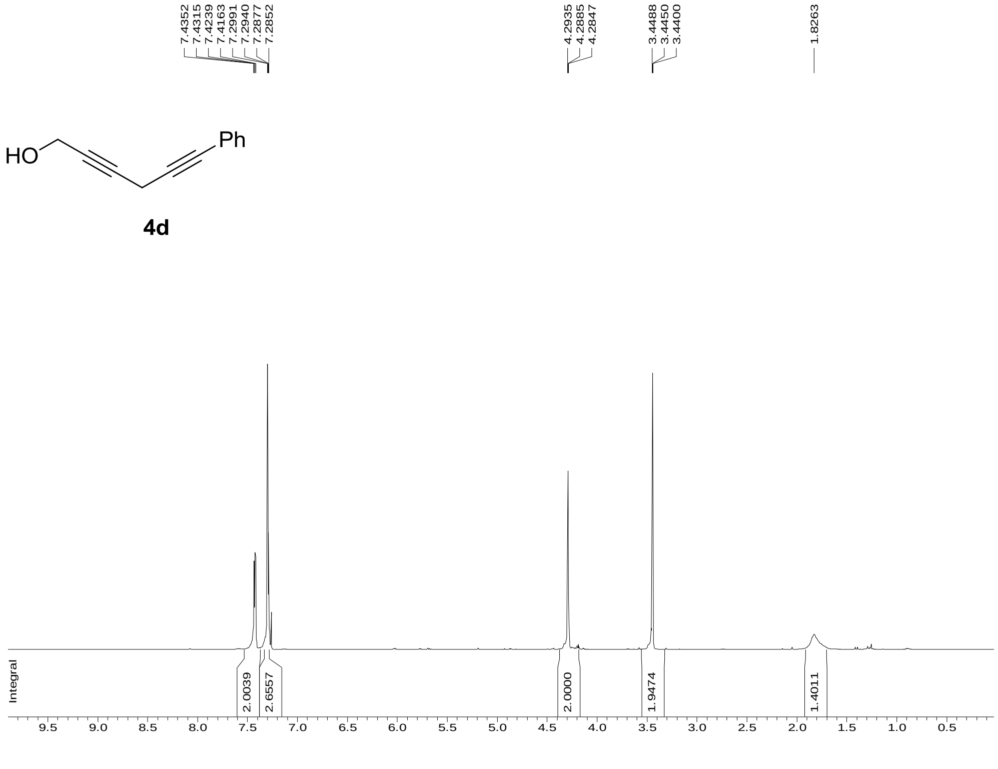

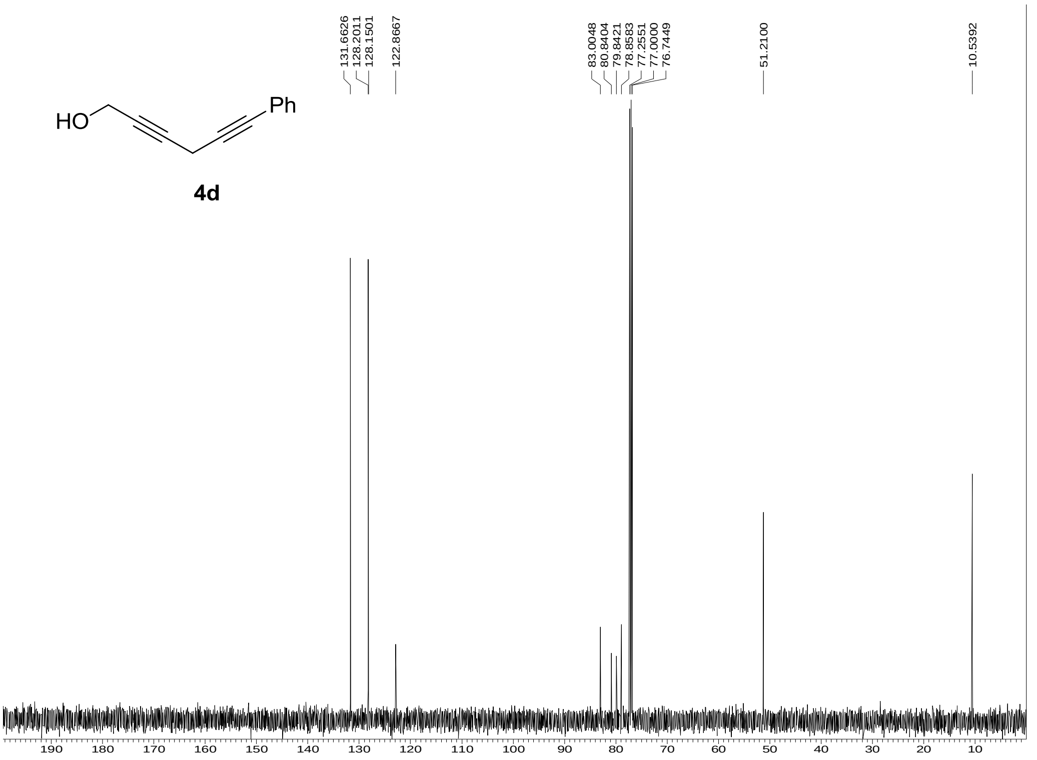

Dodeca-2,5-diyn-1-ol (4e): Pale yellow oil, 86% yield, eluent system: 5:1 hexane-EA, ^{1}H NMR (300 MHz, $CDCl_3$) δ 4.21 (t, *J* = 2.2 Hz, 2H), 3.14 (p, *J* = 2.3 Hz, 2H), 2.61 (s, 1H), 2.10 (tt, *J* = 7.2, 2.4 Hz, 2H), 1.48 – 1.34 (m, 2H), 1.33 – 1.21 (m, 6H), 0.84 (t, *J* = 6.9 Hz, 3H); ^{13}C NMR (75 MHz, $CDCl_3$) δ 81.05, 80.49, 78.28, 73.23, 50.89, 31.21, 28.53, 28.44, 22.41, 18.53, 13.89, 9.67.

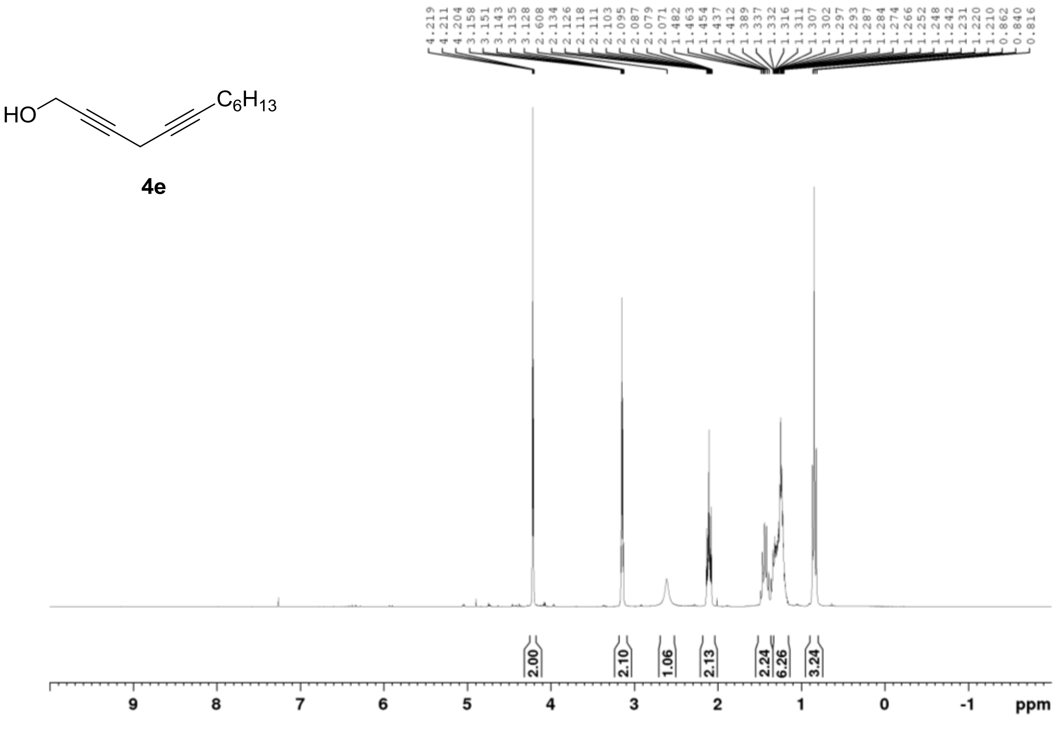

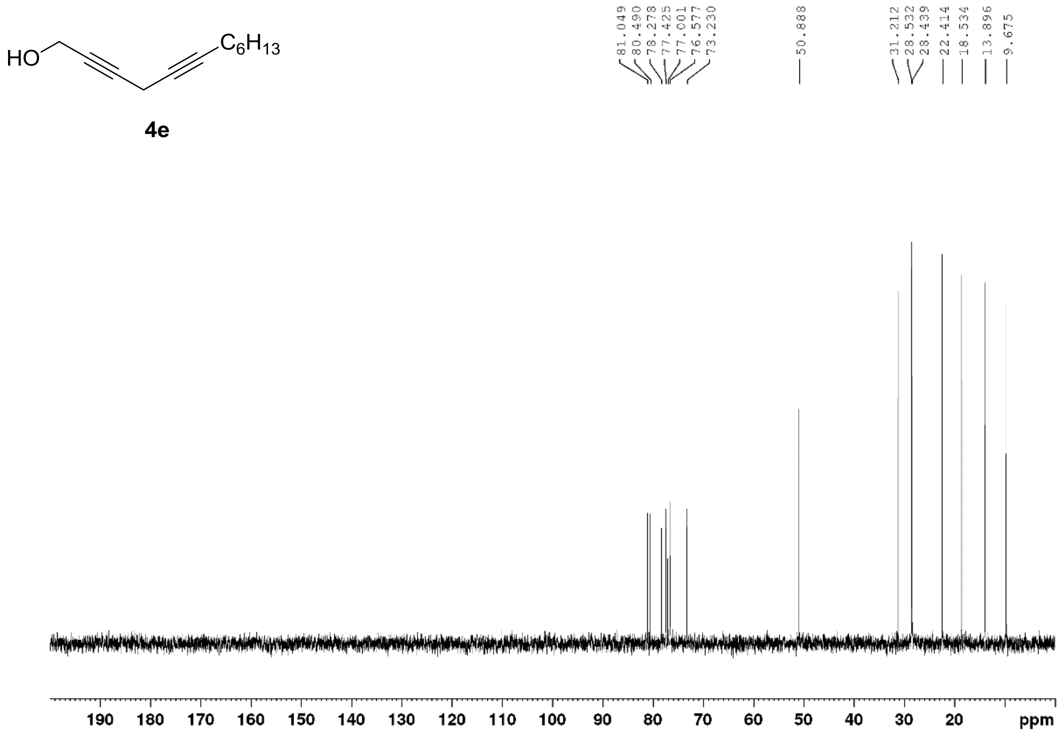

1-bromoundeca-2,5-diyne (5a): Yellow oil, 73% yield. ^{1}H NMR (500 MHz, $CDCl_3$) δ 3.91 (dd, *J* = 3.0, 1.5 Hz, 2 H), 3.20 (dd, *J* = 3.2, 1.5 Hz, 2 H), 2.17 – 2.08 (m, 2 H), 1.51 – 1.43 (m, 2 H), 1.38 – 1.24 (m, 4 H), 0.86 (t, *J* = 6.95 Hz, 3H); ^{13}C NMR (125 MHz, $CDCl_3$) δ 82.10, 81.38, 75.23, 72.77, 31.04, 28.35, 22.20, 18.62, 14.85, 13.96, 10.07.

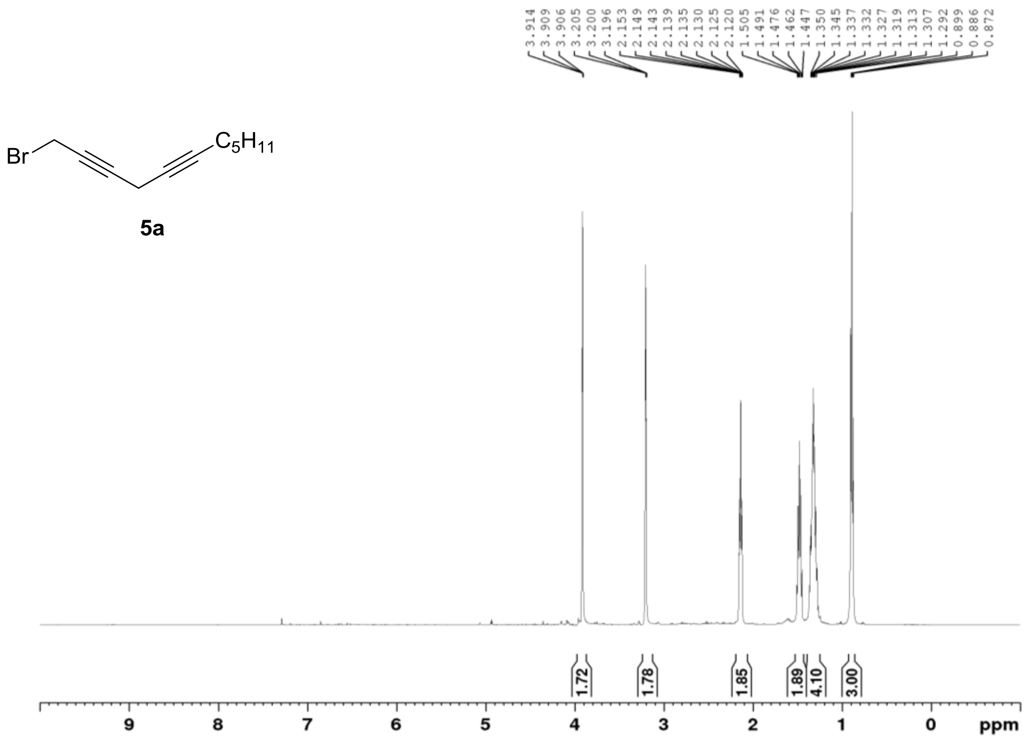

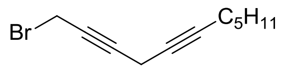

5a

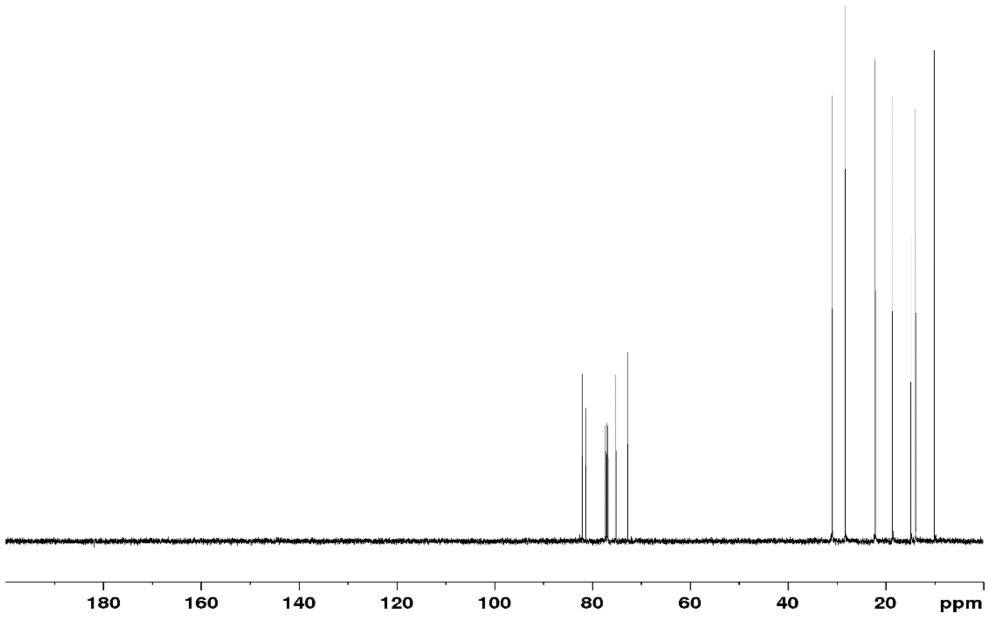

1-bromonona-2,5-diyne (5b): Yellow oil, 77% yield. ^{1}H NMR (500 MHz, $CDCl_3$) δ 3.90 (t, *J* = 2.3 Hz, 2H), 3.29 – 3.14 (m, 2H), 2.11 (tt, *J* = 7.1, 2.4 Hz, 2H), 1.59 – 1.35 (m, 2H), 0.94 (t, *J* = 7.4 Hz, 3H); ^{13}C NMR (125 MHz, $CDCl_3$) δ 81.98, 81.07, 75.12, 72.83, 21.96, 20.52, 14.76, 13.35, 9.95.

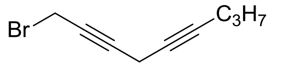

5b

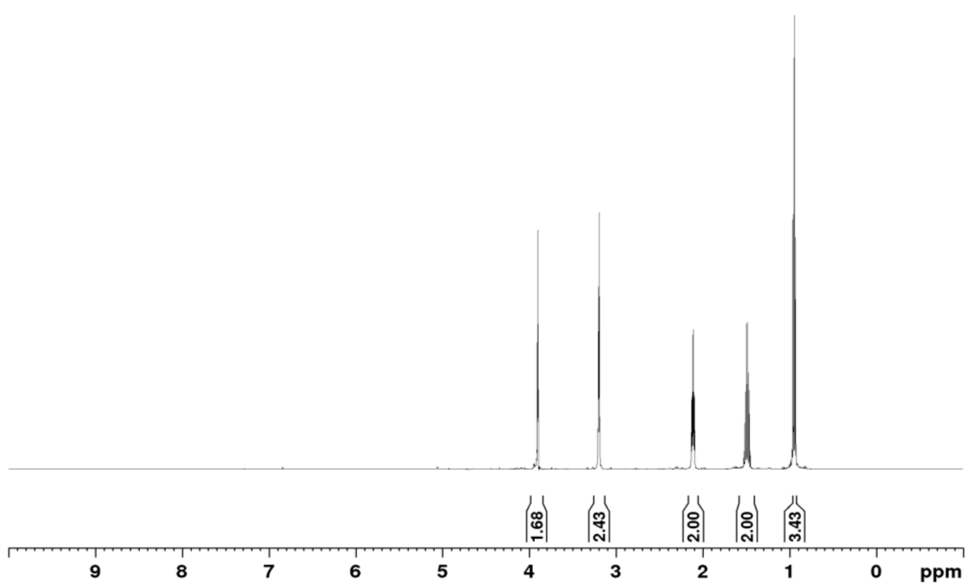

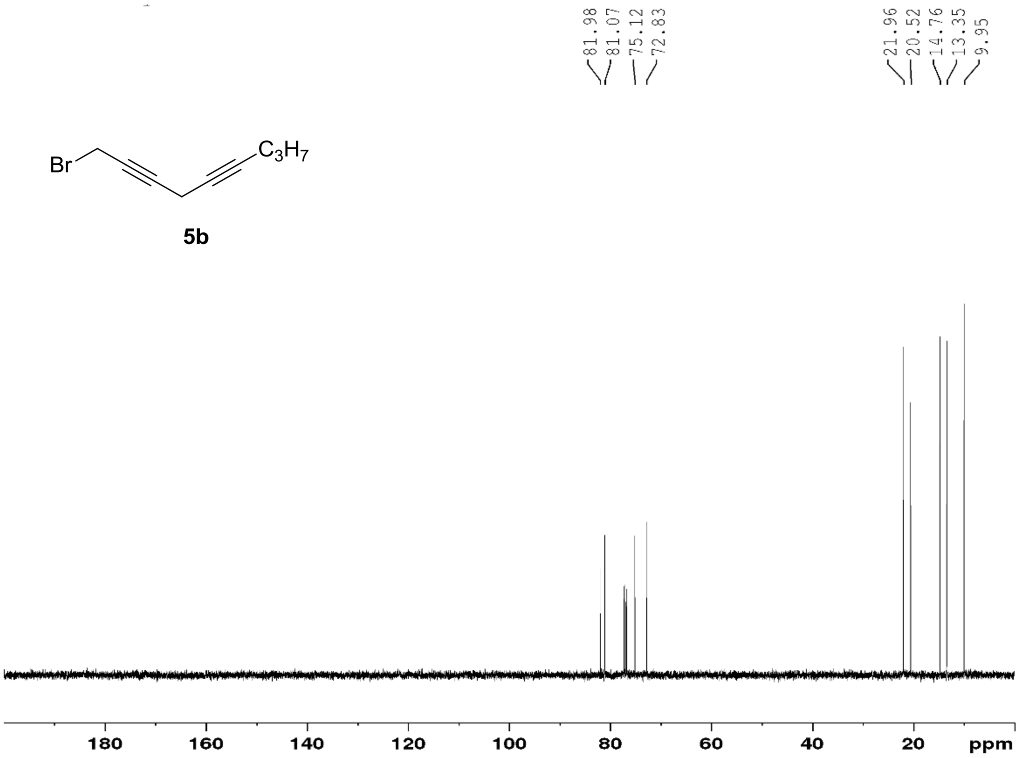

1-bromotetradeca-2,5-diyne (5c): Yellow oil, 60% yield. ^{1}H NMR (300 MHz, $CDCl_3$) δ 3.88 (t, *J* = 2.3 Hz, 2 H), 3.17 (dd, *J* = 4.6, 2.3 Hz, 2 H), 2.10 (tt, *J* = 7.1, 2.3 Hz, 2 H), 1.43 (dd, *J* = 14.5, 7.1 Hz, 2 H), 1.37 – 1.13 (m, 10 H), 0.84 (t, *J* = 6.6 Hz, 3 H); ^{13}C NMR (75 MHz, $CDCl_3$) δ 81.94, 81.22, 75.09, 72.62, 31.71, 29.07, 28.98, 28.75, 28.54, 22.54, 18.53, 14.69, 13.96, 9.94.

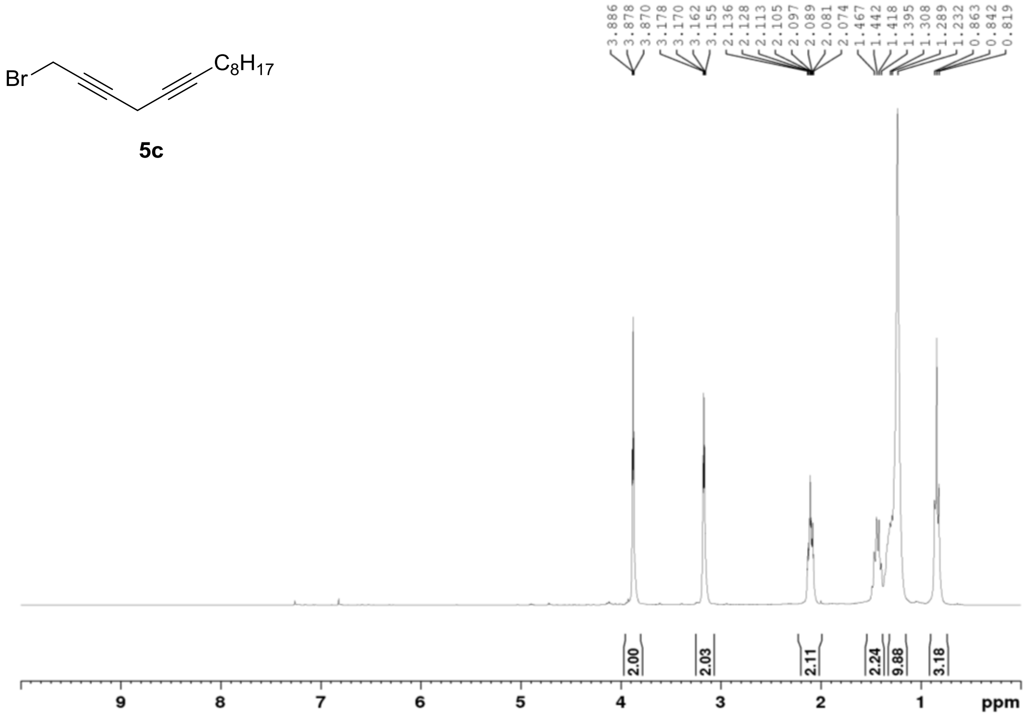

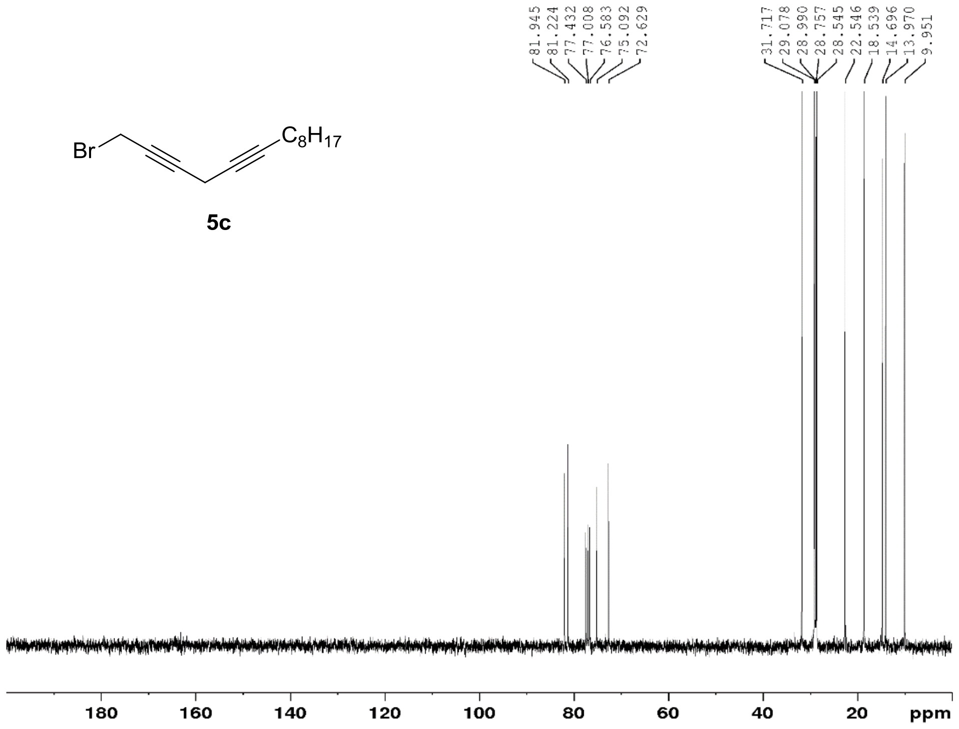

(6-bromohexa-1,4-diyn-1-yl)benzene (5d): Yellow oil, 65% yield. ^{1}H NMR (500 MHz, $CDCl_3$) δ 7.44 – 7.42 (m, 2H), 7.30 (dd, *J* = 5.0, 1.8 Hz, 3H), 3.94 (t, *J* = 2.3 Hz, 2H), 3.48 (t, *J* = 2.3 Hz, 2H); ^{13}C NMR (125 MHz, $CDCl_3$) δ 131.68(2C), 128.21(2C), 128.20, 122.80, 82.48, 81.07, 81.03, 75.75, 14.71, 10.80.

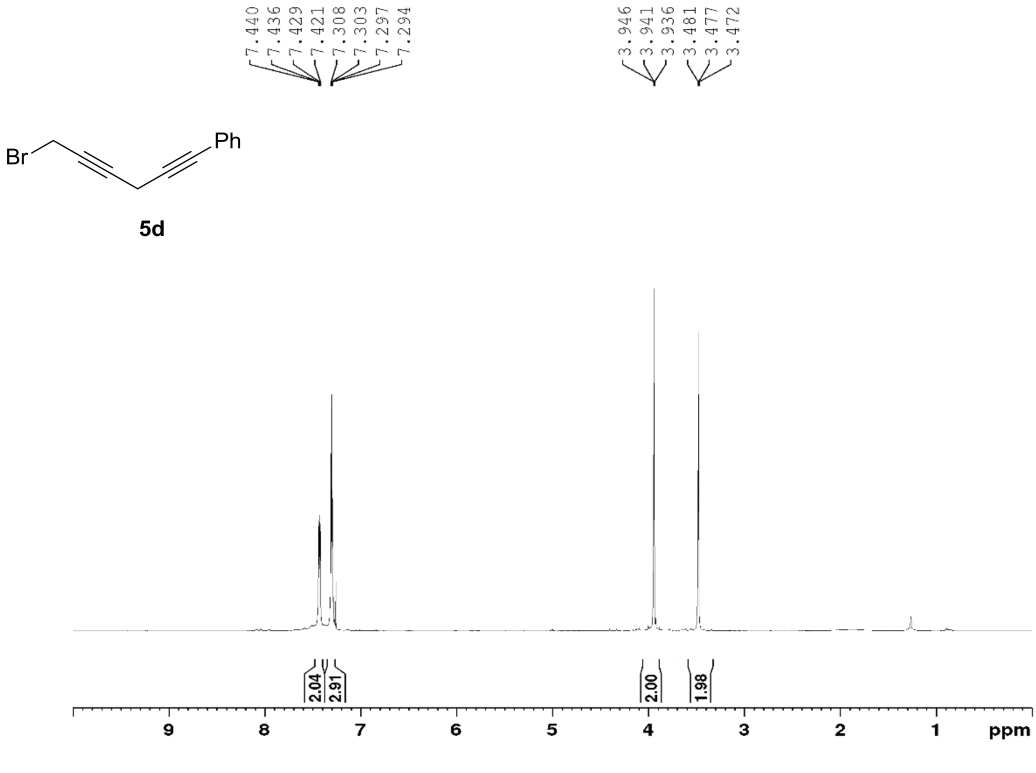

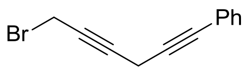

5d

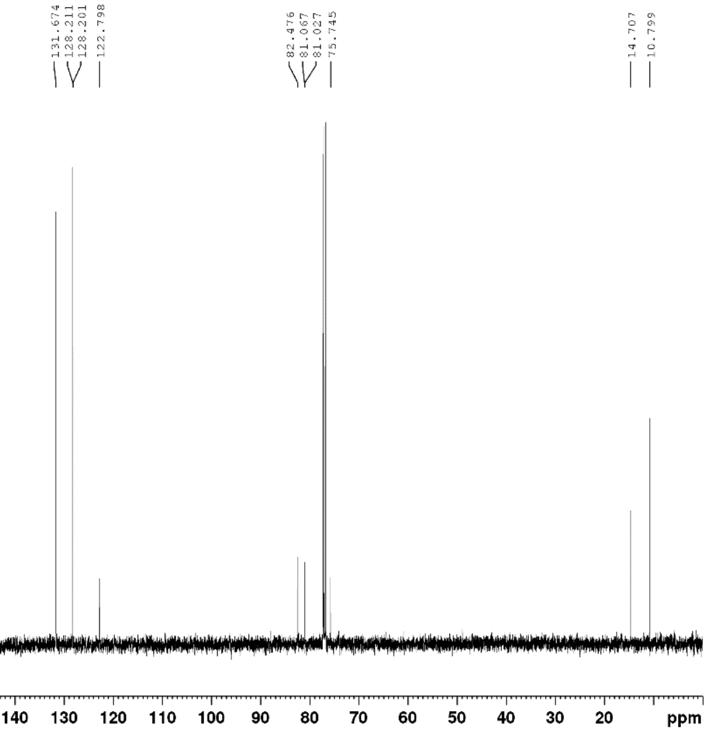

1-bromododeca-2,5-diyne (5e): Yellow oil, 69% yield. ^{1}H NMR (500 MHz, $CDCl_3$) δ 3.93 (t, *J* = 2.3 Hz, 2H), 3.23 – 3.22 (m, 2H), 2.16 (tt, *J* = 7.2, 2.4 Hz, 2H), 1.49 (dd, *J* = 15.0, 7.4 Hz, 2H), 1.40 – 1.36 (m, 2H), 1.30 (ddd, *J* = 14.4, 8.8, 2.8 Hz, 4H), 0.90 (t, *J* = 7.0 Hz, 3H); ^{13}C NMR (125 MHz, $CDCl_3$) δ 82.08, 81.40, 75.17, 72.71, 31.28, 28.57, 28.49, 22.50, 18.62, 14.82, 14.00, 10.05.

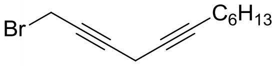

5e

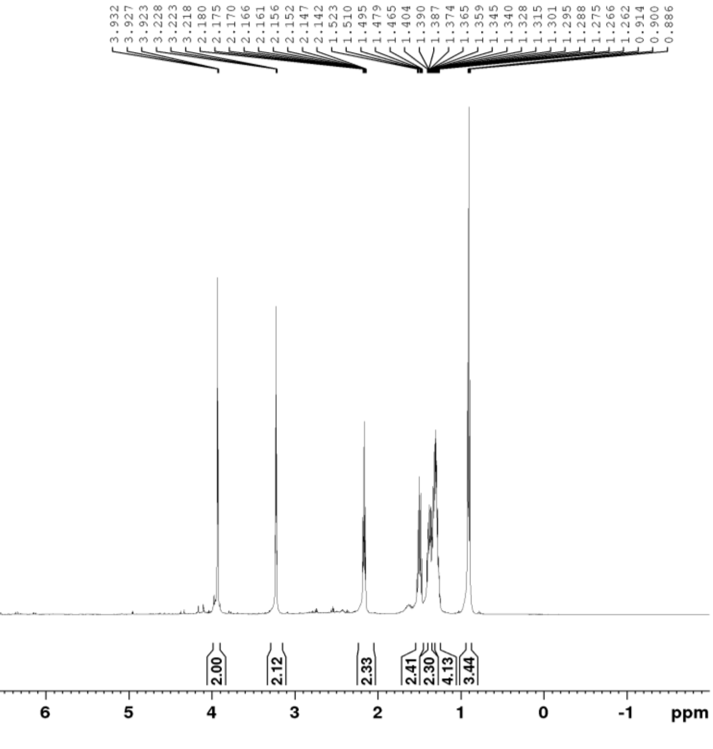

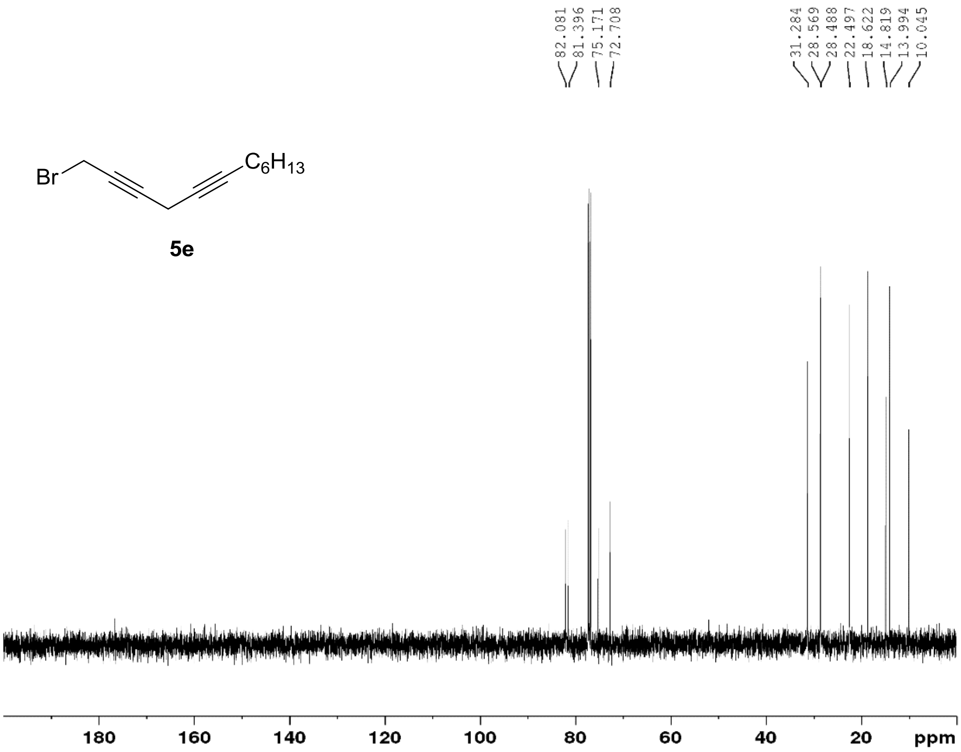

Tetradeca-2,5,8-triyn-1-ol (6a): Yellow oil, 87% yield, eluent system: 5:1 hexane-EA, ^{1}H NMR (300 MHz, $CDCl_3$) δ 4.23 (t, *J* = 1.9 Hz, 2 H), 3.27 – 3.00 (m, 4 H), 2.11 (ddd, *J* = 9.2, 4.6, 2.3 Hz, 2 H), 1.45 (dd, *J* = 13.9, 6.9 Hz, 2 H), 1.37 – 1.14 (m, 4 H), 0.87 (t, *J* = 6.9 Hz, 3 H); ^{13}C NMR (75 MHz, $CDCl_3$) δ 80.87, 79.71, 78.63, 75.40, 73.61, 73.44, 50.79, 30.93, 28.26, 22.06, 18.50, 13.81, 9.70, 9.55.

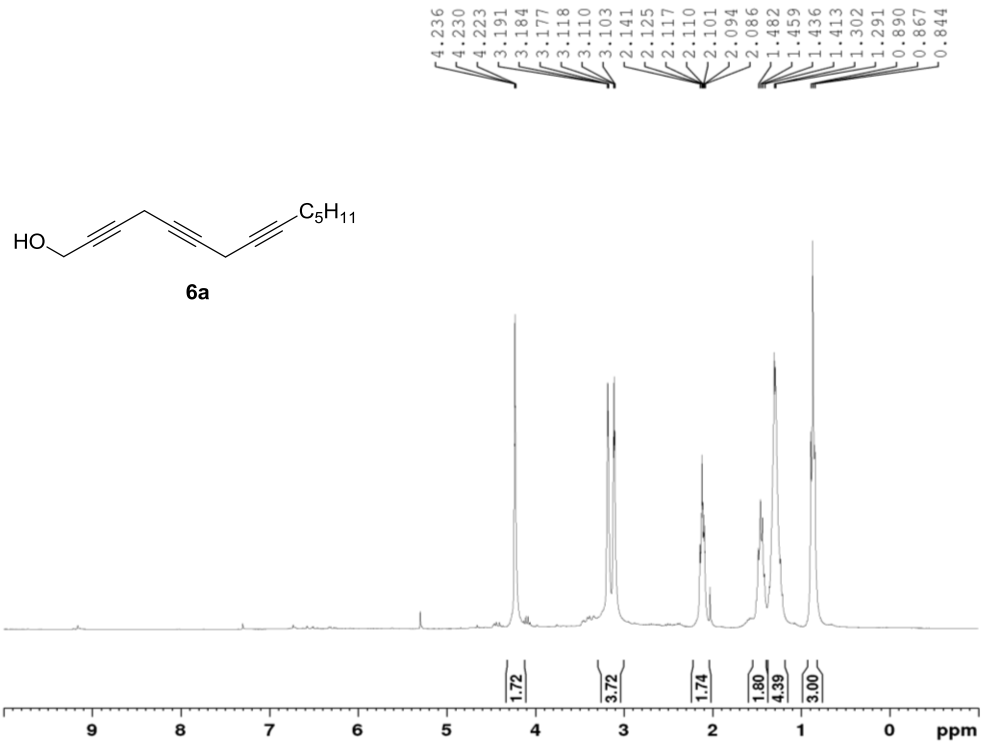

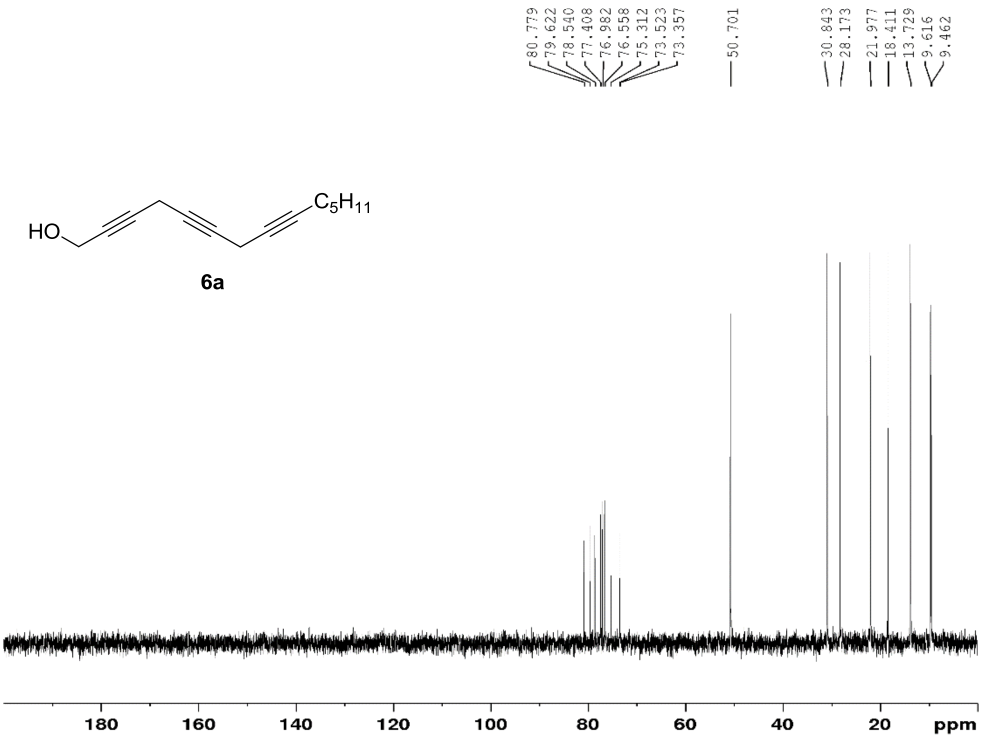

Dodeca-2,5,8-triyn-1-ol (6b): Yellow oil, 66% yield, eluent system: 5:1 hexane-EA, ^{1}H NMR (300 MHz, $CDCl_3$) δ 4.25 (t, *J* = 2.1 Hz, 2H), 3.38 – 2.95 (m, 4H), 2.13 (tt, *J* = 7.1, 2.4 Hz, 2H), 1.91 (s, 1H), 1.51 (dd, *J* = 14.5, 7.2 Hz, 2H), 0.96 (dd, *J* = 9.7, 5.0 Hz, 3H); ^{13}C NMR (75 MHz, $CDCl_3$) δ 80.83, 80.11, 78.64, 75.55, 73.62, 51.21, 22.09, 20.65, 13.47, 9.87, 9.71.

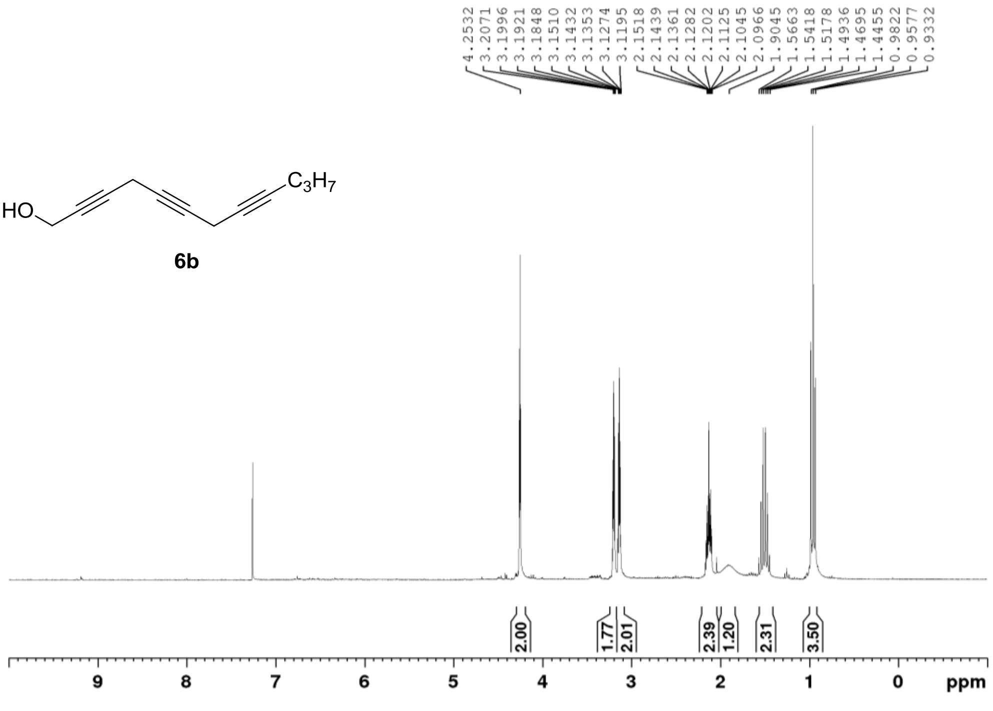

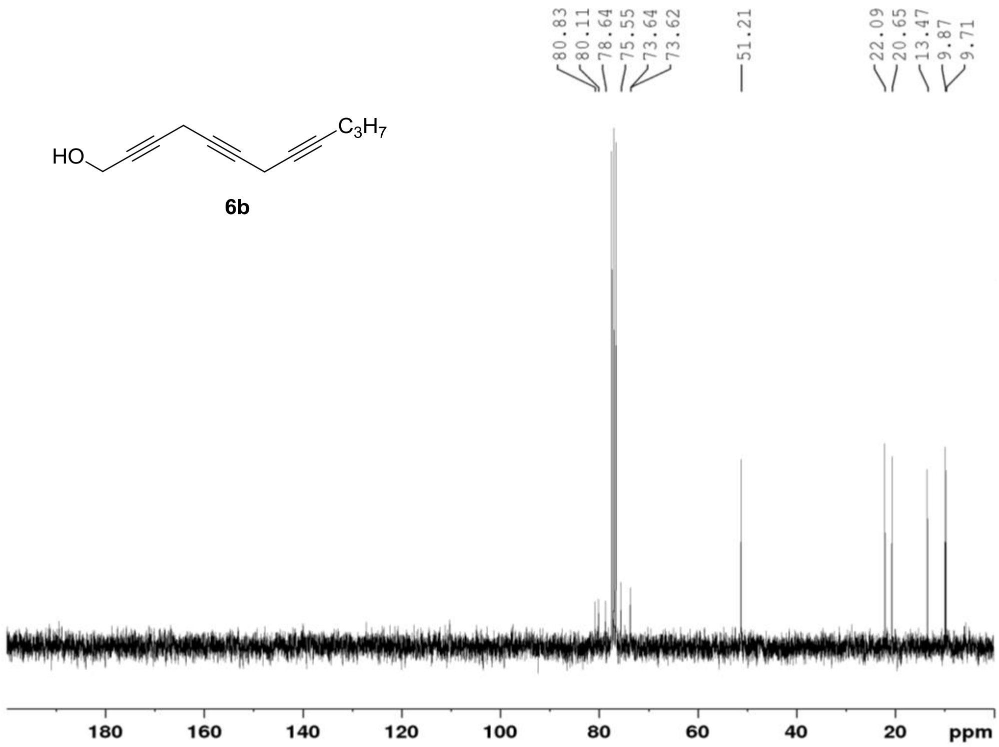

Heptadeca-2,5,8-triyn-1-ol (6c): Pale yellow solid, 62% yield, eluent system: 5:1 hexane-EA,^{1}H NMR (300 MHz, $CDCl_3$) δ 4.20 (dd, *J* = 7.6, 6.1 Hz, 2 H), 3.19 – 2.97 (m, 4 H), 2.43 (s, 1 H), 2.08 (ddd, *J* = 11.7, 9.3, 7.1 Hz, 2 H), 1.51 – 1.00 (m, 12 H), 0.91 – 0.67 (m, 3 H); ^{13}C NMR (125 MHz, $CDCl_3$) δ 81.33, 81.08, 75.80, 75.57, 73.42, 73.13, 31.84, 29.19, 29.11, 28.90, 28.71, 22.66, 18.70, 14.69, 14.10, 10.14, 9.75.

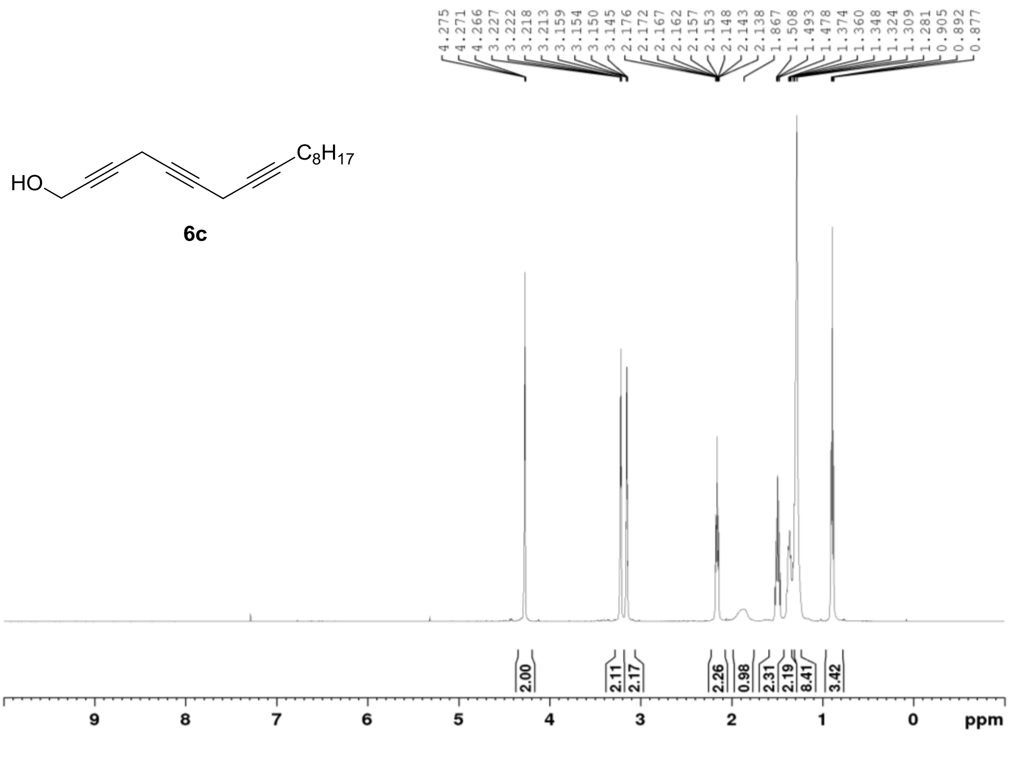

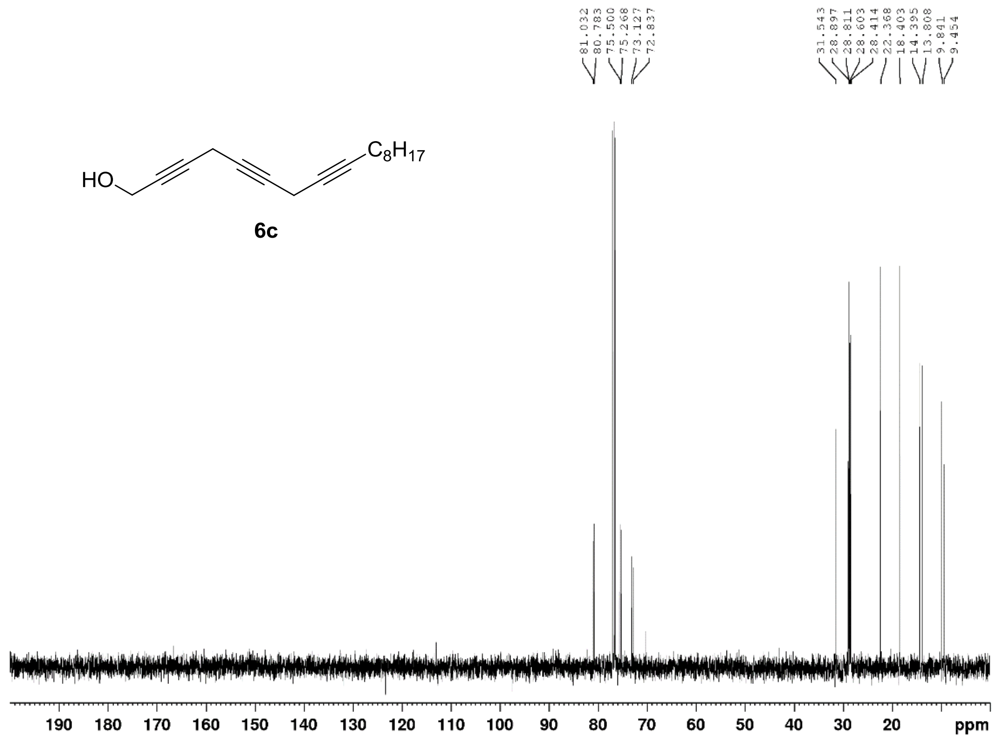

9-phenylnona-2,5,8-triyn-1-ol (6d): Yellow oil, 75% yield, eluent system: 5:1 hexane-EA, ^{1}H NMR (500 MHz, $CDCl_3$) δ 7.43 – 7.41 (m, 2H), 7.30 – 7.26 (m, 3H), 4.25 (t, *J* = 2.1 Hz, 2H), 3.39 (t, *J* = 2.3 Hz, 2H), 3.24 – 3.22 (m, 2H), 2.46 (s, 1H); ^{13}C NMR (125 MHz, $CDCl_3$) δ 131.73(2C), 128.27(2C), 128.18, 123.00, 83.44, 80.78, 79.84, 78.94, 74.68, 74.41, 51.07, 10.51, 9.93.

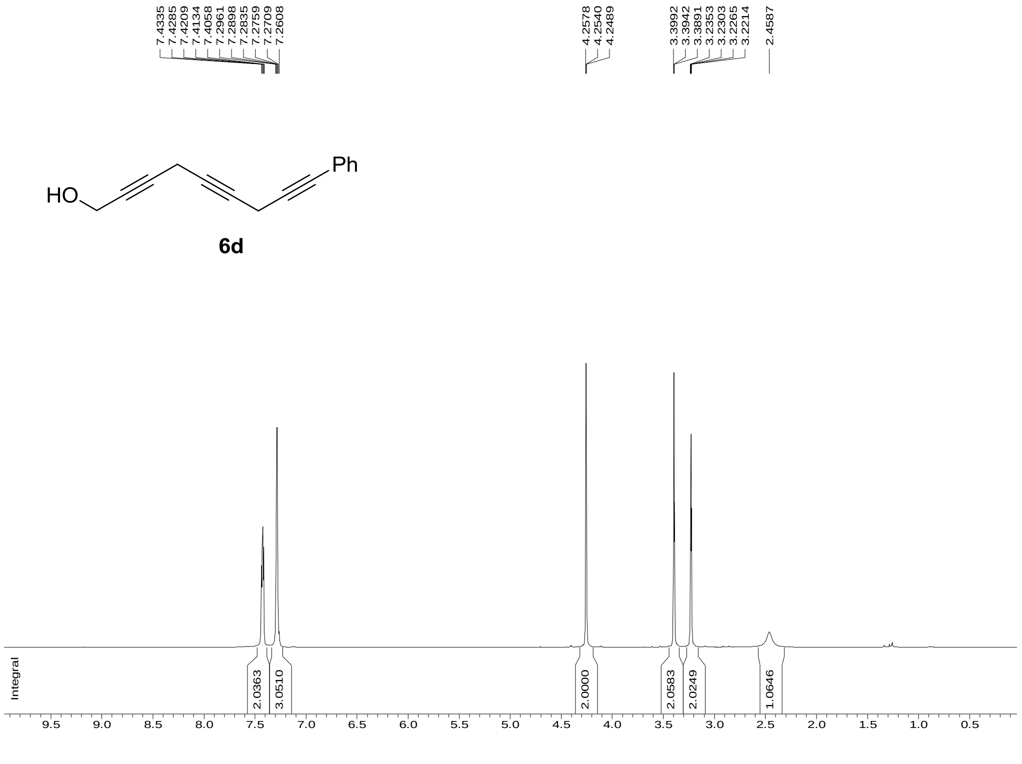

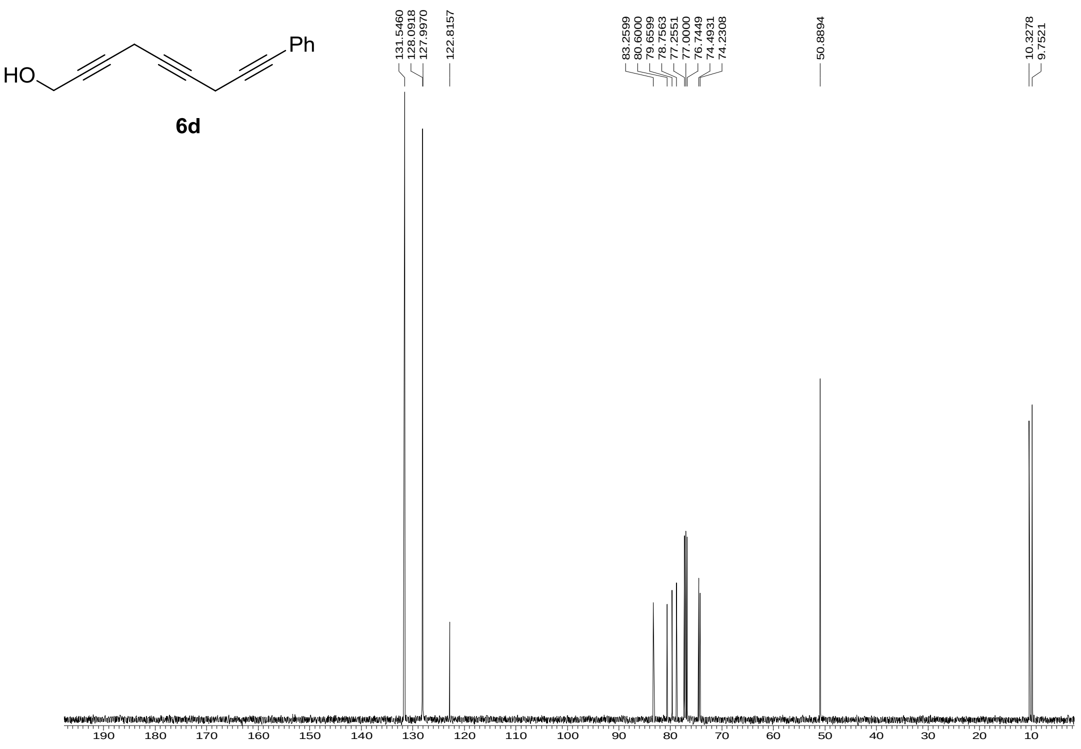

Pentadeca-2,5,8-triyn-1-ol (6e): Yellow oil, 77% yield, eluent system: 5:1 hexane-EA, ^{1}H NMR (500 MHz, $CDCl_3$) δ 4.26 (s, 2H), 3.21-3.19 (m, 2H), 3.14 (p, *J* = 2.3 Hz, 2H), 2.16 – 2.13 (m, 2H), 1.51 – 1.45 (m, 2H), 1.38 – 1.35 (m, 2H), 1.34 – 1.24 (m, 4H), 0.88 (t, *J* = 7.0 Hz, 3H); ^{13}C NMR (125 MHz, $CDCl_3$) δ 81.05, 80.14, 78.66, 77.26, 77.00, 76.75, 75.57, 73.61, 73.47, 51.25, 31.33, 28.66, 28.55, 22.53, 18.69, 14.03, 9.88, 9.74.

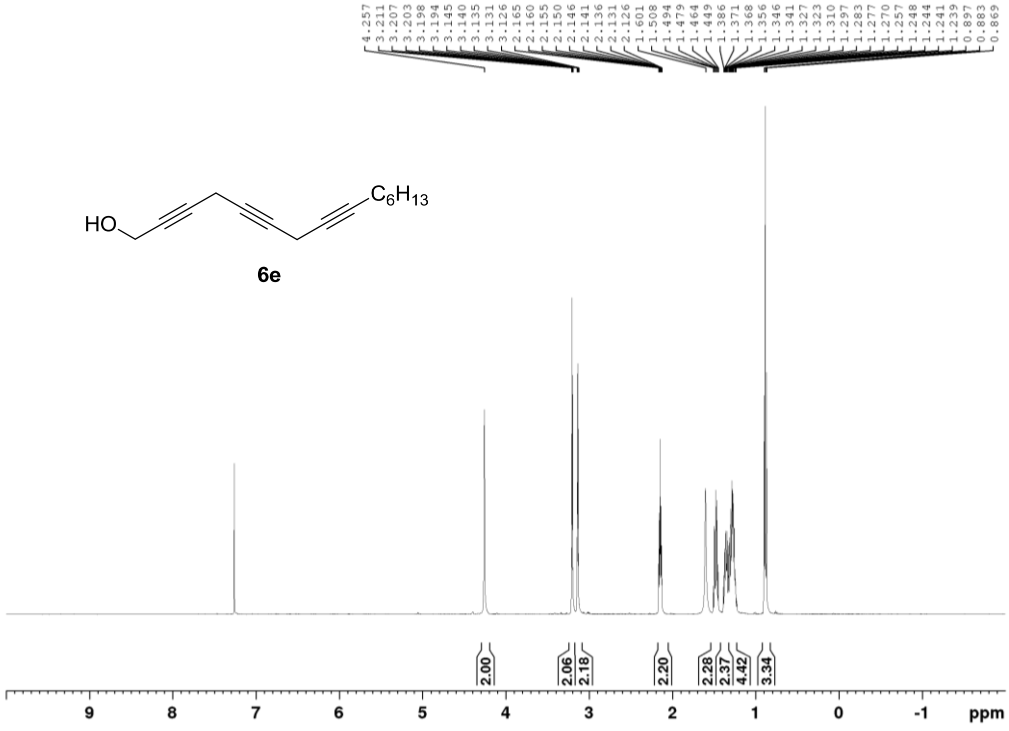

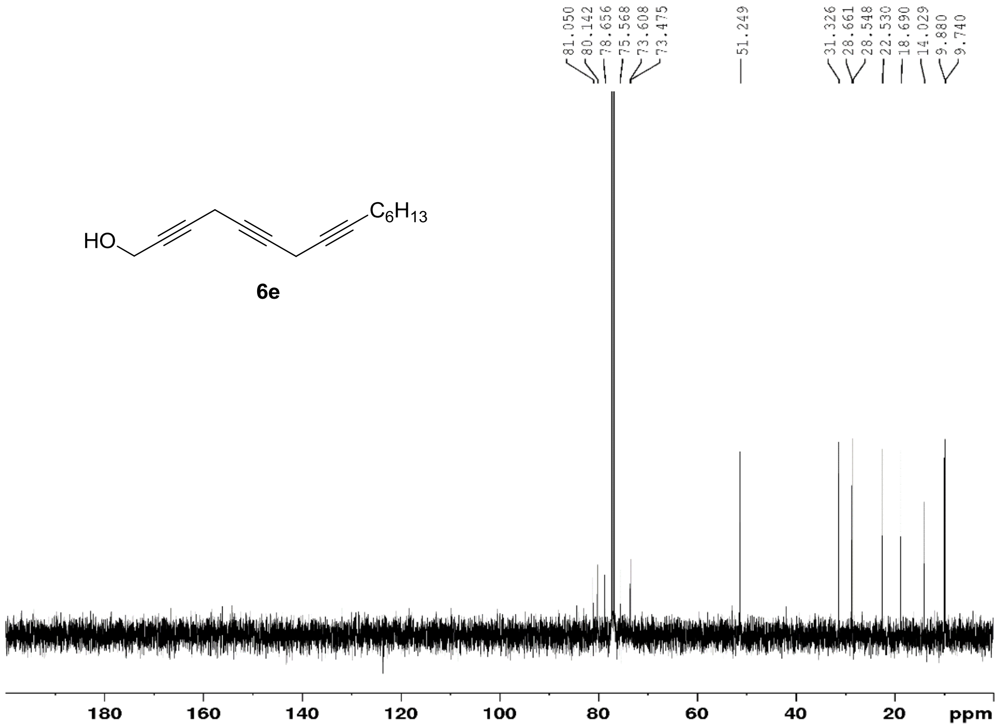

1-bromotetradeca-2,5,8-triyne (7a): Yellow oil, 71% yield. ^{1}H NMR (500 MHz, $CDCl_3$) δ 3.90 (dd, *J* = 3.1, 1.4 Hz, 2 H), 3.22 (dd, *J* = 4.6, 2.2 Hz, 2 H), 3.12 (dd, *J* = 4.7, 2.4 Hz, 2 H), 2.13 (tt, *J* = 7.1, 2.3 Hz, 2 H), 1.52 – 1.41 (m, 2 H), 1.39 – 1.25 (m, 4 H), 0.88 (t, *J* = 7.0 Hz, 3 H); ^{13}C NMR (125 MHz, $CDCl_3$) δ 81.34, 81.03, 75.81, 75.60, 73.49, 73.15, 31.06, 28.39, 22.20, 18.65, 14.71, 13.98, 10.12, 9.74.

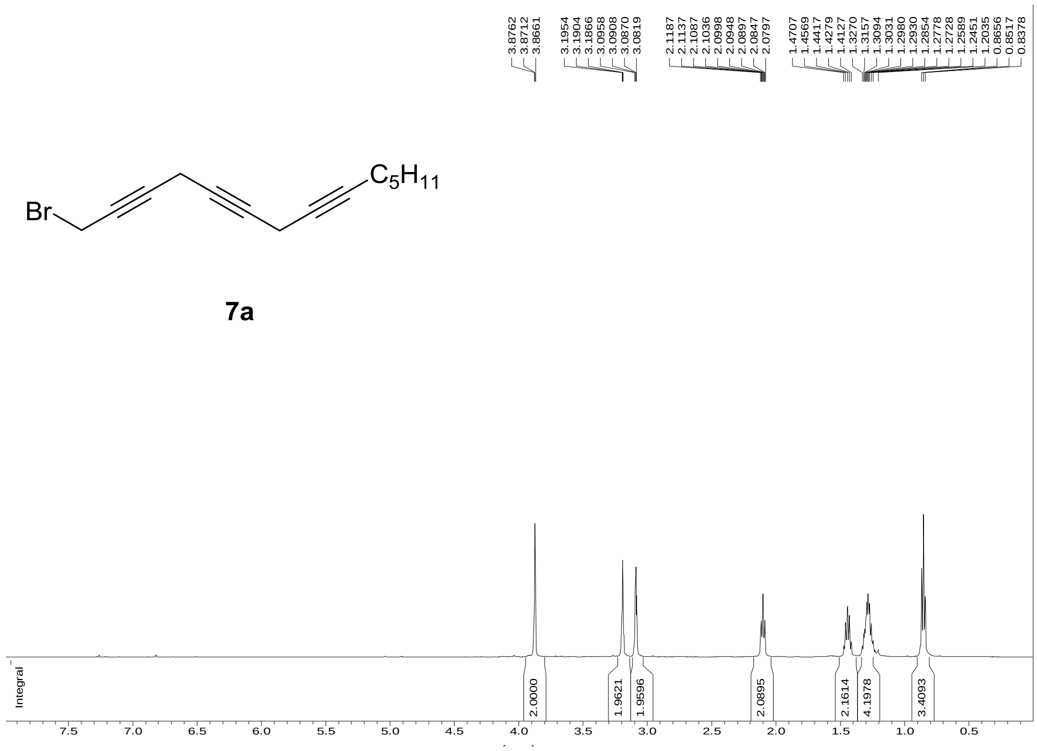

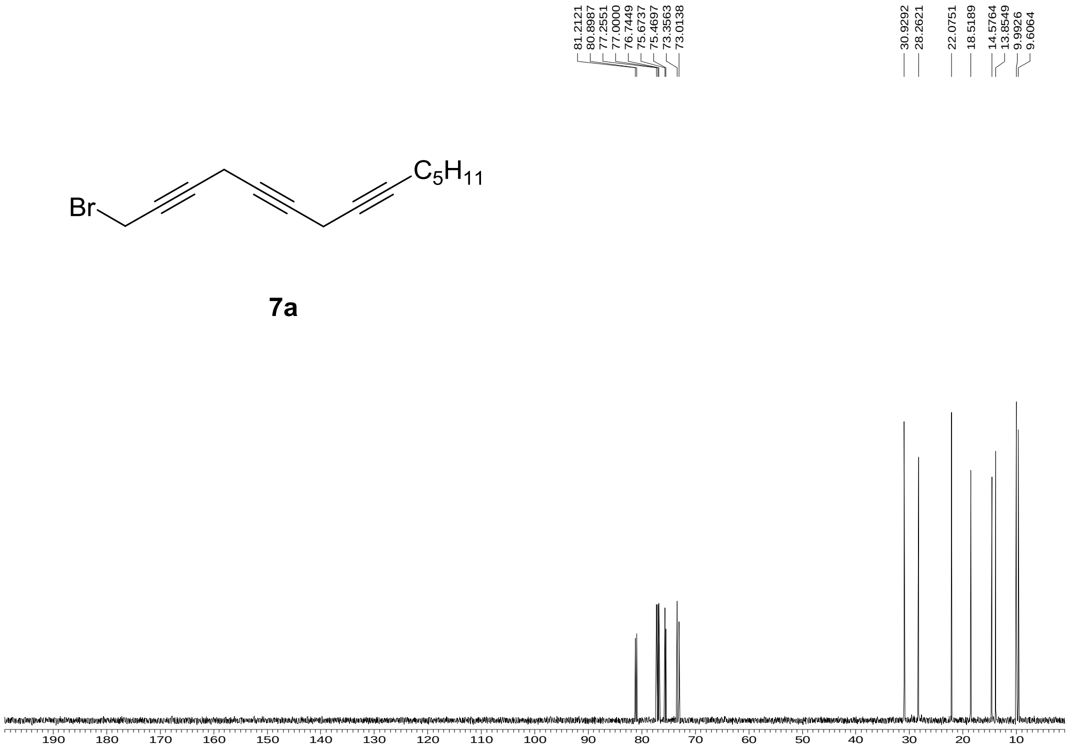

7a

1-bromododeca-2,5,8-triyne (7b): Yellow oil, 70% yield. ^{1}H NMR (300 MHz, CDCl$_3$) δ 3.89 (t, *J* = 2.3 Hz, 2H), 3.21 (p, *J* = 2.3 Hz, 2H), 3.12 (p, *J* = 2.4 Hz, 2H), 2.11 (tt, *J* = 7.1, 2.4 Hz, 2H), 1.58 – 1.39 (m, 2H), 0.95 (t, *J* = 7.4 Hz, 3H); ^{13}C NMR (75 MHz, CDCl$_3$) δ 81.27, 80.80, 75.72, 75.50, 73.54, 73.08, 22.05, 20.60, 14.65, 13.43, 10.06, 9.66.

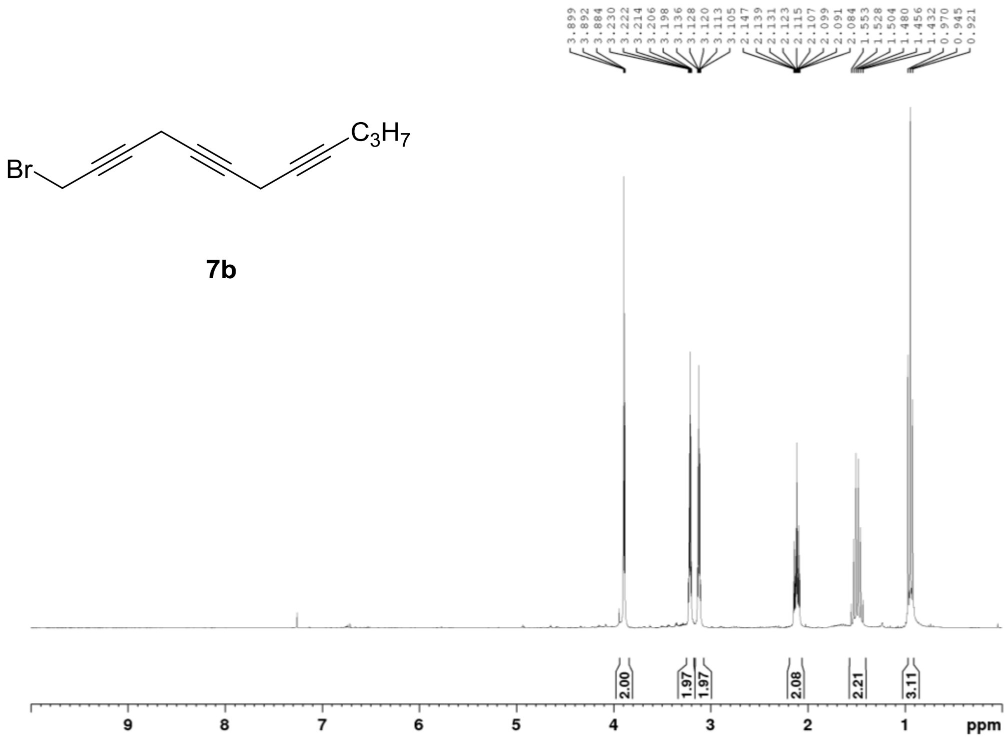

7b

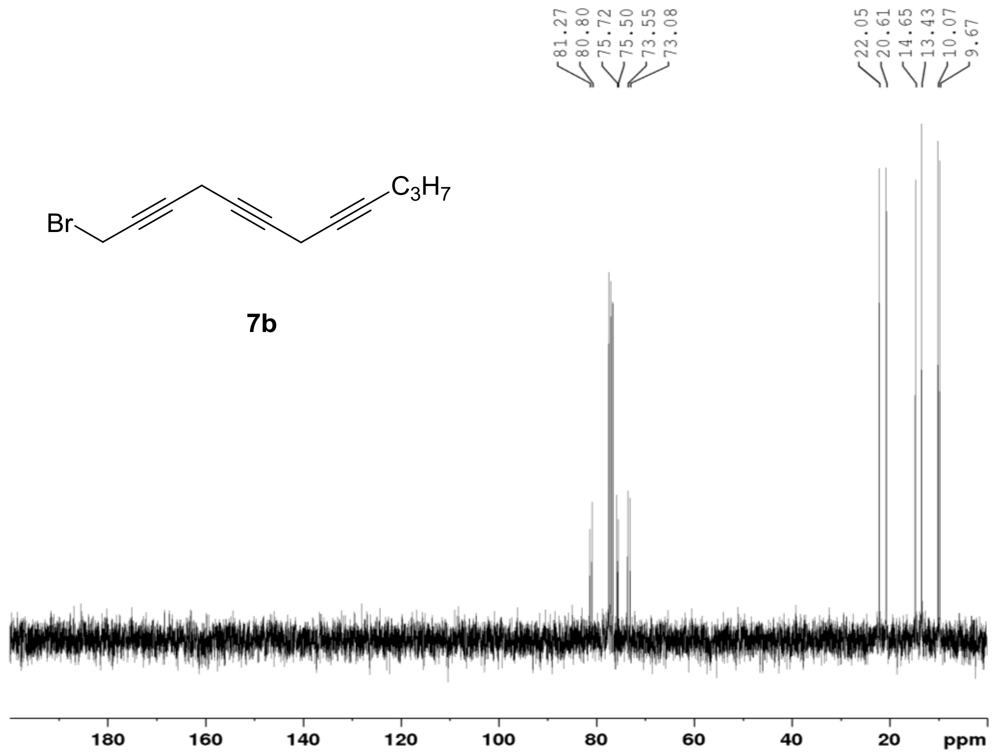

1-bromoheptadeca-2,5,8-triyne (7c): Yellow oil, 77% yield. ^{1}H NMR (300 MHz, $CDCl_3$) δ 3.90 (t, *J* = 2.3 Hz, 2 H), 3.26 – 3.17 (m, 2 H), 3.17 – 3.05 (m, 2 H), 2.13 (tt, *J* = 7.1, 2.4 Hz, 2 H), 1.46 (dd, *J* = 14.6, 7.2 Hz, 2 H), 1.40 – 1.15 (m, 10 H), 0.87 (t, *J* = 6.7 Hz, 3 H); ^{13}C NMR (125 MHz, $CDCl_3$) δ 81.33, 81.08, 77.30, 77.04, 76.79, 75.80, 75.57, 73.43, 73.13, 31.84, 29.19, 29.11, 28.90, 28.71, 22.66, 18.70, 14.69, 14.10, 10.14, 9.75.

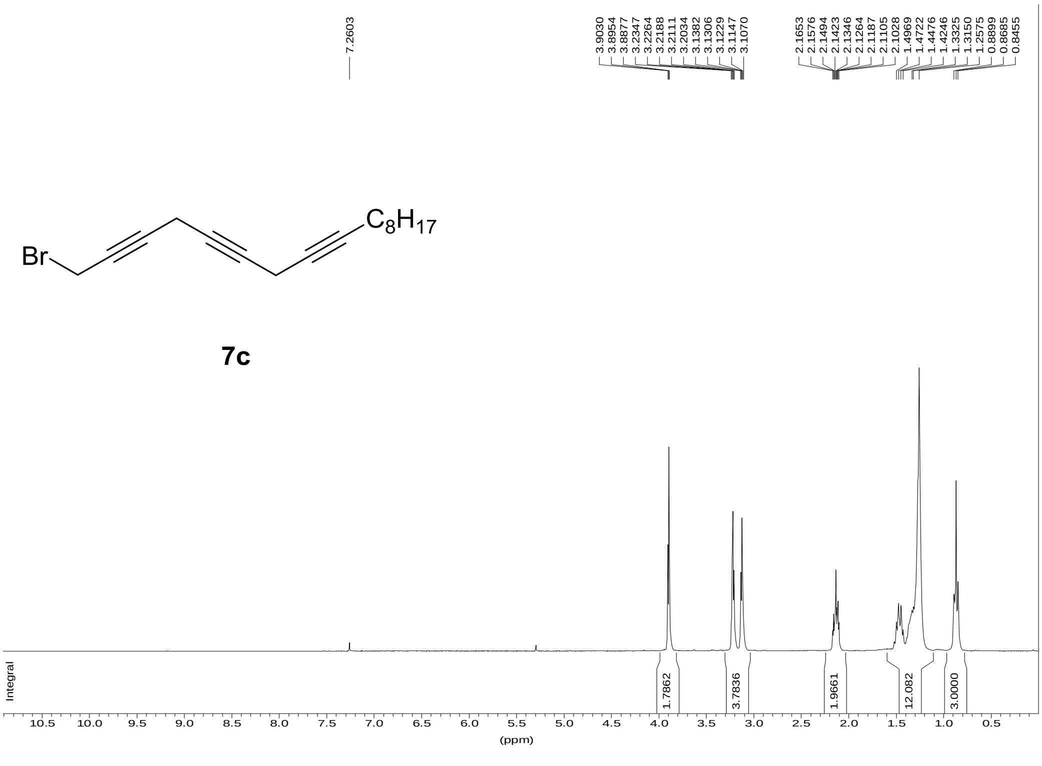

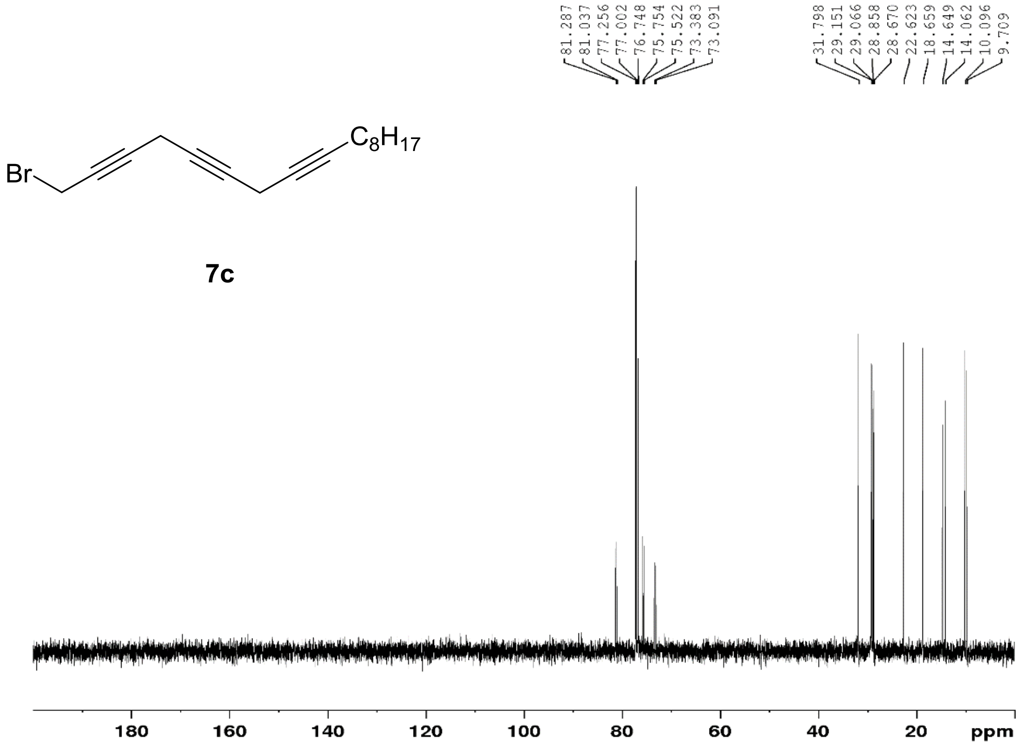

(9-bromonona-1,4,7-triyn-1-yl)benzene (7d): Yellow oil, 77% yield. ^{1}H NMR (500 MHz, $CDCl_3$) δ 7.45 – 7.43 (m, 2H), 7.30 (dd, *J* = 4.4, 2.1 Hz, 3H), 3.91 (d, *J* = 2.5 Hz, 2H), 3.40 (d, *J* = 2.5 Hz, 2H), 3.26 (d, , *J* = 2.5 Hz, 2H). ^{13}C NMR (125 MHz, $CDCl_3$) δ 131.55(2C), 128.09(2C), 127.99, 122.83, 83.19, 81.07, 80.64, 75.60, 74.74, 73.67, 14.63, 10.36, 10.02.

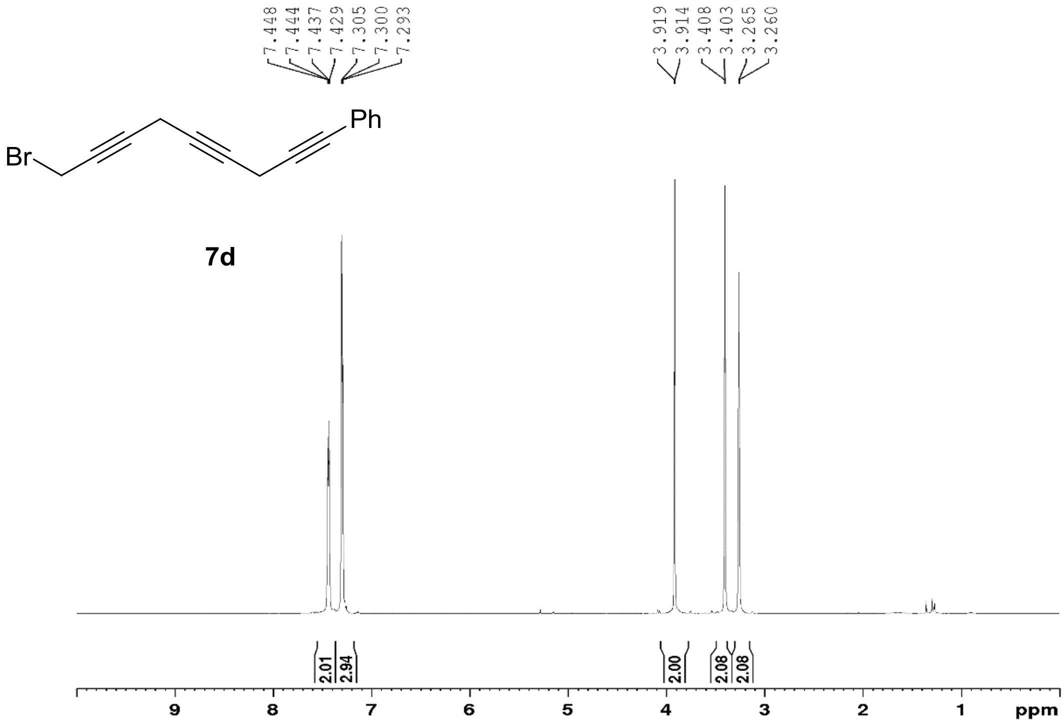

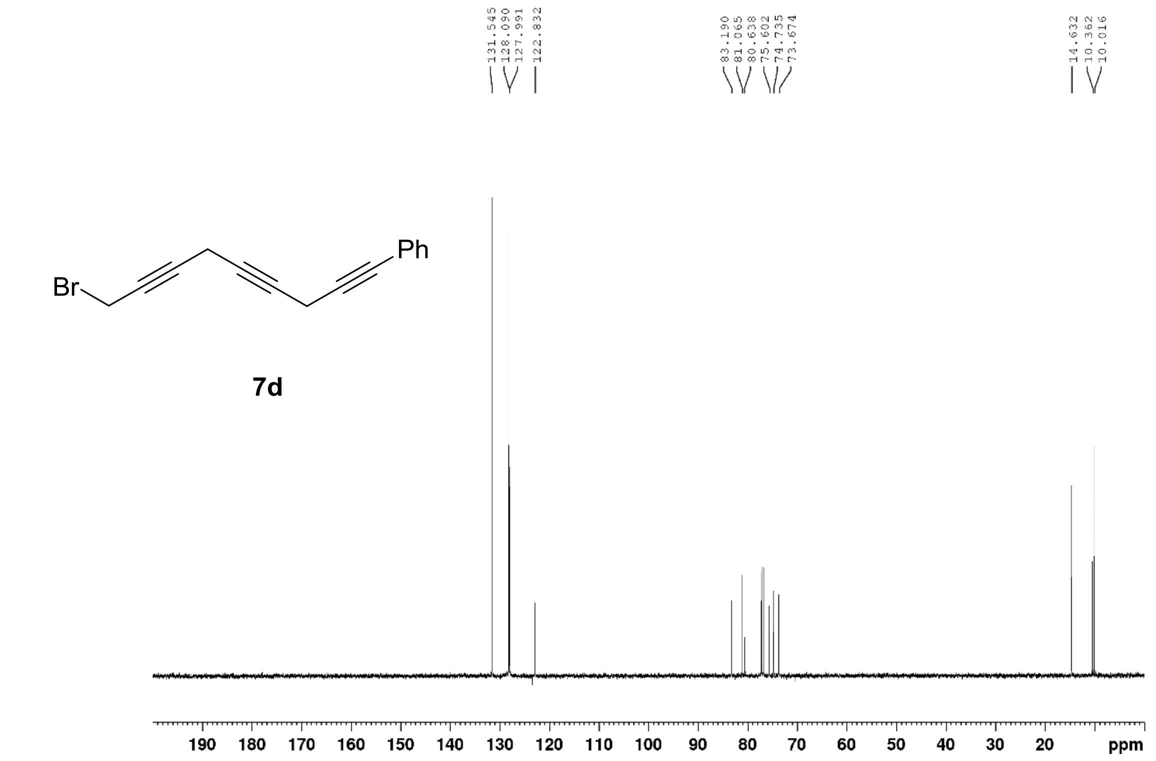

7d

1-bromopentadeca-2,5,8-triyne (7e): Yellow oil, 67% yield. ^{1}H NMR (500 MHz, $CDCl_3$) δ 3.90 (t, *J* = 2.3 Hz, 2H), 3.23 – 3.22 (m, 2H), 3.13 (d, *J* = 3.0, Hz, 2H), 2.16 – 2.13 (m, 2H), 1.50 – 1.45 (m, 2H), 1.39 – 1.31 (m, 2H), 1.30-1.25 (m, 4H), 0.88 (t, *J* = 6.9 Hz, 3H); ^{13}C NMR (125 MHz, $CDCl_3$) δ 81.14, 80.86, 75.62, 75.43, 73.28, 72.97, 31.20, 28.52, 28.41, 22.41, 18.53, 14.59, 13.89, 9.95, 9.56.

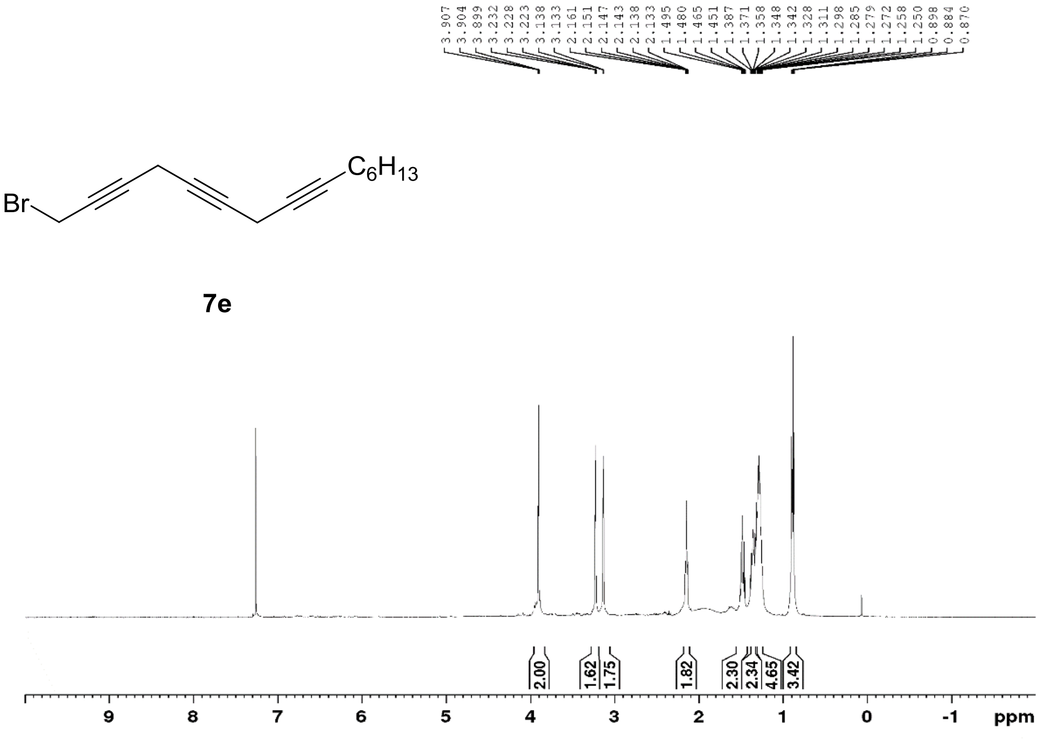

7e

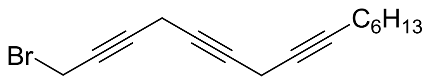

7e

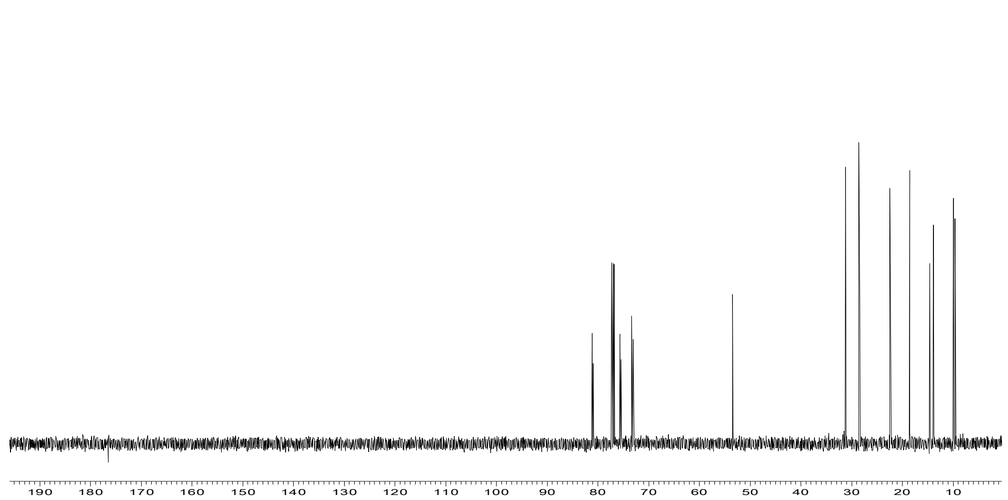

(5Z,8Z,11Z,14Z)-methyl icosa-5,8,11,14-tetraenoate (9a): Yellow oil, 58% yield (over 2 steps), eluent system: 98:2 hexane-EA, ^{1}H NMR (500 MHz, $CDCl_3$) δ 5.54 – 5.29 (m, 8 H), 3.68 (s, 3 H), 2.80 – 2.83 (m, 6 H), 2.34 (t, *J* = 7.5 Hz, 2 H), 2.13 (dd, *J* = 14.0, 7.1 Hz, 2 H), 2.07 (dd, *J* = 14.2, 7.0 Hz, 2 H), 1.79 – 1.66 (m, 2 H), 1.45 – 1.23 (m, 6 H), 0.91 (t, *J* = 6.8 Hz, 3 H); ^{13}C NMR (125 MHz, $CDCl_3$) δ 174.04, 130.48, 128.92, 128.87, 128.57, 128.20, 128.16, 127.86, 127.54, 51.47, 33.43, 31.52, 29.33, 27.22, 26.55, 25.64, 25.63, 25.61, 24.78, 22.58, 14.07.

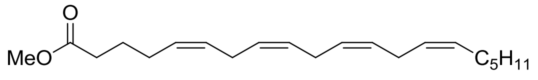

9a

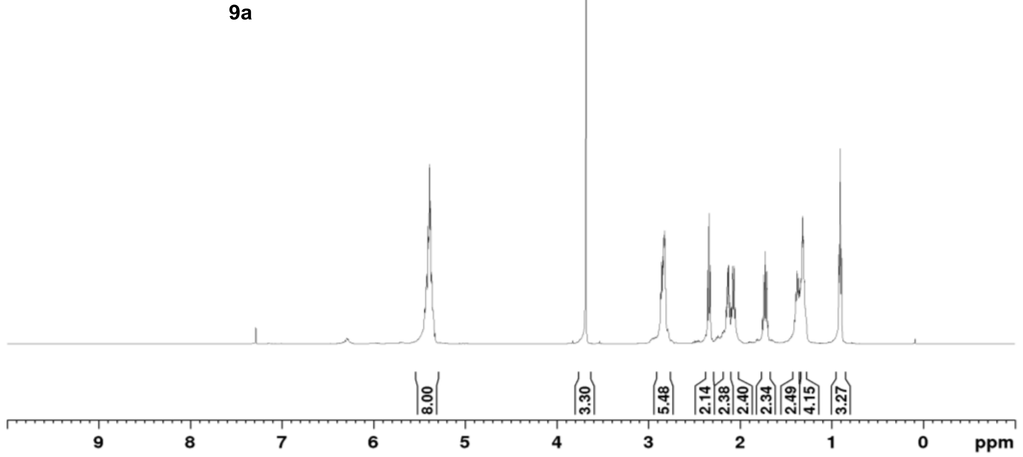

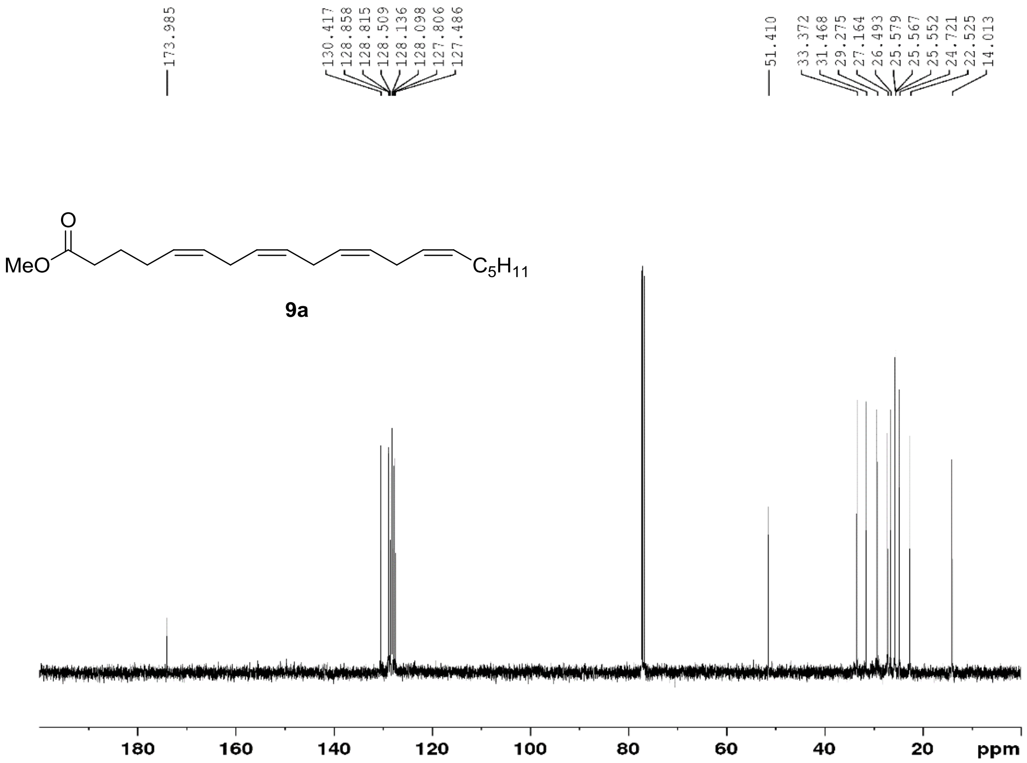

(5Z,8Z,11Z,14Z)-methyl octadeca-5,8,11,14-tetraenoate (9b): Yellow oil, 48% yield (over 2 steps), eluent system: 98:2 hexane-EA, ^{1}H NMR (500 MHz, $CDCl_3$) δ 5.67 – 5.18 (m, 8H), 3.67 (s, 3H), 2.82 (dt, *J* = 17.1, 5.7 Hz, 6H), 2.32 (t, *J* = 7.5 Hz, 2H), 2.08 (ddd, *J* = 33.9, 13.9, 6.9 Hz, 4H), 1.83 – 1.62 (m, 2H), 1.39 (dd, *J* = 14.8, 7.4 Hz, 2H), 0.91 (t, *J* = 7.4 Hz, 3H). ^{13}C NMR (75 MHz, $CDCl_3$) δ174.05, 130.21, 128.92, 128.88, 128.58, 128.20, 128.17, 127.87, 127.77, 51.46, 33.45, 29.30, 26.56, 25.65, 25.63, 25.61, 24.79, 22.77, 13.77.

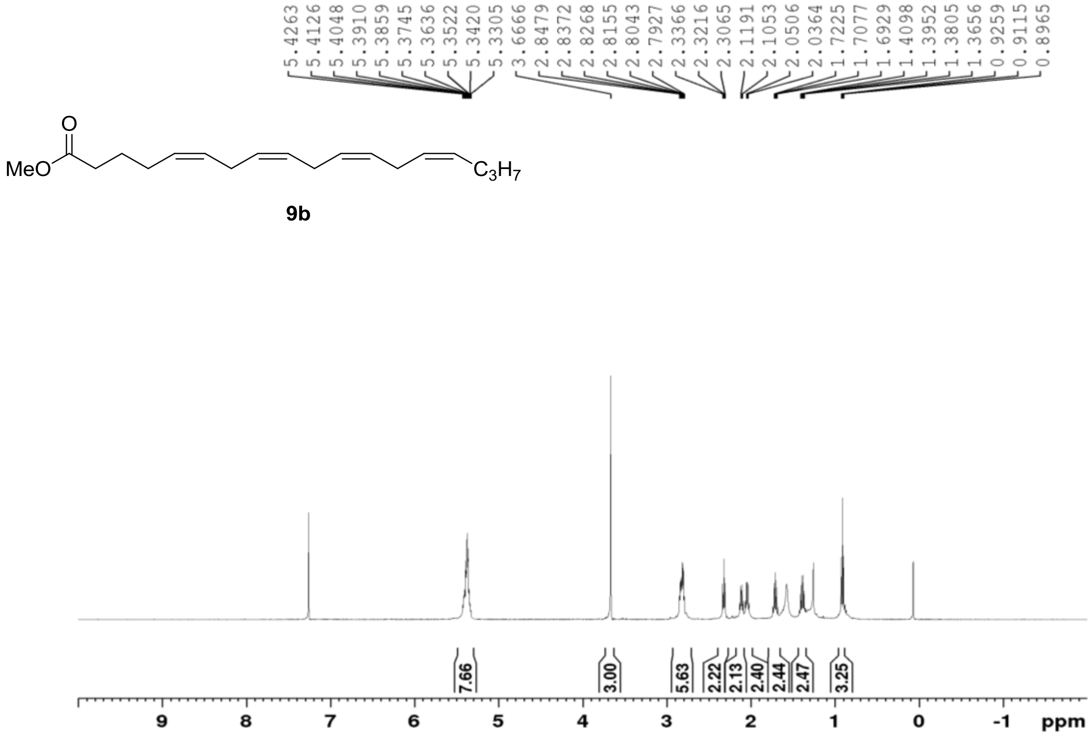

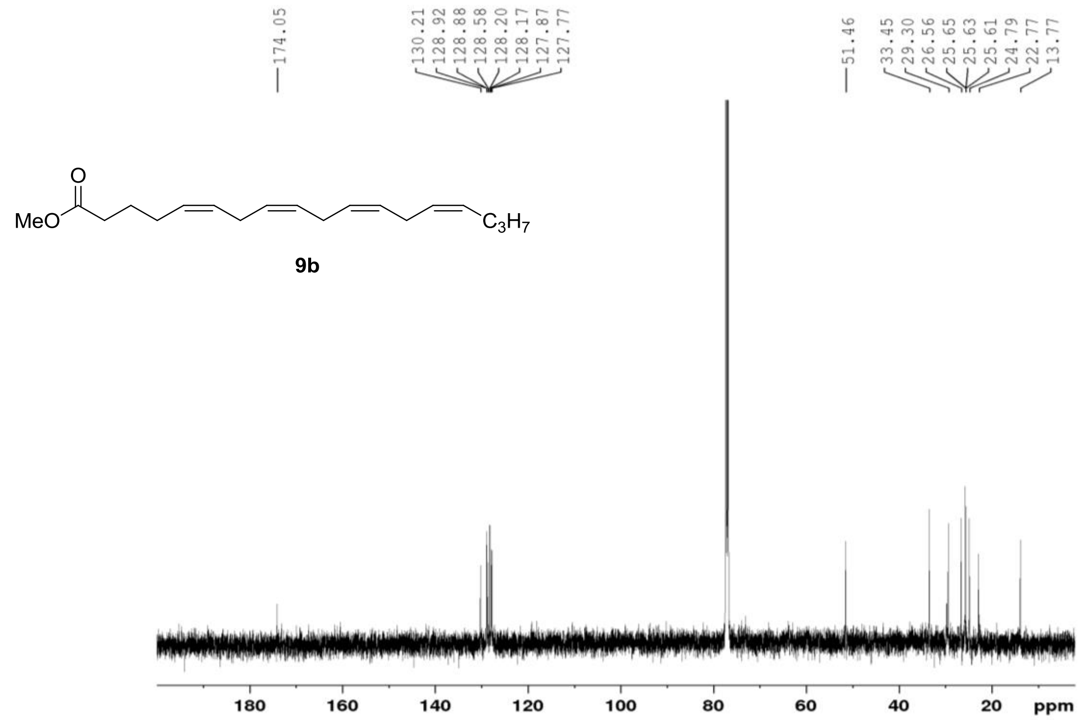

(5Z,8Z,11Z,14Z)-methyl tricosa-5,8,11,14-tetraenoate (9c): Yellow oil, 50% yield (over 2 steps), eluent system: 98:2 hexane-EA, ^{1}H NMR (500 MHz, $CDCl_3$) δ 5.50 – 5.28 (m, 8 H), 3.69 (s, 3 H), 2.85 (dt, *J* = 10.3, 5.5 Hz, 6 H), 2.35 (dd, *J* = 9.5, 5.6 Hz, 2 H), 2.11 (ddd, *J* = 28.7, 14.1, 7.0 Hz, 6 H), 1.79 – 1.66 (m, 2 H), 1.44 – 1.24 (m, 10 H), 0.91 (t, *J* = 6.7 Hz, 1H); ^{13}C NMR (75 MHz, $CDCl_3$) δ 173.91, 130.39, 128.84, 128.81, 128.50, 128.12, 128.08, 127.79, 127.47, 51.35, 33.35, 31.84, 29.60, 29.46, 29.25, 27.20, 27.05, 26.49, 25.55, 24.72, 22.61, 14.02.

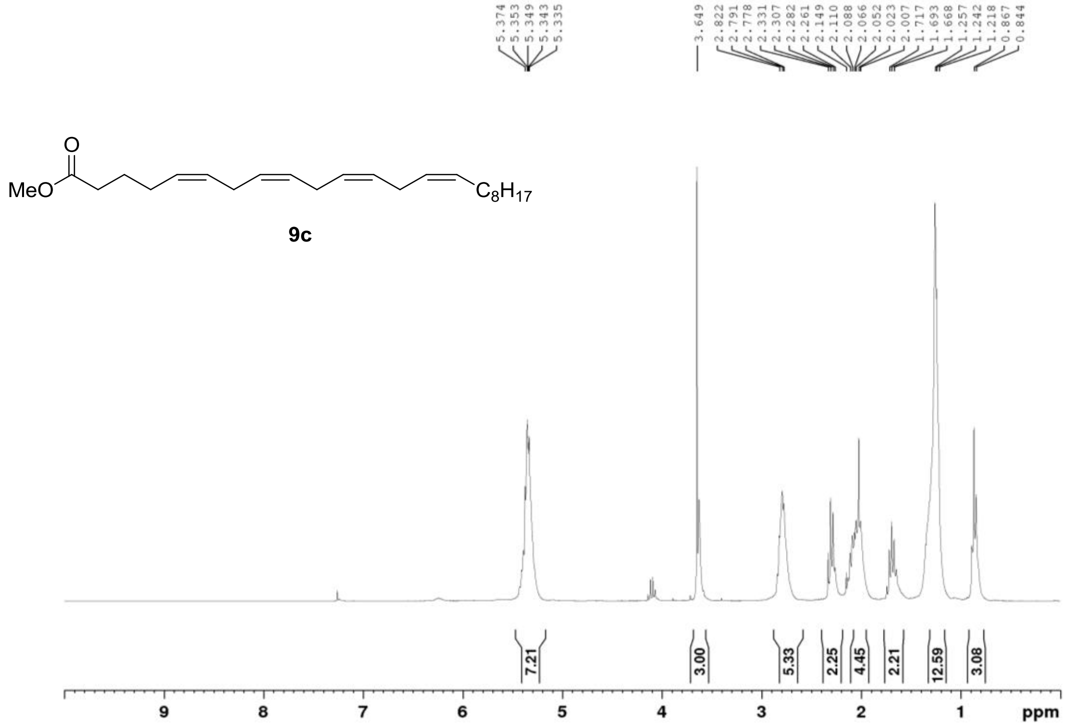

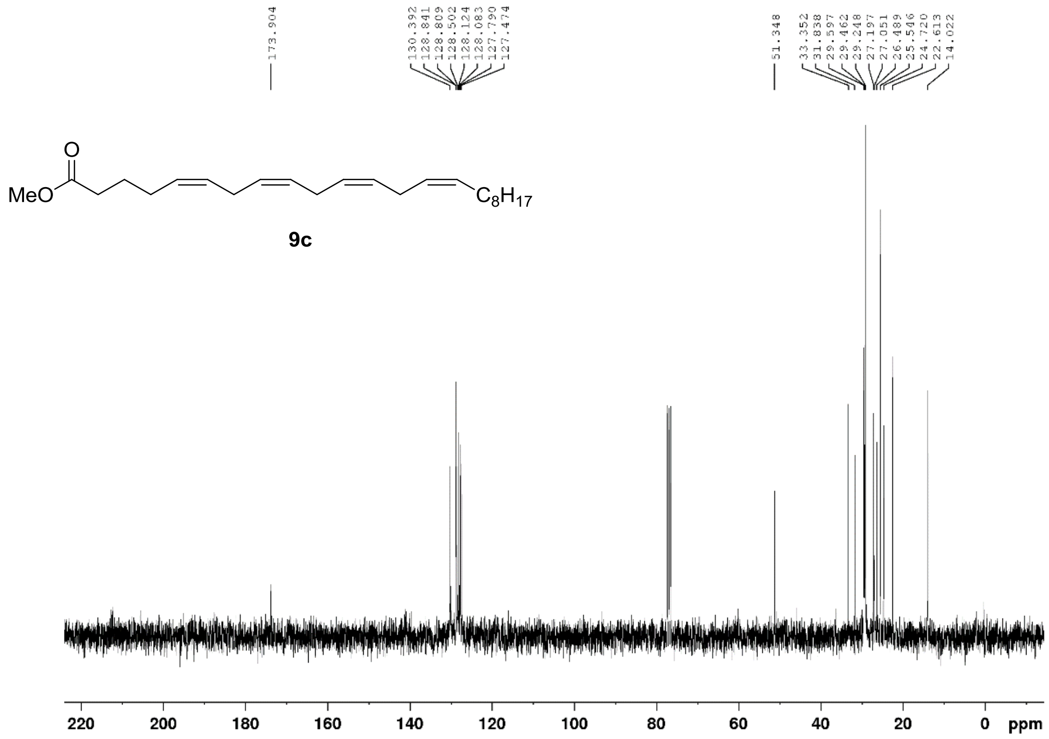

9c

(5Z,8Z,11Z,14Z)-methyl 15-phenylpentadeca-5,8,11,14-tetraenoate (9d): Yellow oil, 46% yield (over 2 steps), eluent system: 98:2 hexane-EA, ^{1}H NMR (500 MHz, $CDCl_3$) δ 7.37 (t, *J* = 7.6 Hz, 2H), 7.32 (d, *J* = 7.4 Hz, 2H), 7.29 – 7.21 (m, 1H) , 6.51 (d, *J* = 11.4 Hz, 1H), 5.69 (dd, *J* = 7.4, 4.1 Hz, 1H), 5.53 – 5.44 (m, 2H), 5.43 – 5.36 (m, 4H), 3.70 (s, 3H), 3.14 (t, *J* = 6.9 Hz, 2H), 2.84 (t, *J* = 5.8 Hz, 2H), 2.80 (t, *J* = 5.2 Hz, 2H), 2.35 (dd, *J* = 9.4, 5.5 Hz, 2H), 2.13 (dd, *J* = 13.3, 6.7 Hz, 2H), 1.77 – 1.71 (m, 2H); ^{13}C NMR (125 MHz, $CDCl_3$) δ173.92, 137.31, 130.53, 129.27, 128.87, 128.77, 128.70, 128.66, 128.23, 128.106, 128.00, 127.90, 126.60, 51.63, 33.63, 26.91, 26.49, 25.67, 25.54, 24.72.

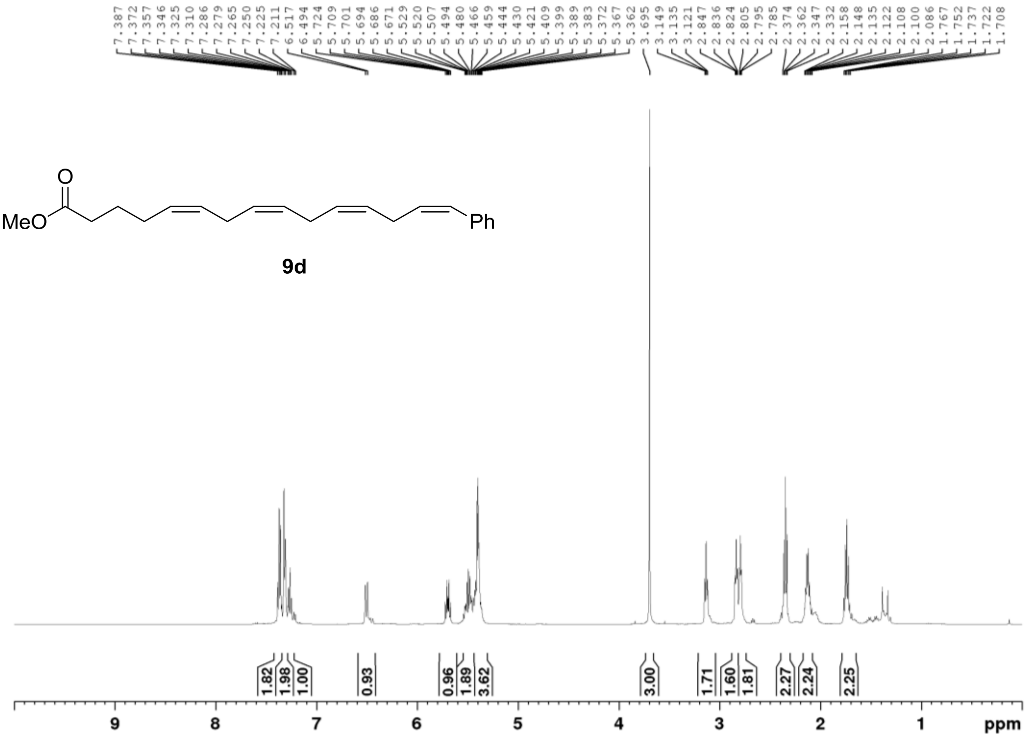

9d

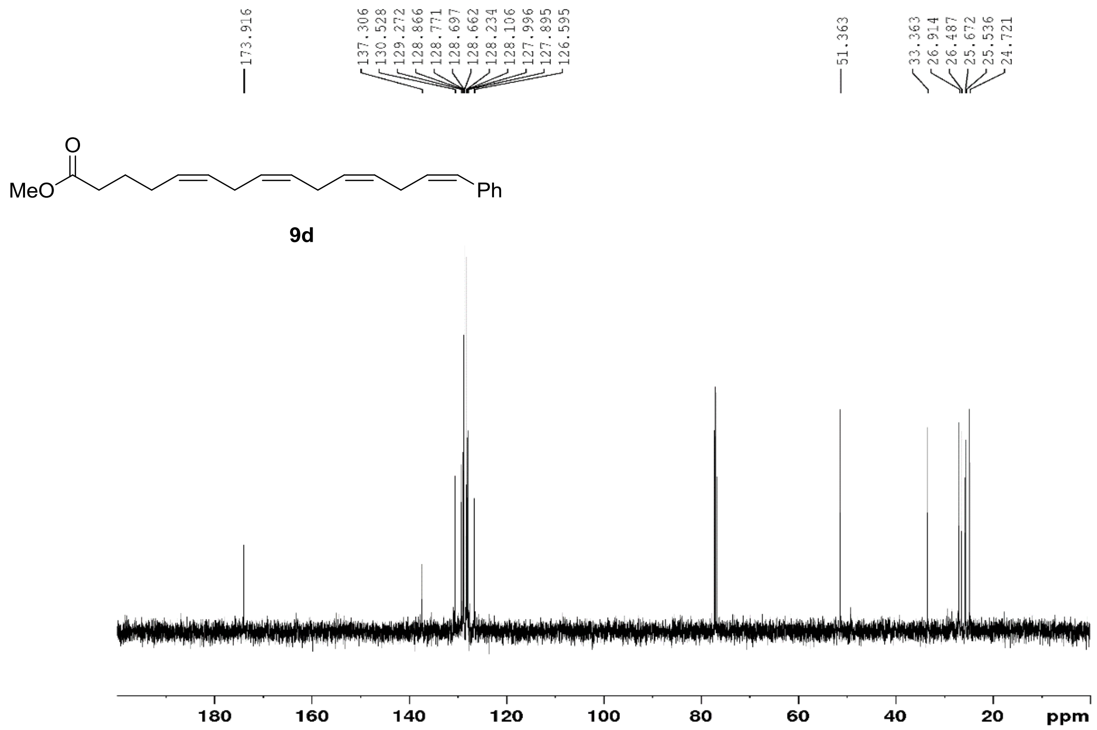

(4Z,7Z,10Z,13Z)-propyl icosa-4,7,10,13-tetraenoate (9e): Yellow oil, 37% yield (over 2 steps), eluent system: 98:2 hexane-EA, ^{1}H NMR (500 MHz, $CDCl_3$) δ 5.41 – 5.37 (m, 8H), 4.05 (dd, *J* = 8.7, 4.7 Hz, 2H), 2.86 – 2.79 (m, 6H), 2.42 – 2.37 (m, 4H), 2.09 – 2.05 (m, 2H), 1.67 (dd, *J* = 14.2, 7.0 Hz, 2H), 1.37 – 1.30 (m, 8H), 0.96 (t, *J* = 7.4 Hz, 3H), 0.90 (t, *J* = 6.8 Hz, 3H). ^{13}C NMR (125 MHz, $CDCl_3$) δ 173.13, 130.42, 129.20, 128.55, 128.28, 127.98, 127.94, 127.80, 127.50, 65.94, 34.22, 31.73, 29.58, 28.94, 27.22, 25.59, 25.55, 22.81, 22.60, 21.96, 14.04, 10.34.

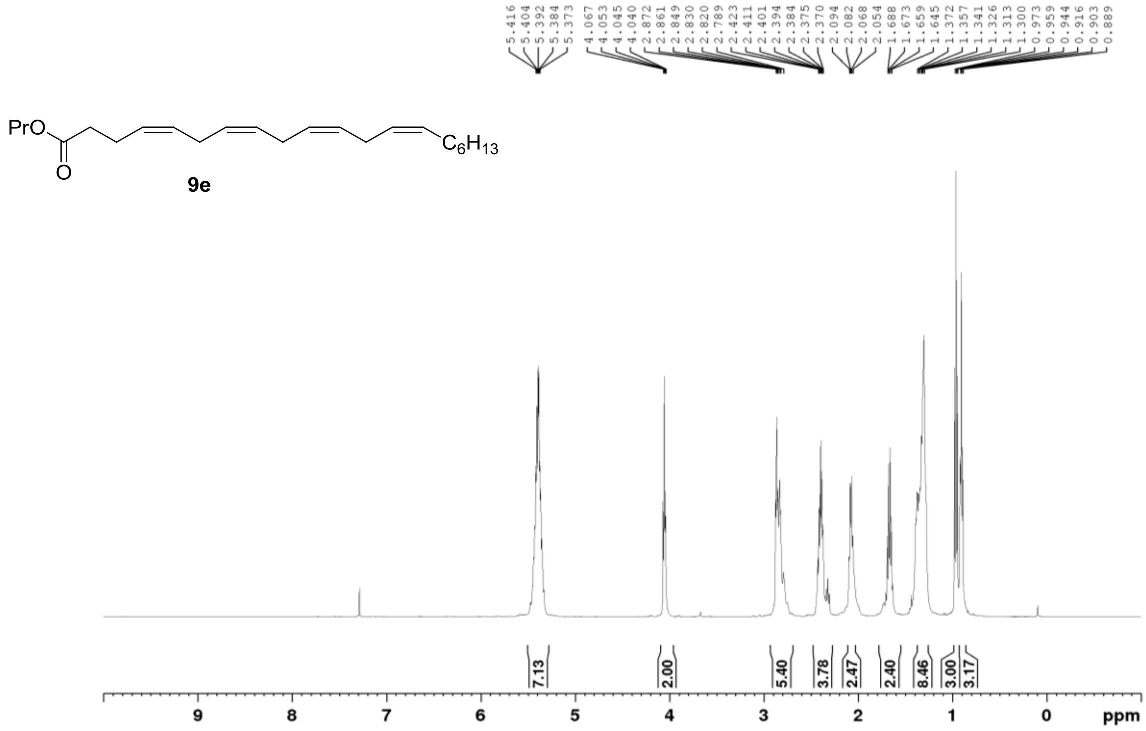

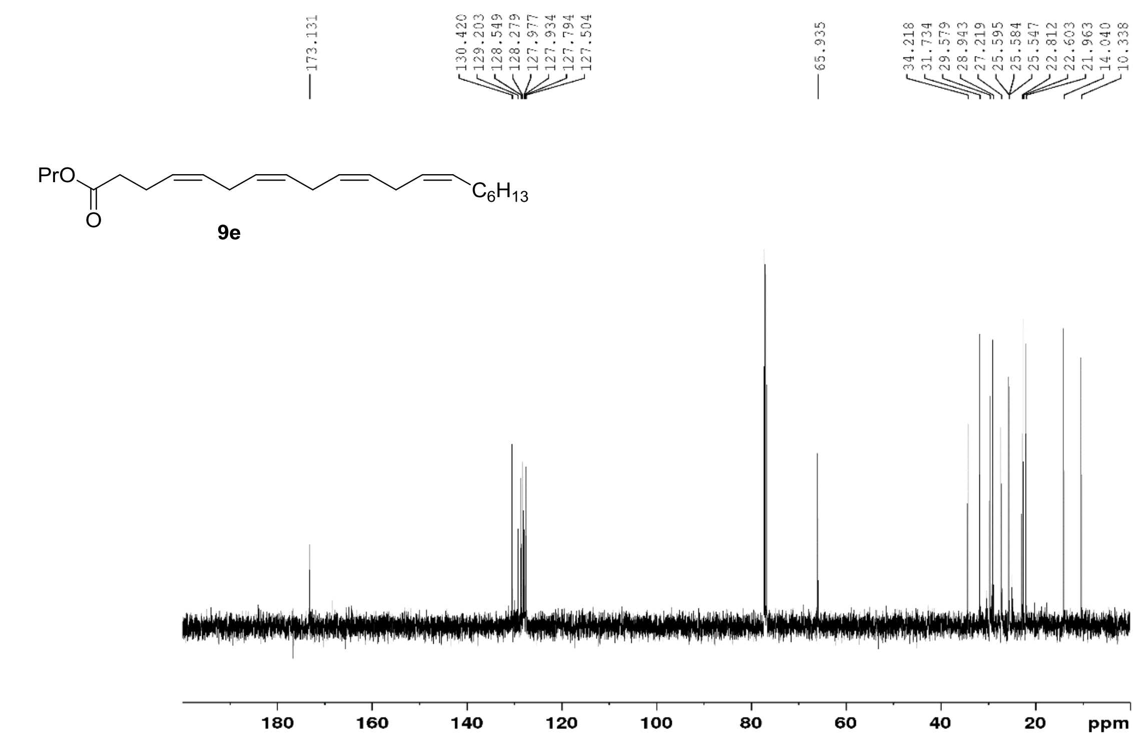

(5Z,8Z,11Z)-methyl heptadeca-5,8,11-trienoate (11a): Yellow oil, 52% yield (over 2 steps), eluent system: 98:2 hexane-EA, ^{1}H NMR (300 MHz, $CDCl_3$) δ 5.50 – 5.21 (m, 6 H), 3.65 (s, 3 H), 2.79 (t, *J* = 5.4 Hz, 2 H), 2.31 (t, *J* = 7.5 Hz, 2 H), 2.19 – 1.90 (m, 4 H), 1.69 (dt, *J* = 14.4, 7.2 Hz, 2 H), 1.29 (s, 8 H), 0.88 (t, *J* = 6.4 Hz, 3 H); ^{13}C NMR (75 MHz, $CDCl_3$) δ 173.93, 130.36, 128.86, 128.75, 128.39, 127.83, 127.46, 51.34, 33.33, 31.42, 29.22, 27.77, 27.12, 26.45, 25.53, 24.69, 22.47, 13.95.

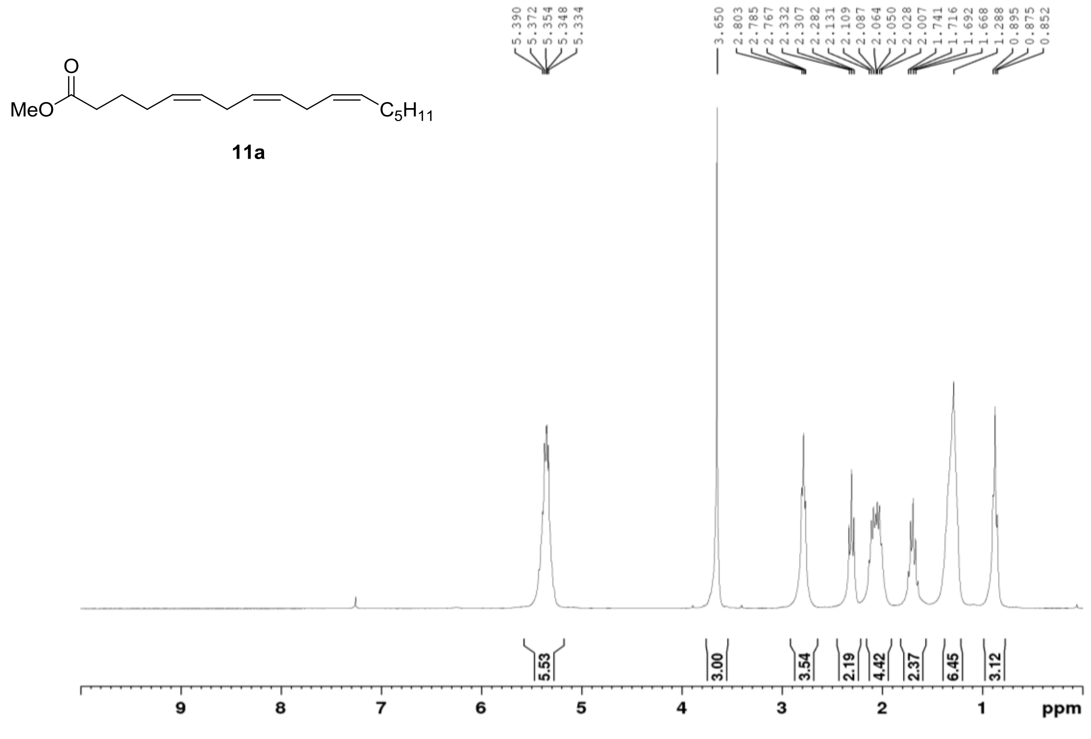

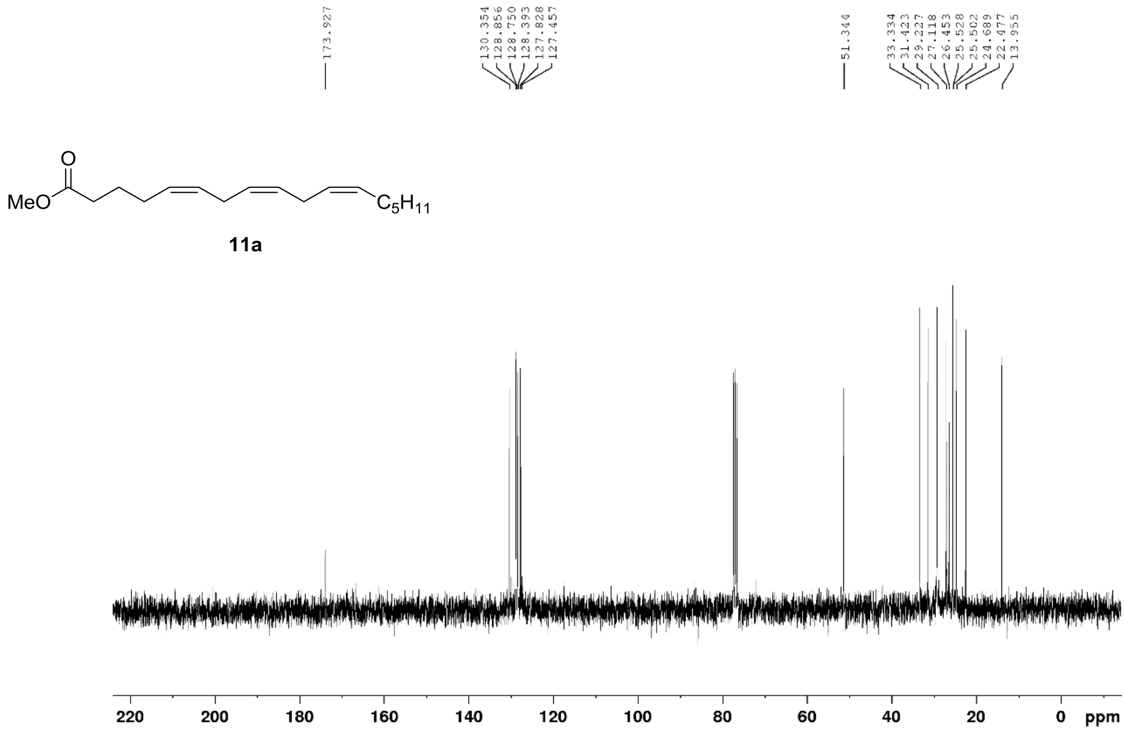

(5Z,8Z,11Z)-methyl pentadeca-5,8,11-trienoate (11b): Yellow oil, 30% yield (over 2 steps), eluent system: 98:2 hexane-EA, ^{1}H NMR (300 MHz, $CDCl_3$) δ 5.44 – 5.38 (m, 6H), 3.70 (s, 3H), 2.82 (d, *J* = 5.3 Hz, 4H), 2.36 (t, *J* = 7.5 Hz, 4H), 2.16 – 2.04 (m, 2H), 1.76 – 1.72 (m, 2H), 1.42 (dd, *J* = 14.7, 7.3 Hz, 2H), 0.94 (t, *J* = 7.3 Hz, 3H). ^{13}C NMR (75 MHz, $CDCl_3$) δ 174.02, 130.14, 128.90, 128.81, 128.45, 127.88, 127.73, 51.42, 33.39, 29.25, 26.50, 25.59, 25.55, 24.74, 22.73, 13.74.

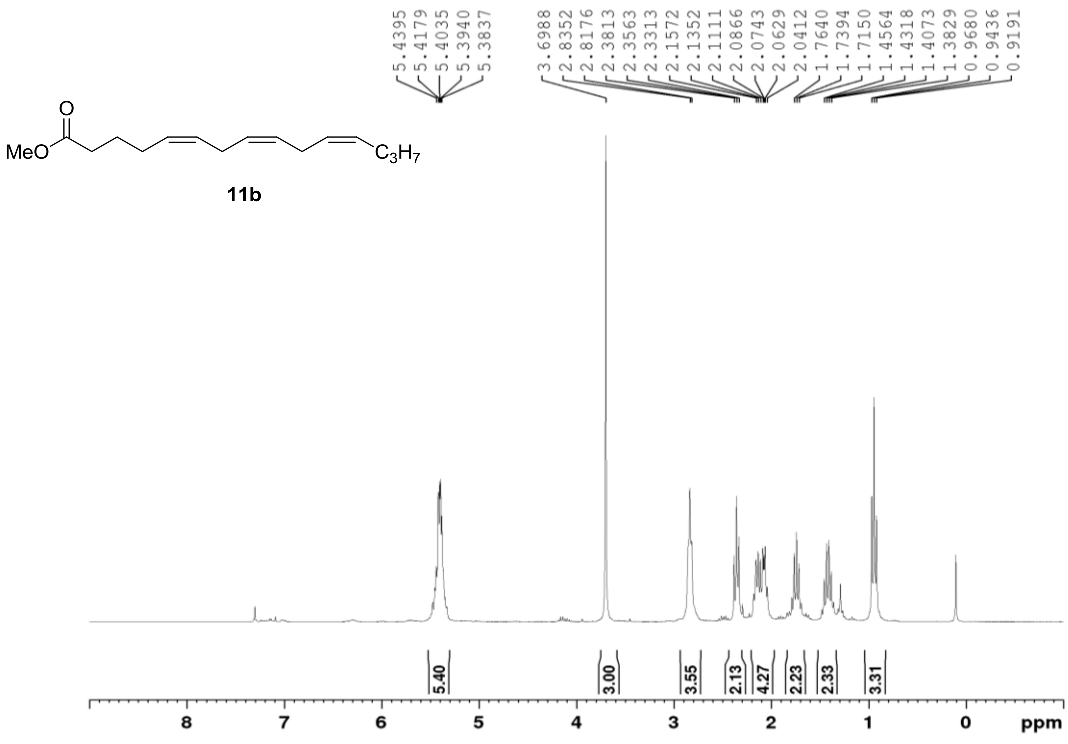

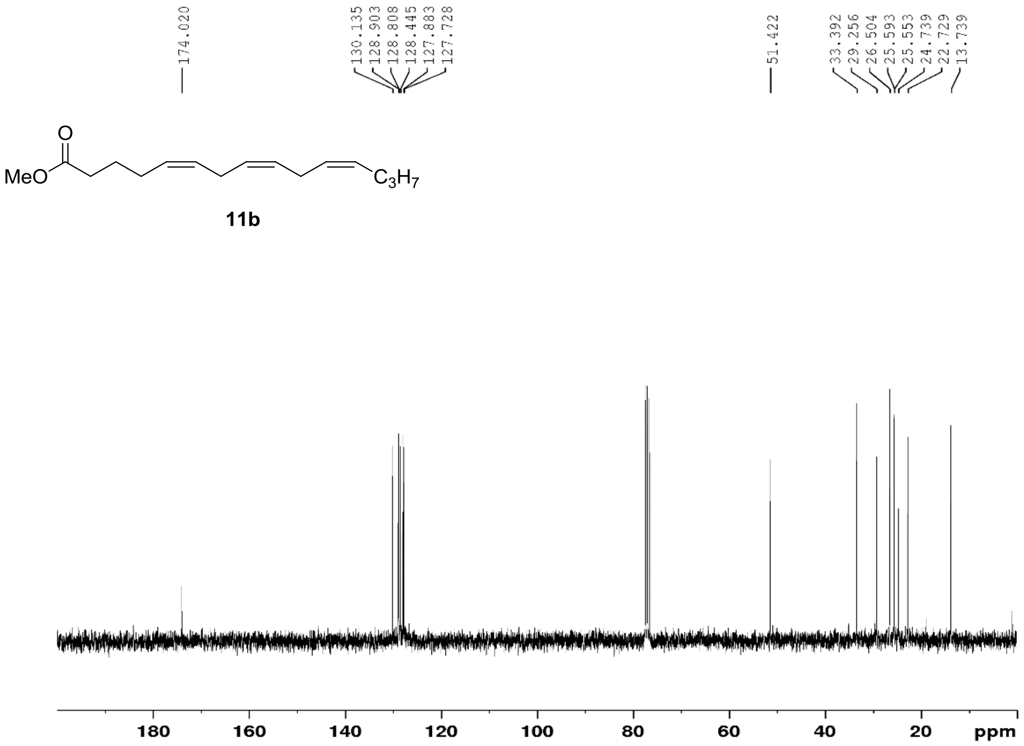

(5Z,8Z,11Z)-methyl icosa-5,8,11-trienoate (11c): Yellow oil, 40% yield (over 2 steps), eluent system: 98:2 hexane-EA, ^{1}H NMR (300 MHz, $CDCl_3$) δ 5.46 – 5.22 (m, 5H), 3.65 (s, 3H), 2.78 (t, *J* = 5.4 Hz, 1H), 2.31 (t, *J* = 7.5 Hz, 1H), 2.17 – 1.91 (m, 2H), 1.76 – 1.59 (m, 1H), 1.26 (s, 5H), 0.86 (t, *J* = 6.3 Hz, 1H); ^{13}C NMR (75 MHz, $CDCl_3$) δ 173.95, 130.38, 128.88, 128.77, 128.41, 127.84, 127.46, 51.37, 33.34, 31.83, 29.59, 29.46, 29.25 (2 C), 27.19, 26.47, 25.52 (2 C), 24.70, 22.61, 14.03.

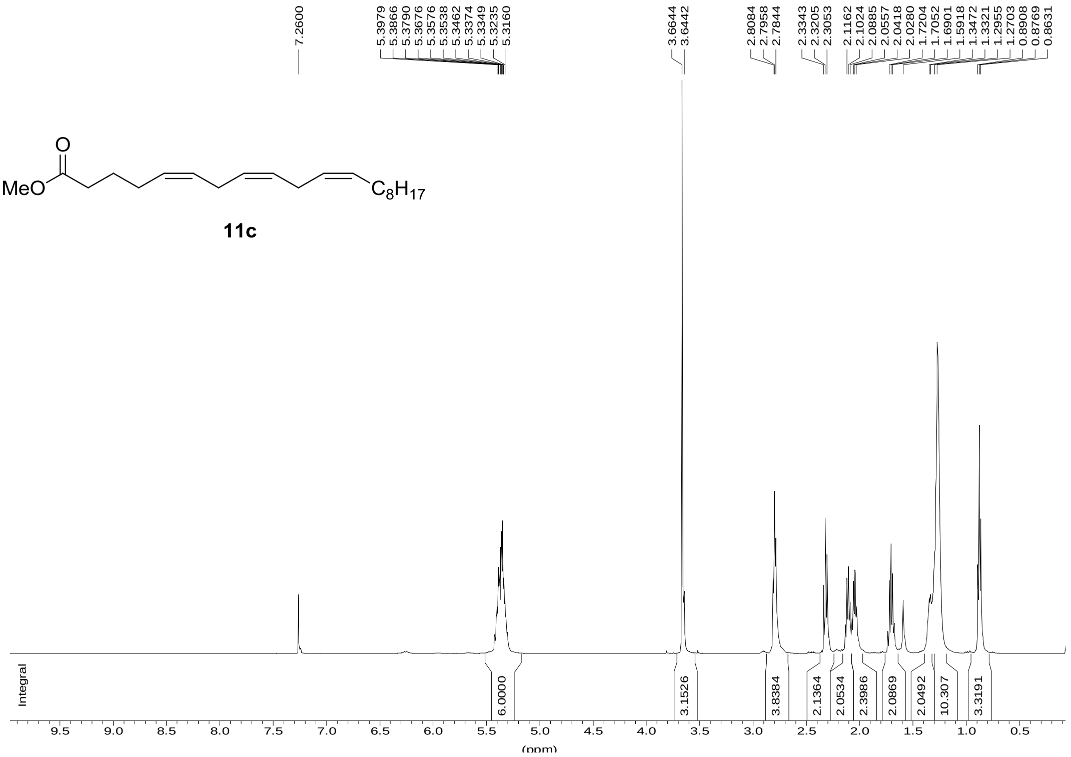

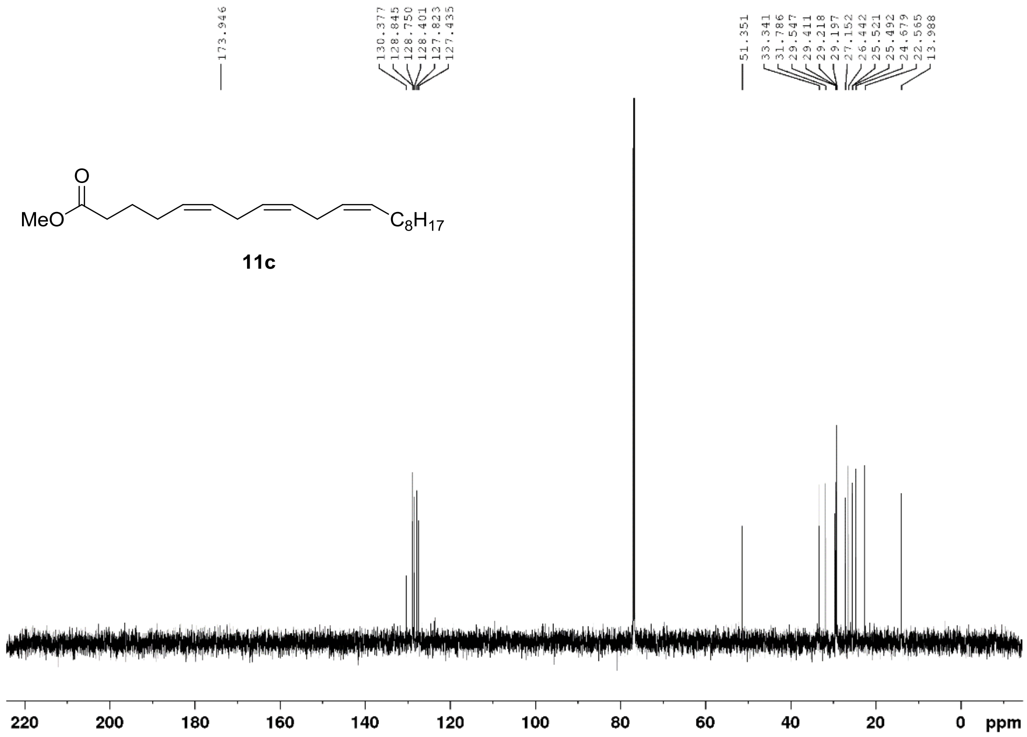

Oct-2-yn-1-ol (13a): Colourless oil, 47% yield. ^{1}H NMR (300 MHz, $CDCl_3$) δ 4.24 (t, *J* = 2.1 Hz, 2H), 2.61 (s, 1H), 2.20 (tt, *J* = 7.2, 2.1 Hz, 2H), 1.49 (dd, *J* = 14.2, 7.1 Hz, 2H), 1.41 – 1.28 (m, 4H), 0.89 (t, *J* = 6.5 Hz, 3H). ^{13}C NMR (75 MHz, $CDCl_3$) δ 86.21, 78.21, 50.98, 30.90, 28.18, 22.05, 18.55, 13.78.

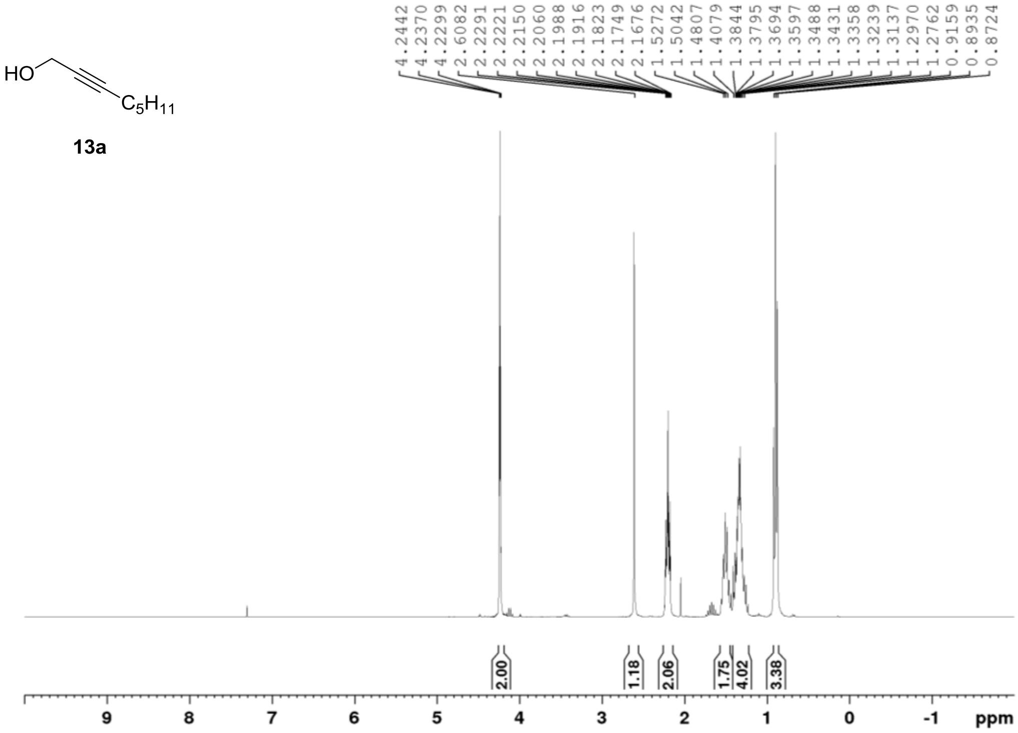

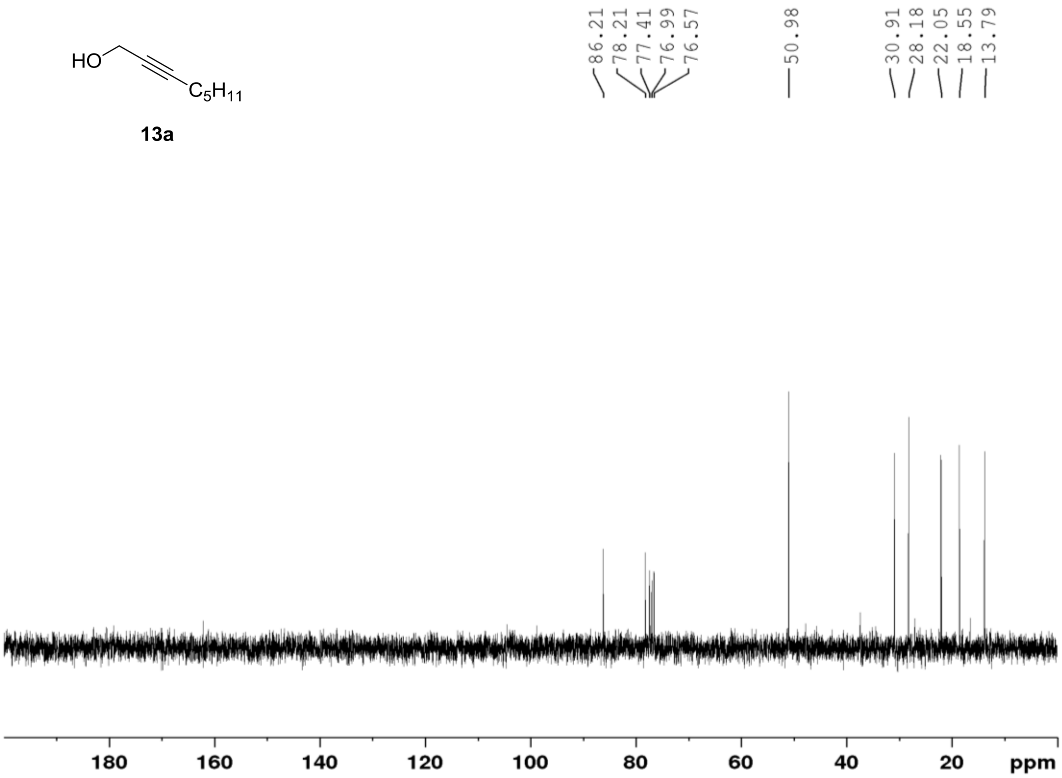

13a

Dec-2-yn-1-ol (13b): Colourless oil, 86% yield. ^{1}H NMR (500 MHz, $CDCl_3$) δ 4.21 (s, 2 H), 2.29 (s, 1 H), 2.24 (tt, *J* = 7.2, 1.9 Hz, 2 H), 1.52 – 1.41 (m, 2 H), 1.37 (dt, *J* = 15.3, 6.8 Hz, 2 H), 1.32 – 1.19 (m, 6 H), 0.85 (t, *J* = 6.9 Hz, 3 H); ^{13}C NMR (125 MHz, $CDCl_3$) δ 87.06, 78.96, 51.81, 32.34, 29.45, 29.41, 29.25, 23.23, 19.34, 14.64.

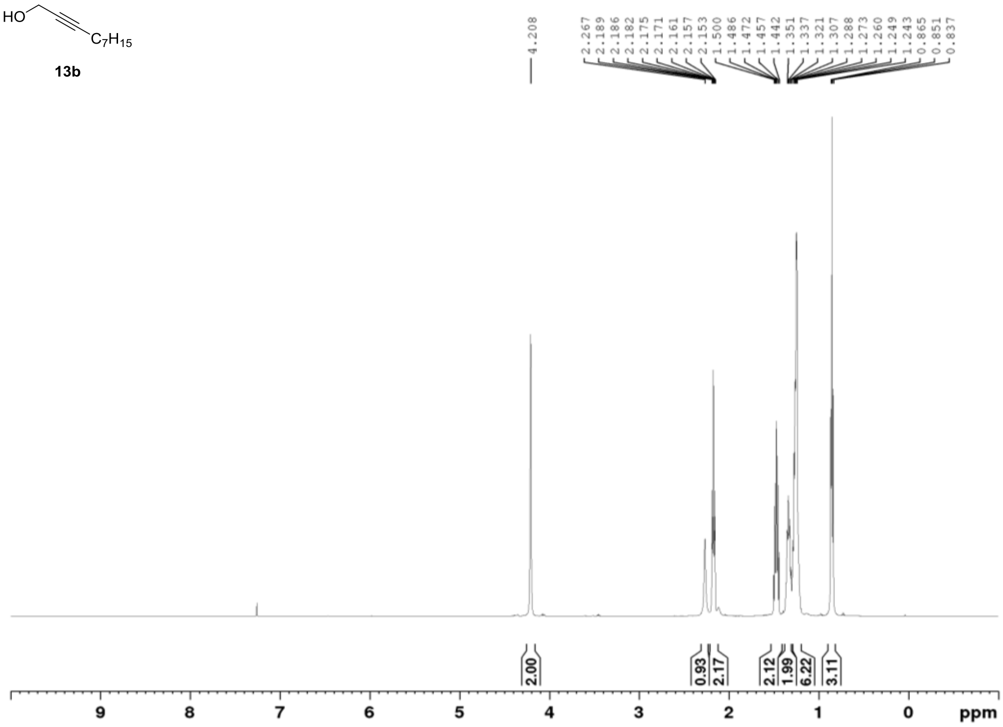

13b

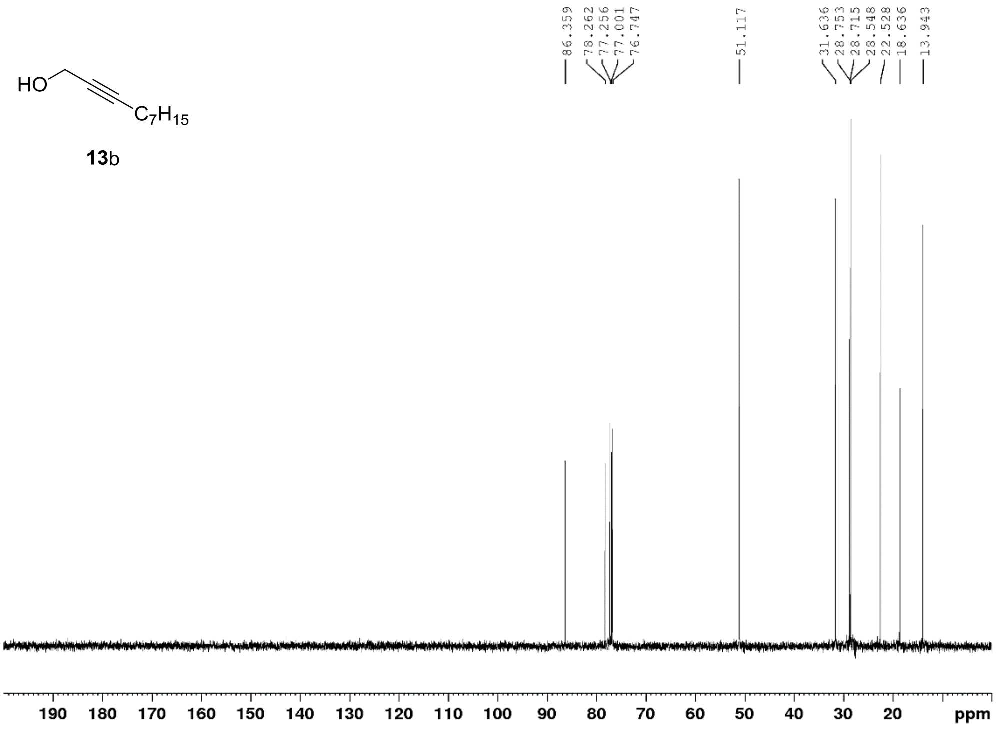

13b

Tetradec-2-yn-1-ol (13c): White solid, 50% yield. ^{1}H NMR (500 MHz, $CDCl_3$) δ 4.22 (s, 2 H), 2.18 (ddd, *J* = 9.2, 4.8, 2.0 Hz, 2 H), 1.89 (d, *J* = 5.4 Hz, 1 H), 1.48 (dd, *J* = 14.4, 7.1 Hz, 2 H), 1.24 (m, 16 H), 0.86 (t, *J* = 6.7 Hz, 3H); ^{13}C NMR (125 MHz, $CDCl_3$) δ 86.55, 78.31, 51.31, 31.90, 29.62, 29.61, 29.51, 29.33, 29.14, 28.88, 28.62, 22.67, 18.72, 14.07.

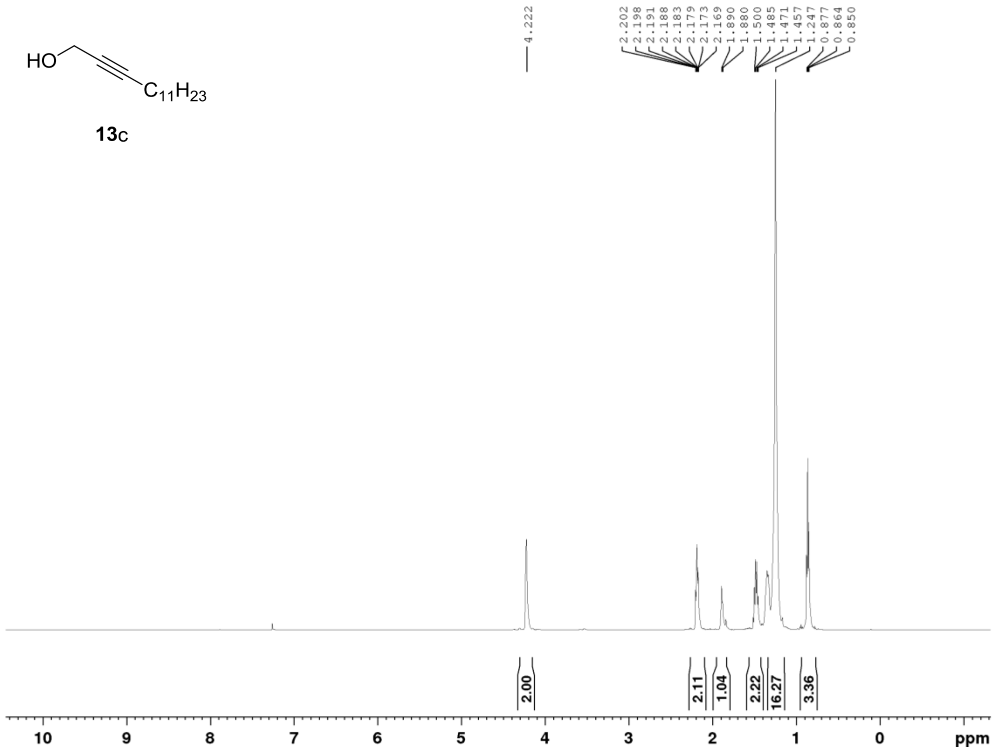

13c

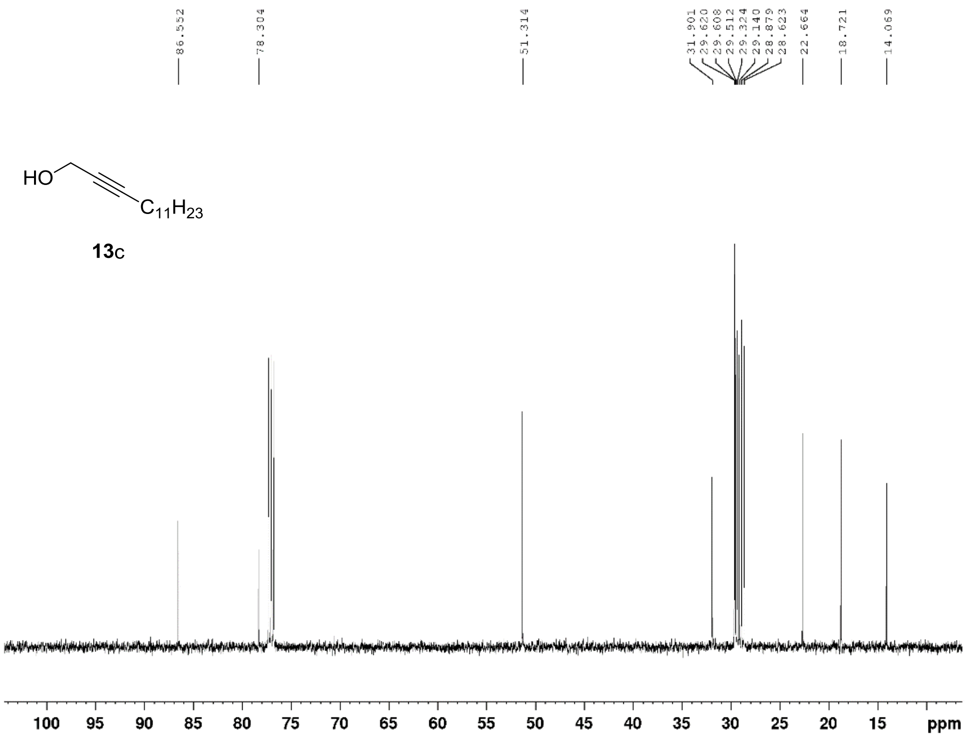

Hexadec-2-yn-1-ol (13d): White solid, 65% yield. ^{1}H NMR (500 MHz, $CDCl_3$) δ 4.22 (s, 2 H), 2.18 (tt, *J* = 7.1, 2.1 Hz, 2 H), 2.09 (s, 1 H), 1.48 (dt, *J* = 15.1, 7.1 Hz, 2 H), 1.40 – 1.17 (m, 20 H), 0.86 (t, *J* = 7.0 Hz, 3 H); ^{13}C NMR (125 MHz, $CDCl_3$) δ 86.49, 78.31, 51.26, 31.92, 29.68, 29.66 (2 C), 29.65, 29.53, 29.35, 29.16, 28.89, 28.63, 22.68, 18.72, 14.08.

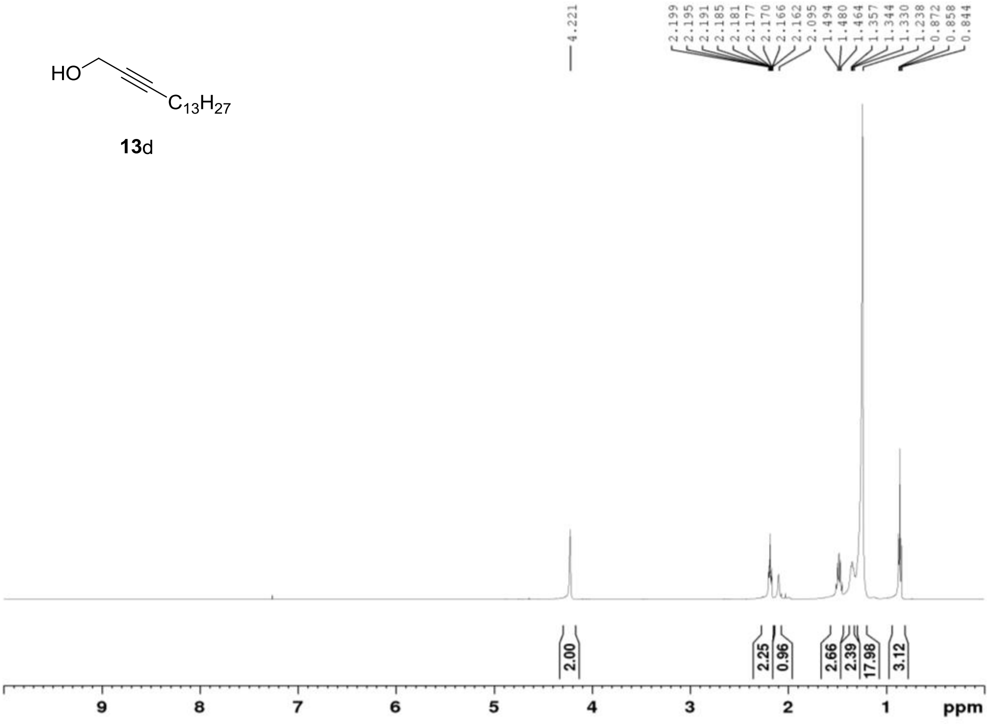

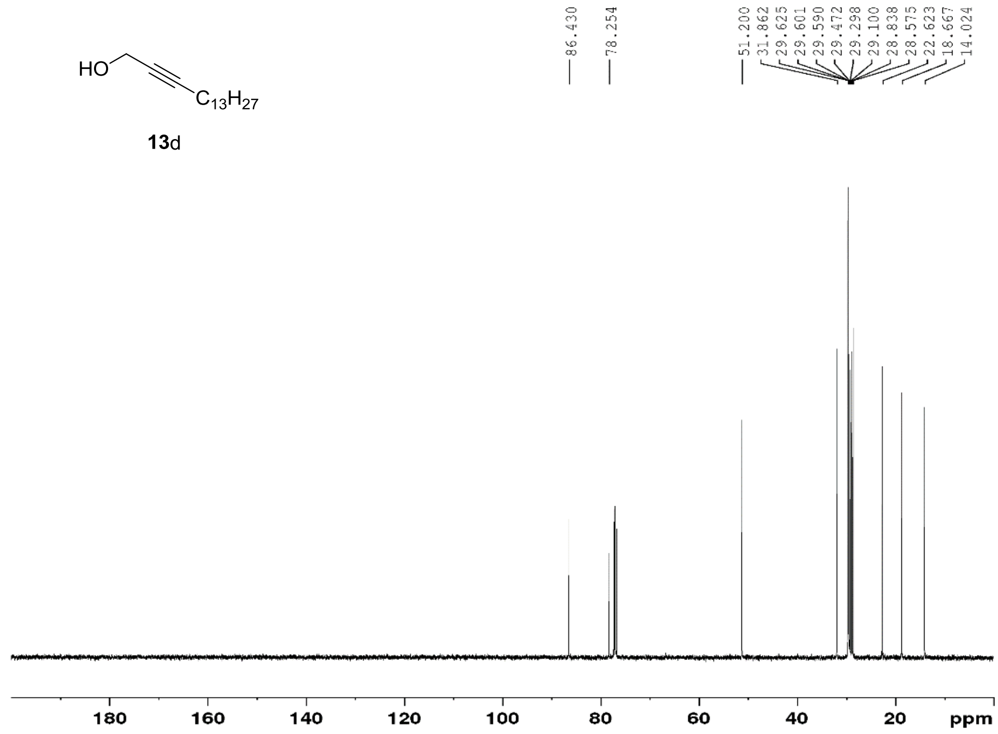

1-bromooct-2-yne (14a): Colourless oil, 97% yield. ^{1}H NMR (500 MHz, $CDCl_3$) δ 4.34 (t, *J* = 2.3 Hz, 2H), 2.65 (ddt, *J* = 9.3, 4.7, 2.2 Hz, 2H), 1.94 – 1.91 (m, 2H), 1.77 – 1.72 (m, 4H), 1.31 (t, *J* = 7.1 Hz, 5H). ^{13}C NMR (125 MHz, $CDCl_3$) δ 88.28, 75.23, 30.92, 27.97, 22.09, 18.87, 15.76, 13.93.

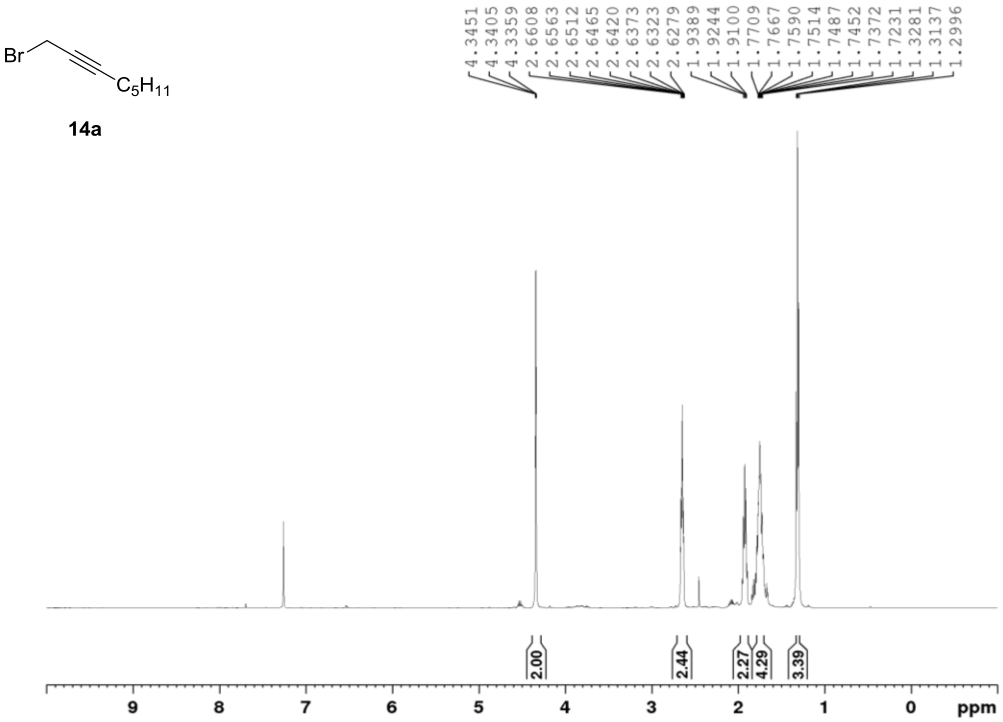

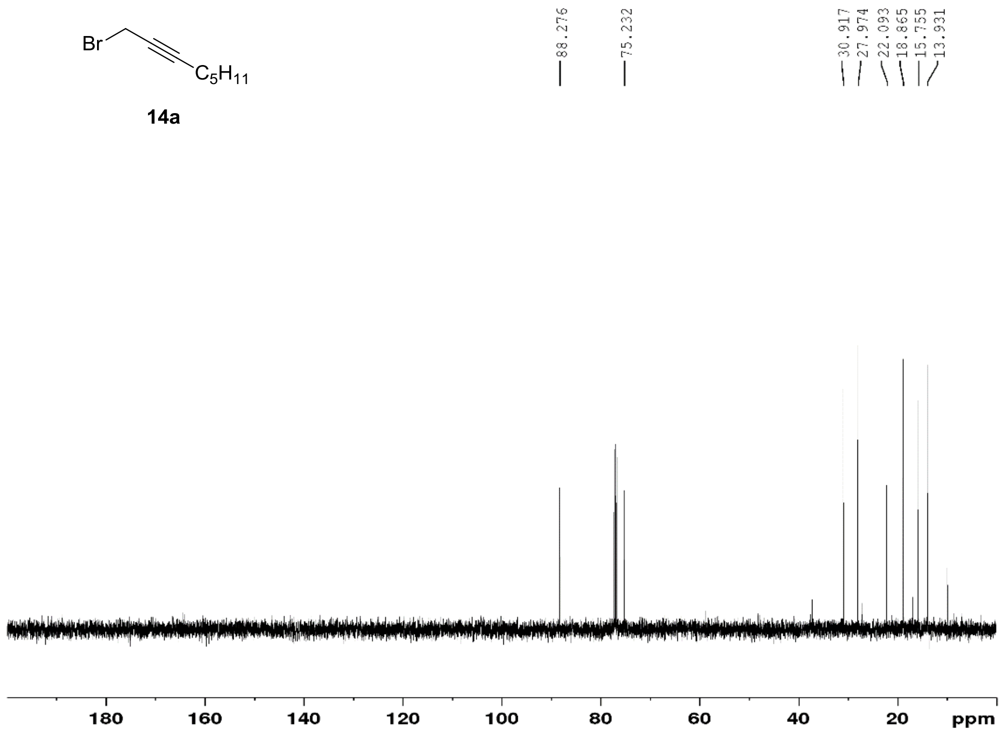

14a

1-bromodec-2-yne (14b): Colourless oil, quantitative yield. ^{1}H NMR (500 MHz, $CDCl_3$) δ 3.94 (t, *J* = 2.4 Hz, 2 H), 2.24 (tt, *J* = 7.2, 2.3 Hz, 2 H), 1.56 – 1.46 (m, 2 H), 1.38 (dt, *J* = 15.3, 6.8 Hz, 2 H), 1.34 – 1.23 (m, 6 H), 0.90 (t, *J* = 6.9 Hz, 3 H); ^{13}C NMR (125 MHz, $CDCl_3$) δ 88.32, 75.27, 31.72, 28.80, 28.78, 28.39, 22.63, 18.96, 15.78, 14.09.

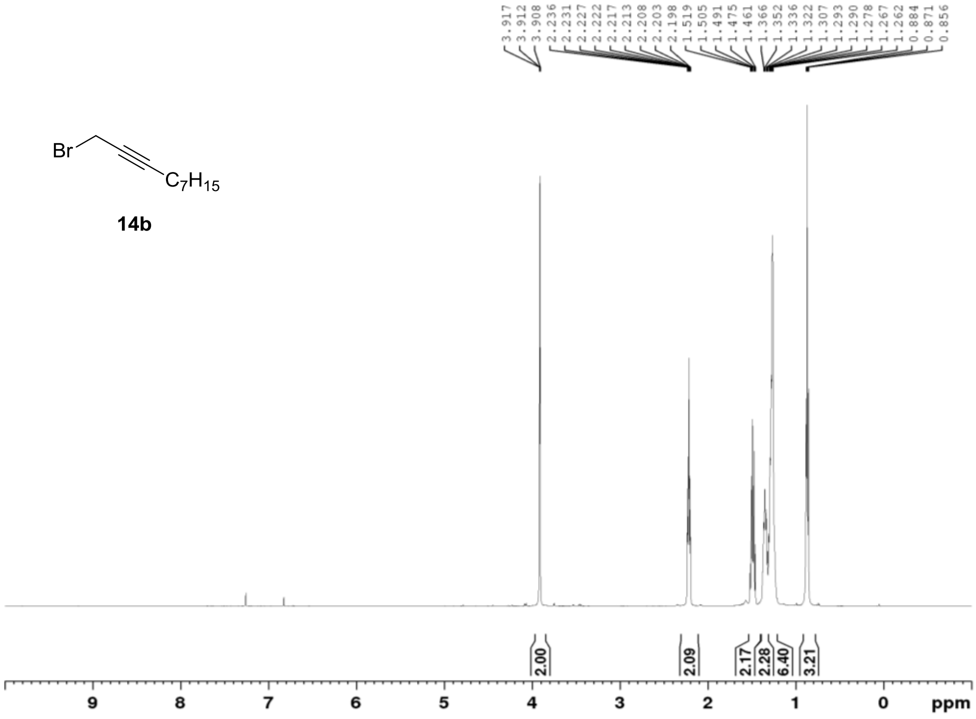

14b

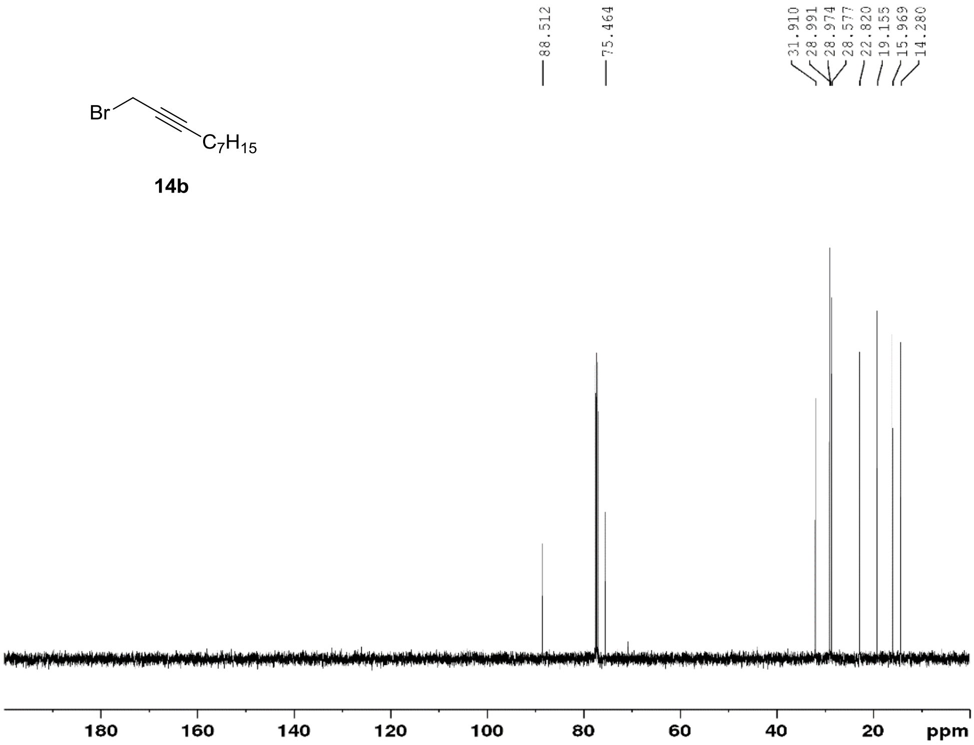

14b

1-bromotetradec-2-yne (14c): Colourless oil, quantitative yield. ^{1}H NMR (500 MHz, $CDCl_3$) δ 3.94 (s, 2 H), 2.24 (tt, J = 7.1, 2.3 Hz, 2 H), 1.56 – 1.47 (m, 2 H), 1.42 – 1.34 (m, 2 H), 1.30 (d, J = 24.3 Hz, 14 H), 0.89 (t, J = 6.9 Hz, 3 H); ^{13}C NMR (125 MHz, $CDCl_3$) δ 88.31, 75.29, 31.94, 29.64 (2 C), 29.53, 29.38, 29.13, 28.86, 28.40, 22.72, 18.98, 15.73, 14.15.

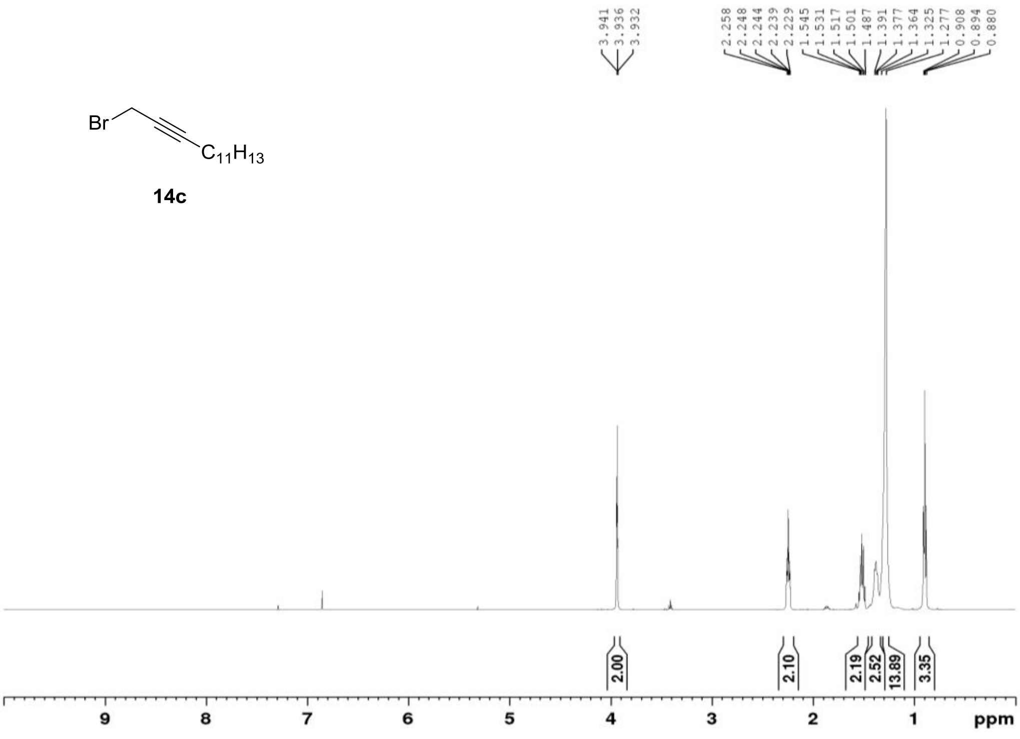

14c

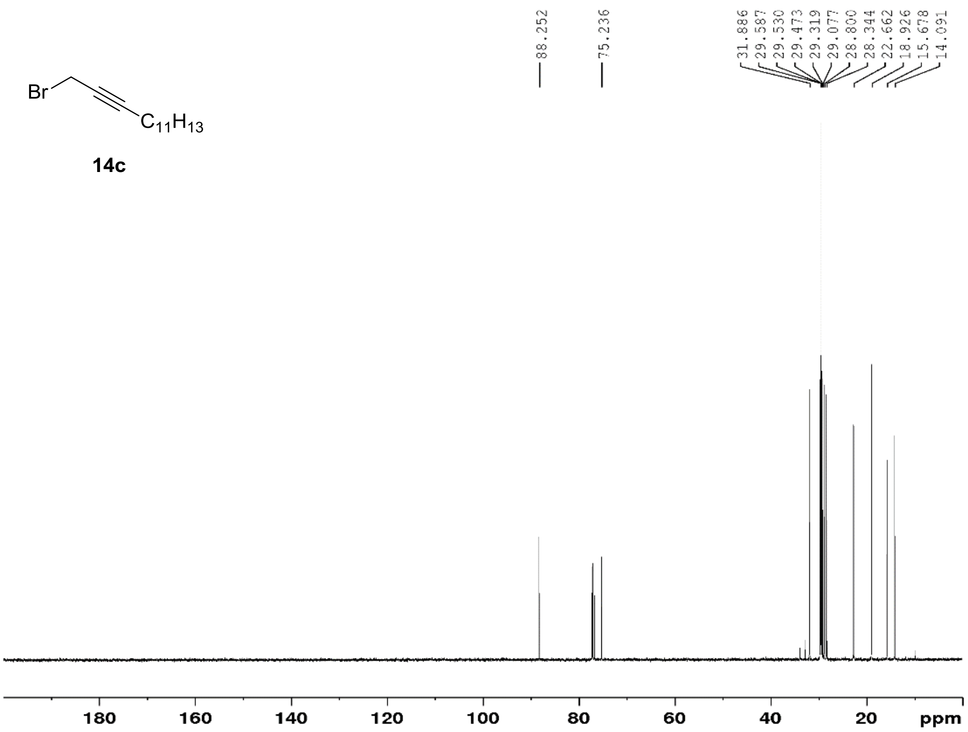

1-bromohexadec-2-yne (14d): Colourless oil, quantitative yield. ^{1}H NMR (300 MHz, $CDCl_3$) δ 3.96 (t, *J* = 2.2 Hz, 2 H), 2.27 (tt, *J* = 7.0, 2.2 Hz, 2 H), 1.62 – 1.46 (m, 2 H), 1.46 – 1.16 (m, 20 H), 0.92 (t, *J* = 6.6 Hz, 3 H); ^{13}C NMR (75 MHz, $CDCl_3$) δ 88.24, 75.17, 31.85, 29.61 (2 C), 29.58, 29.53, 29.42, 29.28, 29.02, 28.75, 28.30, 22.61, 18.87, 15.64, 14.03.

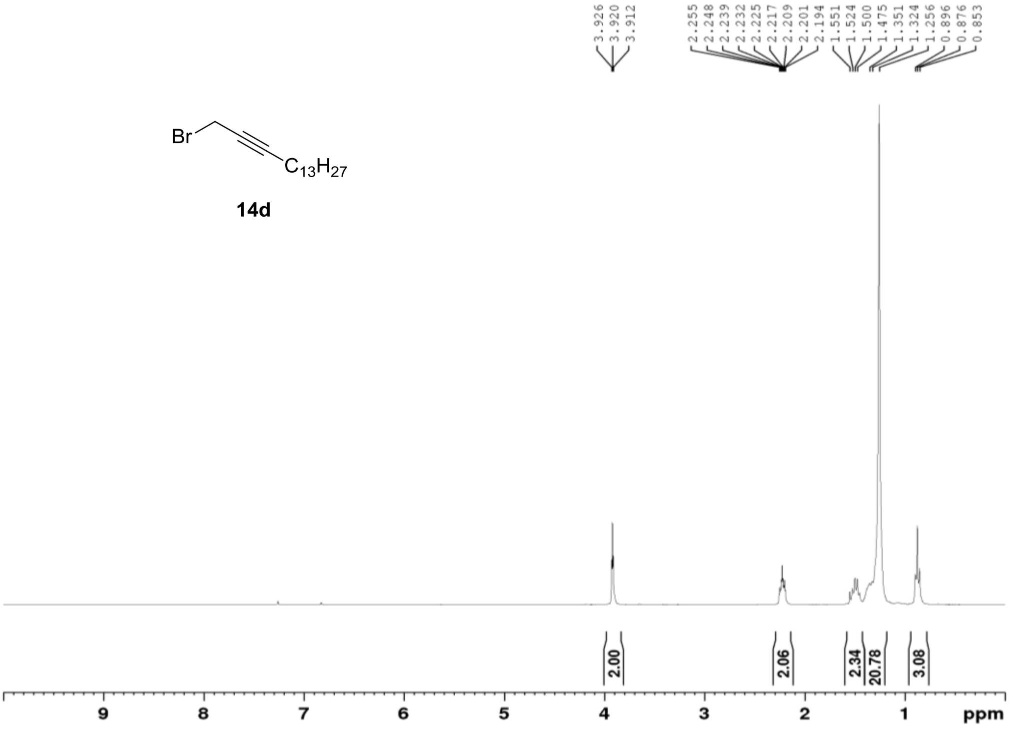

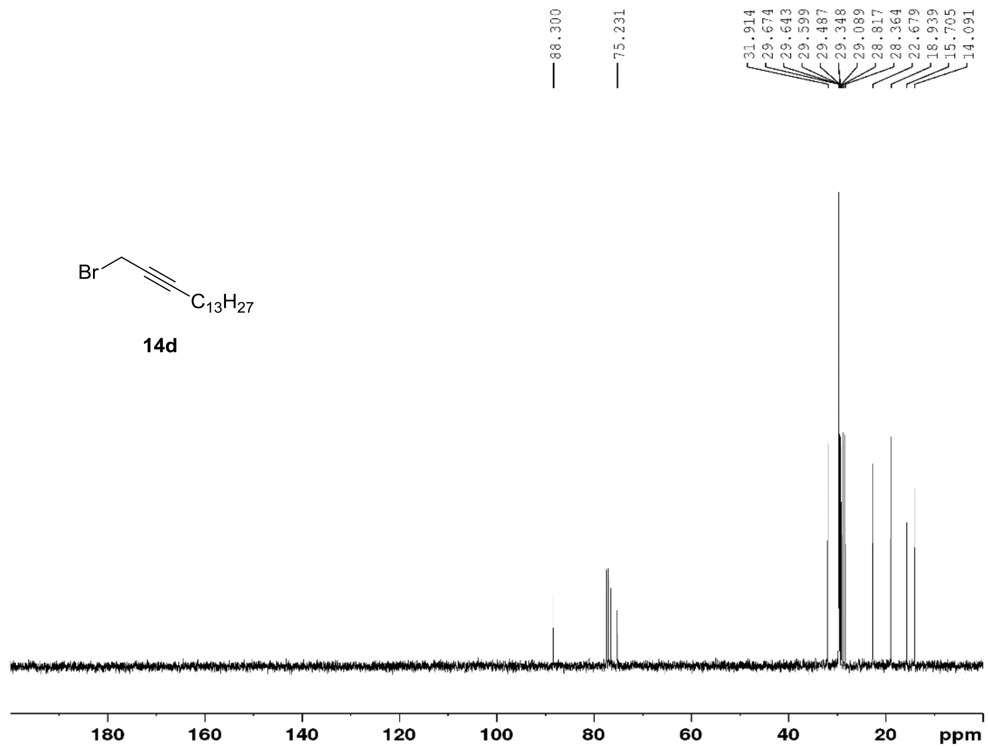

(5Z,8Z)-methyl tetradeca-5,8-dienoate (16a): Pale yellow oil, 29% yield (over 2 steps), eluent system: 98:2 hexane-EA, ^{1}H NMR (500 MHz, $CDCl_3$) δ 5.40 – 5.31 (m, 4H), 3.67 (s, 3H), 2.77 (t, *J* = 6.7 Hz, 2H), 2.33 (dd, *J* = 9.8, 5.3 Hz, 2H), 2.13 – 2.09 (m, 2H), 2.06 – 2.03 (m, 2H), 1.72 (dd, *J* = 14.9, 7.5 Hz, 2H), 1.37 – 1.30 (m, 6H), 0.89 (t, *J* = 6.8 Hz, 3H); ^{13}C NMR (125 MHz, $CDCl_3$) δ 174.01, 130.35, 129.21, 128.58, 127.58, 51.39, 33.40, 31.47, 29.28, 27.16, 26.49, 25.55, 24.76, 22.52, 14.00.

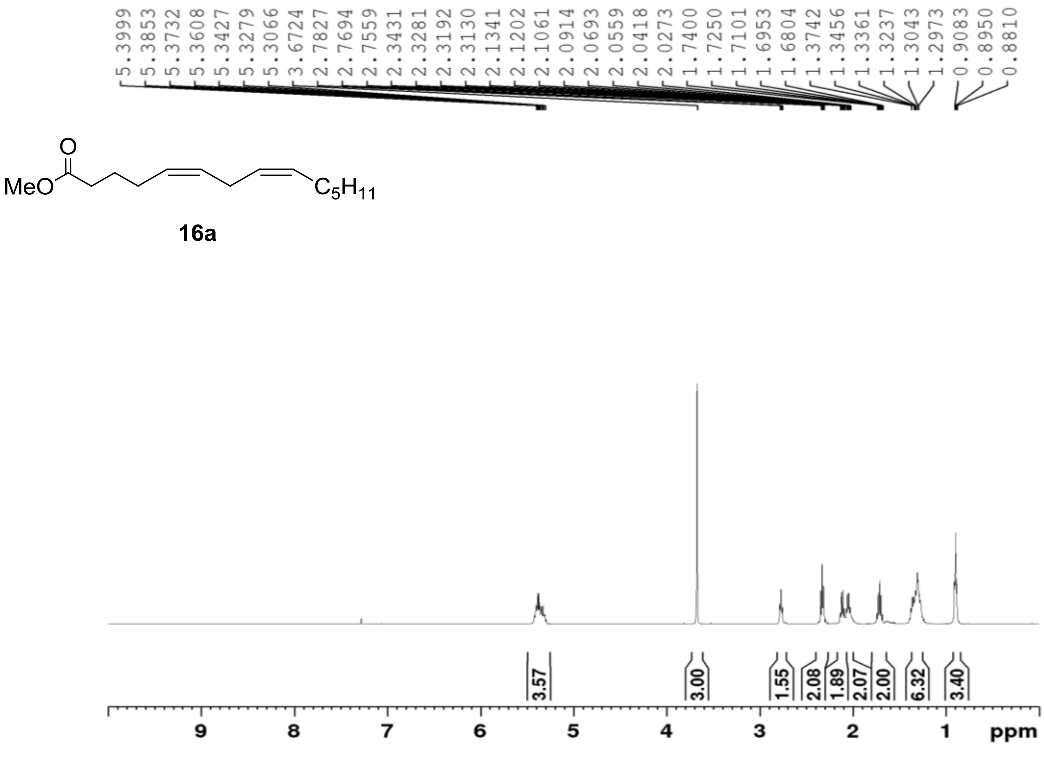

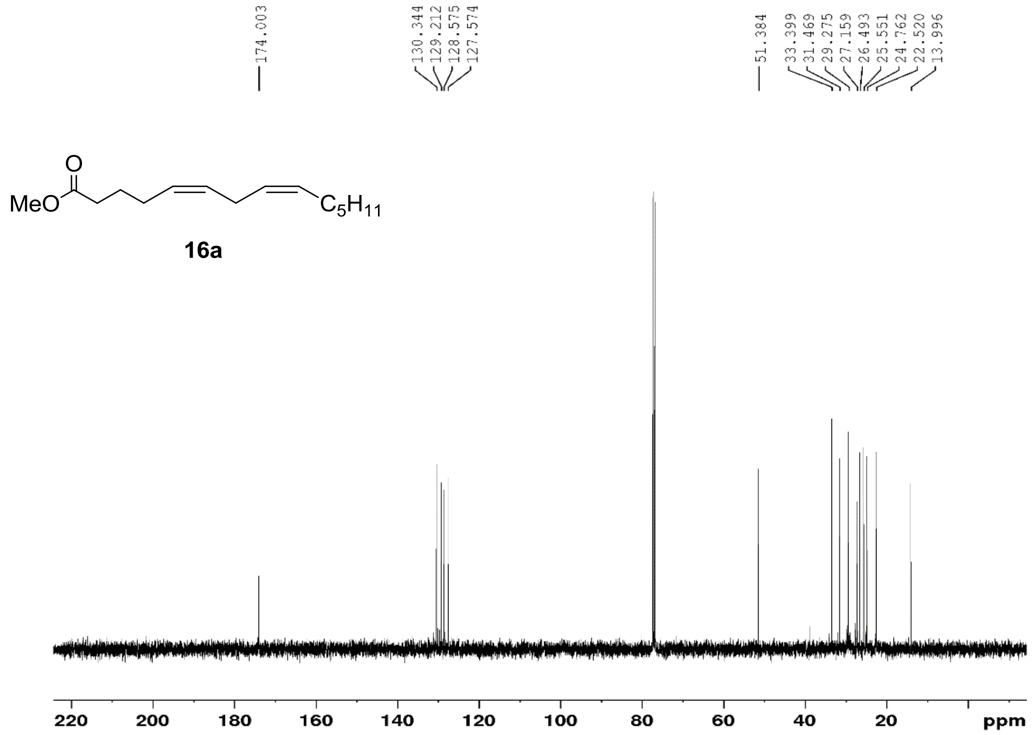

(5Z,8Z)-methyl hexadeca-5,8-dienoate (16b): Yellow oil, 40% yield (over 2 steps), eluent system: 98:2 hexane-EA,^{1}H NMR (500 MHz, $CDCl_3$) δ 5.43 – 5.25 (m, 4 H), 3.65 (s, 3 H), 2.75 (t, *J* = 6.7 Hz, 2 H), 2.36 – 2.25 (m, 2 H), 2.09 (dd, *J* = 14.2, 7.1 Hz, 2 H), 2.03 (dd, *J* = 14.0, 7.0 Hz, 2 H), 1.73 – 1.65 (m, 2 H), 1.38 – 1.19 (m, 10 H), 0.87 (t, *J* = 6.8 Hz, 3 H); ^{13}C NMR (125 MHz, $CDCl_3$) δ 174.04, 130.39, 129.26, 128.61, 127.60, 51.42, 33.44, 31.86, 29.65, 29.27, 29.22, 27.24, 26.53, 25.59, 24.80, 22.66, 14.08.

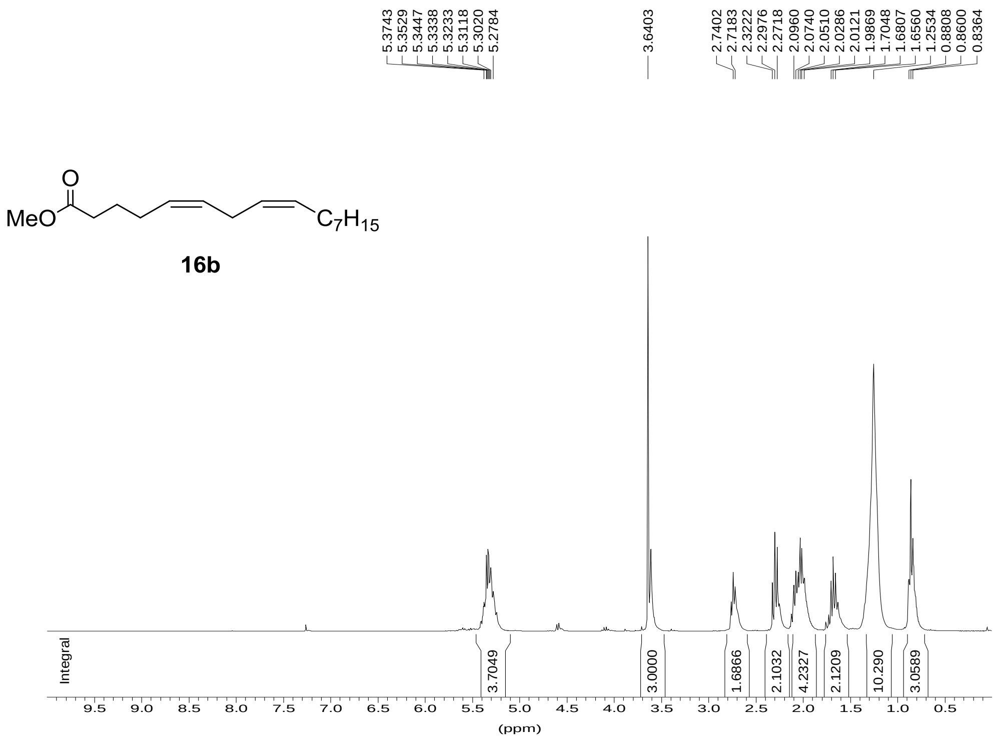

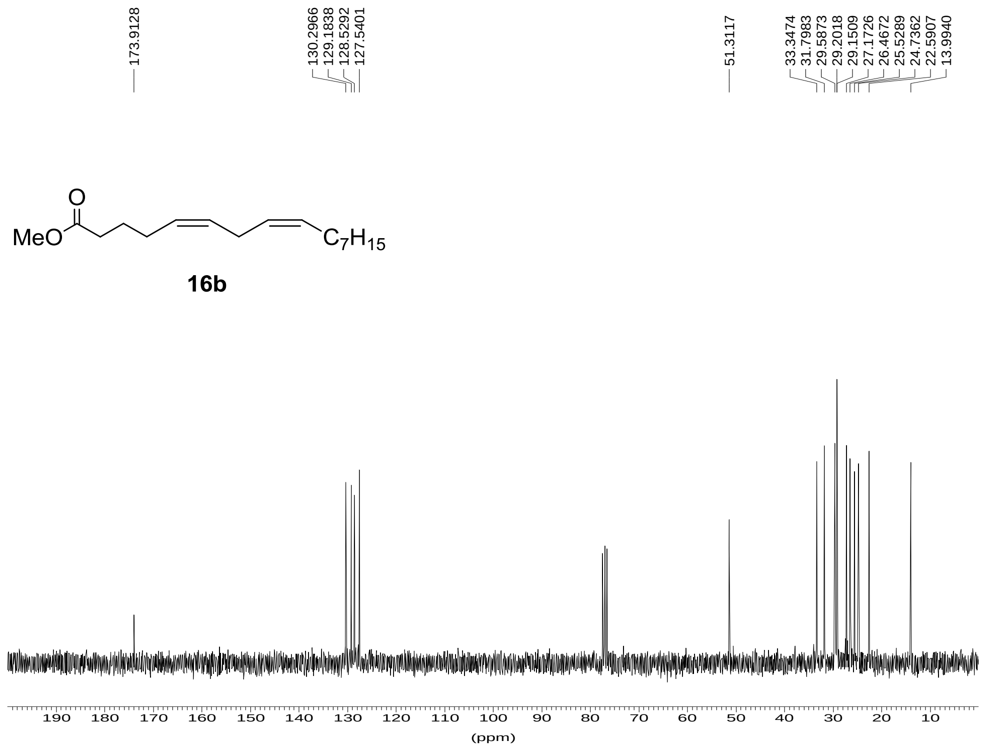

(5Z,8Z)-methyl icosa-5,8-dienoate (16c): Yellow oil, 23% yield (over 2 steps), eluent system: 98:2 hexane-EA, ^{1}H NMR (500 MHz, $CDCl_3$) δ 5.43 – 5.30 (m, 4 H), 3.68 (s, 3 H), 2.78 (t, *J* = 6.8 Hz, 2 H), 2.35 – 2.32 (m, 2 H), 2.12 (dd, *J* = 14.2, 7.1 Hz, 2 H), 2.06 (dd, *J* = 14.0, 7.0 Hz, 2 H), 1.75 – 1.69 (m, 2 H), 1.28 (m, 18 H), 0.90 (t, *J* = 6.9 Hz, 3 H); ^{13}C NMR (125 MHz, $CDCl_3$) δ 174.01, 130.38, 129.26, 128.60, 127.60, 51.40, 33.43, 31.92, 29.68 (2 C), 29.65 (2 C), 29.56, 29.35, 29.32, 27.24, 26.53, 25.59, 24.80, 22.68, 14.08.

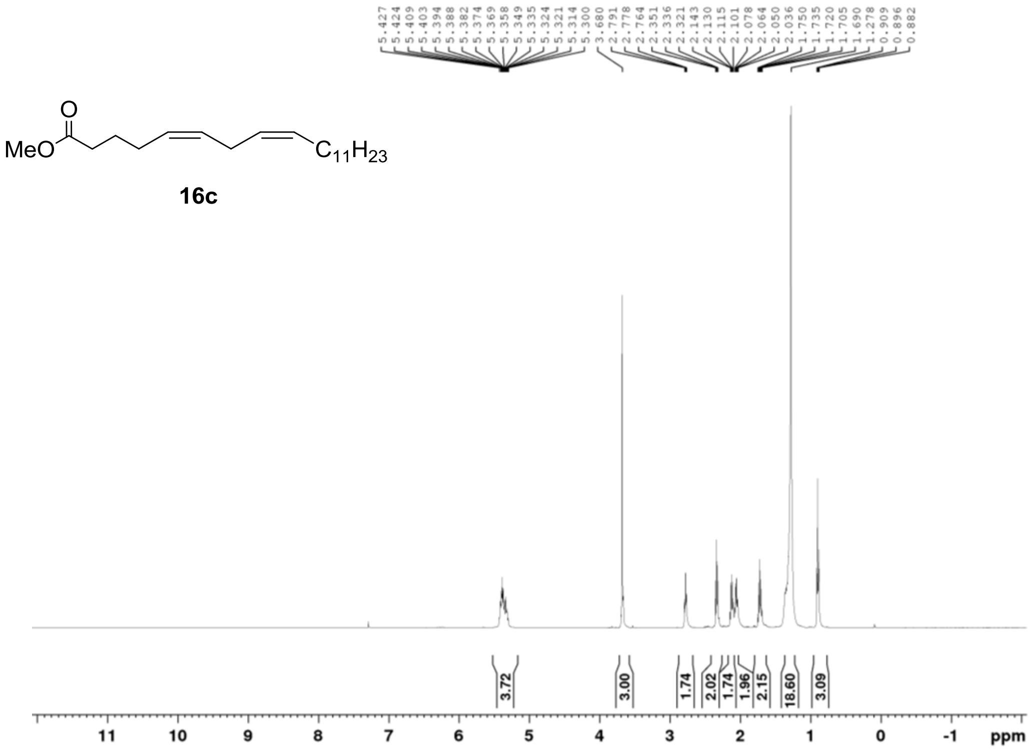

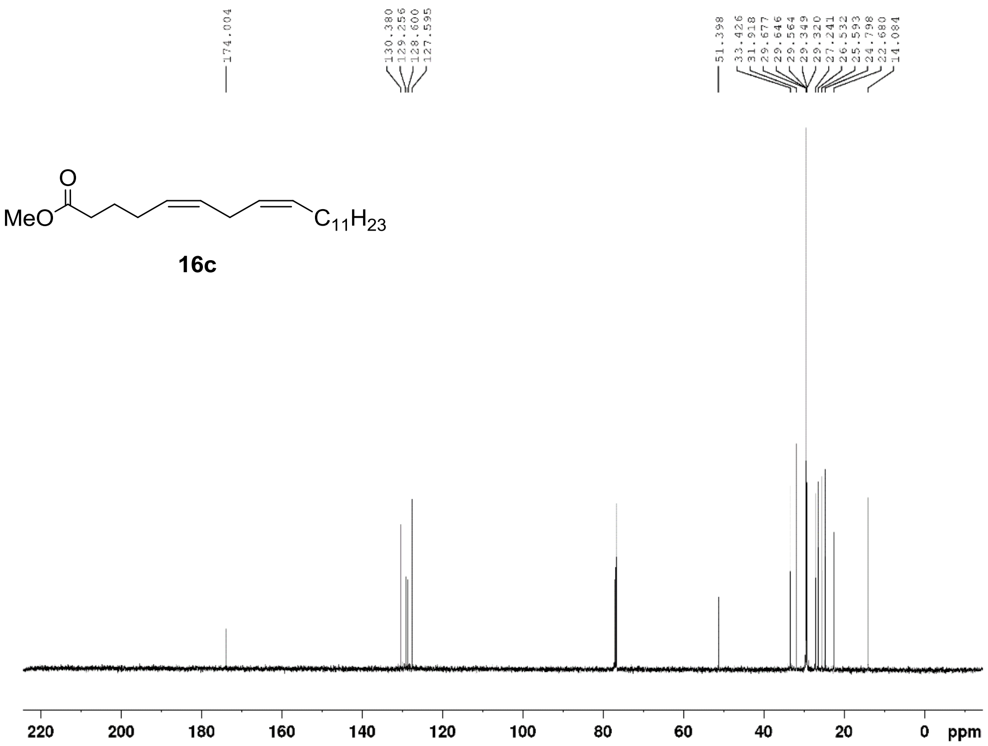

(5Z,8Z)-methyl docosa-5,8-dienoate (16d): Yellow oil, 70% yield (over 2 steps), eluent system: 98:2 hexane-EA, ^{1}H NMR (500 MHz, $CDCl_3$) δ 5.49 – 5.27 (m, 4 H), 3.68 (s, 3 H), 2.78 (t, *J* = 6.8 Hz, 2 H), 2.38 – 2.29 (m, 2 H), 2.12 (dd, *J* = 14.2, 7.0 Hz, 2 H), 2.06 (dd, *J* = 14.0, 7.0 Hz, 2 H), 1.77 – 1.67 (m, 2 H), 1.40 – 1.32 (m, 2 H), 1.30 (d, *J* = 18.6 Hz, 20 H), 0.89 (t, *J* = 6.9 Hz, 3 H); δ ^{13}C NMR (75 MHz, $CDCl_3$) δ 173.65, 130.13, 129.10, 128.41, 127.46, 51.12, 33.20, 31.82, 29.59 (2 C), 29.55 (2 C), 29.46 (2 C), 29.26 (2 C), 29.21, 27.10, 26.38, 25.45, 24.66, 22.56, 13.92.

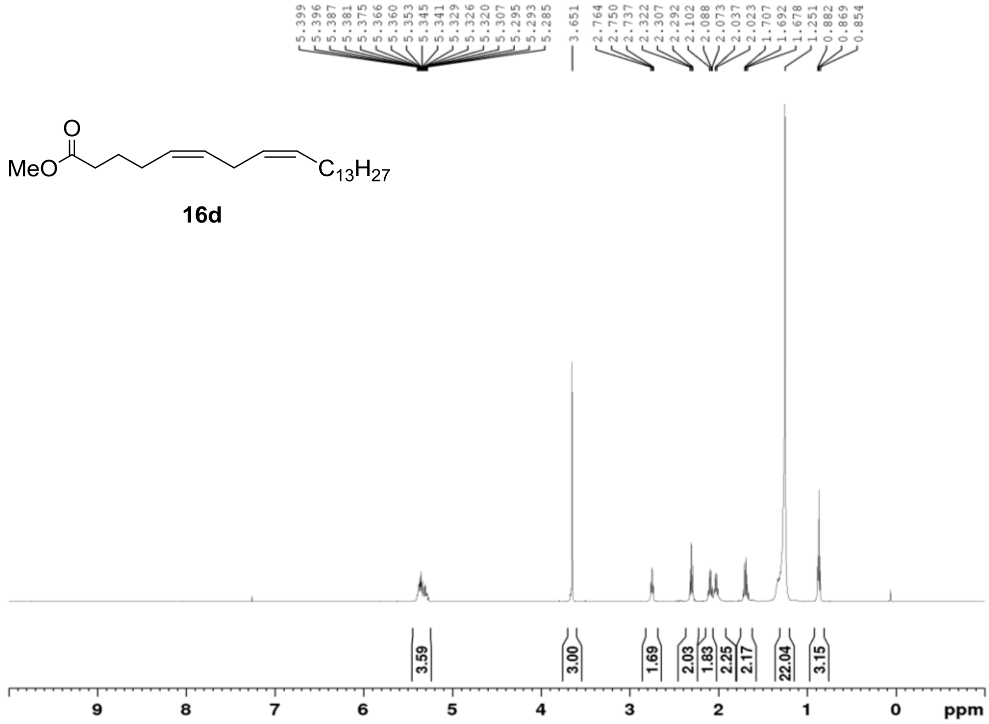

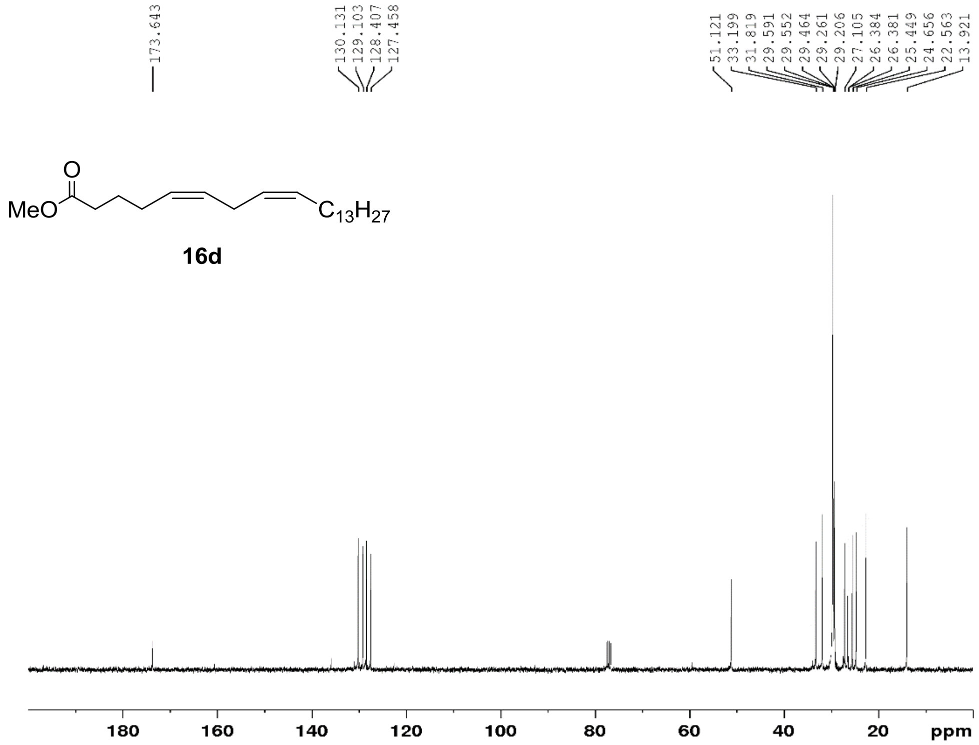

(6Z,9Z,12Z,15Z)-ethyl henicosa-6,9,12,15-tetraenoate (18a): Pale yellow liquid, 48% yield (over 2 steps), eluent system: 98:2 hexane-EA, ^{1}H NMR (300 MHz, $CDCl_3$) δ 5.41 – 5.38 (m, 7H), 4.15 (q, *J* = 7.1 Hz, 2H), 2.86 – 2.80 (m, 4H), 2.32 (t, *J* = 7.5 Hz, 2H), 2.10 – 2.05 (m, 6H), 1.70 – 1.62 (m, 2H), 1.40 – 1.33 (m, 4H), 1.32 – 1.25 (m, 7H), 0.92 (t, *J* = 6.7 Hz, 3H); ^{13}C NMR (75 MHz, $CDCl_3$) δ 173.56, 130.36, 129.60, 128.45, 128.22, 128.06, 128.01, 127.82, 127.49, 60.08, 34.16, 31.45, 29.26, 29.03, 27.15, 27.05, 26.80, 25.56(2C), 24.54, 22.50, 14.17, 13.99.

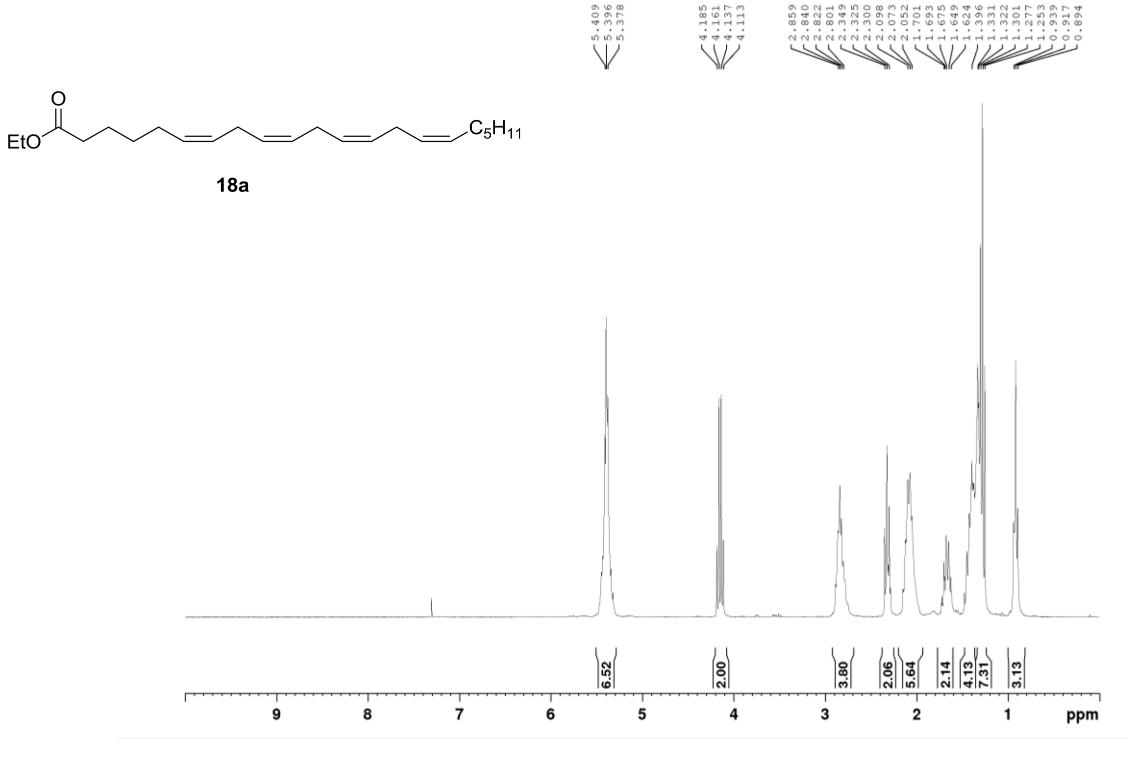

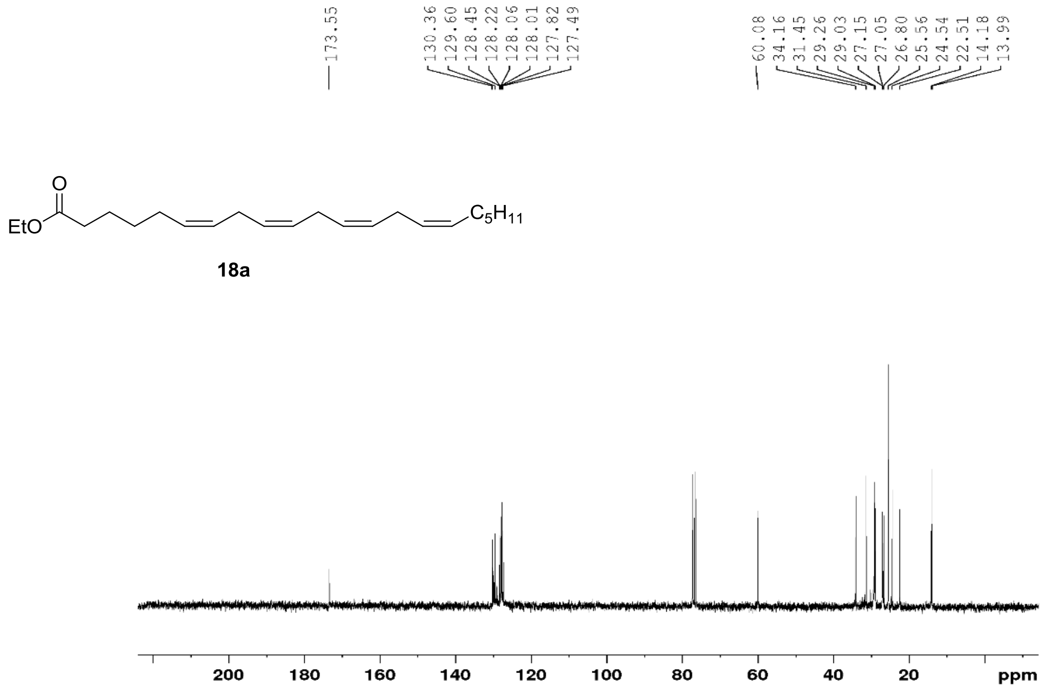

18a

(6Z,9Z,12Z,15Z)-ethyl nonadeca-6,9,12,15-tetraenoate (18b): Pale yellow liquid, 55% yield (over 2 steps), eluent system: 98:2 hexane-EA,^{1}H NMR (500 MHz, $CDCl_3$) δ 5.40 – 5.38 (m, 8H), 4.15 (q, *J* = 7.1 Hz, 2H), 2.87 – 2.84 (m, 6H), 2.32 (t, *J* = 7.5 Hz, 2H), 2.12 – 2.04 (m, 4H), 1.69 – 1.64 (m, 2H), 1.42 (ddd, *J* = 11.8, 7.4, 2.1 Hz, 4H), 1.28 (t, *J* = 7.1 Hz, 3H), 0.94 (t, *J* = 7.4 Hz, 3H); ^{13}C NMR (125 MHz, $CDCl_3$) δ 173.63, 130.16, 129.68, 128.54, 128.31, 128.13, 128.10, 127.90, 127.78, 60.14, 34.24, 29.29, 29.08, 26.86, 25.65, 25.63(2C), 24.60, 22.75, 14.22, 13.74.

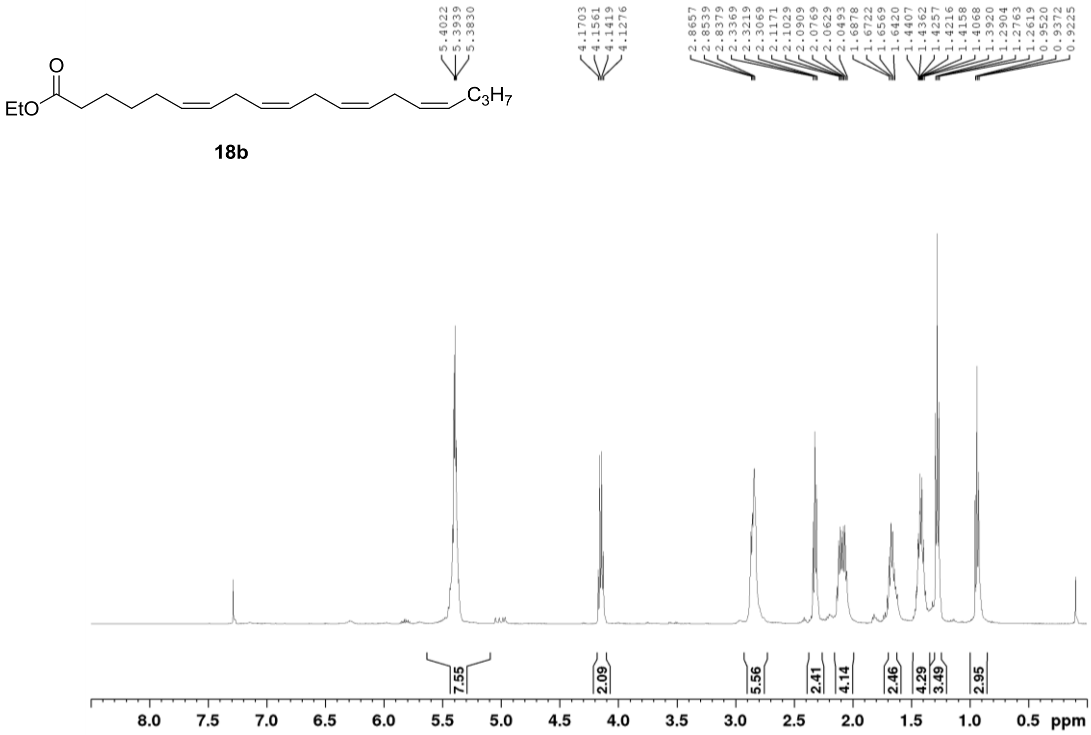

18b

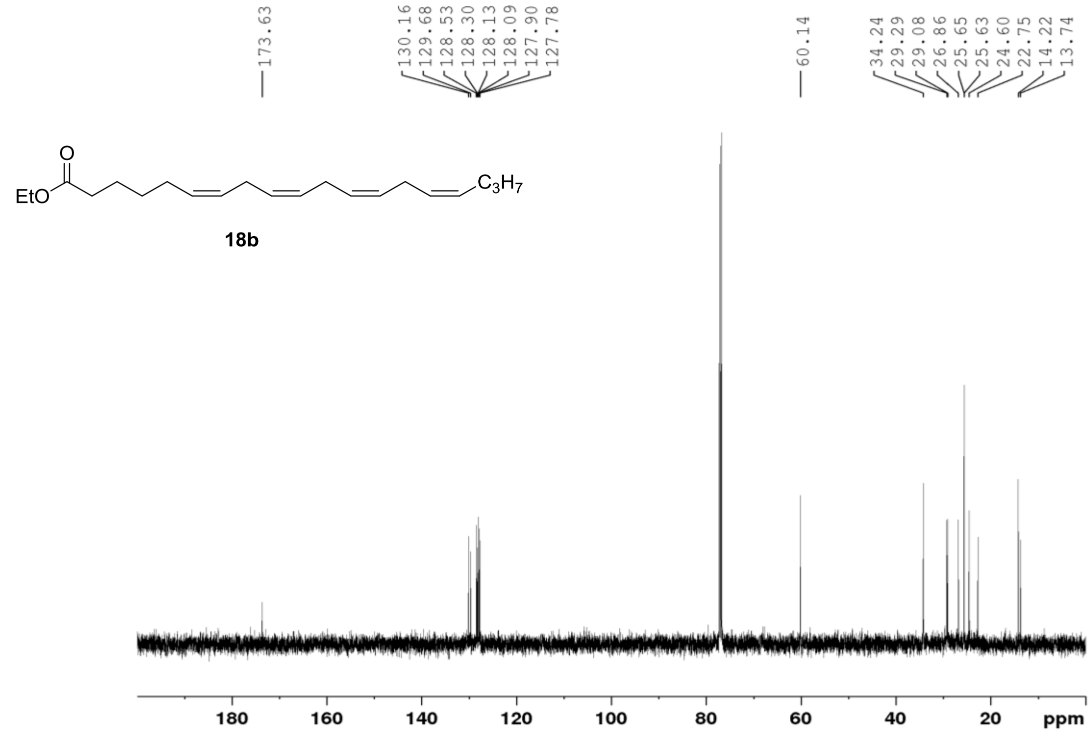

(7Z,10Z,13Z,16Z)-methyl docosa-7,10,13,16-tetraenoate (18c): Yellow liquid, 25% yield (over 2 steps), eluent system: 98:2 hexane-EA, ^{1}H NMR (500 MHz, $CDCl_3$) δ 5.39 – 5.34 (m, 8H), 3.67 (s, 3H), 2.85 – 2.77 (m, 6H), 2.31 (t, *J* = 7.5 Hz, 2H), 2.07 – 2.03 (m, 4H), 1.65 – 1.60 (m, 2H), 1.39 – 1.33 (m, 6H), 1.32 – 1.25 (m, 4H), 0.88 (dd, *J* = 9.1, 4.7 Hz, 3H); ^{13}C NMR (125 MHz, $CDCl_3$) δ 174.20, 130.47, 130.03, 128.53, 128.39, 128.06, 127.93, 127.90, 127.57, 51.43, 34.05, 31.52, 29.32, 29.25, 28.79, 27.22, 27.04, 25.64(3C), 24.86, 22.56, 14.04.

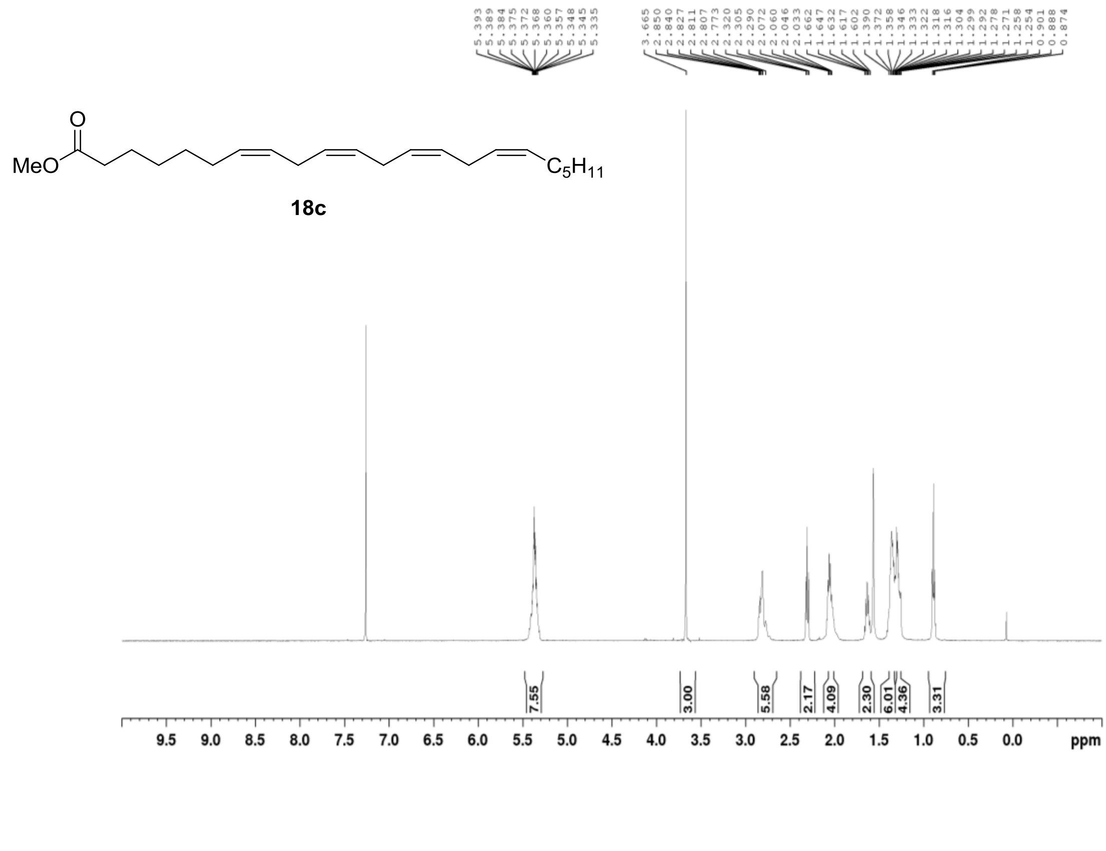

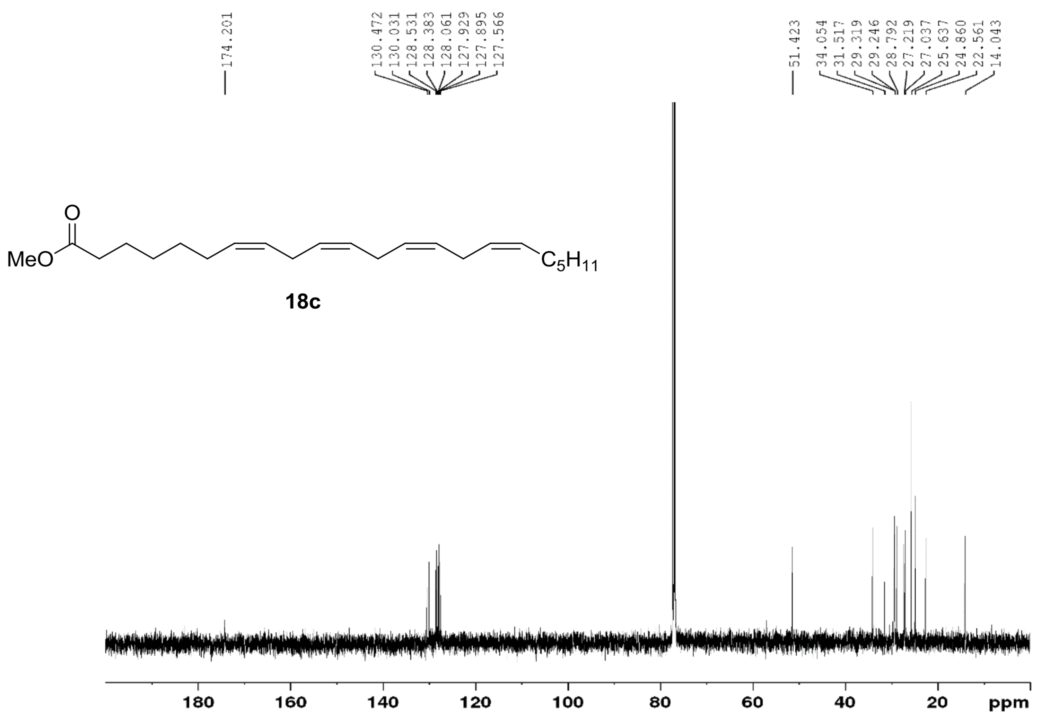

Methyl 4-((5Z,8Z,11Z,14Z)-icosa-5,8,11,14-tetraenamido)butanoate (20a): Pale yellow liquid, 53% yield (over 2 steps), eluent system: 3:1 hexane-ethyl acetate. ^{1}H NMR (300 MHz, $CDCl_3$) δ 5.73 (s, 1 H), 5.41 – 5.34 (m, 8 H), 3.66 (s, 3 H), 3.27 (dd, *J* = 12.9, 6.7 Hz, 2 H), 2.84 – 2.78 (m, 6 H), 2.34 (q, *J* = 7.0 Hz, 2 H), 2.18 – 2.01 (m, 6 H), 1.84 – 1.77 (m, 4 H), 1.72 – 1.67 (m, 2 H), 1.29 – 1.26 (m, 8 H), 0.87 (t, *J* = 6.7 Hz, 3 H); ^{13}C NMR (75 MHz, $CDCl_3$) δ 173.87, 172.93, 130.47, 129.07, 128.70, 128.55, 128.18, 128.12, 127.81, 127.47, 51.68, 38.86, 36.05, 31.44, 29.72, 29.66, 29.27, 27.17, 26.63, 25.58 (2 C), 25.47, 24.61, 22.52, 14.02. HRMS (ESI): calculated for $C_{25}H_{40}NO_3$ (M-1) 402.3008, found 402.3019.

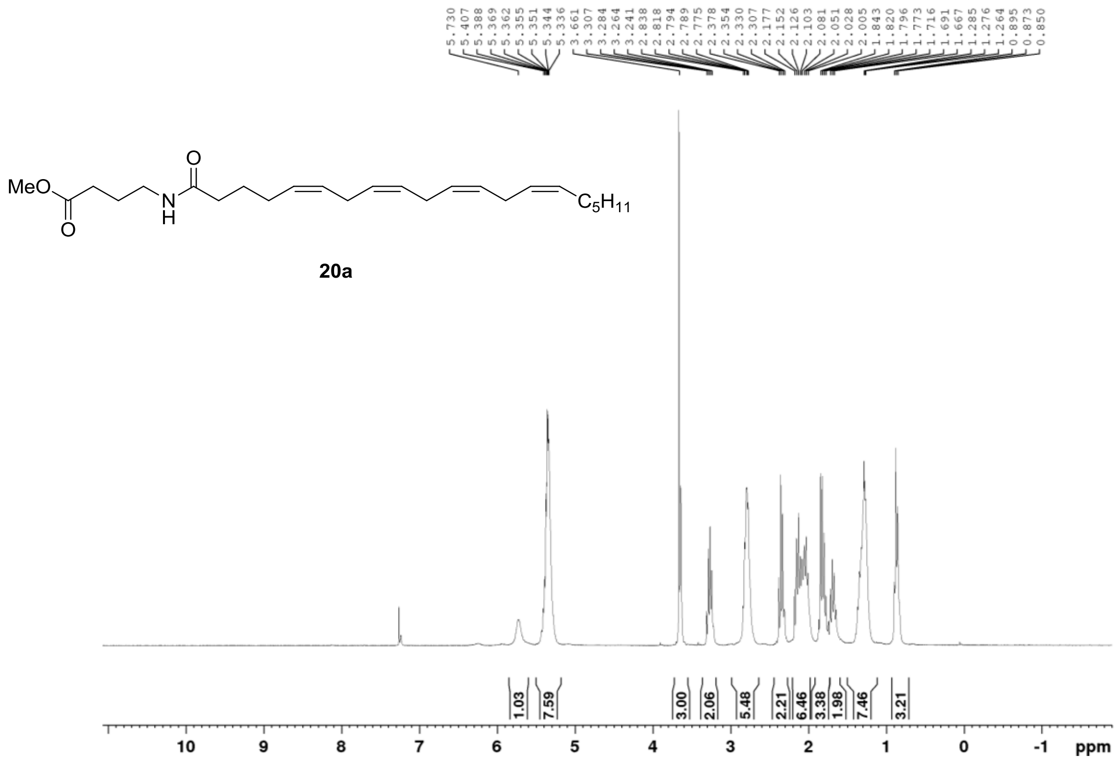

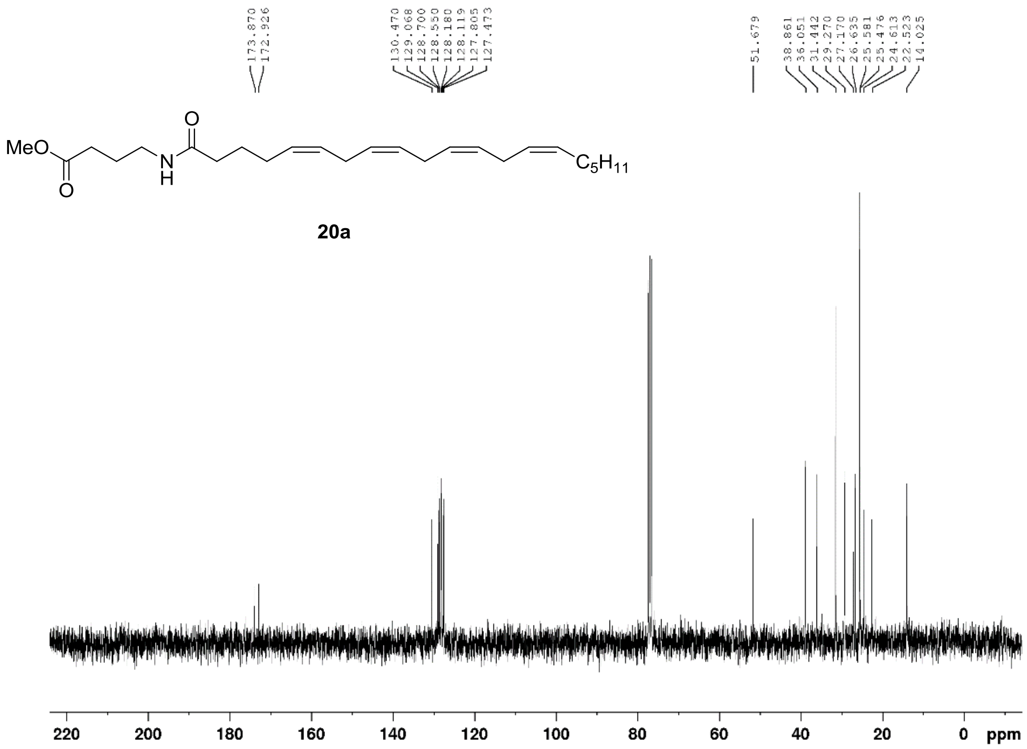

Methyl 5-((5Z,8Z,11Z,14Z)-icosa-5,8,11,14-tetraenamido)pentanoate (20b): Pale yellow liquid, 53% yield (over 2 steps), eluent system: 3:1 hexane- ethyl acetate. ^{1}H NMR (500 MHz, $CDCl_3$) δ 5.62 (s, 1 H), 5.47 – 5.29 (m, 8 H), 3.68 (s, 3 H), 3.27 (q, *J* = 6.6 Hz, 2 H), 2.89 – 2.77 (m, 4 H), 2.35 (t, *J* = 7.2 Hz, 2 H), 2.21 – 2.16 (m, 2 H), 2.12 (dd, *J* = 13.4, 6.9 Hz, 2 H), 2.07 (dd, *J* = 14.2, 7.1 Hz, 2 H), 1.73 (dt, *J* = 14.9, 7.5 Hz, 2 H), 1.66 (dd, *J* = 15.2, 7.4 Hz, 2 H), 1.59 – 1.51 (m, 2 H), 1.42 – 1.25 (m, 8 H), 0.90 (t, *J* = 6.7 Hz, 3 H); ^{13}C NMR (125 MHz, $CDCl_3$) δ 173.91, 172.85, 130.51, 129.15, 128.73, 128.60, 128.22, 128.18, 127.86, 127.53, 51.55, 38.96, 36.12, 33.46, 31.50, 29.31, 29.06, 27.21, 26.69 (2 C), 25.63, 25.56 (2 C), 22.55, 22.05, 14.04. HRMS (ESI): calculated for $C_{26}H_{42}NO_3$ (M-1) 416.3031, found 416.3039.

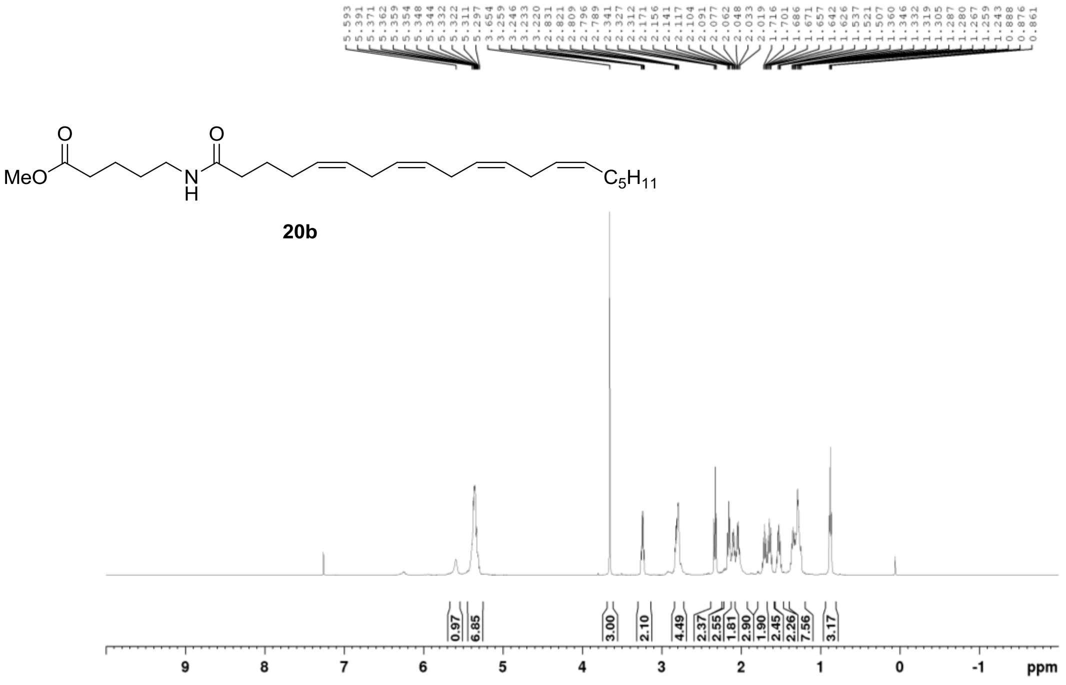

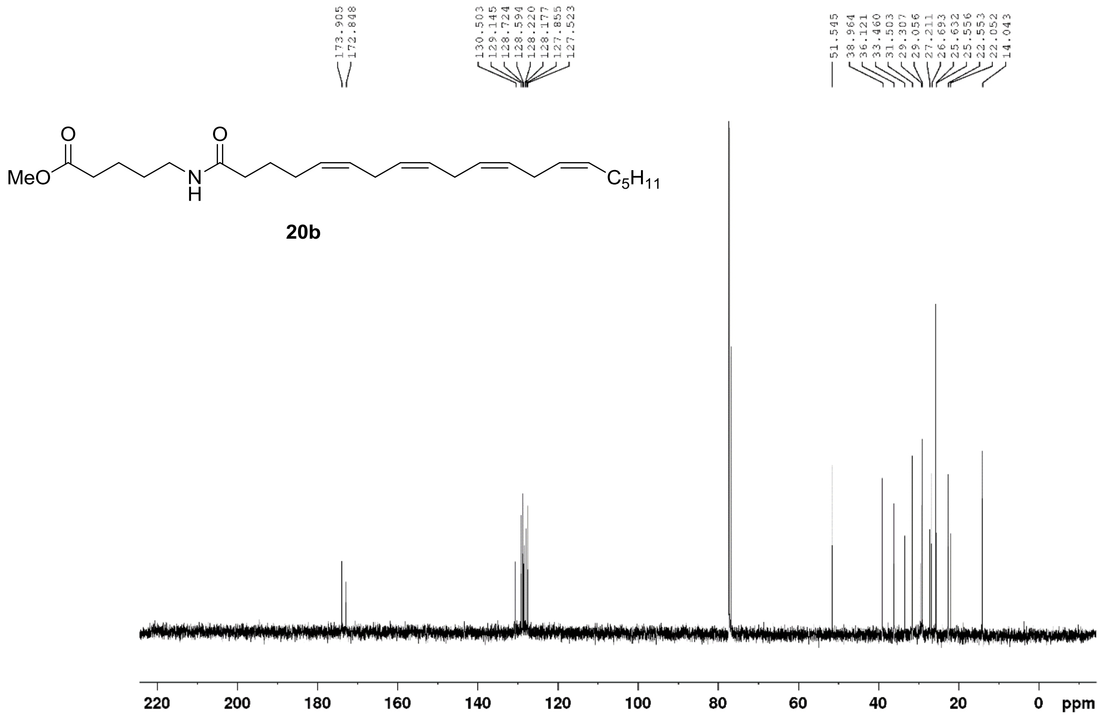

Methyl 6-((5Z,8Z,11Z,14Z)-icosa-5,8,11,14-tetraenamido)hexanoate (20c): Pale yellow liquid, 62% yield (over 2 steps), eluent system: 3:1 hexane- ethyl acetate. ^{1}H NMR (300 MHz, $CDCl_3$) δ 5.55 (s, 1H), 5.38 – 5.34 (m, 8H), 3.65 (s, 3H), 3.23 (dd, *J* = 13.1, 6.8 Hz, 2H), 2.82 – 2.80 (m, 6H), 2.30 (t, *J* = 7.4 Hz, 2H), 2.14 (dd, *J* = 14.9, 7.0 Hz, 2H), 2.08 – 2.00 (m, 4H), 1.66 (ddt, *J* = 22.6, 15.1, 7.6 Hz, 4H), 1.52 – 1.47 (m, 2H), 1.36 – 1.28 (m, 8H), 0.87 (t, *J* = 6.7 Hz, 3H). ^{13}C NMR (75 MHz, $CDCl_3$) δ 174.00, 172.78, 130.46, 129.10, 128.66, 128.54, 128.17, 128.12, 127.80, 127.46, 51.45, 39.14, 36.10, 33.77, 31.45, 29.25(2C), 27.16, 26.63, 26.28, 25.58(3C), 25.52, 24.38, 22.51, 14.01. HRMS (ESI): calculated for $C_{27}H_{45}NO_3Na$ (M+23) 454.3292, found 454.3302.

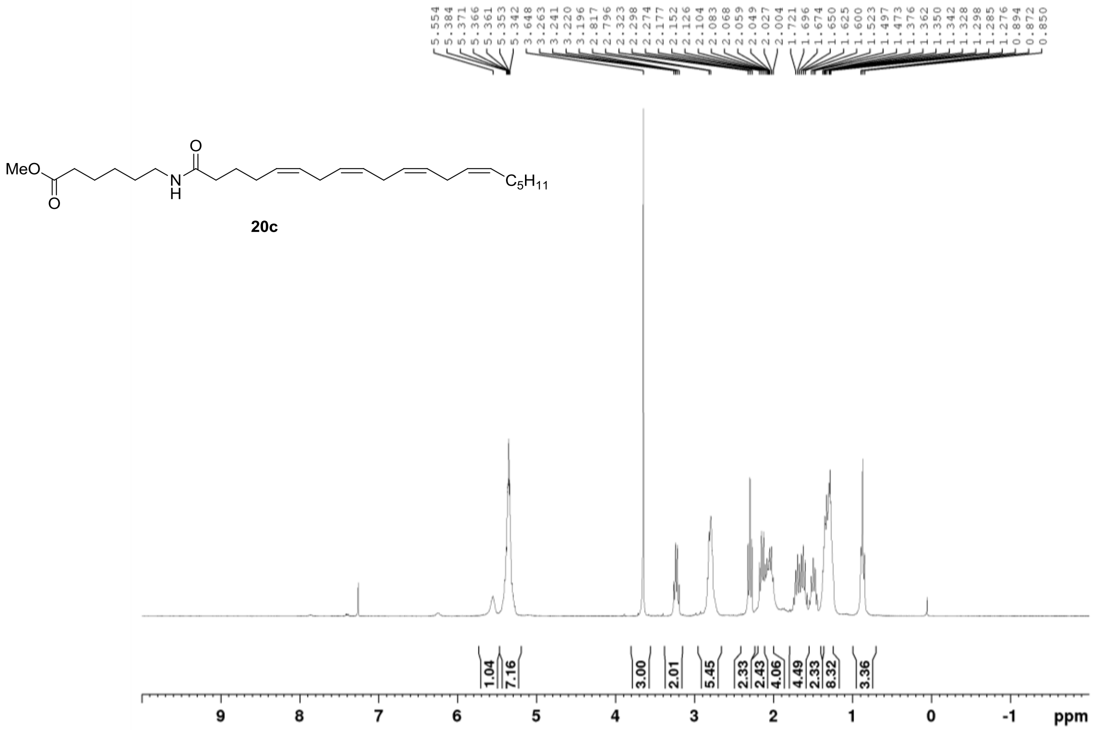

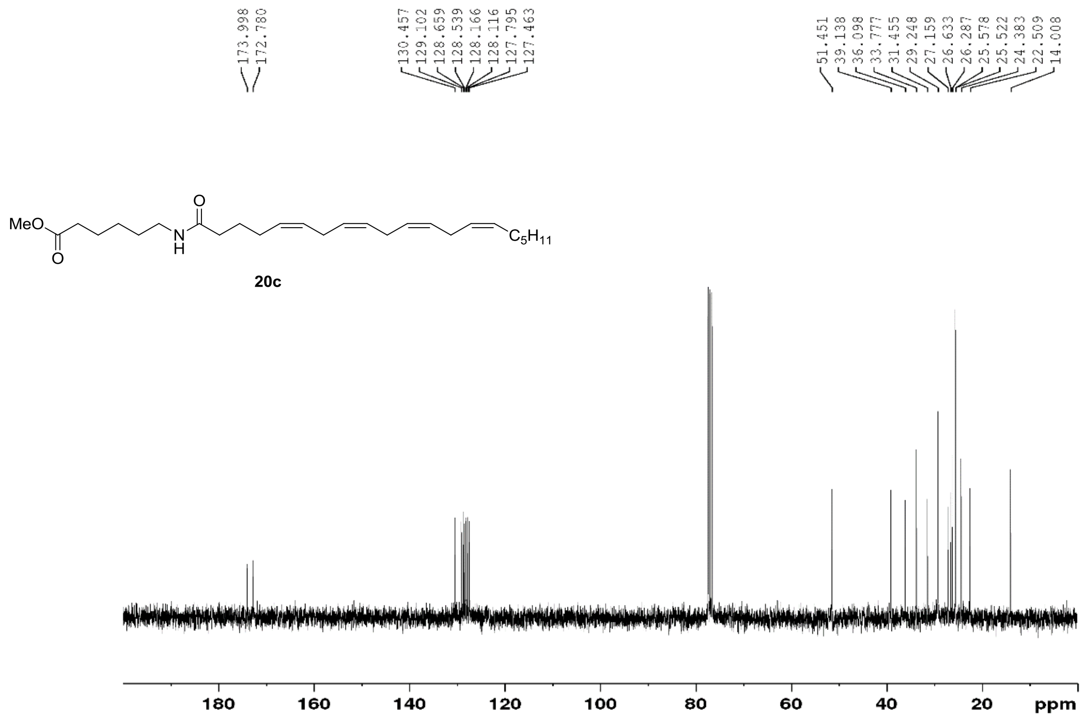

Methyl 3-((5Z,8Z,11Z,14Z)-icosa-5,8,11,14-tetraenamido)benzoate (20d): white solid, 55% yield (over 2 steps), eluent system: 3:1 hexane- ethyl acetate. ^{1}H NMR (300 MHz, DMSO) δ 10.10 (s, 1H), 8.28 (s, 1H), 7.85 (d, *J* = 7.3 Hz, 1H), 7.62 (d, *J* = 7.7 Hz, 1H), 7.43 (t, *J* = 7.9 Hz, 1H), 5.39 – 5.29 (m, 8H), 3.85 (s, 3H), 2.76 (dd, *J* = 11.3, 5.4 Hz, 5H), 2.34 (t, *J* = 7.4 Hz, 2H), 2.11 – 2.07 (m, 2H), 2.01 – 1.97 (m, 3H), 1.69 – 1.64 (m, 2H), 1.29 – 1.24 (m, 6H), 0.83 (t, *J* = 5.6 Hz, 3H). ^{13}C NMR (75 MHz, DMSO) δ 171.27, 166.09, 139.68, 129.99, 129.87, 129.26, 129.04, 128.19, 128.05, 127.94, 127.75, 127.60, 127.45, 123.48, 123.36, 119.46, 52.09, 35.71, 30.87, 28.70, 26.58, 26.17, 25.16(3C), 24.87, 21.96, 13.87. HRMS (ESI): calculated for $C_{28}H_{38}NO_3$ (M-1) 436.2857, found 436.2848.

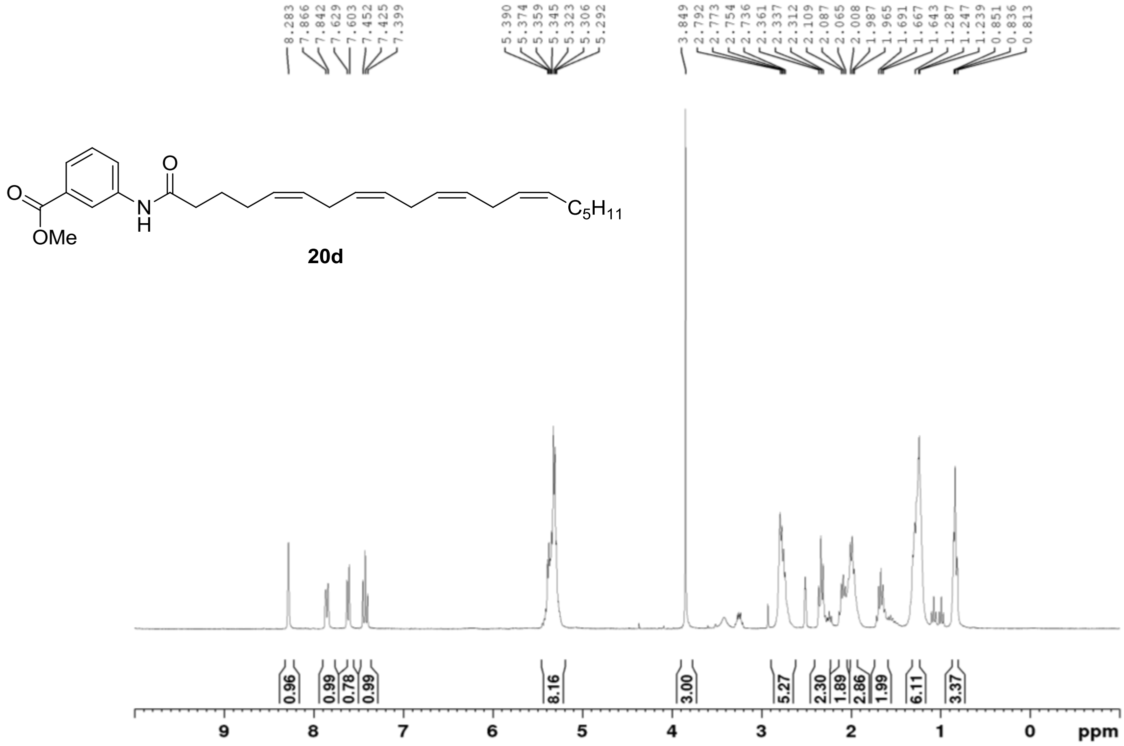

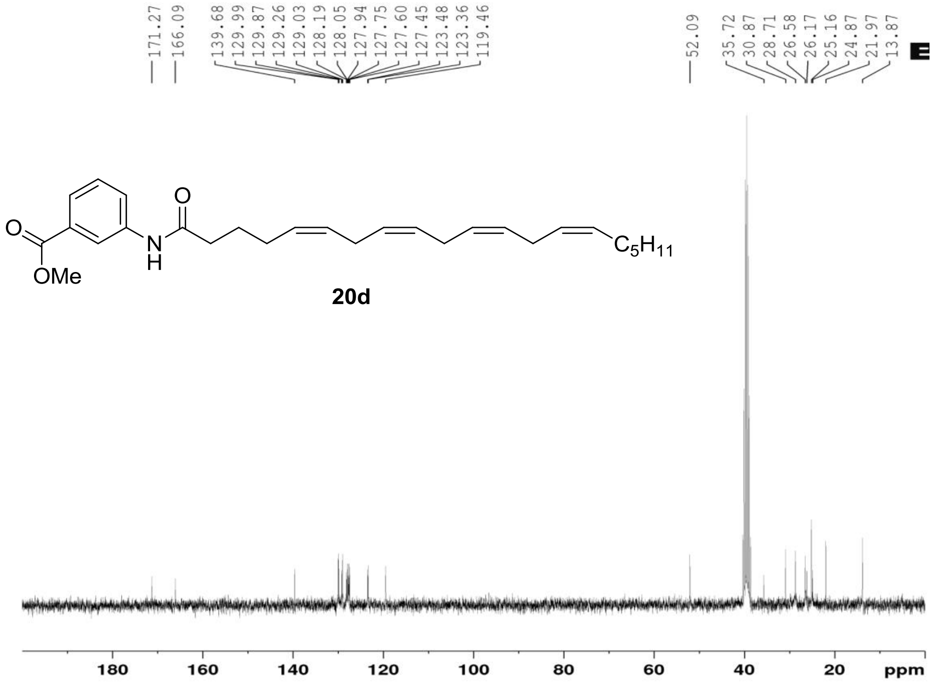

Methyl 4-((5Z,8Z,11Z,14Z)-icosa-5,8,11,14-tetraenamidomethyl)benzoate (20e): Pale yellow solid, 76% yield (over 2 steps), eluent system: 3:1 hexane-ethyl acetate. ^{1}H NMR (300 MHz, $CDCl_3$) δ 7.96 (d, *J* = 8.3 Hz, 2H), 7.31 (d, *J* = 8.1 Hz, 2H), 6.02 (s, 1H), 5.39 – 5.34 (m, 8H), 4.46 (d, *J* = 5.8 Hz, 2H), 3.89 (s, 3H), 2.82 – 2.78 (m, 6H), 2.30 – 2.21 (m, 2H), 2.15 – 2.11(m, 2H), 2.08 – 2.01 (m, 2H), 1.75 (dd, *J* = 14.8, 7.4 Hz, 2H), 1.34 – 1.29 (m, 6H), 0.87 (t, *J* = 6.7 Hz, 3H). ^{13}C NMR (75 MHz, $CDCl_3$) δ 172.85, 166.74, 143.61, 130.46, 129.89(2C), 129.19, 128.93, 128.78, 128.53, 128.18, 128.04, 127.76, 127.44(3C), 52.04, 43.08, 35.89, 31.43, 29.24, 27.14, 26.59, 25.56(3C), 25.42, 22.49, 14.00, HRMS (ESI): calculated for $C_{29}H_{40}NO_3$ (M-1) 450.3014, found 450.3015.

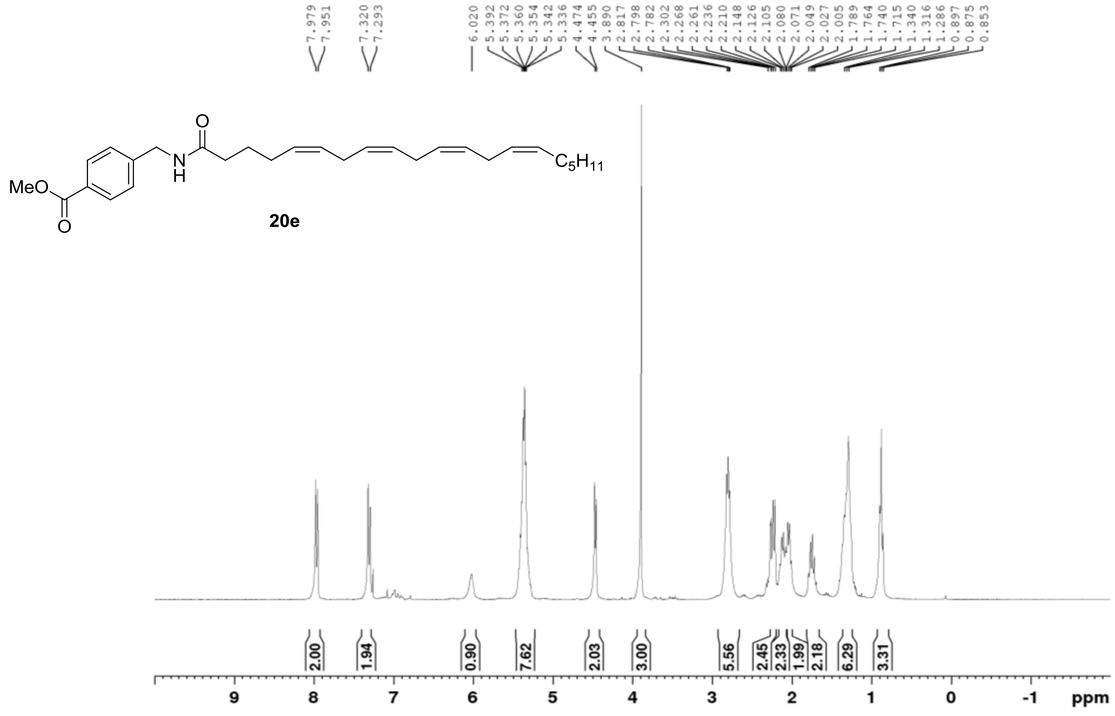

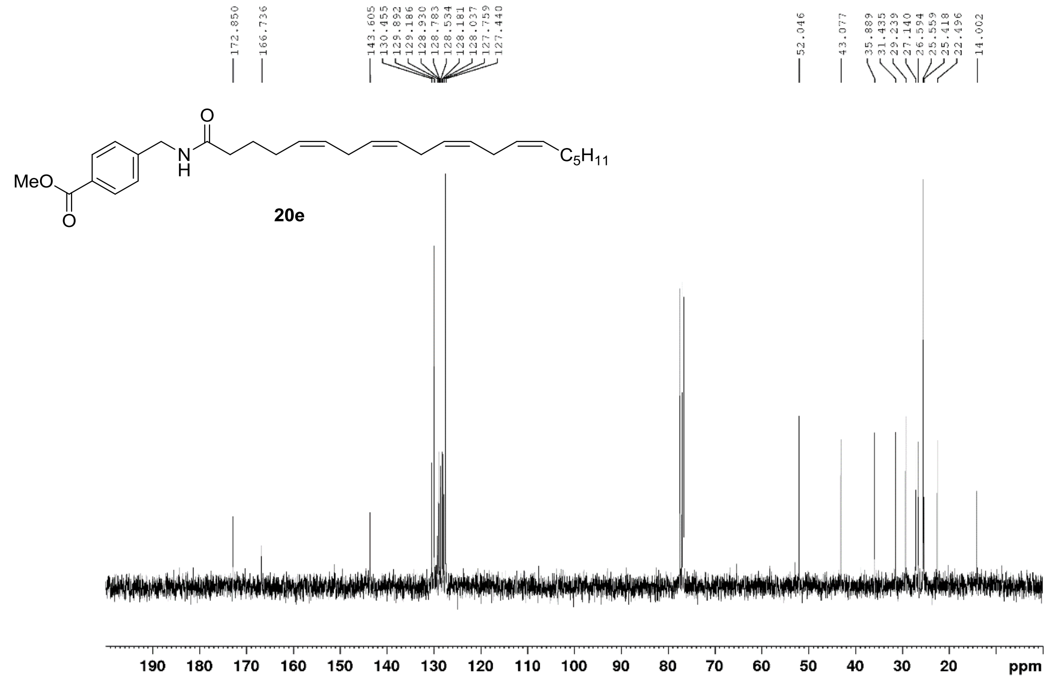

Methyl 3-((5Z,8Z,11Z,14Z)-icosa-5,8,11,14-tetraenamidomethyl)benzoate (20f): Pale yellow solid, 70% yield (over 2 steps), eluent system: 3:1 hexane-ethyl acetate. ^{1}H NMR (300 MHz, $CDCl_3$) δ 7.97 (dd, *J* = 6.8, 1.4 Hz, 2H), 7.52 (d, *J* = 7.8 Hz, 1H), 7.43 (dd, *J* = 11.2, 4.8 Hz, 1H), 5.91 (s, 1H), 5.42 – 5.38 (m, 8H), 4.52 (d, *J* = 5.8 Hz, 2H), 3.94 (s, 3H), 2.86 – 2.82 (m, 6H), 2.30 – 2.25 (m, 2H), 2.15 – 2.13 (m, 2H), 2.09 – 2.07 (m, 2H), 1.84 – 1.76 (m, 2H), 1.34 – 1.29 (m, 6H), 0.92 (t, *J* = 6.8 Hz, 3H). ^{13}C NMR (75 MHz, $CDCl_3$) δ 172.79, 166.82, 138.81, 132.34(2C), 130.50, 129.01, 128.79(2C), 128.66(2C), 128.57, 128.21, 128.10, 127.81, 127.48, 52.15, 43.12, 35.97, 31.47, 29.28, 27.18, 26.63, 25.59(2C), 25.44, 22.53, 14.03. HRMS (ESI): calculated for $C_{29}H_{40}NO_3$ (M-1) 450.3014, found 450.3018.

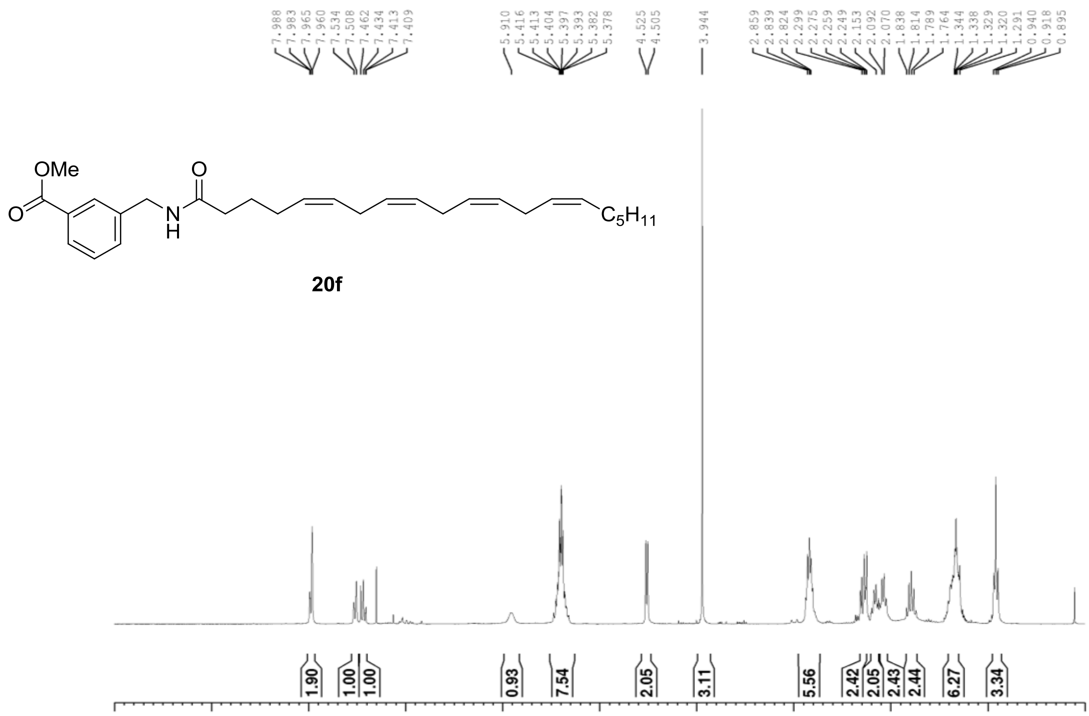

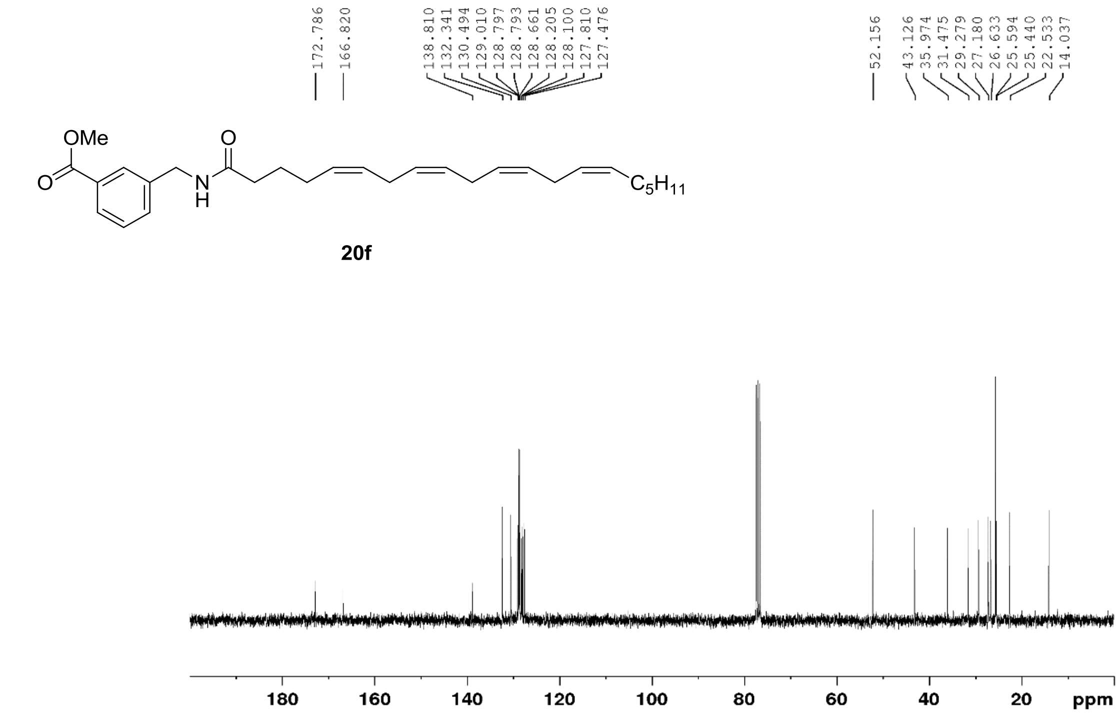

Methyl 5-((6Z,9Z,12Z,15Z)-henicosa-6,9,12,15-tetraenamido)pentanoate (20g): Pale yellow solid, 45% yield (over 2 steps), eluent system: 3:1 hexane-ethyl acetate. ^{1}H NMR (500 MHz, $CDCl_3$) δ 5.67 (s, 1H), 5.35 – 5.34 (m, 8H), 3.65 (s, 3H), 3.23 (dd, *J* = 12.9, 6.6 Hz, 2H), 2.82 – 2.75 (m, 4H), 2.33 (t, *J* = 7.2 Hz, 2H), 2.15 (t, *J* = 7.5 Hz, 2H), 2.07 – 2.01 (m, 6H), 1.65 – 1.61 (m, 4H), 1.53 – 1.50 (m, 2H), 1.39 – 1.35 (m, 2H), 1.34 – 1.27 (m, 6H), 0.87 (t, *J* = 6.7 Hz, 3H); ^{13}C NMR (125 MHz, $CDCl_3$) δ 173.93, 173.00, 130.43, 129.67, 128.49, 128.25, 128.04(2C), 127.83, 127.48, 51.53, 38.89, 36.63, 33.39, 31.45, 29.26, 29.19, 28.96, 27.15, 27.07, 26.87, 25.57(2C), 25.34, 22.51, 21.97, 14.01. HRMS (ESI): calculated for $C_{27}H_{44}NO_3$ (M-1) 430.3316, found

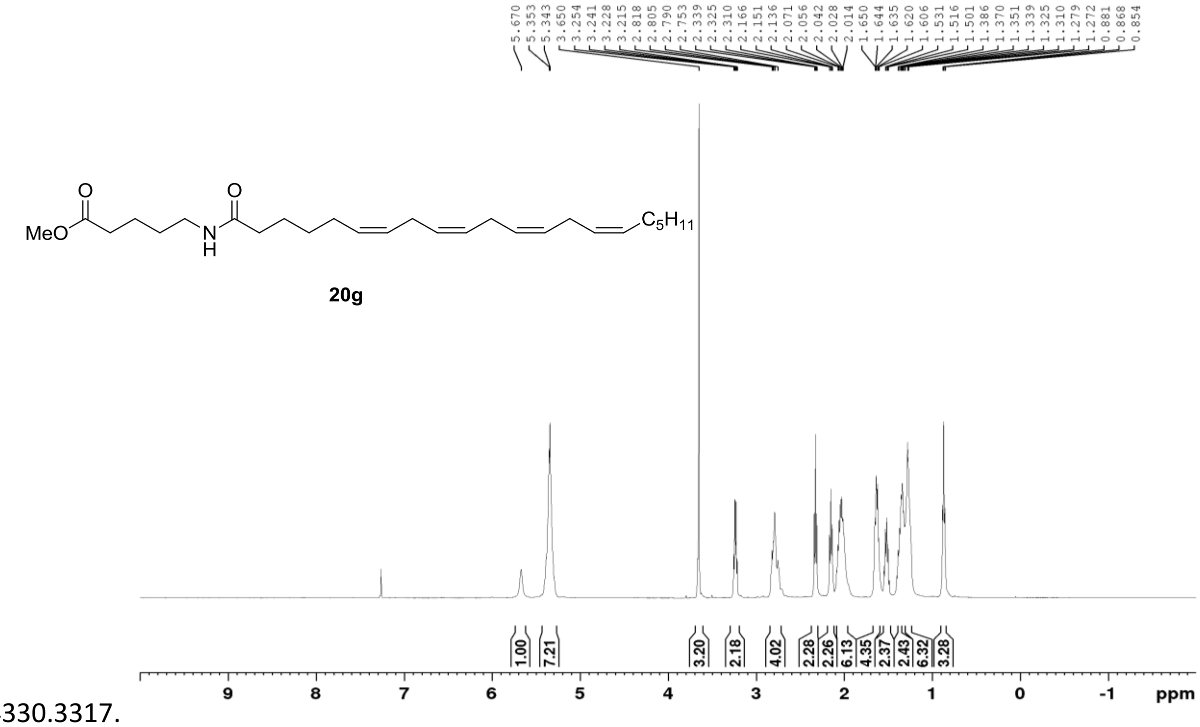

4330.3317.

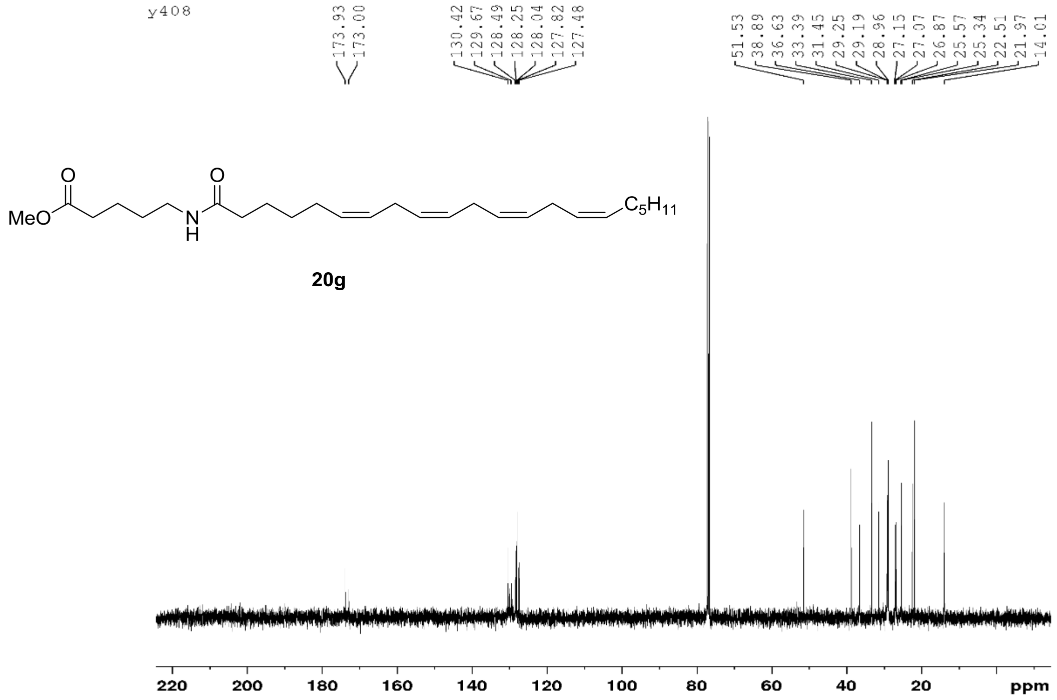

Methyl 5-((6Z,9Z,12Z,15Z)-nonadeca-6,9,12,15-tetraenamido)pentanoate (20h): Pale yellow solid, 41% yield (over 2 steps)， eluent system: 3:1 hexane-ethyl acetate. ^{1}H NMR (500 MHz, $CDCl_3$) δ 5.55 (s, 1H), 5.37 – 5.36 (m, 8H), 3.67 (s, 3H), 3.25 (dd, *J* = 12.8, 6.6 Hz, 2H), 2.83 – 2.80 (m, 6H), 2.34 (t, *J* = 7.2 Hz, 2H), 2.16 (t, *J* = 7.6 Hz, 2H), 2.09 – 2.03 (m, 4H), 1.67 – 1.64 (m, 4H), 1.53 (t, *J* = 7.0 Hz, 2H), 1.38 (q, *J* = 7.6 Hz, 4H), 0.91 (t, *J* = 7.4 Hz, 3H); ^{13}C NMR (125 MHz, $CDCl_3$) δ 173.92, 172.97, 130.20, 129.73, 128.56, 128.33, 128.12(2C), 127.90, 127.78, 51.53, 38.96, 36.70, 33.46, 29.30, 29.25, 29.05, 26.93, 25.65, 25.64(2C), 25.38, 22.75, 22.04, 13.75. HRMS (ESI): calculated for $C_{25}H_{41}NO_3Na$ (M+Na) 426.2979, found 426.2989.

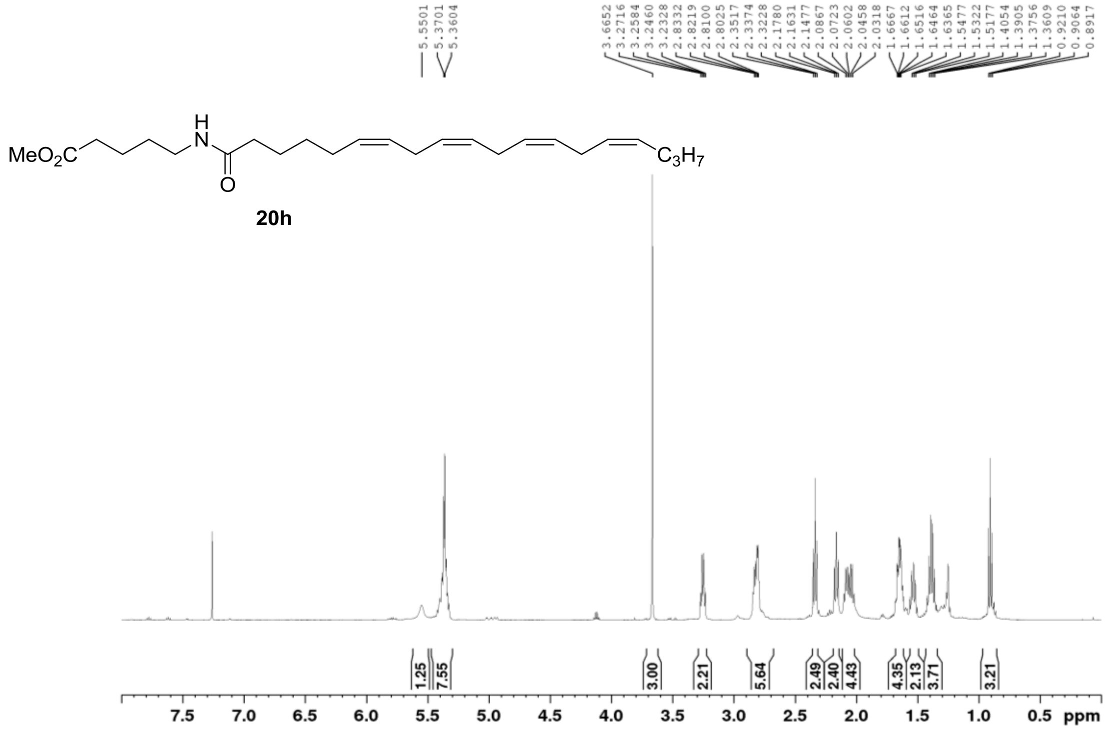

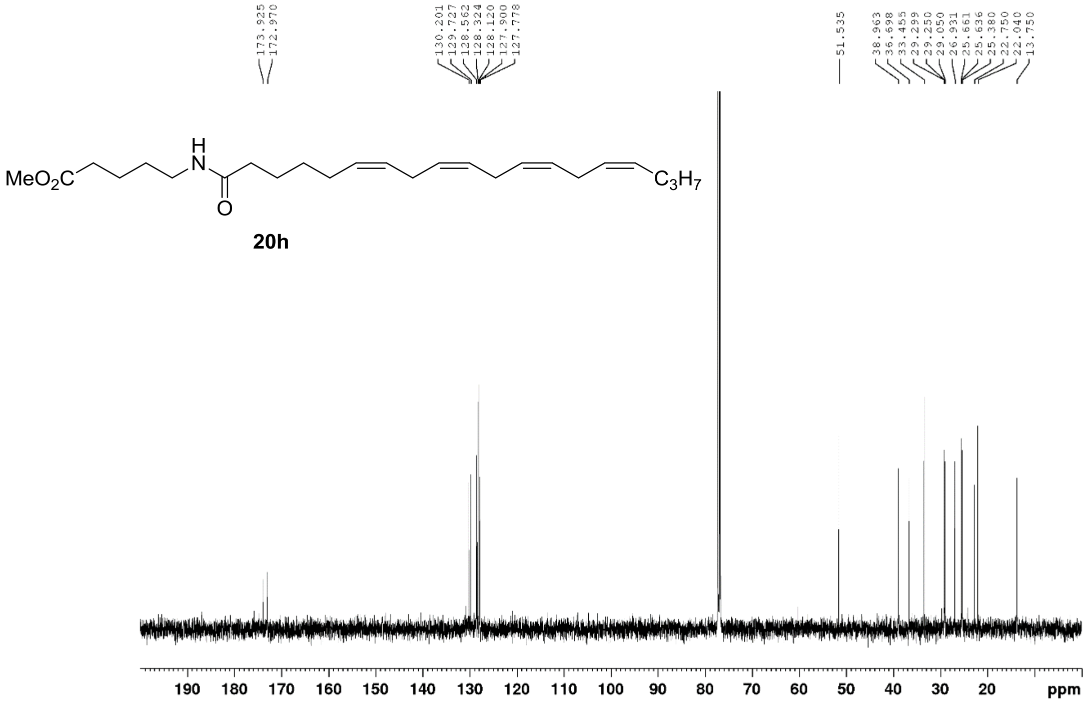

20h

Methyl 3-((6Z,9Z,12Z,15Z)-henicosa-6,9,12,15-tetraenamido)benzoate (20i): Pale yellow solid, 66% yield (over 2 steps), eluent system: 3:1 hexane-ethyl acetate. ^{1}H NMR (300 MHz, $CDCl_3$) δ 8.01 (s, 1H), 7.90 (d, *J* = 7.9 Hz, 1H), 7.77 (d, *J* = 7.8 Hz, 1H), 7.39 (t, *J* = 7.9 Hz, 1H), 5.41 – 5.35 (m, 8H), 3.91 (s, 3H), 2.86 – 2.79 (m, 6H), 2.38 (t, *J* = 7.5 Hz, 2H), 2.13 – 2.02 (m, 4H), 1.76 (dt, *J* = 15.3, 7.5 Hz, 2H), 1.44 (dt, *J* = 15.0, 7.5 Hz, 2H), 1.30 – 1.26 (m, 6H), 0.88 (t, *J* = 6.6 Hz, 3H); ^{13}C NMR (75 MHz, $CDCl_3$) δ 171.28, 166.69, 138.12, 130.91, 130.51, 129.57, 129.17, 128.59, 128.30, 128.27, 128.17, 127.88, 127.54, 125.25, 124.28, 120.53, 52.19, 37.59, 31.51, 29.31, 29.16, 27.22, 26.92, 25.65(3C), 25.09, 22.55, 14.03. HRMS (ESI): calculated for $C_{29}H_{41}NO_3Na$ (M+Na) 474.2979, found 474.2982.

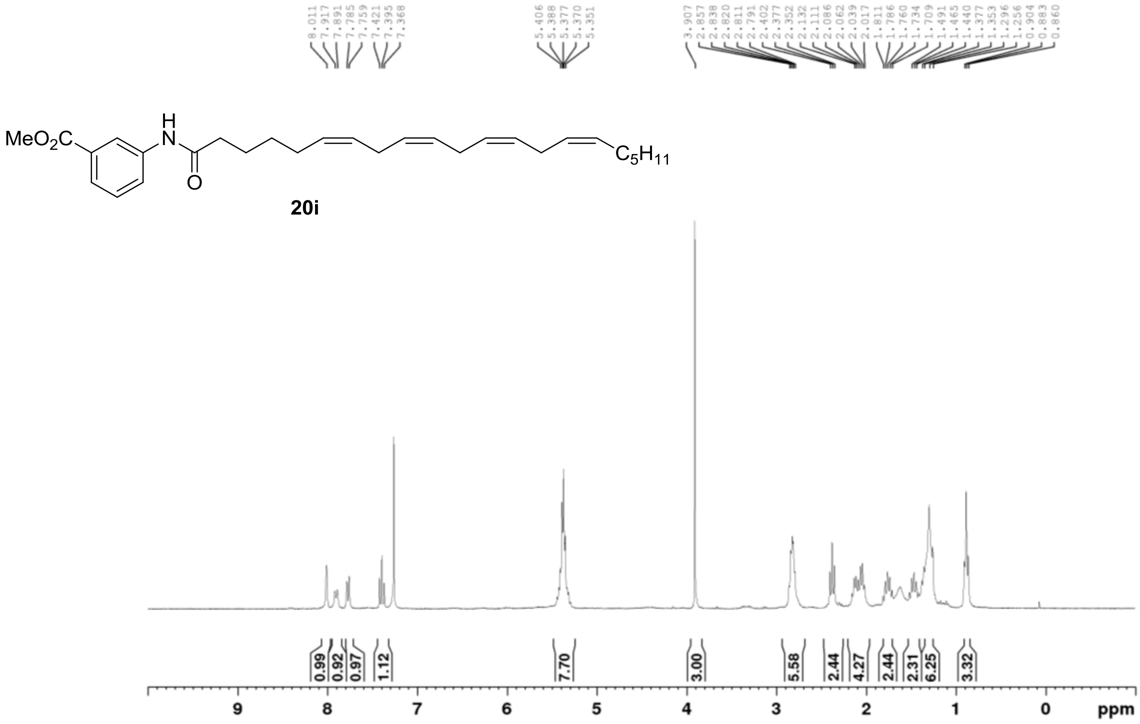

20i

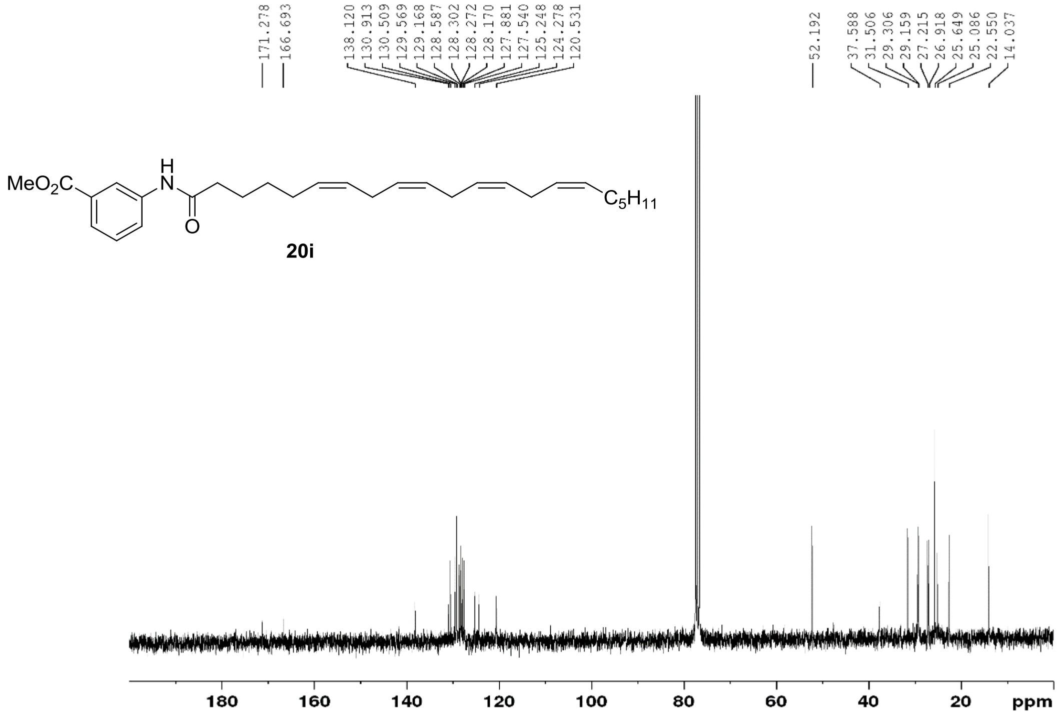

Methyl 3-((6Z,9Z,12Z,15Z)-nonadeca-6,9,12,15-tetraenamido)benzoate (20j): Pale yellow solid, 50% (over 2 steps), eluent system: 3:1 hexane-ethyl acetate.^{1}H NMR (500 MHz, $CDCl_3$) δ 8.01 (s, 1H), 7.91 (d, *J* = 7.6 Hz, 1H), 7.77 (d, *J* = 7.7 Hz, 1H), 7.39 (t, *J* = 7.9 Hz, 1H), 7.29 (s, 1H), 5.39 – 5.37 (m, 8H), 3.91 (s, 3H), 2.84 – 2.81 (m, 6H), 2.38 (t, *J* = 7.5 Hz, 2H), 2.12 (dd, *J* = 13.2, 7.1 Hz, 2H), 2.04 (dd, *J* = 14.1, 6.9 Hz, 2H), 1.76 (dt, *J* = 15.3, 7.6 Hz, 2H), 1.47 (dt, *J* = 15.1, 7.5 Hz, 2H), 1.38 (dd, *J* = 14.7, 7.3 Hz, 2H), 0.91 (t, *J* = 7.3 Hz,3H); ^{13}C NMR (125 MHz, $CDCl_3$) δ 171.25, 166.67, 138.16, 130.94, 130.22, 129.57, 129.16, 128.59, 128.32, 128.28, 128.18, 127.89, 127.78, 125.24, 124.30, 120.57, 52.17, 37.59, 29.31, 29.17, 26.92, 25.67(3C), 25.10, 22.76, 13.75. HRMS (ESI): calculated for $C_{27}H_{36}NO_3$ (M-1) 422.2701, found 422.2707.

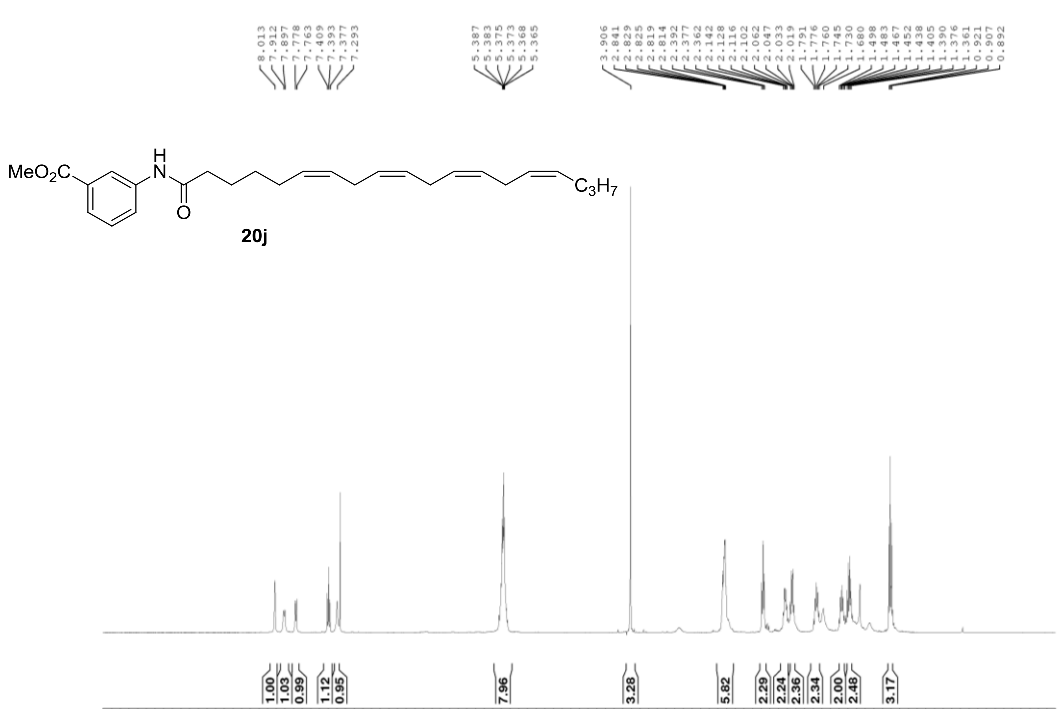

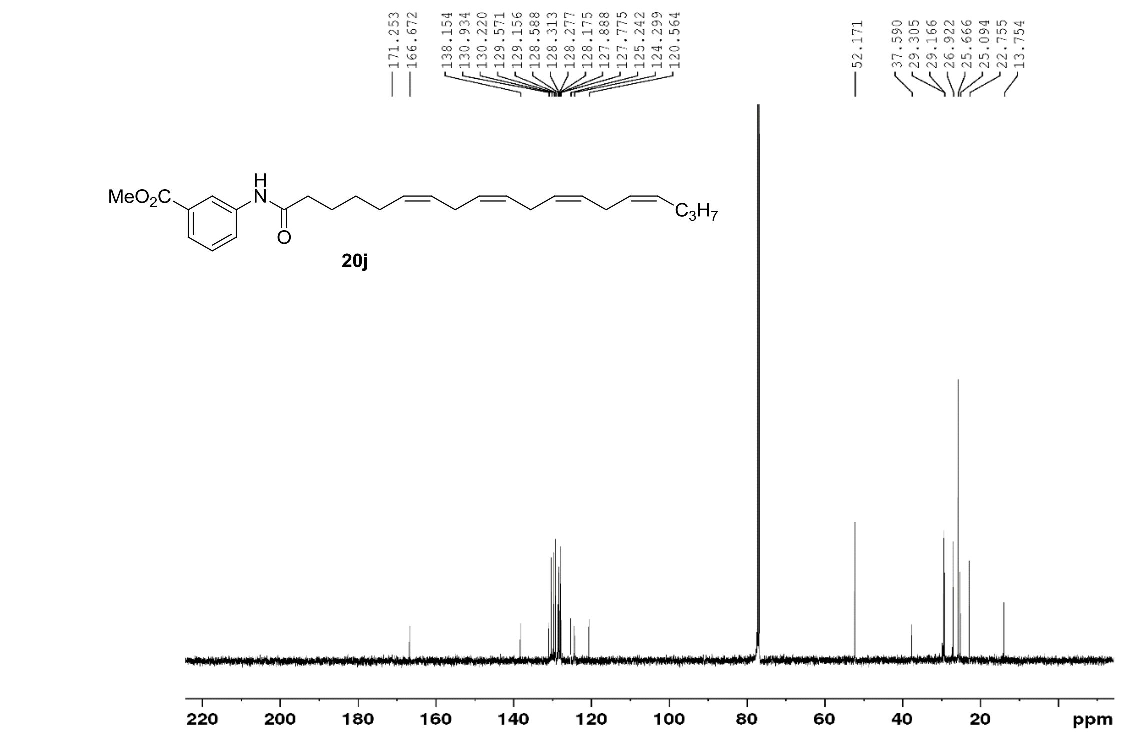

Methyl 5-((7Z,10Z,13Z,16Z)-docosa-7,10,13,16-tetraenamido)pentanoate (20k): Pale yellow solid, 47% yield (over 2 steps), eluent system: 3:1 hexane-ethyl acetate. ^{1}H NMR (500 MHz, $CDCl_3$) δ 5.56 (s, 1H), 5.38 – 5.36 (m, 8H), 3.67 (s, 3H), 3.28 – 3.25 (m, 2H), 2.84 – 2.80 (m, 6H), 2.34 (t, *J* = 7.2 Hz, 2H), 2.18 – 2.15 (m, 2H), 207 – 2.03 (m, 6H), 1.55 – 1.52 (m, 2H), 1.34 – 1.32 (m, 6H), 1.32 – 1.29 (dd, *J* = 11.6, 8.3 Hz, 4H), 1.28 – 1.27 (m, 2H), 0.89 (t, *J* = 6.9 Hz, 3H); ^{13}C NMR (125 MHz, $CDCl_3$) δ 178.76, 173.96, 130.49, 130.07, 128.55, 128.39, 128.07, 127.93, 127.88, 127.56, 51.57, 38.99, 36.77, 33.45, 31.51, 29.68, 29.34, 29.32, 29.04, 28.95, 27.22, 27.07, 25.67, 25.64, 25.56, 22.56, 22.02, 14.04, HRMS (ESI): calculated for $C_{28}H_{46}NO_3$ (M-1) 445.3556, found 445.3559.

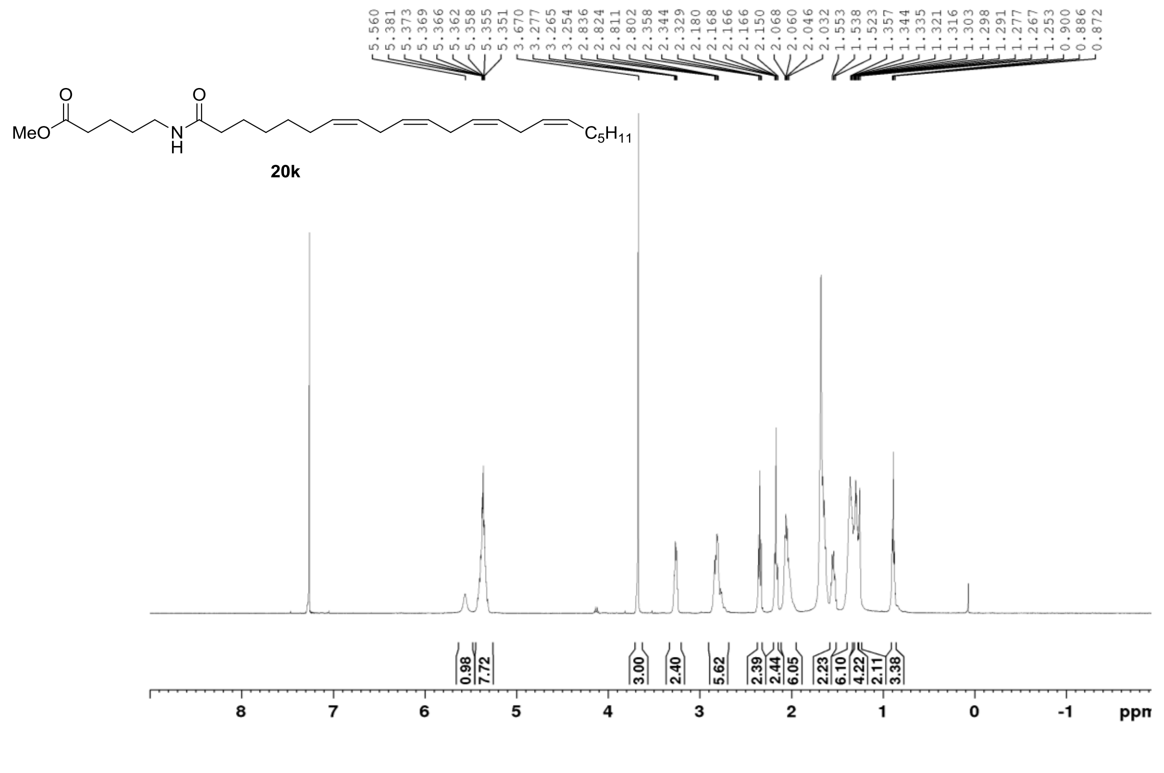

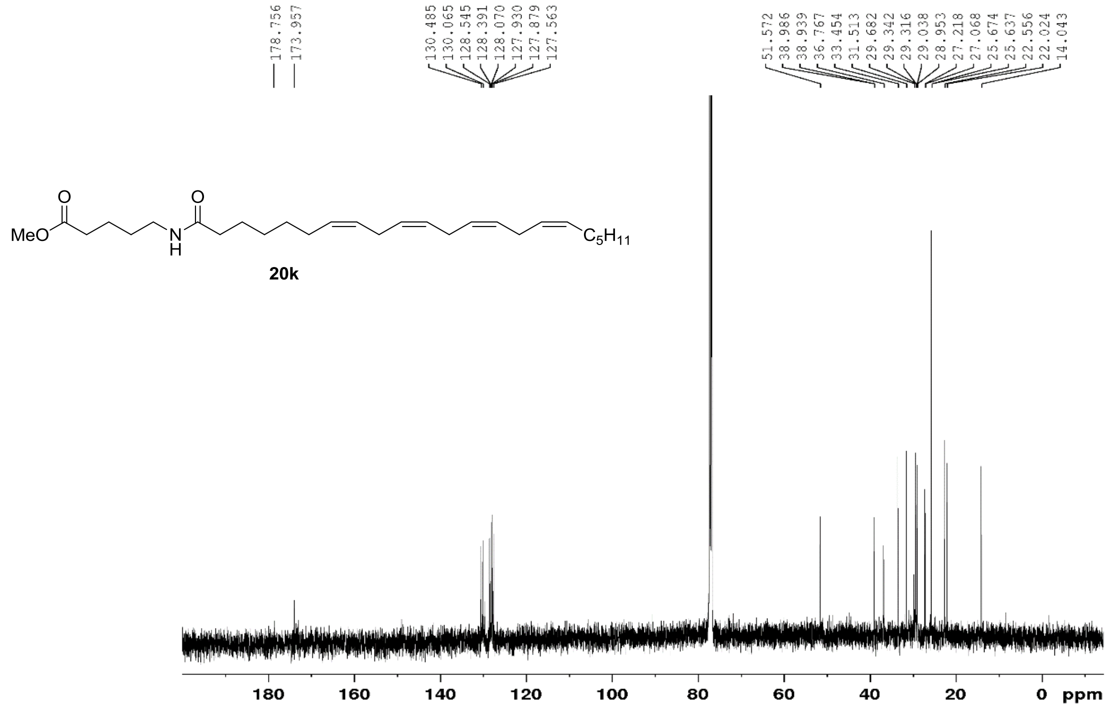

Methyl 4-((5Z,8Z,11Z,14Z)-icosa-5,8,11,14-tetraenamido)benzoate (20n): White solid, 64 % yield (over 2 steps), eluent system: 3:1 hexane-ethyl acetate. ^{1}H NMR (500 MHz, $CDCl_3$) δ 7.99 (d, *J* = 8.7 Hz, 2H), 7.59 (d, *J* = 8.5 Hz, 2H), 7.38 (s, 1H), 5.42 – 5.34 (m, 7H), 3.89 (s, 3H), 2.83 – 2.78 (m, 6H), 2.40 – 2.37 (m, 2H), 2.18 – 2.15 (m, 2H), 2.09 – 2.02 (m, 2H), 1.84 – 1.82 (m, 2H), 1.35 – 1.34 (m, 2H), 1.29 – 1.25 (m, 6H), 0.88 (t, *J* = 6.9 Hz, 3H); ^{13}C NMR (125 MHz, $CDCl_3$) δ 171.24, 166.58, 142.08, 130.83(2C), 130.53(2C), 129.12, 128.83, 128.62, 128.31, 128.04, 127.78, 127.48, 125.50, 118.68, 51.98, 37.04, 31.49, 29.29, 27.20, 26.50, 25.65, 25.62(2C), 25.09, 22.55, 14.04. HRMS (ESI): calculated for $C_{28}H_{38}NO_3$ (M-1) 436.2857, found 436.2838.

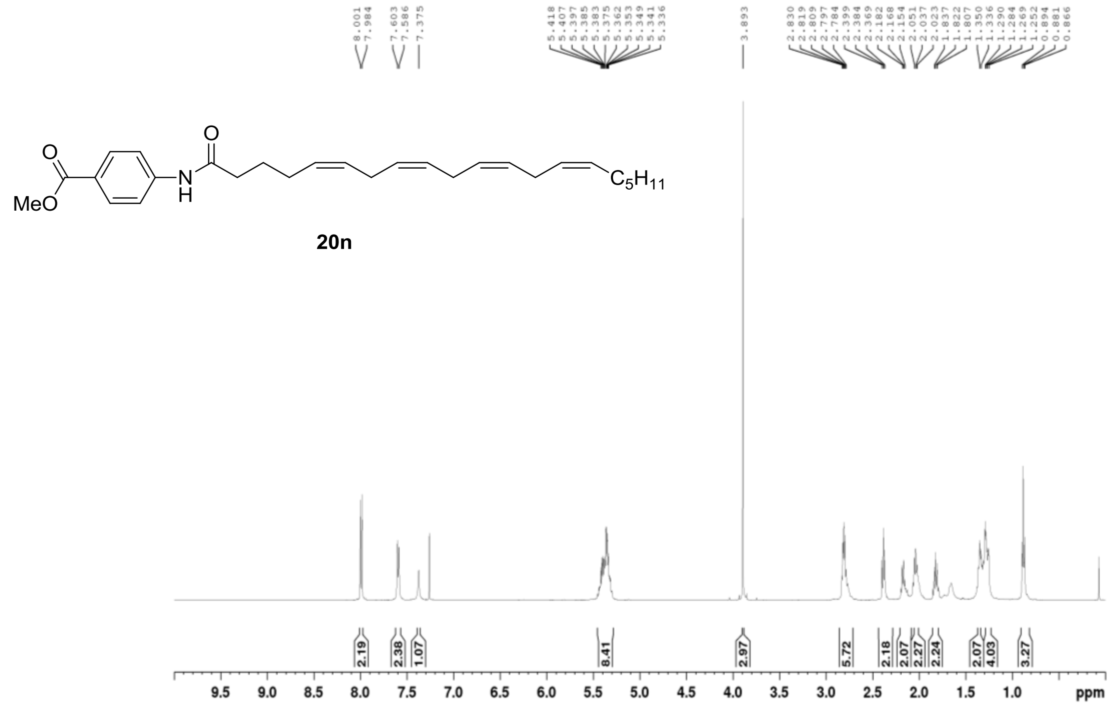

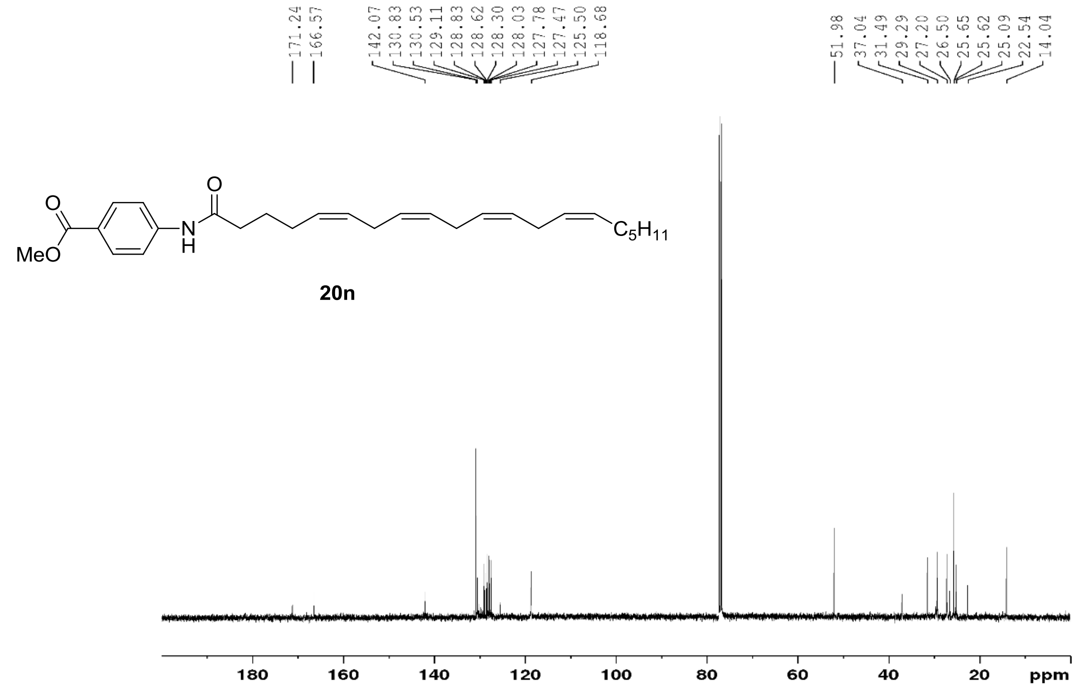

Methyl 2-((5Z,8Z,11Z,14Z)-icosa-5,8,11,14-tetraenamido)benzoate (20o): Pale yellow solid, 32% yield (over 2 steps), eluent system: 3:1 hexane-ethyl acetate. ^{1}H NMR (500 MHz, $CDCl_3$) δ 11.07 (s, 1H), 8.73 (d, *J* = 8.5 Hz, 1H), 8.03 (d, *J* = 8.0 Hz, 1H), 7.54 (t, *J* = 7.8 Hz, 1H), 7.07 (t, *J* = 7.6 Hz, 1H), 5.42 – 5.34 (m, 8H), 3.93 (s, 3H), 2.81 (dd, *J* = 11.1, 4.8 Hz, 6H), 2.46 (t, *J* = 7.6 Hz, 2H), 2.19 – 2.17 (m, 2H), 2.06 – 2.02 (m, 2H), 1.86 – 1.83 (m, 2H), 1.35 – 1.26 (m, 6H), 0.88 (t, *J* = 6.7 Hz, 3H); ^{13}C NMR (125 MHz, $CDCl_3$) δ 171.94, 168.77, 141.69, 134.69, 130.79, 130.47, 129.05, 128.90, 128.56, 128.21, 128.19, 127.89, 127.57, 122.31, 120.39, 114.75, 52.29, 38.01, 31.51, 29.69, 29.32, 27.21, 26.64, 25.66, 25.64, 25.30, 22.56, 14.05.HRMS (ESI): calculated for $C_{28}H_{38}NO_3$ (M-1) 436.2857, found 436.2856.

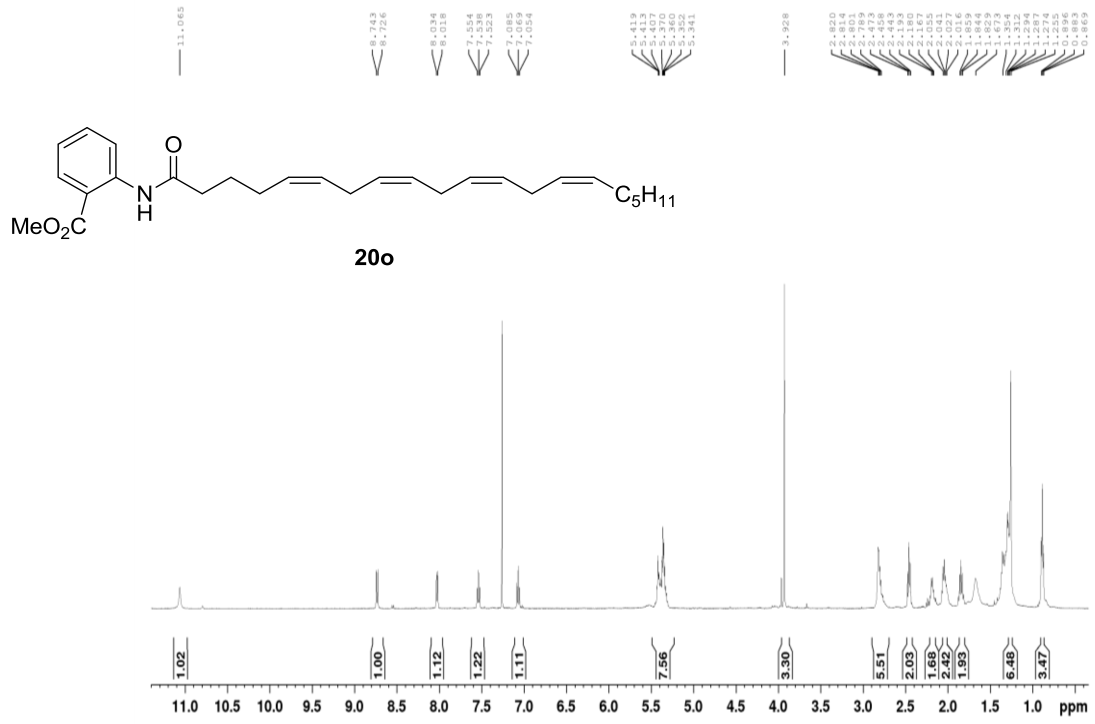

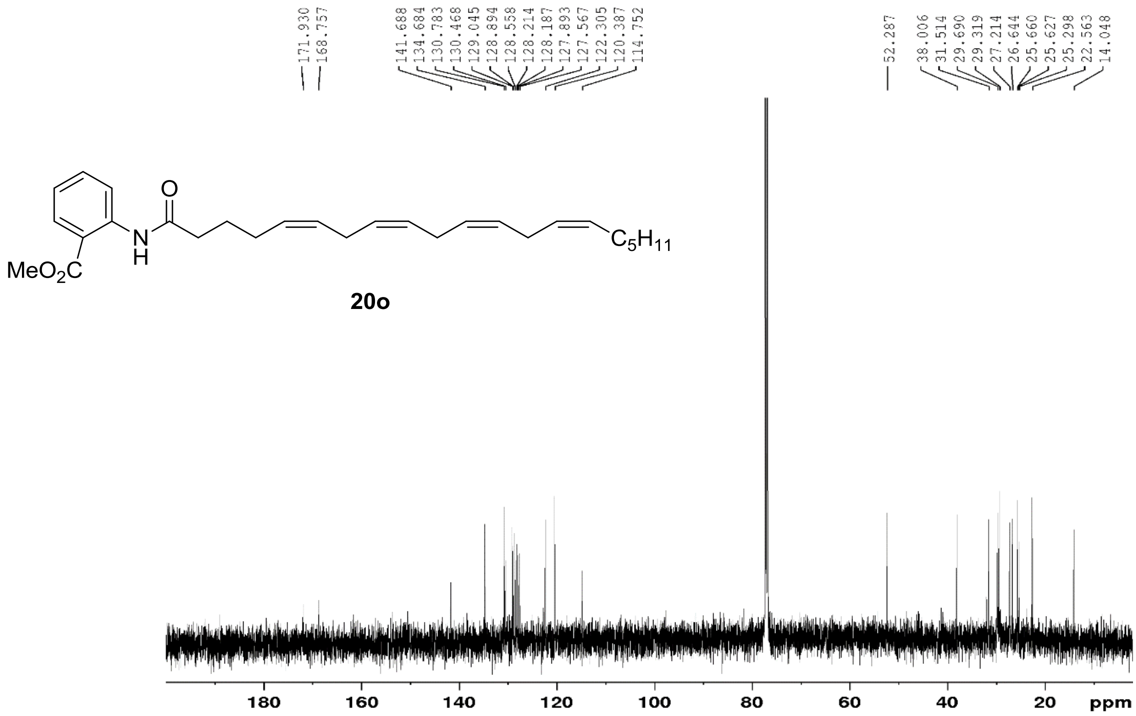

20o

Methyl 5-(pent-4-ynamido)pentanoate (22): Colourless liquid, 96% yield, eluent system: 4:1 hexane-ethyl acetate.^{1}H NMR (500 MHz, $CDCl_3$) δ 6.69 (s, 1H), 3.50 (s, 3H), 3.10 (dd, *J* = 13.0, 6.6 Hz, 2H), 2.35 (dd, *J* = 8.8, 4.7 Hz, 2H), 2.26 (t, *J* = 7.2 Hz, 2H), 2.18 (t, *J* = 7.3 Hz, 2H), 1.88 (t, *J* =2.6 Hz, 1H), 1.51 (t, *J* =7.4 Hz, 2H), 1.39 (t, *J* = 7.0 Hz, 2H); ^{13}C NMR (125 MHz, $CDCl_3$) δ 173.53, 170.93, 82.66, 68.90, 51.13, 38.65, 34.79, 33.13, 28.52, 21.75, 14.57. HRMS (ESI): calculated for $C_{11}H_{16}NO_3$ (M-1) 210.1136, found 210.1135.

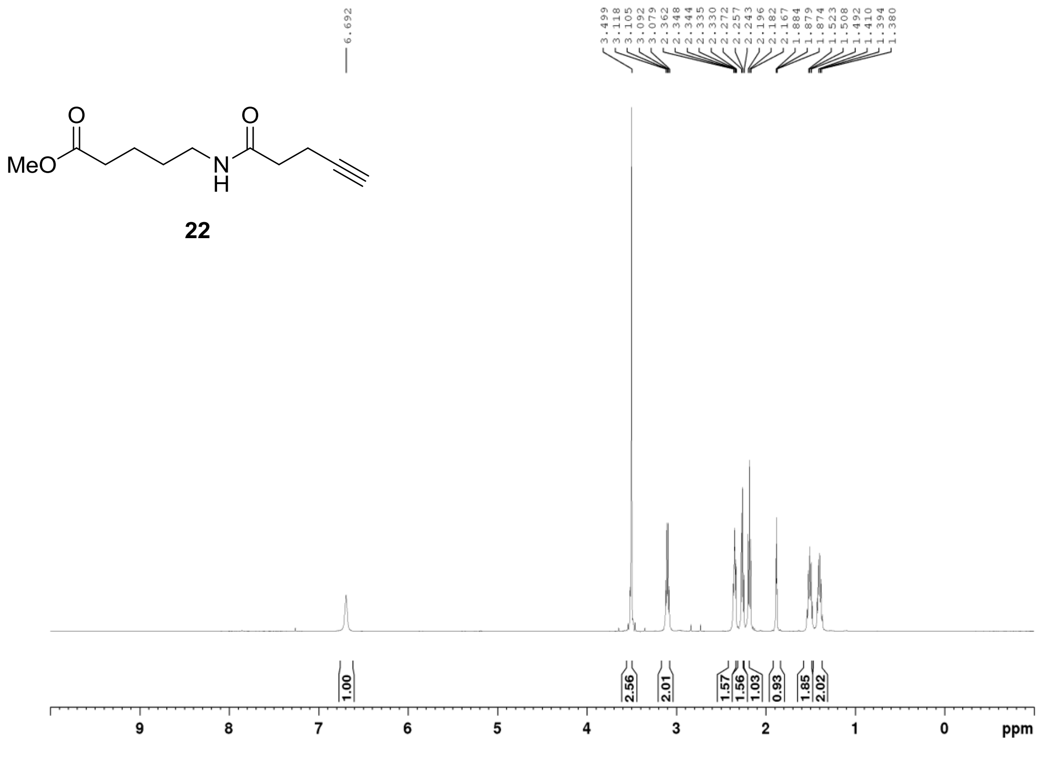

22

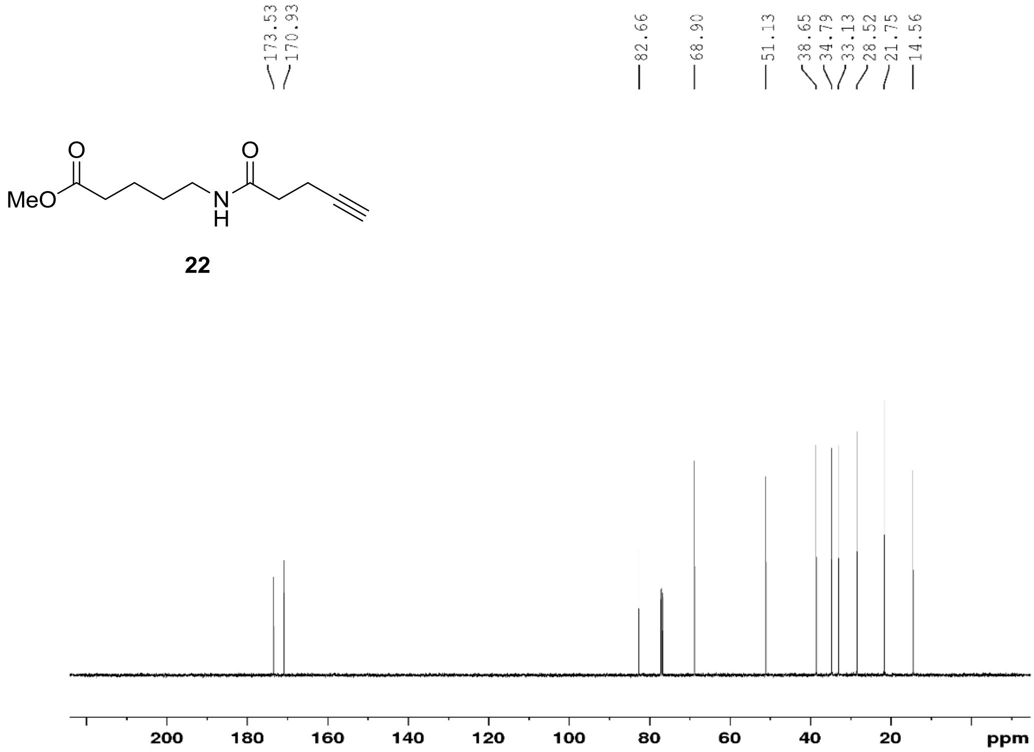

22

Methyl 5-((4Z,7Z,10Z,13Z)-nonadeca-4,7,10,13-tetraenamido)pentanoate (24): Pale yellow solid, 43% yield (over 2 steps), eluent system: 3:1 hexane-EA, ^{1}H NMR (500 MHz, DMSO) δ 7.77 – 7.76 (m, 1H), 5.35 – 5.30 (m, 7H), 3.57 (s, 3H), 3.02 – 3.01 (m, 2H), 2.82 – 2.74 (m, 4H), 2.29 (t, *J* = 7.4 Hz, 2H), 2.09 – 2.04 (m, 2H), 2.03 – 1.99 (m, 2H), 1.51 – 1.48 (m, 2H), 1.39 -1.36 (m, 2H), 1.31 – 1.30 (m, 4H), 1.26 – 1.23 (m, 6H), 0.85 (t, *J* = 6.7 Hz, 3H); ^{13}C NMR (125 MHz, DMSO) δ 173.20, 171.19, 129.92, 128.86, 128.08, 128.03, 128.00, 127.74, 127.66, 127.50, 51.13, 37.91, 35.21, 32.88, 30.85, 28.69, 28.53, 26.57, 25.18, 25.17, 25.13, 23.16, 21.94, 21.84, 13.88. HRMS (ESI): calculated for $C_{25}H_{41}NO_3Na$ (M+Na) 426.2979, found 426.2995.

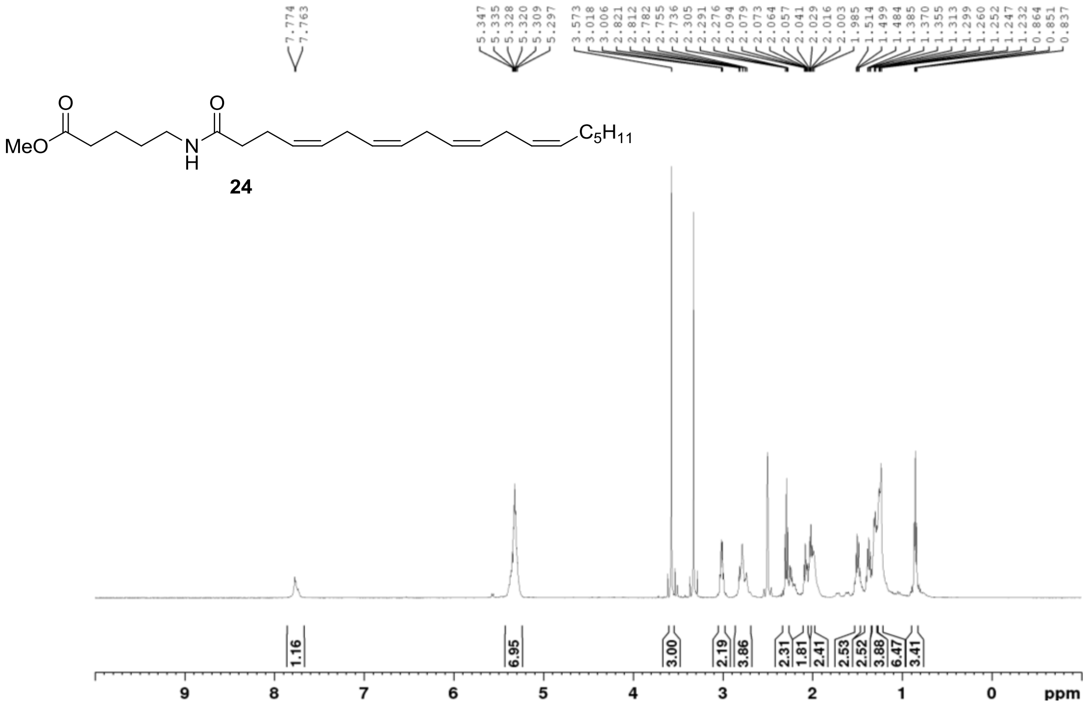

24

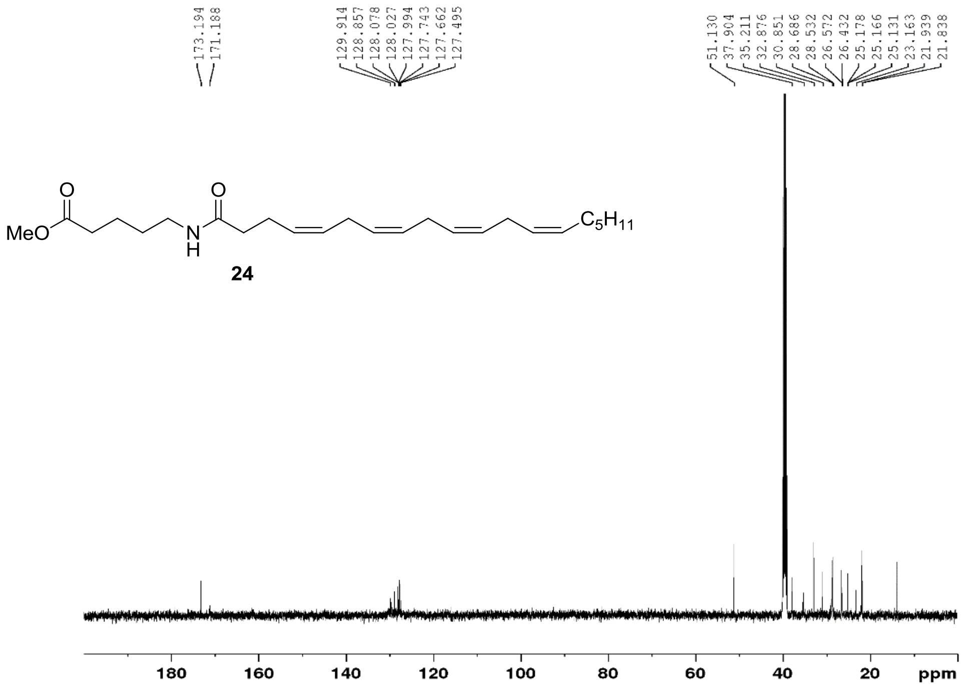

24

Docking. Protein modeling. The crystal structure of $cPLA_2$ in its apo form (PDB ID 1CJY, resolution 2.5Å) is available[4-5], however the experimental structure has a few missing regions (residues 407 to 414, 431 to 462, 498 to 538 and 626-632). The missing structural regions were modeled using homology and loop modeling methods. These models were generated using the program Modeller (version 9.12)[6]. Several models were generated and the models with the best stereochemical properties were refined further using all atom MD simulations.

Ligand preparation. The 3D structures of the test compounds were built using *Maestro* and minimized using the *Macromodel* module employing the OPLS-2005 force field in Schrodinger 9.0[7-8]. All the inhibitors were then prepared with *Ligprep* that generates low energy tautomers and enumerates realistic protonation states at physiological pH.

Ligand docking. The prepared inhibitors were docked into the binding pockets of the models of $cPLA_2$ using *Glide*[9]. A box of size 10 x 10 x 10 Å for molecular docking centered on the selected active site residue (Ser228) was used to confine the search space of each docked ligand. For the grid generation, the default *Glide* settings were used. A rigid receptor docking (RRD) protocol was used which fixes the protein conformation while allowing the ligands to be flexible. All inhibitors were docked into the active sites of $cPLA_2$ using this protocol and the docked conformation of each ligand was evaluated using the *Glide* Extra Precision (XP) scoring function. Docking was carried out on several conformational substrates of $cPLA_2$ identified by clustering of MD trajectories. The partial charges and force field parameters for each inhibitor

were generated using the *Antechamber* module in Amber. All atom versions of the Amber 03 force field (ff03) and the general Amber force field (GAFF)[10] were used for the protein and the inhibitors respectively.

Western blots

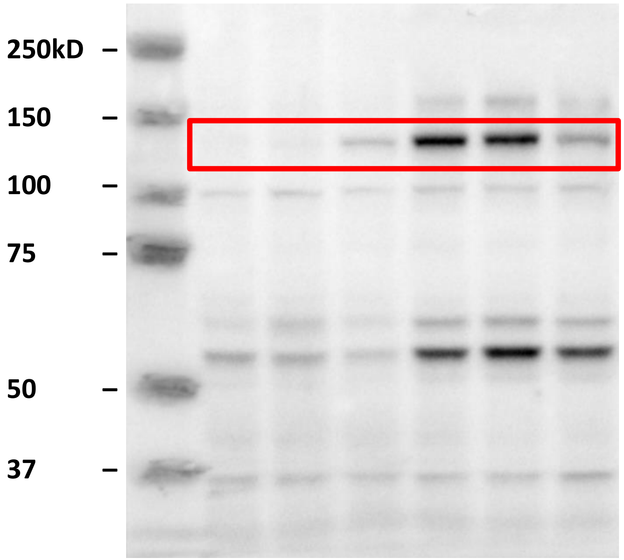

INOS

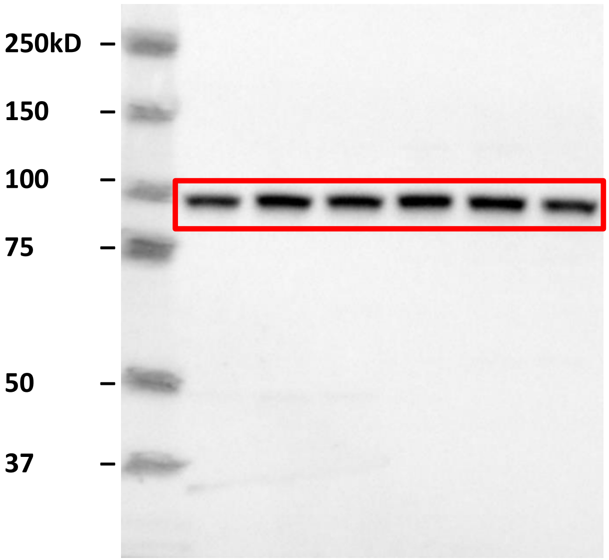

$cPLA_2$

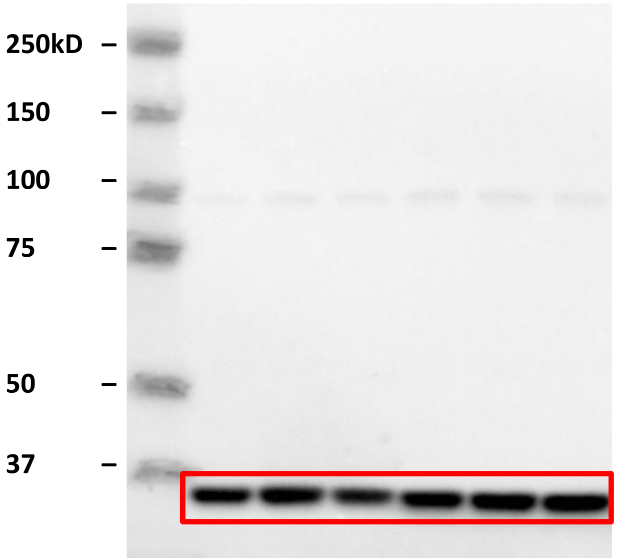

GAPDH

Figure S2: Images of blots presented in Figure 3

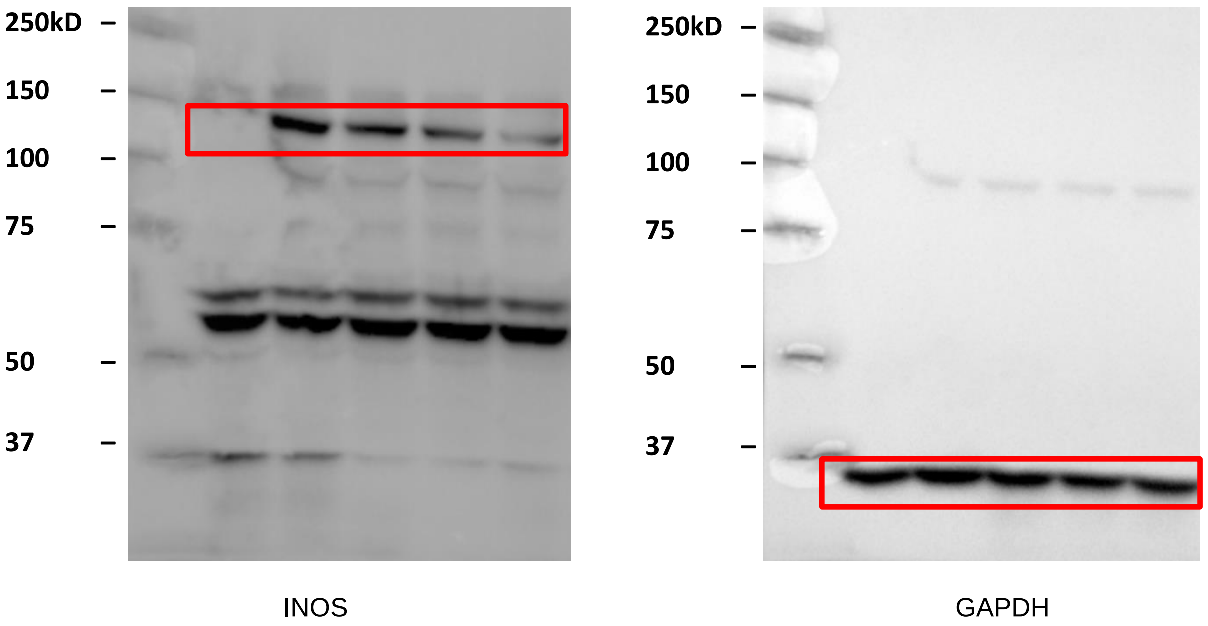

Figure S3: Images of blots presented in Figure 4

References:

1. Qi, L., Meijler, M.M., Lee, S.H., Sun, C. & Janda, K.D. Solid-phase synthesis of anandamide analogues. *Org. Lett*. **6**, 1673-1675 (2004).
2. Ackermann, E.J., Conde-Frieboes, K. & Dennis, E.A. Inhibition of macrophage Ca^{2+} -independent phospholipase A2 by bromoenol lactone and trifluoromethyl ketones. *J. Biol. Chem*. **270**, 445-450 (1995).
3. Yoshida, T., Murai, M., Abe, M. , Ichimaru, N., Harada, T., Nishioka, T., Miyoshi, H. *Biochemistry* **2007**, 10365-10372.
4. Mouchlis, V.D. Michopoulout, V., Constantinou-Kokotou, V., Mavromoustakos, T., Dennis, E. A., Kokotos, G. Binding conformation of 2-oxoamide inhibitors to Group IVA cytosolic phospholiase A2 determined by molecular docking combined with molecular dynamics. *J. Chem. Inf. Model.* **52**, 243-254 (2012).
5. Yu, L., Deems, R.A., Hajdu, J. & Dennis, E.A. The interaction of phospholipase A_2 with phospholipid analogues and inhibitors. *J. Biol. Chem.* **265**, 2657-2664 (1990).
6. Sali, A. & Blundell, T.L. Comparative protein modelling by satisfaction of spatial restraints. *J. Mol. Biol.* **234**, 779-815 (1993).

7. Kaminski, G.A., Friesner, R.A., Tirado-Rives, J. & Jorgensen, W.L. Evaluation and reparametrization of the OPLS-AA force field for proteins via comparison with accurate quantum chemical calculations on peptides*. J. Phys. Chem. B* **105**, 6474-6487 (2001).

8. *Schrodinger*, version 9.0, Schrödinger, LLC, New York, NY, 2009.

9. Friesner, R.A. *et al*. Glide: a new approach for rapid, accurate docking and scoring.1. Method and assessment of docking accuracy. *J. Med. Chem.* **47**, 1739**-**1749 (2004).

10. Wang, J. *et al*. Development and testing of a general amber force field. *J. Comput. Chem.* **25**, 1157**-**1174 (2004).
